# Dienedioic acid as a useful diene building block via directed Heck-decarboxylate coupling

**DOI:** 10.1038/s42004-020-0295-0

**Published:** 2020-04-20

**Authors:** Lei Ke, Zhilong Chen

**Affiliations:** School of Pharmacy, Huazhong University of Science and Technology (HUST), 13 Hangkong Road, Wuhan, Hubei 430030 P. R. China

**Keywords:** Synthetic chemistry methodology, Medicinal chemistry

## Abstract

The concise construction of diene scaffolds is quite useful in the synthesis of polyenes. Many diene building blocks have been developed based on Suzuki, Still and Hiyama couplings. Herein, the commercially available and environmentally friendly compound dienedioic acid is used as a diene building block. Broad substrate scope, good functional group tolerance, and late-stage derivatization of complex drug molecules are achieved. Different moieties can be conveniently introduced to both sides. Piperine and the methyl ester of azoxymycin C are each prepared in three steps. Additionally, one product shows promising anticancer activities in leukemia K562 and MV-4-11 cells. Mechanistic studies indicate that the reaction proceeds through a Heck-decarboxylate coupling procedure, and the carboxylic group acts as a directing group to promote the reaction and control regioselectivity. Our research suggests that dienedioic acid can serve as a good alternative for diene preparation via a directed Heck-decarboxylate coupling.

## Abstract (web summary)

Dienes are useful reagents for cross-couplings in the synthesis of unsaturated natural products, but decarboxylative Heck reactions of dienes are limited by facile *E/Z* isomerism. Here configurationally stable divinyldicarboxylic acid is used as a coupling reagent in which the carboxylates direct the Heck reaction prior to decarboxylation.

## Introduction

**Fig. 1:**
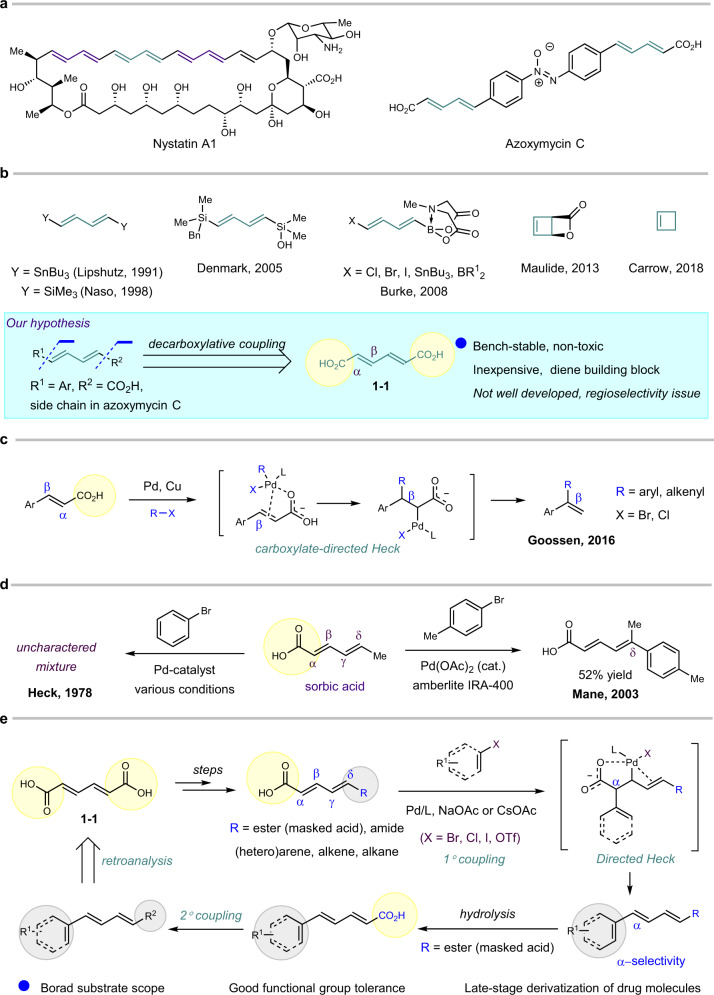
Representatives of polyene compounds, diene-building blocks, and our hypothesis. **a** Representive of natural products containing diene moieties. **b** Representative reported diene-building blocks and our hypothesis of utilizing dienedioic acids as diene-building blocks. **c** Carboxylate-directed Heck-decarboxylation coupling with vinyl carboxylic acid^25^. **d** Previous Heck coupling in dienoic acid by Heck^30^ and Mane^31^. **e** Reaction design and mechanism hypothesis.

Dienes are a commonly encountered and important motif in natural products, organic dyes, and medicinal chemistry. Polyene scaffolds can be viewed as conjugation of one or several diene motifs as shown in the antibiotic compound nystatin A1^1^ and unusual azoxy alkaloid azoxymycin C^2^ (Fig. 1a). From the viewpoint of retrosynthetic analysis, all of these diene or polyene moieties can be prepared from a diene-building block via dual-coupling procedure. Many excellent diene-building blocks have been developed, like *bis*-trimethylstannylbutadiene^3^, *bis*-trimethylsilylbutadiene^4^, diene silanols^5^, and diene–methyliminodiacetic acid (MIDA) boronate^6^. Cyclobutenes have also been used to introduce diene scaffolds in a ring-opening fashion^7,8^. Albeit such significant progress has been achieved, there is still room for improvement, particularly considering that these well-established building blocks suffer from either being not commercially available or requiring multiple steps to prepare or being toxic. Therefore, additional efforts are worthy to develop novel, economically available, and environment-friendly diene-building blocks (Fig. 1b).

As one part of our continuing efforts in azoxy research^9^, we are quite interested in azoxymycin C^2^ for its unique structure. Within our established tungstate-catalyzed azoxy formation approach^9^, a challenging step would be rapidly installing the diene motifs. It is not difficult to design a synthetic route to introduce the diene motif in azoxymycin C via Heck coupling from pentadienoic acid. However, this compound is relatively expensive and not bench-stable as an E/Z mixture. Decarboxylative coupling emerges as a good approach for cross-coupling reactions^10–18^, including C–C, C–O^19^, and C–N^20–22^ bond formation. From this perspective, in theory, the dienedioic acids, like *trans*–*trans*-muconic acid (**1–1**), can serve as good complementary diene-building blocks by decarboxylative coupling, particularly considering that it is a commercially available, cost friendly, and bench-stable white solid (Fig. 1b).

Although vinyl carboxylic acid has been investigated in decarboxylative coupling^23–28^, the dienedioic acid has never been explored to the best of our knowledge. As studied by Goossen^25^, the decarboxylative coupling of vinyl acids favored the β-selectivity in the presence of Pd/Cu with aryl bromide and chloride through a deciduous carboxylate-directed mechanism^25,29^, in sharp contrast to the α-selectivity with aryl iodide observed by Wu^23^ and Miura^27,28^ (Fig. 1c). In addition, from the inherent properties of dienoic acid, such as sorbic acid, the α-selective product is not favored in normal Heck coupling as observed by Heck^30^ and Mane^31^ (Fig. 1d). Accordingly, to obtain the desired α-selective product with dienedioic acid, **1–1** is quite challenging: (1) the β-selectivity product might dominate the reaction under carboxylate-directed Heck-coupling mechanism; (2) the innate steric and electronic properties of dienedioic acid also favored the β- or γ-selectivity.

Herein we report that the dienedioic acid can serve as a good complementary building block in diene synthesis, through the directed Pd-catalyzed decarboxylative coupling mechanism. Broad substrate scope, good functional group tolerance, and late-stage functionalization of complex drug molecules are achieved in this reaction (Fig. 1e).

## Results

### Condition optimization for directed Heck-decarboxylative coupling of dienedioic acid

Compounds **1–2** and **2–1** were selected as model substrates for condition optimization. Initially, P(tol)_3_ was selected as ligand according to Heck’s^30^ and Goossen’s reports^25^, and luckily the desired product **3–1** was isolated in unsatisfactorily 14% yield (Table 1, entry 1). The ligand played a significant role in this reaction. Replacing P(tol)_3_ with electronically rich and steric-hindered PCy_3_ increased the efficiency a little bit (entry 2). Significant improvements were achieved with bidentate ligands, like dppf and dppe (Table 1, entries 3–4). By contrast, trace amount of product was observed with dppp (Table 1, entry 5), and evident erosion of efficiency was also observed in the presence of dppb, (*R*)-BINAP and XantPhos (Table 1, entries 6–8). The bulky and electronically rich phosphines, developed by Buchwald, also promoted the reaction, such as XPhos (Table 1, entry 9) and DavePhos (Table 1, entry 11). But BrettPhos failed to afford the desired product. Since such electronic-rich ligands even work well when coupling with aryl chloride, further condition optimization were focused on them. Switching the base from NaOAc to CsOAc in the presence of XPhos-G2 afforded the product in the same isolated yield as those of dppf and dppe. Under Miura’s condition, the conversion of the reaction was very low without product **3–1** detected. But increasing catalyst loading to 9% facilitates the reaction, only affording 20% yield of the product by H-NMR (Table 1, entry 13).

**Table 1:** Condition optimization for Heck-decarboxylative coupling of dienedioic acid.

^a^All the reactions were conducted in **2–1** (0.25 mmol, 1.0 equivalent), **1–2** (0.375 mmol, 1.5 equivalent), and NaOAc (0.375 mmol, 1.5 equivalent) isolated yield.

^b^Yield determined by H-NMR with CH_2_Br_2_ as internal standard.

^c^Miura’s condition: Pd(OAc)_2_ (3 mol%), LiOAc (2.0 equivalent), and LiCl (1.5 equivalent).

^d^Pd(OAc)_2_ (9 mol%), LiOAc (2.0 equivalent), and LiCl (1.5 equivalent). N. D. = no desired product.

### Evaluation of aryl halides as a coupling partner

**Fig. 2:**
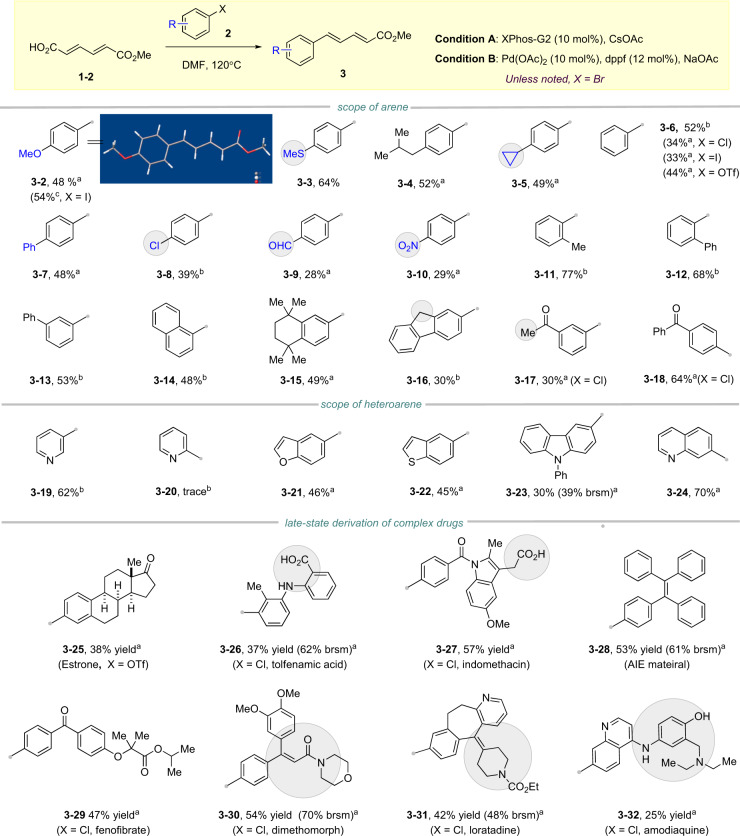
Scope of aryl electrophile in Heck-decarboxylate coupling of dienedioic acid. All the reactions were conducted in **2** (0.25 mmol, 1.0 equivalent), **1–2** (0.375 mmol, 1.5 equivalent), CsOAc, or NaOAc (0.375 mmol, 1.5 equivalent) isolated yield. ^a^Under condition A: with XPhos-G2 (10 mol%) and CsOAc. ^b^Under condition B: with Pd(OAc)_2_ (10 mol%), dppf (12 mol%), and NaOAc.

Within the optimized condition at hand, the scope of aryl electrophiles was investigated as summarized in Fig. 2. Diverse functional groups were tolerated, including methoxyl (**3–2**)/methylthio (**3–3**)/alkyl (**3–4**, **3–5**, **3–16**)/aryl (**3–7**, **3–12**, **3-13**)/cyclopropyl group (**3–5**), aldehyde (**3–9**), nitrone (**3–10**), as well as ketone (**3–18**, **3–19**). Aryl bromide, chloride, iodide, and trifluoromethanesulfonate worked well in this reaction, affording comparable yields based on the substrates (**3–2, 3–6**). Generally, the aryl bromides bearing electronically neutral or rich groups afforded better yields compared with those bearing electronically deficient ones. Notably, selective coupling with aryl bromide in the presence of aryl chloride was achieved with dppf as ligand (**3–8**, condition B). Steric hindrance was also well tolerated. For example, 2-bromo-1,1’-biphenyl even gave better yield than bromobenzene (**3–11** vs. **3–6**). 9*H*-fluorene could afford the desired product **3–16** as well, albeit that possible Michael addition might be involved^32^. Heteroarenes are frequently encountered in medicinal chemistry, and it is important to develop novel access to their functionalization. Therefore, several heteroaryl bromides were selected and tested in this reaction, including benzofuran (**3–20**), benzothiophene (**3–21**), pyridine (**3–22**), carbazole (**3–23**), and quinoline (**3–24**), all of which afforded the desired products as expected. Of note, pyridine (**3–19**) and isoquinoline (**3–24**) bearing Lewis basic nitrogen can be well tolerated, although they could coordinate to palladium. However, 2-bromopyridine failed to afford the desired coupling product (**3–20**).

The late-stage functionalization of complex molecules is quite useful in drug discovery and complex molecule synthesis. Given the good functional group tolerance obtained previously, it is reasonable to hypothesize that late-stage installing diene motif via our method should work as well. Indeed, estrone can smoothly afford the desired product (**3–25**) in a two-step sequence. Anti-inflammation drugs tolfenamic acid (**3–26**) and indomethacin (**3–32**) also gave coupling products in moderate yield, albeit that they contained an aryl and alkyl carboxylic acid moiety, respectively, demonstrating the high selectivity among vinyl, aryl, and alkyl carboxylate in our approach. Pesticide dimethomorph (**3–29**) and antihistamine drug loratadine (**3–30**) bearing alkene motifs, successfully afforded the products as well. The installation of diene moiety on aggregation-induced emission (AIE) material was also proved successful (**3–28**). Remarkably, even amodiaquine, a drug to cure malaria, containing phenol and amine moieties, worked smoothly in this reaction (**3–31**).

### Evaluation of dienedioic acid in dual coupling

**Fig. 3:**
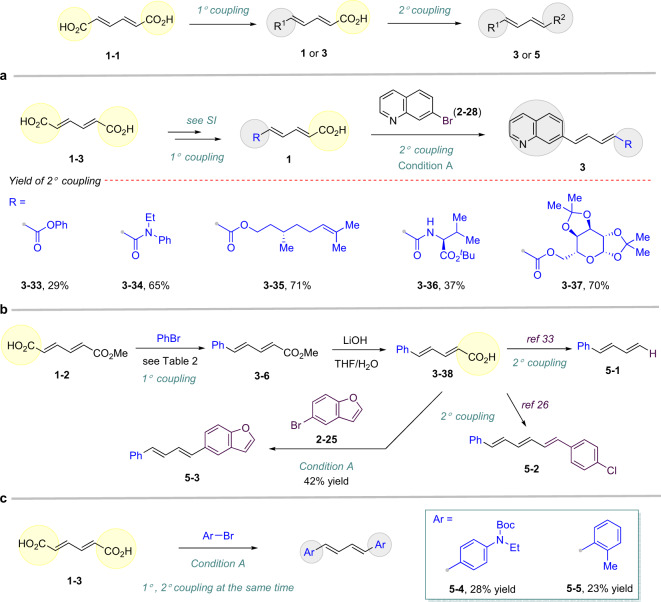
Dual coupling of *trans*–*trans*-muconic acid (**1–1**) to afford complex diene products. All the reactions during the coupling step were conducted according to general procedure A as shown in Fig. 2 (Supplementary Fig. 3), isolated yield. **a** Two-step sequential coupling: late decarboxylative coupling in the second step. **b** Two-step sequential coupling: late decarboxylative coupling in the first step (Supplementary Fig. 20). **c** One-step dual decarboxylative coupling (Supplementary Fig. 21).

In theory, various coupling partners can be installed on both sides of dienedioic acid either by a two-step sequential or one-step dual-coupling procedure as summarized in Fig. 3. Thus, three types of synthetic applications were developed based on the sequence of installing aryl motifs: (1) late coupling of aryl halides; (2) early coupling of aryl halides; (3) dual coupling of aryl halides spontaneously. For instance, the conventional coupling of carboxylate of dienedioic acid **1–3** can proceed first, such as formation of ester, amide, and so on. Notably, even complex partners were tolerated well, including diacetone-*D*-galactose (**3–37**), *L*-valine t-butyl ester (**3–36**), and β-citronellol (**3–35**) in the following decarboxylative coupling. Simple benzo ester and amide also afford the desired products (**3–33**, **3–34**). The dienedioic acid **1–2** and bromobenzene can also provide compound **3–6** via first decarboxylative coupling, followed by hydrolysis (**3–38)**. From compound **3–38**, various transformations can take place based on the second decarboxylative coupling, like protodecarboxylation (**5–1**)^33^, introducing a vinyl moiety (**5–2**)^26^, and installing a (hetero)arene motif (**5–3**). The two (hetero)aryl scaffolds can be installed via a dual coupling as well, affording the 1,4-di(hetero)aryl-buta-1,3-diene (**5–4**, **5–5**).

### Evaluation of other substrates

**Fig. 4:**
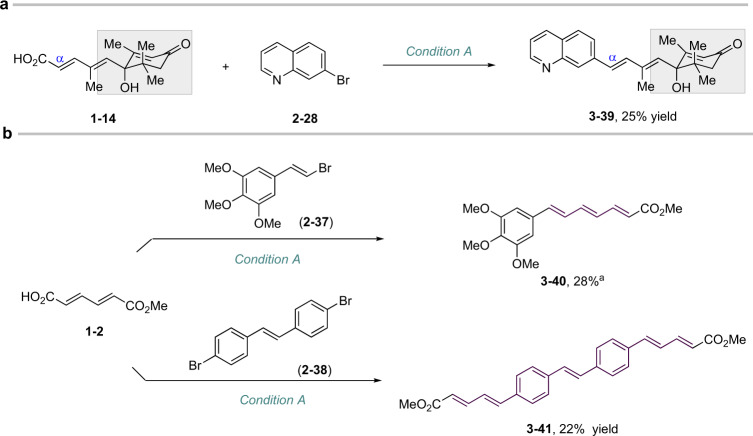
Other coupling reactions. **a** Late-stage coupling of abscisic acid. **b** Vinyl bromide as a coupling electrophile and preparation of a large π-conjugation system.

Of note, even the abscisic acid, bearing multiple functional groups, afforded the desired product **3–39**, particularly taking into account that many possible side reactions can be involved (Fig. 4a). The vinyl electrophiles also work well in this reaction, like vinyl bromide **2–37**, giving a triene product. However, construction of tertene from dieneoic bromide was proven unsuccessful. In addition, large π-conjugation scaffolds can be conveniently accessed by our approach, such as installing two diene motifs from compound **2–38** (Fig. 4b).

### Application in natural products

**Fig. 5:**
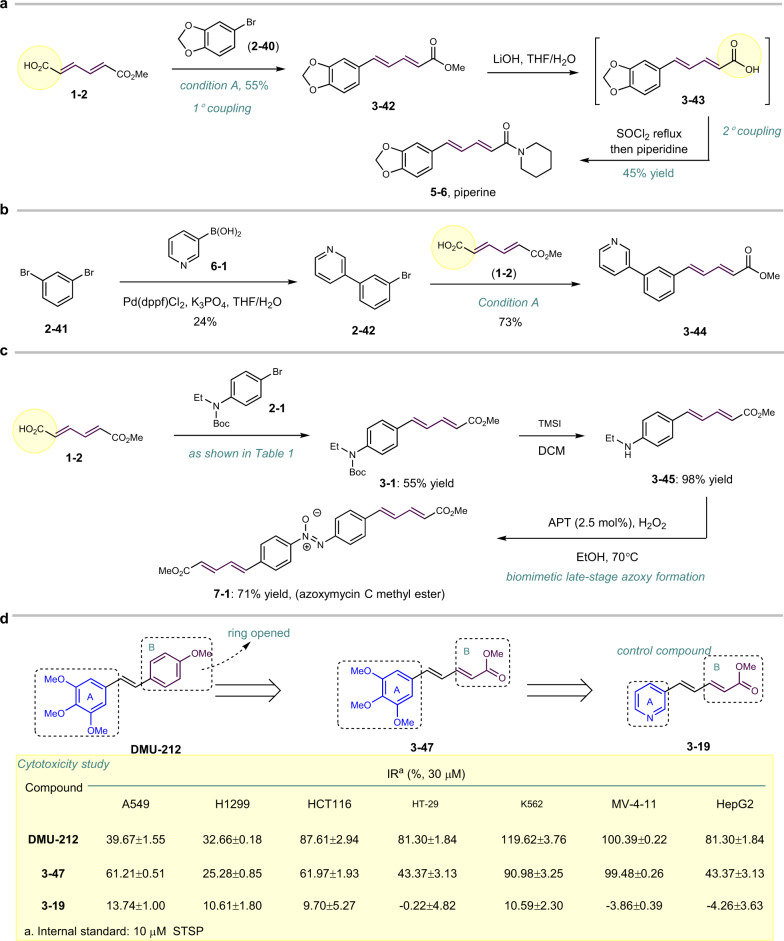
Application in natural product synthesis and medicinal chemistry. **a** Total synthesis of piperine. **b** Synthesis of piperine analog. **c** Biomimic synthesis of methylester of azoxymycin C. **d** Application in the design and synthesis of DMU-212; STSP: staurosporine.

Piperine, a natural alkaloid isolated from *Piper nigrum* L., exhibits diverse biological activities, including antioxidant, immunomodulatory, anti-asthmatic, and anti-inflammatory^34^. Utilizing our approach, the diene moiety in piperine was introduced smoothly from compound **2–40**. After hydrolysis and amide formation, piperine **5–6** was obtained in moderate yield (Fig. 5a). The analogs of piperine can be accessed similarly. For instance, compound **3–44** was conveniently obtained from 1,3-dibromobenzene **2–29** via a sequential Suzuki and decarboxylative coupling (Fig. 5b). Azoxymycin **A**, **B**, and **C** are quite unique natural products containing diarylazoxy scaffolds isolated from *Streptomyces chattanoogensis* by Li^2^. Interestingly, the proposed biosynthetic pathway^35^ is quite similar to our research in tungstate-catalyzed azoxy synthesis^9^. Therefore, a late-stage biomimetic synthesis of azoxymycin C was conducted (see Supplementary Note 1), utilizing both the decarboxylative coupling and tungstate-catalyzed azoxy formation^9^ developed in our lab. After installing the diene moiety, deprotonation, and azoxy formation from compound **1–2**, the desired product **7–1** was isolated as a yellow solid in moderate yield (Fig. 5c, see Supplementary Fig. 9).

### Application in anticancer small-molecule design and synthesis

3,4,5,4′-tetramethoxystilbene (DMU-212)^36,37^ is an analog of resveratrol, exhibiting good anticancer bioactivities in several cancer cell lines. From the perspective of SAR (structure and activity relationship) study, the B ring in DMU-212 can be replaced by a vinyl carboxylate, as shown in compound **3–47** prepared from 3,4,5-trimethoxylbenzyl bromide by this decarboxylative coupling. In all the tested cancer cell lines (human non-small lung cancer A549 and H1299 cells, colon cancer HCT116 and HT29 cells, leukemia K562 and MV-4-11 cells, and liver cancer HepG2 cells), compound **3–47** showed comparable inhibition rates as those of DMU-212, except HT29 and HepG2 cells. By contrast, a control compound **3–19** bearing a pyridine motif in the A ring and vinyl ester in the B ring, failed to show any cytotoxicity. Such evident different performances excluded the possibility that they worked only as a Michael acceptor that led to cytotoxicity. Thus, compound **3–47** can be a starting point for anticancer drug discovery, indicating the good potential of our methods in medicinal chemistry (Fig. 5d).

### Possible reaction mechanisms

**Fig. 6:**
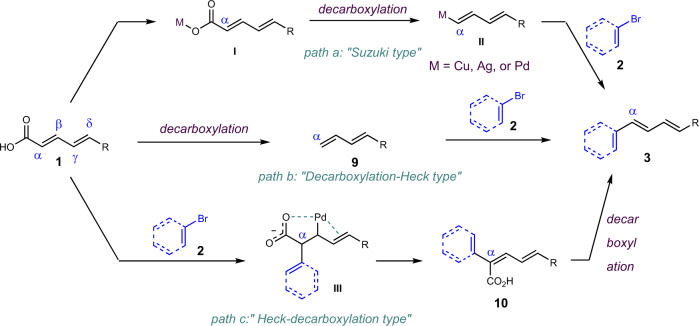
Possible reaction mechanisms. **a** “Suzuki type” reaction pathway. **b** “Decarboxylation-Heck type” reaction pathway. **c** “Heck-decarboxylation type” reaction pathway.

Three possible reaction pathways could be involved in this reaction: (1) “Suzuki type”, in which decarboxylation occurred first to form C–M (M = Cu, Ag, and Pd) bond followed by transmetallation and reductive elimination similar to Suzuki coupling (Fig. 6, path a); (2) “Decarboxylation-Heck type”, in which decarboxylation occurred first to form alkene followed by Heck coupling (Fig. 6, path b); (3) “Heck-decarboxylation type”, in which Heck coupling took place first followed by decarboxylation, and the carboxylic acid played as a directing group to control the α-selectivity as proposed in the “Introduction” (Fig. 6, path c).

### Mechanism studies

**Fig. 7:**
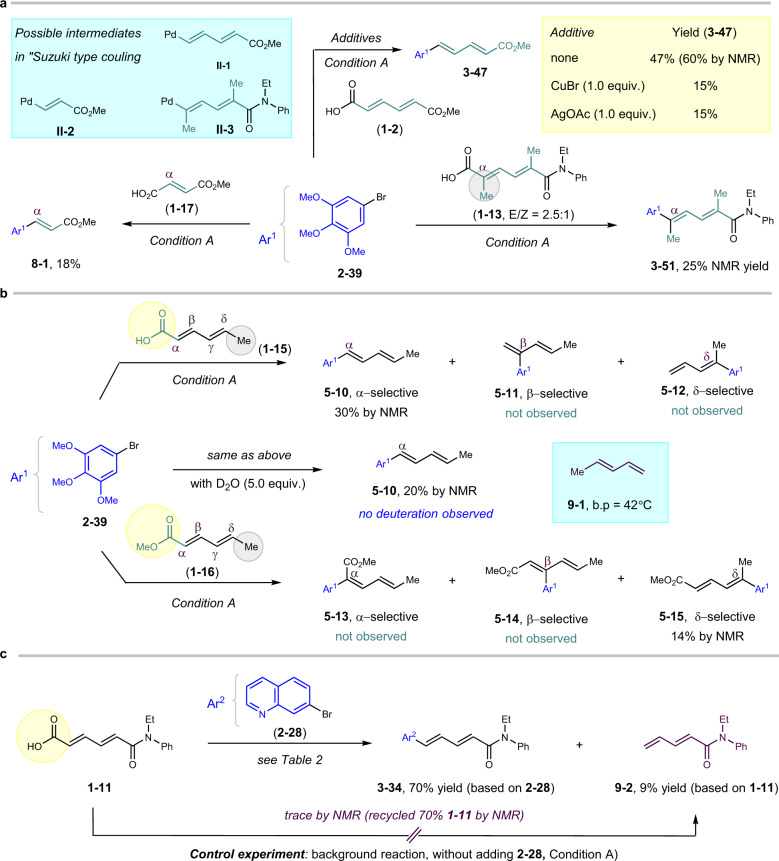
Reaction mechanism investigation 1. **a** “Suzuki type” mechanism investigation. **b** Carboxylate-directed Heck-coupling investigation. **c** Heck-decarboxylation control experiments.

To dig out more details about the reaction mechanism, several control experiments were conducted (Supplementary Note 2). Cu or Ag salts were used a lot in “Suzuki type” decarboxylative coupling^7,18,23,25,38,39^, facilitating decarboxylation to form C–Cu/Ag bonds. In our reaction, adding either CuBr or AgOAc failed to promote the reaction, but instead inhibited the reaction. In addition, both substrates **1–17**^28,40^ and **1–13**, in theory, can also afford the desired coupling products, if the reaction followed the “Suzuki type” mechanism. The possible Pd intermediates **II-2** and **II-3** should be formed similarly as **II-1**, leading to comparable yields of products. However, significant erosion of efficiency was observed with only 8% and 25% yield of products, respectively (Fig. 7a).

Since compound **1–2** embodied carboxylates on both sides, it was difficult to distinguish the electronically and sterically similar ɑ-/β-positions. Accordingly, sorbic acid (**1–15**) with significantly electronically and sterically different ɑ-/β-positions, bearing one carboxylic group on one side and a methyl group on the other side, was selected as control substrate. To our surprise, the ɑ-selective product **5–10** still dominated the reaction. Sterically and electronically favored β-/δ-selective products (**5–11**, **5–12**) from conventional Heck coupling were not observed. In contrast, under normal Heck-coupling condition, the δ-selective coupling product from sorbic acid was obtained^31^ (Supplementary Fig. 12). Adding 5.0 equivalent of D_2_O failed to afford any deuterated product of **5–10**, supporting a β-decarboxylative elimination procedure^41^ (Supplementary Fig. 16). Meanwhile, methyl sorbate (**1–16**) bearing similar steric and electronic properties as those of sorbic acid, afforded no ɑ- and β-selective products (**5–13**, **5–14**). Instead, the δ-selective product **5–15** was observed by H-NMR, in consistent with Heck’s discovery^42^ (Fig. 7b; Supplementary Figs. 12, 13). Quite interestingly, during the substrate scope investigation, a by-product **9–2** from the decarboxylation of **1–11** was isolated in 9% yield, evoking our suspension that this reaction might follow the “Decarboxylation-Heck type” mechanism. However, the background reaction without adding substrate **2–24** failed to yield any decarboxylation product **9–2**, while ~70% starting material **1–11** remained untouched^33^ (Fig. 7c; Supplementary Fig. 18). Taking into account of the volatile property of diene **9–1** (around 42 °C), no deuteration product was observed when adding D_2_O, and no intermediate **9–2** was observed from the background experiment; the “Decarboxylation-Heck type” mechanism is less favored, but might be involved in a complex fashion.

**Fig. 8:**
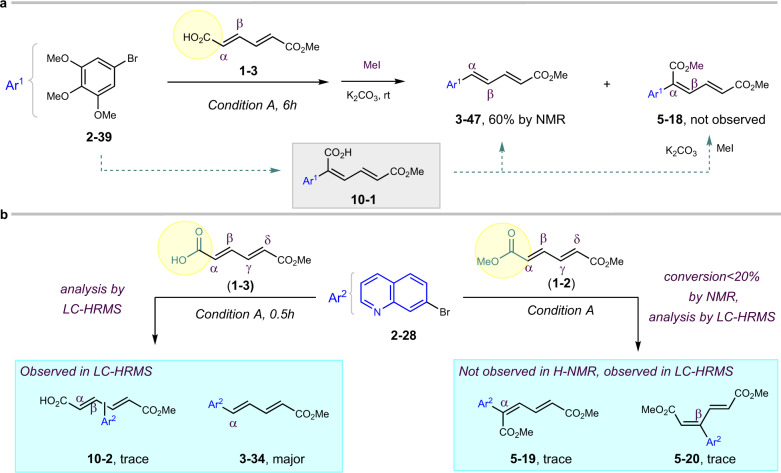
Reaction mechanism investigation 2. **a** Effect of carboxylate during the coupling experiment. **b** Experiment to trap the reaction intermediate.

Further control experiments were conducted between **1–3** and **2–39** for 6 h, followed by adding MeI to trap possible intermediate **10–1**^25^. However, no corresponding product **5–18** derived from **10–2** was detected by H-NMR of crude reaction mixture, except 60% yield of product **3–47** (Fig. 8a; Supplementary Fig. 15). In addition, shielding the carboxylic acid in **1–2** with methylester totally shut down the reaction as observed in substrate **1–3**, and only trace Heck-coupling products **5–19** and **5–20** could be observed in LC-HRMS, suggesting that the carboxylate not only controls the ɑ-selectivity but also promotes the reaction. When stopping the reaction between substrates **1–2** and **2–28** after 0.5 h, only trace amount of possible intermediate **10–2** was detected by LC-HRMS along with the major product **3–34** (Fig. 8b; Supplementary Fig. 17). Combining this phenomena and the result of the deuteration experiment in Fig. 7b, such intermediates **10–1** and **10–2** from “Heck-decarboxylation” mechanism are less possible to be involved.

### Proposed reaction mechanism

**Fig. 9:**
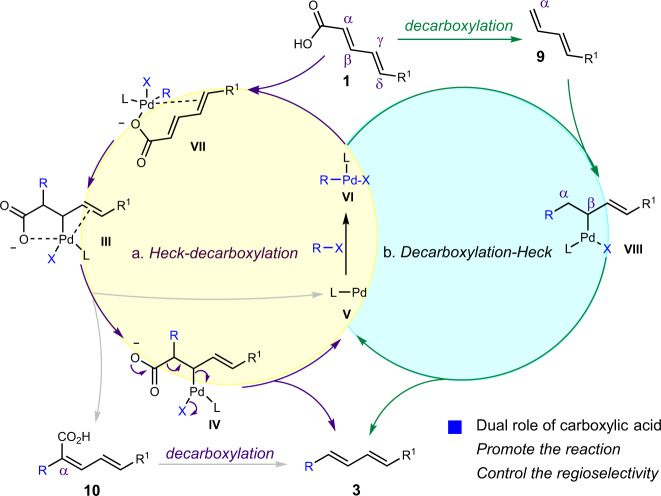
Proposed reaction mechanism. **a** Directed Heck-decarboxylation mechanism. **b** Decarboxylation-Heck mechanism.

Accordingly, based on all the mechanism investigations, evidently the carboxylic group in this reaction plays significant dual roles via directing effects: (1) promote the reaction and (2) control the ɑ-regioselectivity albeit the substrates’ innate electronic and steric properties. Besides, the diene motif also played an important role to promote the reaction. Thus, having identified the importance of both carboxylic acid and diene moieties, the reaction mechanism was proposed as the following. The oxidative insertion of aryl halides of Pd-catalyst (**V**) took place to afford catalyst intermediate (**VI**), followed by ligand coordination from dienedioic acid **2** to afford intermediate **VII**. The carboxylic acid then directed the insertion step to afford intermediate **III**, of which both carboxylic acid and another alkene moiety could help stabilize the Pd catalyst by providing the addition coordination effect. The desired diene-containing product **3** was generated after the β-decarboxylative elimination and releasing Pd-catalyst (**IV**). Meanwhile, the β–H elimination might also be involved to afford intermediate **10** followed by decarboxylation to yield product **3** (Fig. 9a). The decarboxylation-Heck reaction mechanism cannot be excluded as well, but might serve as a minor reaction pathway in a complex manner. Substrate **1** first undergoes the decarboxylation to afford diene **9**, followed by Heck coupling to afford product **3** (Fig. 9b).

In summary, a convenient approach to quickly install diene scaffold from dienedioic acids via Pd-catalyzed directed Heck-dicarboxylate coupling has been developed. This reaction exhibits broad substrate scope and good functional group tolerance. Utilizing this method, the late-stage functionalization of complex drug molecules and construction of various diene products via dual coupling can be conveniently achieved. Piperine and the methylester of azoxymycin C were synthesized in three steps. Moreover, our method provides an easy access to an analog of DMU-212, a small molecule bearing anticancer activities, and one product showed comparable activities. The mechanism studies showed that carboxylic acid group played as a directing group by two means: (1) promoting the reaction; (2) controlling the regioselectivity. Further work to utilize the method to application in quick access of biologically important polyenes is under progress in our lab.

## Methods

### Synthetic procedures

See Supplementary Methods and Supplementary Figs. 1–5.

### Synthetic procedure for piperine and methylester of azoxymycin C

See Supplementary Figs. 6–9.

### In vitro bioanalysis

See Supplementary Methods.

### Characterization of starting materials, intermediates, and products

See Supplementary Methods, Supplementary Figs. 20-31, 35-152.

## Supplementary information

Supplementary Information

Description of Additional Supplementary Files

Supplementary Data 1

## Data Availability

Procedures for experiments and characterization data for products are available in the Supplementary Information. The data for the X-ray crystallographic structure of **3–2** are available free of charge from the Cambridge Crystallographic Data Center under accession number CCDC: 1972800. Any other data are available from the corresponding author upon reasonable request.
