# Supplementary material for: Dienedioic acid as a useful diene building block via directed Heck-decarboxylate coupling
Source: Commun Chem. 2020 Apr 20;3:48. doi: 10.1038/s42004-020-0295-0 (PMC9814911; doi:10.1038/s42004-020-0295-0)
Supplement: Supplementary file 1 — Supplementary Information [file 42004_2020_295_MOESM1_ESM.pdf]

Dienedioic Acid as a Useful Diene Building Block *via* Directed Heck-Decarboxylate Coupling

Lei, Ke[1], Zhilong Chen[1]*

[1] School of Pharmacy, Huazhong University of Science and Technology(HUST), 13 Hangkong Road,

Wuhan, Hubei 430030, P. R. China, pmchenzl@hust.edu.cn

Supplementary Information

Supplementary Methods

I. General Information

Glassware and stir bars were dried in an oven at 70 °C for at least 12h and then cooled in a desiccator cabinet over Drierite prior to use. Optimization and substrate screen were performed in 20 mL vials. All other reactions were performed in round-bottom flasks sealed with rubber septa. Plastic syringes or pipets were used to transfer liquid reagents. Reactions were stirred magnetically using Teflon-coated, magnetic stir bars. Analytical thin-layer chromatography (TLC) was performed using glass plates pre-coated with 0.25 mm of 230–400 mesh silica gel impregnated with a fluorescent indicator (254 nm and 320 nm). TLC plates were visualized by exposure to ultraviolet light and/or exposure to $KMnO_4$ stain as well as phosphomolydic acid (PMA) and cerium molybdate stain. Organic solutions were concentrated under reduced pressure using a rotary evaporator. Flash-column chromatography was performed on silica gel (60 Å, standard grade).

Materials and Instrumentation. Nuclear magnetic resonance spectra were recorded at ambient temperature (unless otherwise stated) on Bruke 400 MHz spectrometers. All values for proton chemical shifts are reported in parts per million (δ) and are referenced to the residual protium in NMR solvents ($CDCl_3$ = δ 7.26, CD_3OD= δ 3.31, DMSO-D_6 = δ 2.50 ppm). All values for carbon chemical shifts are reported in parts per million (δ) and are referenced to the carbon resonances in $CDCl_3$ (δ 77.0), CD_3OD (49.00) and DMSO-D_6 (39.52). NMR data are represented as follows: chemical shift, multiplicity (s = singlet, d = doublet, t = triplet, q = quartet, quin = quintet, m = multiplet, br = broad), coupling constant (Hz), and integration. Infrared spectroscopic data is reported in wavenumbers (cm^{-1}). High-resolution mass spectra was obtained using a liquid chromatography-electrospray ionization and Time-of-flight mass spectrometer.

II. Condition optimization

Supplementary Table 1. Ligands screening for condition optimization 1

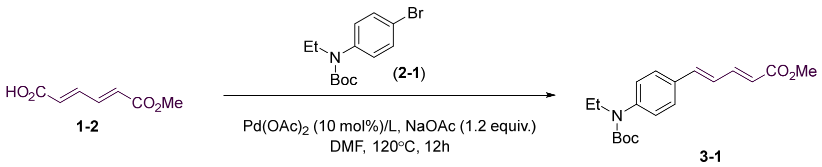

Entry[a]	**1-2: 2-1**	Ligand (mol%)	Yield[b]
1[c]	1.1:1	**L1** (40)	14%[d]
2[c]	1: 1	**L2** (40)	16%
3[c]	1: 1	**L3** (40)	trace
4	1: 1	**L4** (40)	trace
5	1.5:1	**L5** (40)	14%[e]
6	1.5:1	**L6** (20)	17%
7	1.5:1	**dppf** (12)	54% (65%[f])
8[g]	1.5:1	**Pd(dCypf)Cl$_2$** (10)	38%
9	1.5:1	**L8 (dppe)** (12)	55%
10	1.5:1	**L8 (dppp)** (12)	trace
11	1.5:1	**L8 (dppb)** (12)	28%
12	1.5:1	**Xantphos** (40)	trace
13	1.5:1	**(*R*)-BINAP** (40)	36%
14[h]	1.5:1	**MonoPhos** (40)	coversion<50%

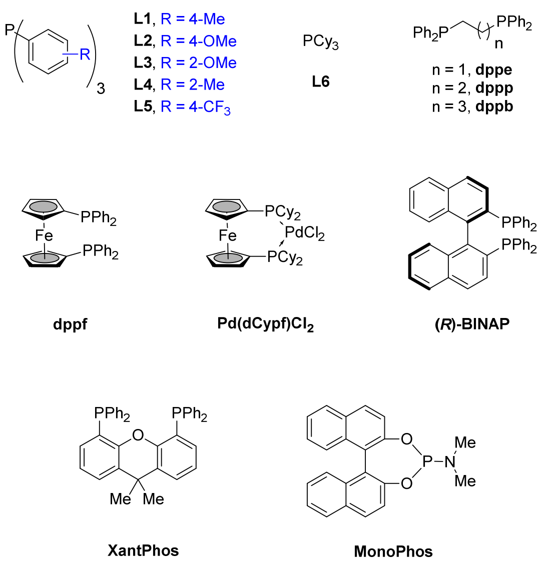

a. all reactions for condition optimization were conducted with compound **1-2** (59 mg, 0.375 mmol, 1.5 equivalent) and **2-1** (75 mg, 0.25 mmol, 1.0 equivalent) with $Pd(OAc)_2$ (5.6 mg, 10 mol%), NaOAc (25 mg, 0.3 mmol, 1.2 equivalent) in 2.0 mL DMF under nitrogen balloon; b, isolated yield; c. 2.0 mL DMF was utilized; d. 0.5 mmol scale; e. CsOAc (58 mg, 0.3 mmol, 1.2 equivalent) was used instead of NaOAc; f: NMR yield determined with CH_2Br_2 as internal standard of crude reaction mixture; g. Pd(dCypf)Cl_2 was used instead of $Pd(OAc)_2$ without adding ligands; h. 1.5 equivalents of NaOAc was used.

Supplementary Table 2. Ligands screening for condition optimization 2

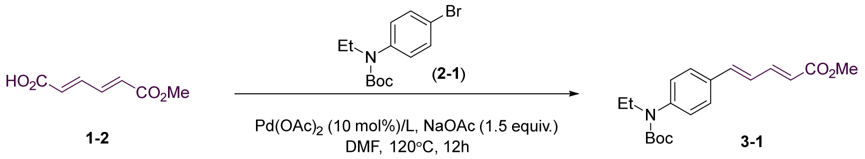

Entry[a]	**1-2**:**2-1**	Ligand (mol%)	Yield[b]
1[c]	1:1	**L9** (40)	17%
2	1.5:1	**XPhos** (40)	20%
3	1.5:1	**BrettPhos** (40)	trace
4	1.5:1	**DavePhos** (20)	41%
5	1.5:1	**XPhos-G2** (10)	44%
6	1.5:1	**XPhos-G3** (10)	44%
7[d]	1.2:1	**SPhos-G2** (20)	12%
8	1.5:1	**RuPhos-G3** (10)	40%

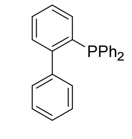
L9

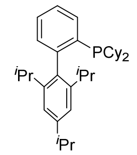
XPhos

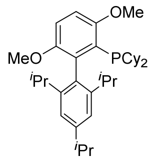
BrettPhos

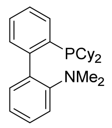
DavePhos

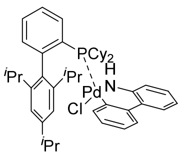
XPhos-G2

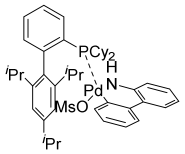
XPhos-G3

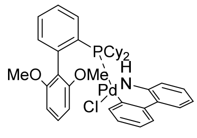
SPhos-G2

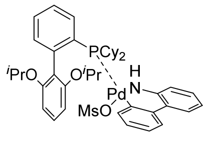
RuPhos-G3

a. all reactions for condition optimization were conducted with compound **1-2** (59 mg, 0.375 mmol, 1.5 equivalent) and **2-1** (75 mg, 0.25 mmol, 1.0 equivalent) with $Pd(OAc)_2$ (5.6 mg, 10 mol%), ligand, NaOAc (31 mg, 0.375 mmol, 1.5 equivalent) in 2.0 mL DMF under nitrogen balloon at 120℃; b. isolated yield; c. with 1.0 equivalent of compound **1-2** and NaOAc; d. with 1.2 equivalent of compound **1-2** and NaOAc

Supplementary Table 3. Condition optimization 3

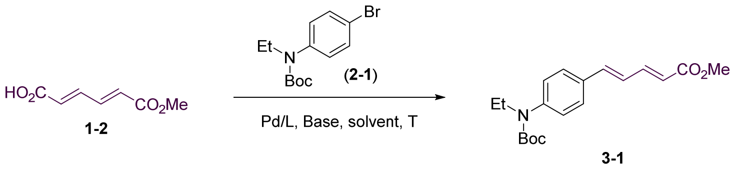

Entry[a]	1-2:2-1	Pd(mol%) / Ligand (mol%)	Base (equiv.)	Solvent	T/C	Yield[b]
Loading of catalyst screening						
1	1.5:1	XPhos-G2 (10)	NaOAc (1.5)	DMF	120	44%
2	1.5:1	XPhos-G2 (15)	NaOAc (1.5)	DMF	120	43%
3	1.2:1	XPhos-G2 (20)	NaOAc (1.2)	DMF	120	17%
Ratio of starting materials screening						
4	2:1	XPhos-G2 (10)	NaOAc (2.0)	DMF	120	44%
5	1:1	XPhos-G2 (10)	NaOAc (1.2)	DMF	120	37%
6	1:2	XPhos-G2 (10)	NaOAc (2.0)	DMF	120	41%
7	1:2	XPhos-G2 (5)	NaOAc (1.2)	DMF	120	27%
Base screening						
8	1.5:1	XPhos-G2 (10)	LiOAc (1.5)	DMF	120	33%
9	1.5:1	XPhos-G2 (10)	KOAc (1.5)	DMF	120	35%
10	1.5:1	XPhos-G2 (10)	CsOAc (1.5)	DMF	120	55%
11	1.5:1	XPhos-G2 (10)	Li_2CO_3 (1.5)	DMF	120	trace
12	1.5:1	XPhos-G2 (10)	Na_2CO_3 (1.5)	DMF	120	30%
13	1.5:1	XPhos-G2 (10)	K_2CO_3 (1.5)	DMF	120	37%
14	1.5:1	XPhos-G2 (10)	Cs_2CO_3 (1.5)	DMF	120	42%
15[c]	1.5:1	$Pd(OAc)_2$ (10)/dppf (12)	CsOAc (1.5)	DMF	120	50%
Reaction temperture screening						
16[c]	1.5:1	$Pd(OAc)_2$ (10)/dppf (12)	NaOAc (1.5)	DMF	120	54%
17[c]	1.5:1	$Pd(OAc)_2$ (10)/dppf (12)	NaOAc (1.5)	DMF	110	conversion<50%
19[c]	1.5:1	$Pd(OAc)_2$ (10)/dppf (12)	NaOAc (1.5)	DMF	130	24%
20[c]	1.5:1	$Pd(OAc)_2$ (10)/dppf (12)	NaOAc (1.5)	DMF	140	trace
Additive effect						
21[d]	1.5:1	$Pd(OAc)_2$ (10)/dppf (12)	NaOAc (1.5)+AgOAc (1.0)	DMF	120	trace
22[d]	1.5:1	$Pd(OAc)_2$ (10)/dppf (12)	NaOAc (1.5)+CuBr(1.0)	DMF	120	trace

a. all reactions for condition optimization were conducted with compound **1-2** (59 mg, 0.375 mmol, 1.5 equivalent) and **2-1** (75 mg, 0.25 mmol, 1.0 equivalent) with $Pd(OAc)_2$ (5.6 mg, 10 mol%), ligand, NaOAc (31 mg, 0.375 mmol, 1.5 equivalent) in 2.0 mL DMF under nitrogen balloon at 120℃; b. isolated yield; c. $Pd(OAc)_2$ (5.6 mg, 10 mol%) and dppf (16.7 mg, 12 mol%) was used instead of XPhos-G2; d. with additives, like AgOAc and CuBr, as promoters for decarboxylation

III. Procedure for starting materials and products synthesis, and derivation

III-1. Starting material synthesis

Compound **1-2** was prepared to according to following procedure.

Step 1: Compound **1-3** was prepared according to reported literature from methyl propiolate (**12)** and 1,4-Diazabicyclo[2.2.2]octane (DABCO) after two steps as a mixture (E/Z = 3:1)[1].

Step 2: To the solution of compound **1-3** (2.3 g, 13.5 mmol, 1.0 equivalent) in DCM/MeOH (50 mL/40 mL) was added NaOH (aq., 1M, 13.5 mL, 1.0 equivalent). And then the reaction mixture was stirred at 35℃ for 48h followed by adding 14 mL HCl (1M). The resulting mixture was extracted by EtOAc, washed by H_2O and brine, dried by Na_2O_4. The desired product **1-2** (1.06 g, 50% yield) was obtained as white solid after filtration, concentration and flash chromatography (Supplementary Fig. 1).

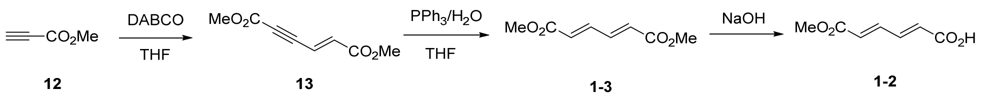

Supplementary Fig. 1 Preparation of compound 1-2

Compound **1-5**, **1-6** and **1-7** were prepared to according to following procedure.

To a 25 mL flask with compound **1-1** (3.0 mmol, 1.0 equivalent) was added 5.0 mL sulfurous dichloride. Next, the reaction was heated to 80℃ for 6-9 h until all compound **1-1** consumed. The desired compound **1-4** was obtained after removing the solvents in rotate evaporation. Next, 10 mL anhydrous DCM was added into the same flask, followed by adding compound **14** (3.0 mmol, 1.0 equivalent, in 3.0 mL anhydrous DCM) and 1.5 mL NEt_3. The reaction mixture was stirring for overnight at room temperature. Upon completion, HCl (aq., 1M) was added to quench the reaction, and the resulting mixture was extracted by EtOAc, washed by H_2O and brine, dried by Na_2SO_4. The desired products **1-5**, **1-6** and **1-7** were obtained after filtration, concentration and flash chromatography (Supplementary Fig. 2).

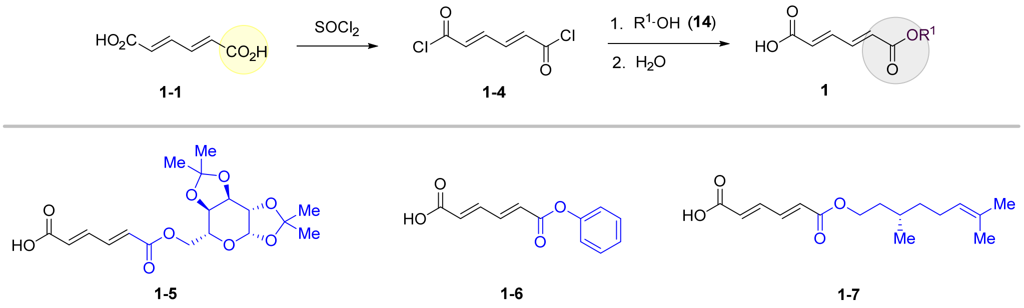

Supplementary Fig. 2 Preparation of compound **1-5**, **1-6**, **1-7**

Compound**1-11** and **1-12** were prepared to according to following procedure.

Compound **1-2** (1.5 mmol, 1.0 equivalent) and 1.5 mL sulfurous dichloride was added into a 25 mL flask, and the mixture was then heated to 80℃ for 6-9 h. Remove all the solvents, and crude compound **1-8** was dissolved in 10 mL anhydrous DCM, followed by adding compound **15** (1.5 mmol in 3.0 mL anhydrous DCM) and 0.5 mL NEt_3. The reaction mixture was stirred at room temperature for overnight and then quenched by adding HCl (aq., 1M), extracted by EtOAc, washed by H_2O and brine, dried by Na_2SO_4. The desired product **1-9** or **1-10** were obtained after filtration, concentration and silica gel column purification,

which was directly utilized in following hydrolysis step.

To the solution of compound **1-9** or **1-10** in THF/H_2O was added LiOH. Upon completion, the reaction was then neutralized by adding HCl (aq., 1M), extracted by EtOAc, washed by H_2O and brine, dried by Na_2SO_4. The product **1-11** or **1-12** was gained as white solid after filtration, concentration and flash chromatography (Supplementary Fig. 3).

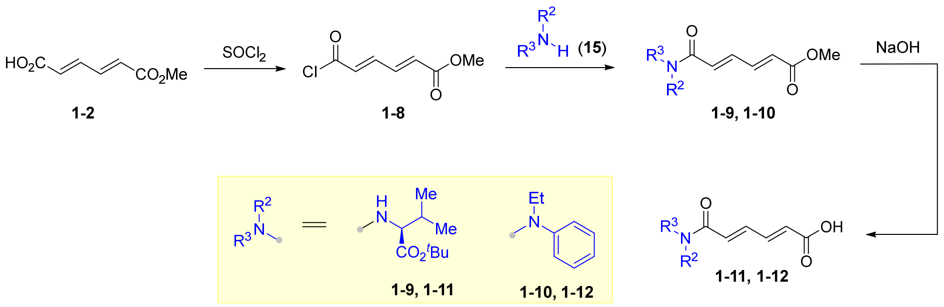

Supplementary Fig. 3 Preparation of compound **1-10** and **1-11**

Compound **2-1** was prepared according to following procedure.

To the mixture of 4-bromoaniline (**2-44**, 2.58 g, 15 mmol, 1.0 equivalent) in DCM (25 mL) was added Boc_2O (5.02 g, 25 mmol, 1.67 equivalent). Next the reaction mixture continued stirring for overnight. Compound **2-45** (4.01 g, 98% yield) was obtained as crude product after removing the solvents, and directly used in the next step.

To the mixture of NaH (1.0 g, 25 mmol, 2.5 equivalent) and TBAI (tetrabutylammonium iodide, 185 mg, 0.5 mmol) in a 50 mL flask was added anhydrous THF (25 mL) at 0℃ under nitrogen balloon. After no H_2 generated, compound **2-45** (2.71 g, 10 mmol, 1.0 equivalent, in 5 mL THF) was added 0℃. The reaction mixture continued stirring for another 15 min, followed by adding bromoethane (2.18 g, 20 mmol, 2.0 equivalent) at 0℃. And then the reaction was warmed to room temperature for overnight, and quenched by adding H_2O (around 0.5 mL) in the open air. Following, the reaction mixture was extracted by EtOAc, washed by H_2O and brine, dried by Na_2SO_4. The desired product **2-1** (2.67 g, 89% yield) was obtained as colorless solid after filtration, concentration and purified by flash chromatography (Supplementary Fig. 4).

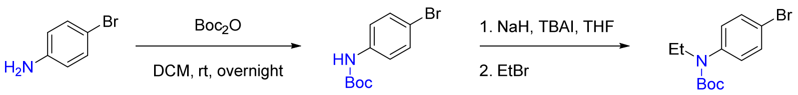

Supplementary Fig. 4 Preparation of compound **2-1**

III-2 Products **3-1** to **3-47** were prepared according to **General procedure A** or **B** as shown in following conditions in Supplementary Fig. 5.

Condition A To a 25 mL Schlenk tube with compound **1** (0.375 mmol, 1.5 equivalent), compound **2** (0.25 mmol, 1.0 equivalent), XPhos-G2 (0.025 mmol, 10 mol%) and CsOAc (or Na_2CO_3, 0.375 mmol, 1.5 equivalent) was added anhydrous DMF (2.0 mL) under nitrogen balloon. Next, the reaction mixture was stirred at 120℃ in oil bath for 12h, and then cooled to room temperature followed by quenched by adding H_2O (around 0.2 mL). The resulting mixture was diluted with EtOAc, washed by H_2O and brine, dried by Na_2SO_4. The desired product **3** or **5** was obtained *via* flash chromatography after filtration and

concentration.

Condition B was the same as **Condition A** by utilizing $Pd(OAc)_2$ (10 mol%), **dppf** (12 mol%) and NaOAc (1.5 equivalent).

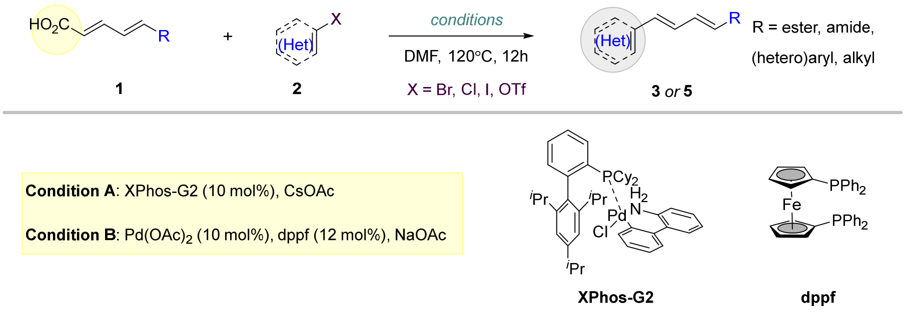

Supplementary Fig. 5 Preparation of products **3** and **5**

IV. Total synthesis of piperine

The piperine was prepared according to following procedure as shown in Supplementary Fig. 6.

Compound **3-43** (32 mg, 55% yield，0.25 mmol scale) was prepared according to **General Procedure A** (Condition A) from substrate **1-2** and **2-28** as shown in Supplementary Fig. 5.

Compound **3-44** (87 mg, 95% yield; 0.42 mmol scale) was prepared the same as compound **3-38** as shown in Figure S20. The piperine (**5-6**, 40.5 mg, 47% yield in 0.4 mmol scale) was obtained the same as compound **1-11** as shown in Supplementary Fig. 6.

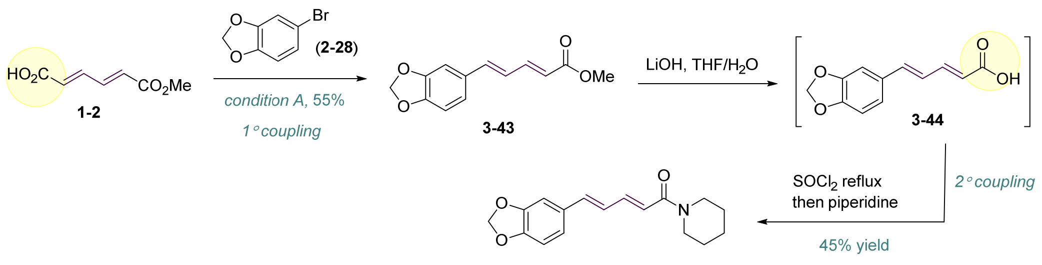

Supplementary Fig. 6 Total synthesis of piperine

Supplementary Note 1

V. Efforts toward to total synthesis of Azoxymycin C

Based on the structural feature of azoxymycin C, two key steps were involved: diene introduction and azoxy formation. The diene motif could be furnished smoothly under this decarboxylative coupling; and the azoxy could be conveniently accomplished by our previous developed tungstate-catalyzed azoxy formation. Therefore, two synthetic plans were designed based on the sequence of these two key steps.

In plan A, the azoxy formation take place first followed by a dual decarboxylative coupling from dieneoic acid **1-2** and (*Z*)-1,2-bis(4-bromophenyl)diazene 1-oxide **2-43**, which can be conveniently prepared according to our previous research on azoxy formation[2]. In plan B, the diene motif will installed first followed by a late-stage azoxy formation from compound **3-45** (Supplementary Fig. 7).

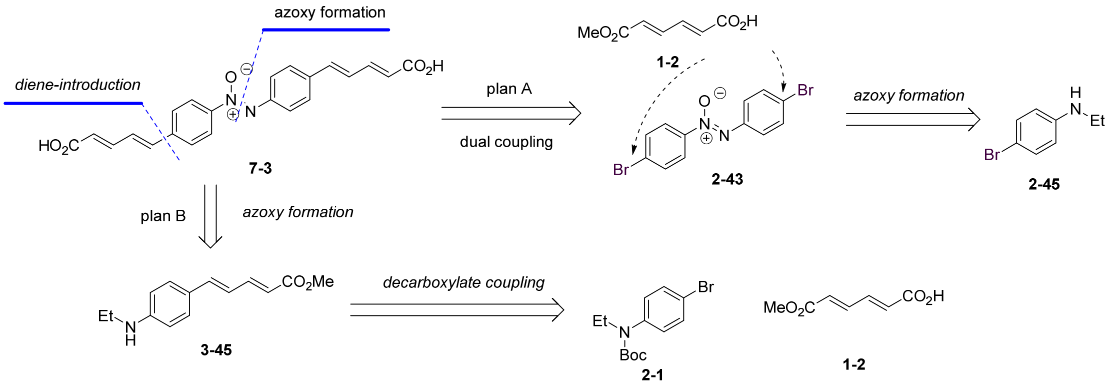

Supplementary Fig. 7 Synthetic retroanalysis of azoxymycin C

Initially, plan A was chosen, however, compound **2-43** failed to afford the desired product **7-1** *via* a dual decarboxylate coupling, probably due the instability of azoxy under current coupling conditions (Supplementary Fig. 8).

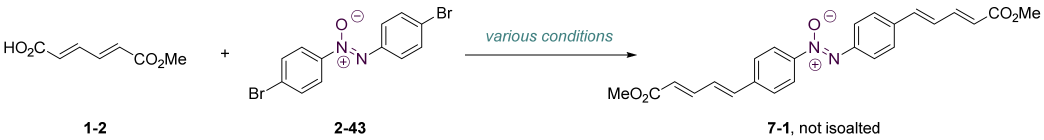

Conditions	Results
Pd(OAc)$_2$ (20 mol%)/P(p-MePh)$_3$ (40 mol%), NaOAc (4.0), DMF, 120°C	No desired product, messy
Pd(OAc)$_2$ (20 mol%)/SPhos-G2 (20 mol%), NaOAc (3.0), DMF, 120°C	No desired product, messy
Pd(OAc)$_2$ (20 mol%)/XPhos-G2 (20 mol%), NaOAc (3.0), DMF, 120°C	No desired product, messy

Supplementary Fig. 8 Efforts towards to synthesis of Azoxymycin C *via* late-stage coupling

Later, we turned attention to synthetic plan B. Currently we have finished the total synthesis of the methyl ester of azoxymycin C, and further efforts toward to total synthesis of azoxymycin A, B and C is still working on our lab. The details to accomplish the synthesis of the methyl ester of azoxymycin C is documented as following (Supplementary Fig. 9).

Step 1: Compound **3-1** was prepared according to **General Procedure A**.

Step 2: To the mixture of compound of **3-1** (85 mg, 0.26 mmol.) in anhydrous DCM was added TMSI (36uL 52mg, 0.26 mmol, 1 equiv.). The reaction mixture was stirred at room temperature for 5-10min. After extraction by EtOAc (30 mL) and being washed by H_2O (10 mL), MeOH(1mL), $NaHCO_3$ (aq., 1 mL) and brine (15 mL), the organic layer was dried by Na_2SO_4. The desired product **3-48** (58 mg, 97% yield) was obtained as yellow solid after filtration, concentration and column purification.

Step 3: To the mixture of compound **3-48** (58 mg, 0.25 mmol) in EtOH was added ammonium paratungstat (APT, 19.1 mg, 2.5 mol%) and H_2O_2 (30% aq., 0.125 mL, 1.25 mol, 5.0 equiv.). Next, the reaction mixture was heated to 70℃ for 36 h, and significant amount of yellow solid decapitated during the reaction. Upon completion, the desired product **7-1** (37 mg, 71% yield, 0.089 mmol scale) was isolated by centrifugation as a yellow solid.

Compound **3-49** was prepared under the same procedure as documented in step 1, and isolated as a yellow solid (37 mg, 71% yield), however, the desired product azoxy product **7-2** failed to isolated under the same procedure probably due to the instability of compound **3-50**.

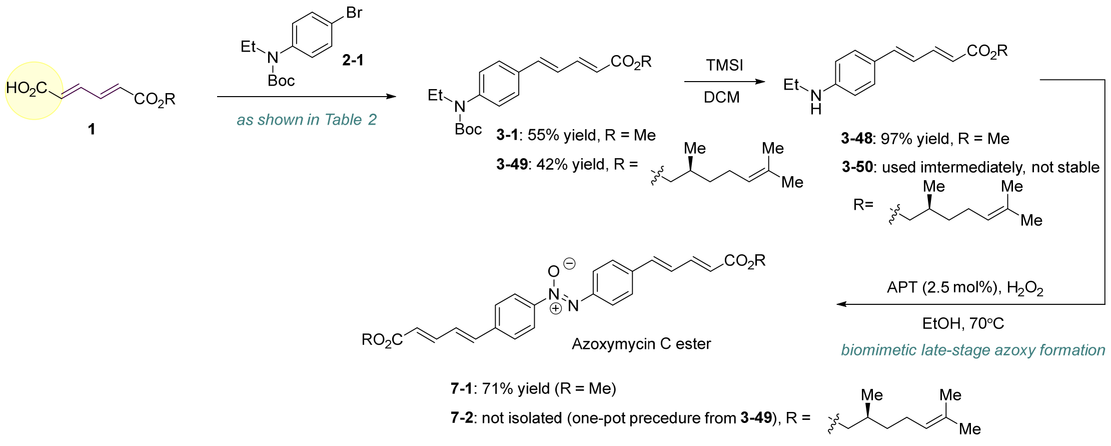

Supplementary Fig. 9 Efforts towards to synthesis of Azoxymycin C

Supplementary Note 2

VI. Mechanism study

Three possible reaction mechanism might be involved in our reaction as illustrated in the manuscript, thus several experiments were designed to identify the reaction mechanism (see Fig.4).

VI-1: Experiments to probe the Suzuki-type reaction mechanism

Decarboxylation to form a C-M (M = Cu, Ag, Pd) bonds is well-documented in literature, and this type of transformation can be facilitated by adding Cu- of Ag-salts[3-5]. However, in our reaction, either adding CuBr (1.0 equivalent) or AgOAc (1.0 equivalent), failed to promote the reaction. Instead, significant erosion of yield was observed, both affording product **3-47** in 15% yield (10.5 and 10.7 mg, respectively) (Supplementary Fig. 10). The same phenomena was also observed in our previous condition screening with substrate **2-1** with **dppf** as ligands (Supplementary Table 3). These results, together, exclude that effects of CuBr and AgOAc as promoter in decarboxylation and transmetallation process in this reaction.

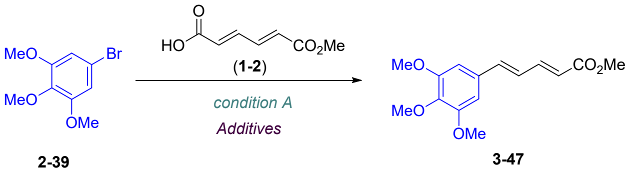

Additive	Yield (3-47)
none	47%
CuBr (1.0 equiv.)	15%
AgOAc (1.0 equiv.)	15%

Supplementary Fig. 10 Additive effects of the reaction

Another two control experiments between substrates **1-17**/**1-13** and**2-39** were conducted according to **General procedure A** (Condition A). If the reaction followed the "Suzuki type" mechanism, there should exist Pd-intermediate **II-1**. Thus, it is reasonable to hypothesize that the other two possible Pd-intermediates **II-2** and **II-3** from **1-17** and **1-13** should exist as well, of which the stability and reactivity should be similar as those of **II-1** (see Fig. 6). Accordingly, the desired coupling product **8-1** and **3-51** should be obtained in similar yields as that of **3-47**. However, product **8-1** was obtained in only 8% yield (11.5 mg), and around 25% yield of product **3-51** was detected by H-NMR analysis of crude reaction mixture. Besides, no protonation product **9-3** from **II-3** was observed (Supplementary Fig. 11, see attached H-NMR analysis)

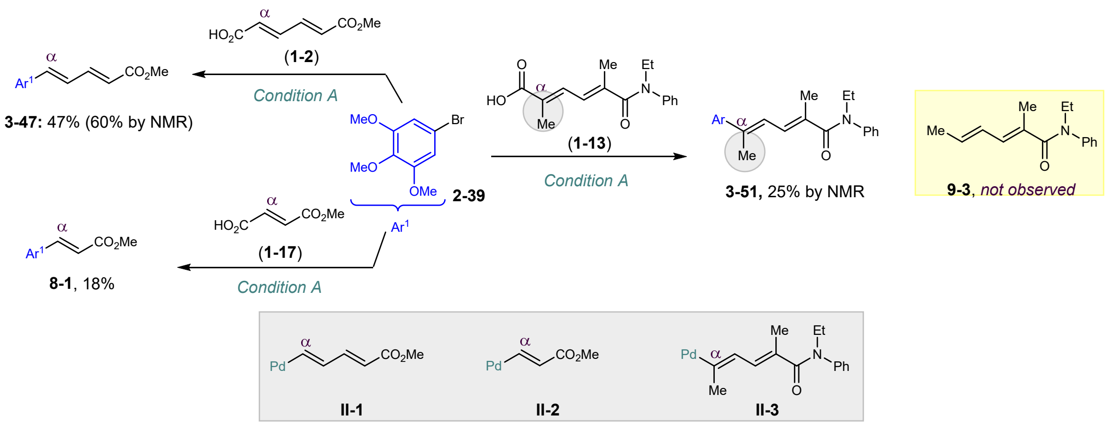

Supplementary Fig. 11 Control experiments to identify the importance of structure of dieneonic acids

VI-2: Experiments to probe the "*Heck type*" reaction mechanism

If reaction might go through a “Heck type” mechanism, the regioselectivity of products should be determined during the insertion step. It was hard to distinguish its α- and δ-position of compound **1-3** given their similar innate electronic and steric properties. Therefore, sorbic acid (**1-20**) and methyl sorbate (**1-21**) were probed as control substrates. Both of their δ-positions are favored for Heck-coupling form either inherent electronic or steric properties (Supplementary Fig.12a). Actually, under conventional Heck-coupling conditions, methyl sorbate (**1-21**) afforded γ- and β-selective products reported by Heck[6] himself early in 1982 (Supplementary Fig.12b); and sorbic acid (**1-20**) afforded the δ-selective product **5-9** reported by Mane[7] (Supplementary Fig.12c).

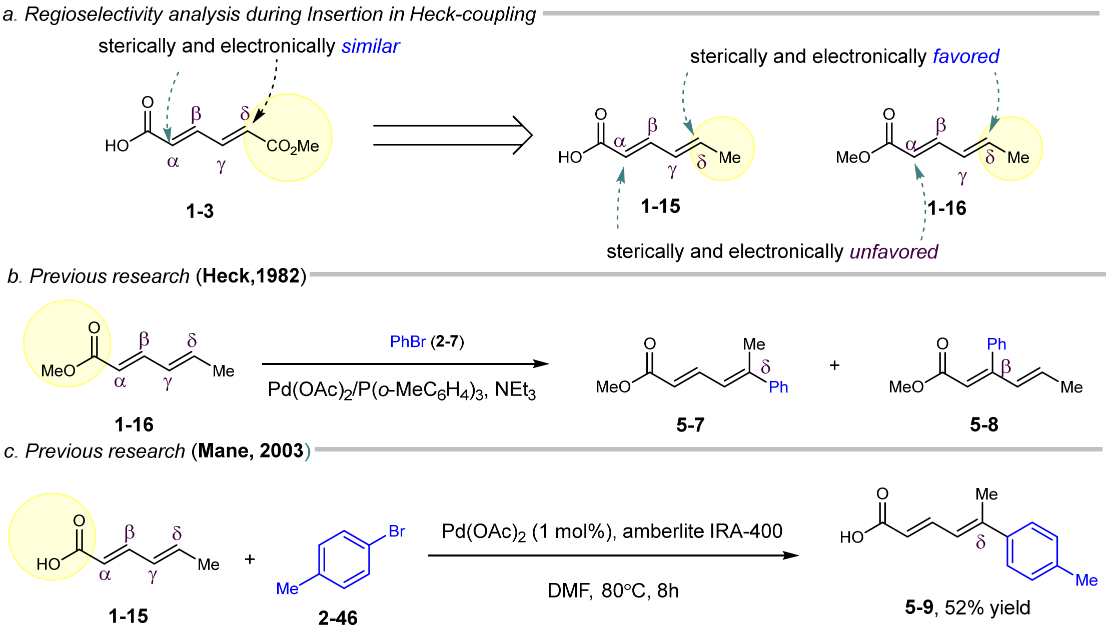

Supplementary Fig. 12 Analysis of the insertion step during the reaction

Remarkably, under our reaction condition, the sorbic acid afford the desired α-selective product (**5-10**), without β- or δ-selective products (**5-11**, **5-12**) detected. In contrast, methyl sorbate (**1-21**) failed to afford any α-selective product **5-13**; but instead, 14% δ-selective products **5-15** was observed under the same reaction condition. Of note, the (*E*)-penta-1,3-diene (**9-1**) is extremely volatile substrate (boiling point: around 42℃), and it was hard to survive under the reaction condition (120℃, 12h). These observations, clearly, indicate the importance of the carboxylic acid group in this reaction, and it is less possible the reaction went through a “Decarboxylation-Heck type” mechanism by forming the diene intermediate like **9-1** (Supplementary Fig.13).

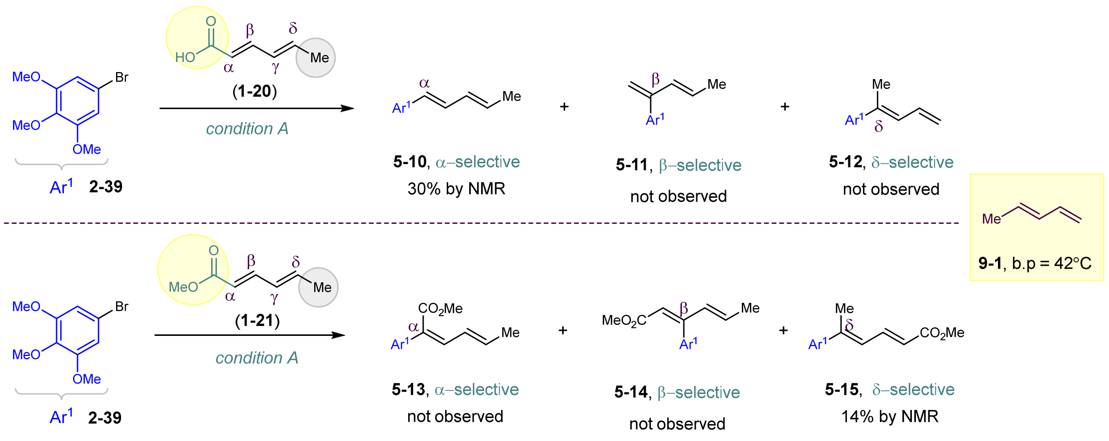

Supplementary Fig. 13 Control experiment of sorbic acid and methyl sorbate

Similar results were also observed in Miura's research[8]. In his hand, sorbic acid (**1-20**) and phenyl iodide also afford α-selective product **5-16** in moderate yield as an E/Z mixture at δ-position. Apparently, if this reaction followed the "Suzuki type" mechanism, no E/Z isomerization at δ-position should be observed. On the other hand, this phenomena can be well explained by "Heck type" mechanism. Either the intermediate **III-1** in "Heck-Decarboxylation" (path a) or **VII-1** in "Decarboxylation-Heck" (path b) can isomerize into **III -3** and **VII-3** respectively, which further undergo reductive elimination to afford desired product **5-17**. This results also consisted with Heck's discovery of coupling of diene reported in 1984[9]. However, considering the volatile property of compound **9-1** (b.p = 42℃) and the reaction condition (140℃), the "Decarboxylation-Heck" mechanism is more rational (Supplementary Fig.14).

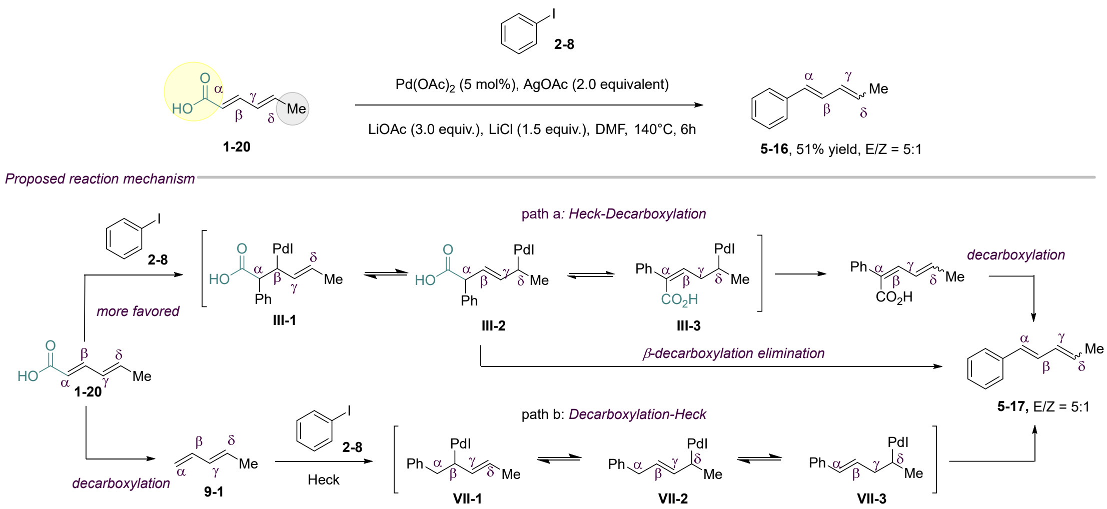

Supplementary Fig. 14 Miura's discovery and our proposed reaction mechanism

Further efforts were conducted to trap possible reaction intermediate **10-1** in the "Decarboxylation-Heck" mechanism. The reaction between **1-3** and **2-31** were stopped at 6h, and then cooled to room temperature followed by adding K_2CO_3, MeI and stirring for overnight. If there existed detectable intermediate **10-1**, then corresponding product **5-19** should be observed. However, the H-NMR of crude reaction mixture shown 60% of product **3-47** without any product **5-18** (Supplementary Fig.15).

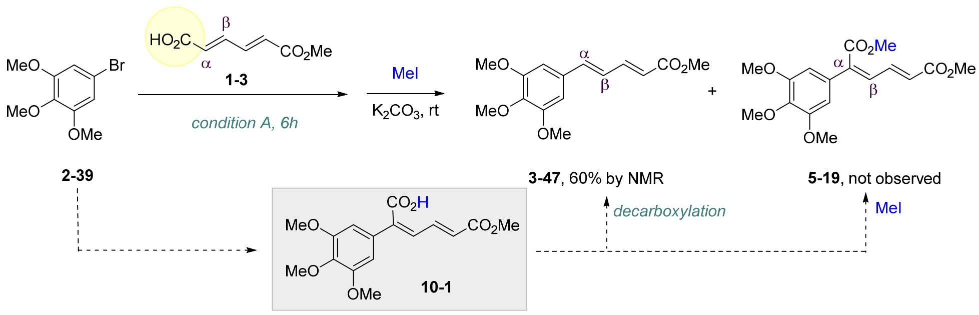

Supplementary Fig. 15 Experiment to trap reaction intermediate

Meanwhile, one additional control experiment was conducted between substrate **2-31** and **1-20** in the presence of 5.0 equivalent of D_2O, to identify whether there was any deuteration in the product. To our surprise, no deuteration at any position of product **5-10** was observed by careful comparation of the crude H-NMR spectrums (see following spectrum), but the yield was a little bit lower. If the reaction followed the "Decarboxylation-Heck" mechanism, then there should be deuterated intermediate **9-1**, given the fact that the exchange of H-D exchange between substrate **1-20** and D_2O was fast (Supplementary Fig.16, path b).

On the other side, this result could be explained by "Heck-Decarboxylation" mechanism in an unusual decarboxylation-elimination process. After the insertion step, the reaction could undergo a fast β-hydride elimination to afford intermediate **10-2D**. The decarboxylation-protonation of this compound, in theory, should yield the α-deuteration product **5-10** in the presence of D_2O (Supplementary Fig.16, path c-1). Thus, it is more reasonable that the reaction went through a decarboxylation-elimination directly from intermediate **10-2-Ia** or **10-2-Ib** to afford product **5-10** (Supplementary Fig.16, path c-2).

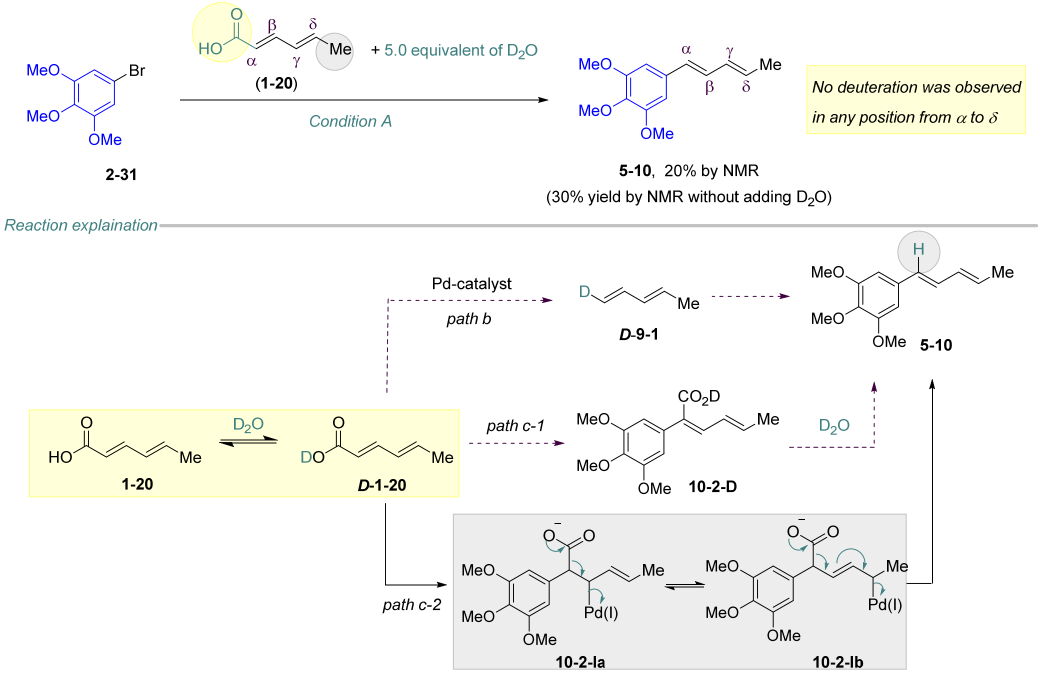

Supplementary Fig. 16 Control experiment by adding D_2O

Additional control experiments were conducted with substrate **1-3** and **1-2**. Since in previous study, the reaction might finish in less than 6h, thus the reaction with substrate **1-3** and **2-28** were conducted according to **General procedure A** (Condition A) and quenched after 0.5 h. The LC-HRMS spectrum confirmed the major products was **3-24**, along with only trace amount of **10-2** detected. Surprisingly, when blocking the acid group as shown in compound **1-2**, the conversion was quite low, and most of both starting materials **1-2** and **2-24** remained untouched by H-NMR of crude reaction mixture. And only trace amount of the Heck-coupling products **5-19** and **5-20** could be detected in LC-HRMS (Supplementary Fig.17).

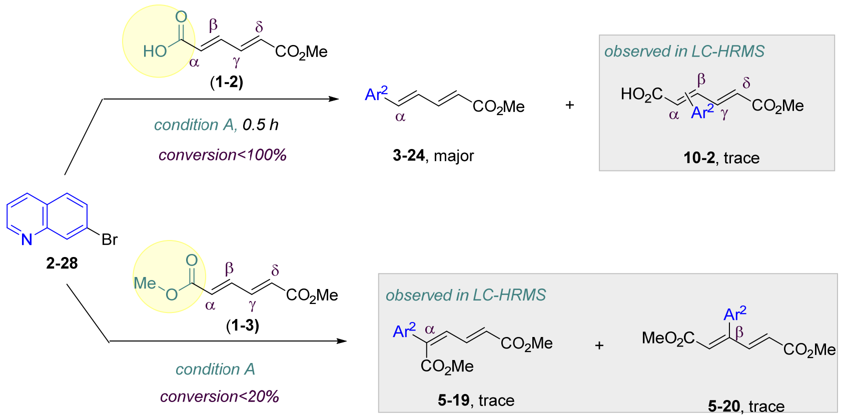

Supplementary Fig. 17 Control experiments between **1-2** and **1-3**

Meanwhile, during the substrate scope investigation between substrate **1-11** and **2-28**, a diene byproduct **9-2** was isolated, which could be reaction intermediate if the reaction followed the "Decarboxylation-Heck" mechanism. Thus, a background reaction with compound **1-11** without adding **2-24** was conducted, however, no compound **9-2** was observed in H-NMR of crude reaction mixture, but instead 70% starting material **1-11** remained untouched. These experiments indicated that the decarboxylation of compound **1-11** to form **9-2** was not that straightforward (Supplementary Fig.18).

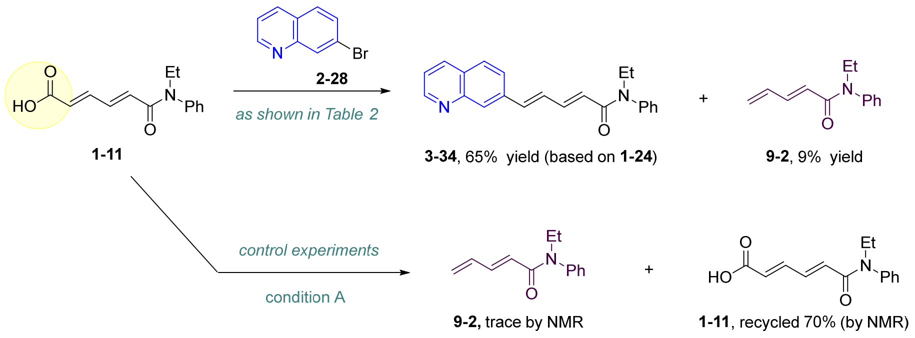

Supplementary Fig. 18 Experiment to probe Decarboxylation-Heck reaction mechanism

The direct decarboxylation of dieneoic acids is not known, but the similar reaction between (*Z*)-maleic acid **1-18** and aryl iodide **2-9** has been reported[10]. The "Heck-Decarboxylation" reaction pathway was proposed, given that compound **8-3** can smoothly transferred into product **8-2** under the same condition. Besides, this decarboxylation step should not be the rate-determining step (RDS), since compound **8-3** could not been observed by GC-MS in reaction mixture. Moreover, without Pd-catalyst, this decarboxylation process could not occur. But the "Decarboxylation-Heck" reaction pathway was partially

involved, considering that vinyl carboxylic acid also afforded product **8-2** under the same condition. Quite interestingly, the (*E*)-maleic acid cannot afford product under the same condition (Supplementary Fig.19).

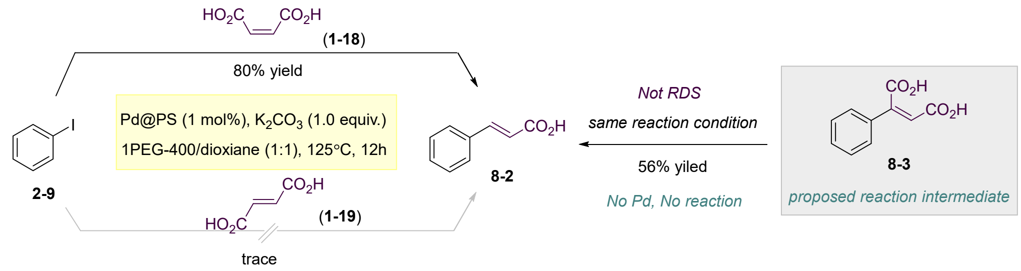

Supplementary Fig. 19 Previous research of decarboxylation of (*Z*)-maleic acid

Thus, in combining all the information from reported literature and our own mechanism studies, we hypothesized that the reaction mainly went through the carboxylate-directed "Heck-Decarboxylation" reaction mechanism. The carboxylic group not only promoted the reaction but also controlled the regioselectivity by directing effect. And the "Decarboxylation-Heck" reaction pathways might be partially involved in the reaction in a complicated manner.

VII.Spectrum analysis in the mechanism study

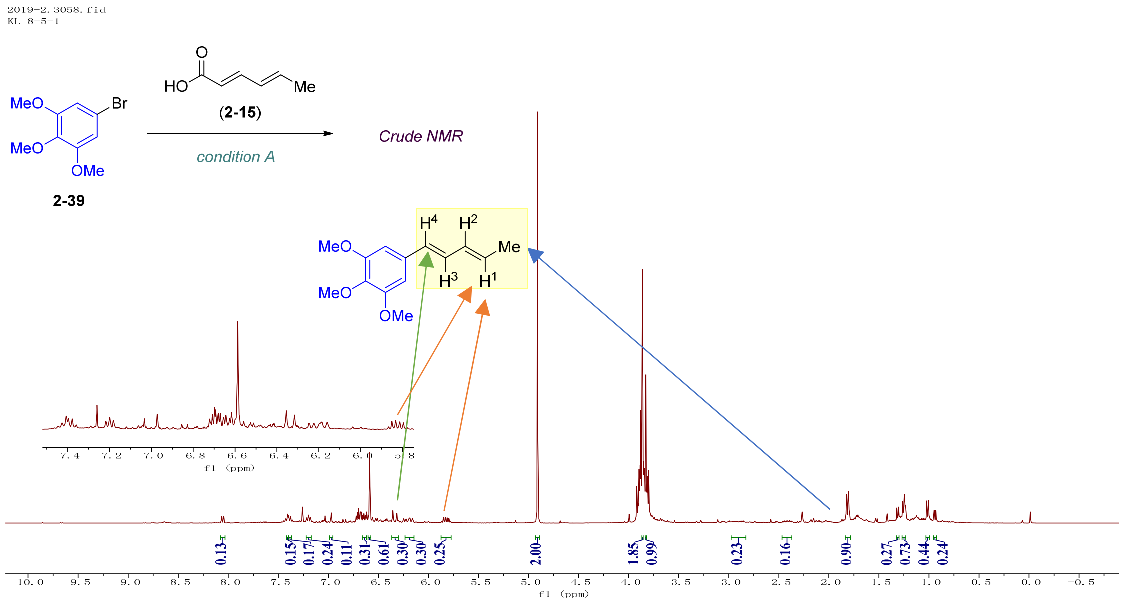

Supplementary Fig. 20

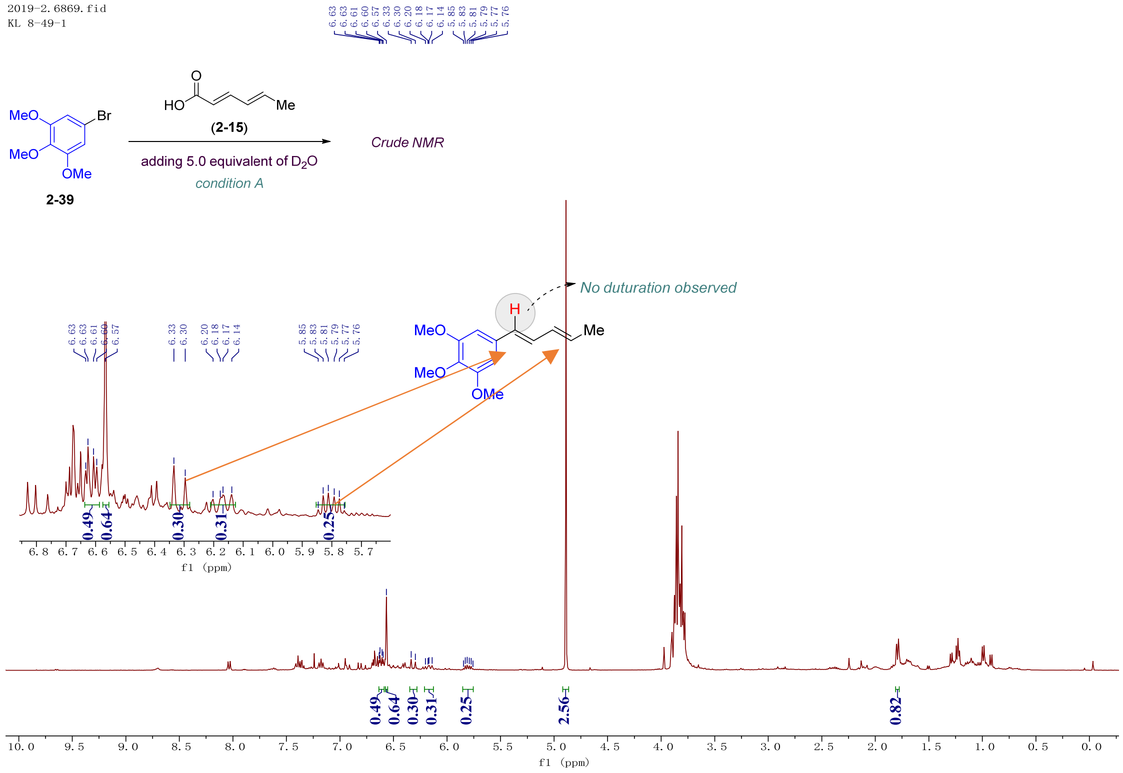

Supplementary Fig. 21

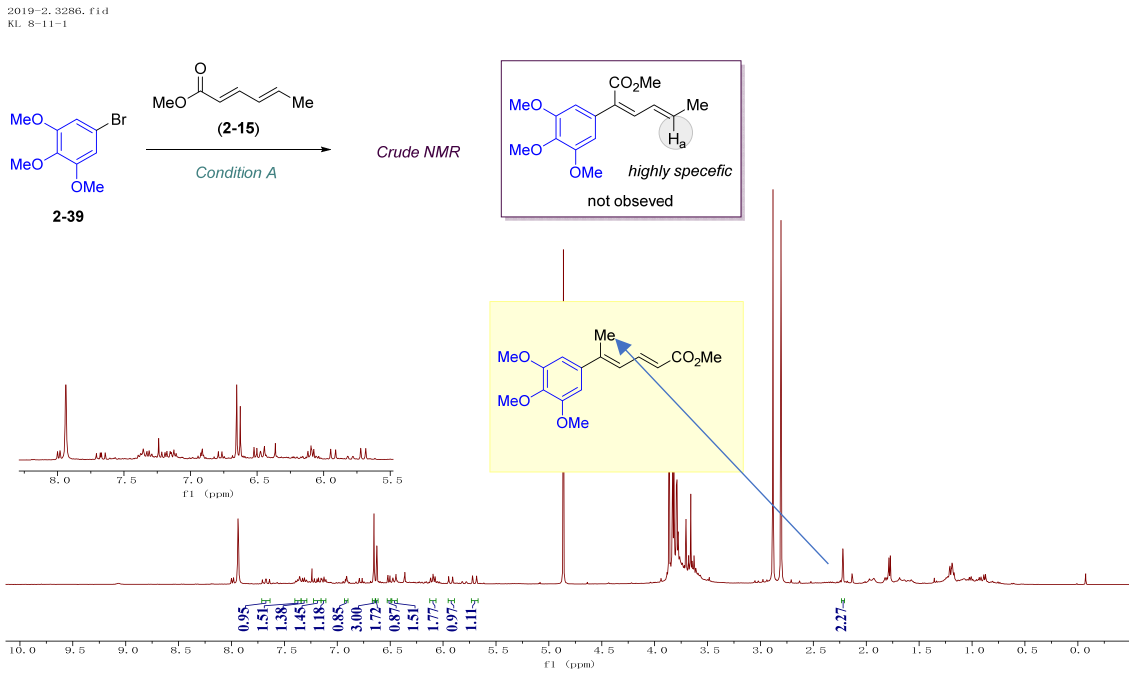

Supplementary Fig. 22

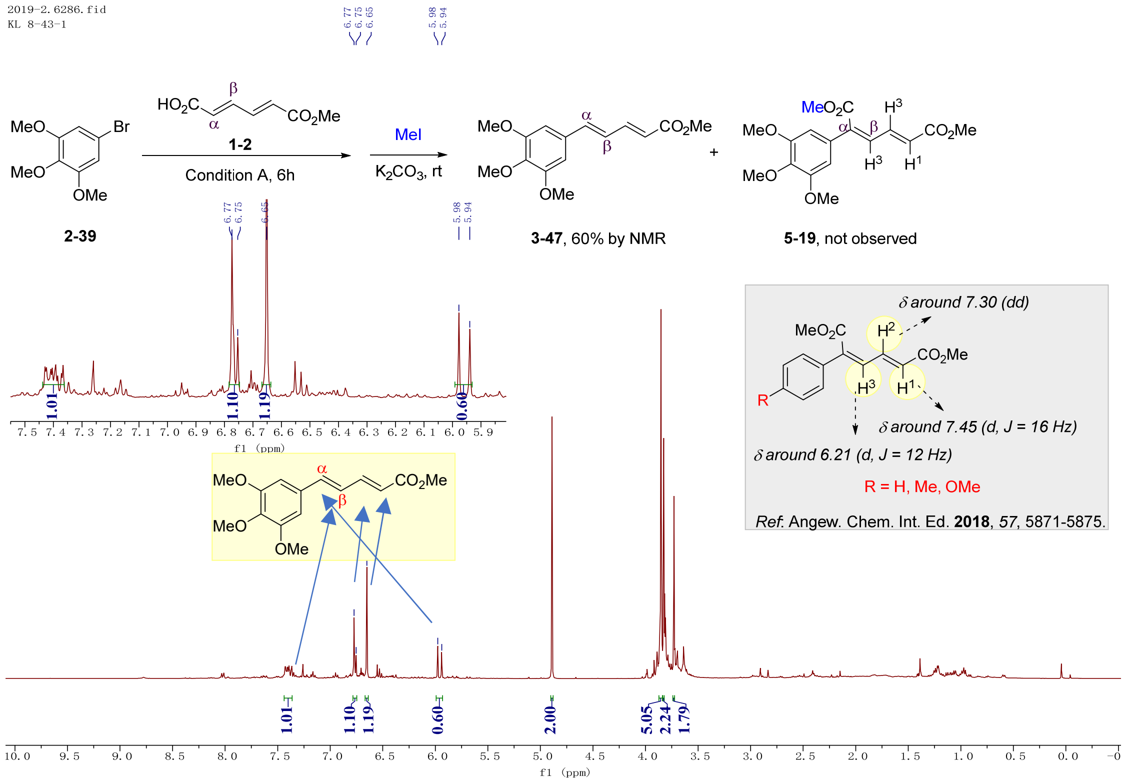

Supplementary Fig. 23

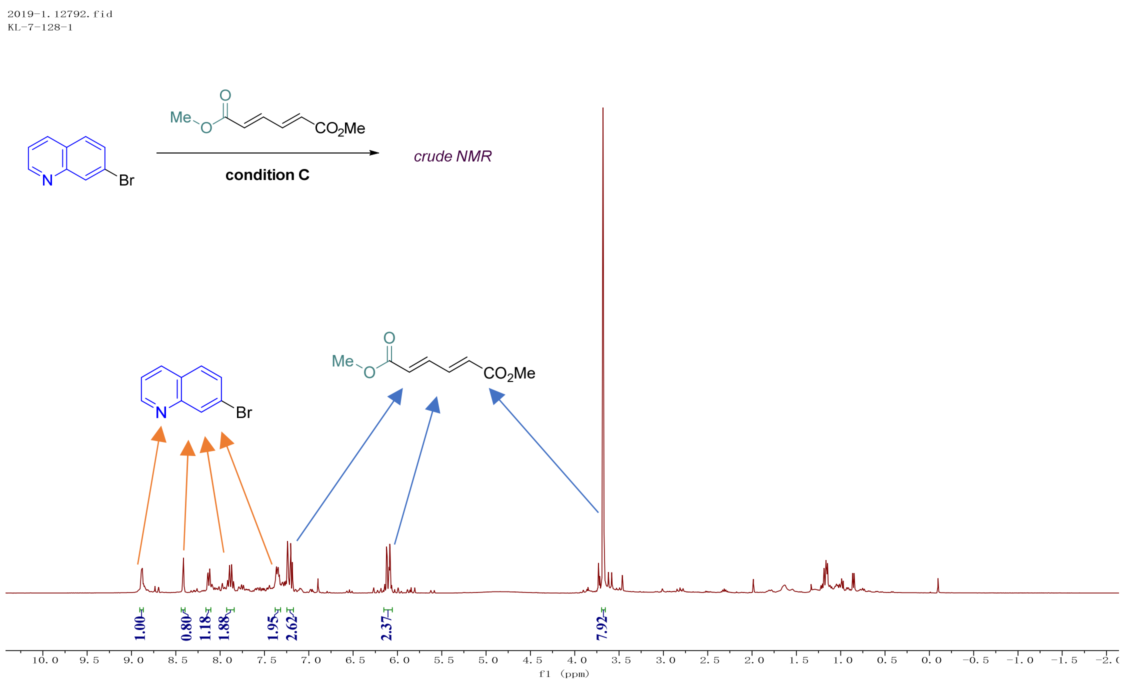

Supplementary Fig. 24

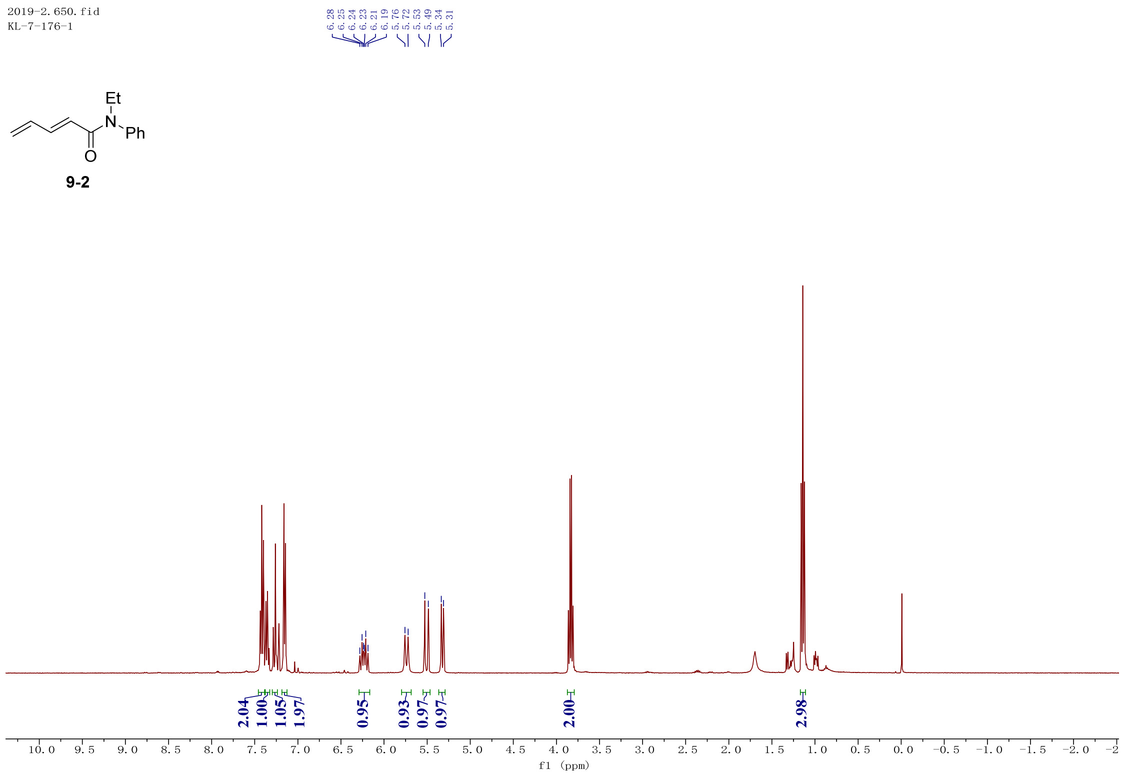

Supplementary Fig. 25

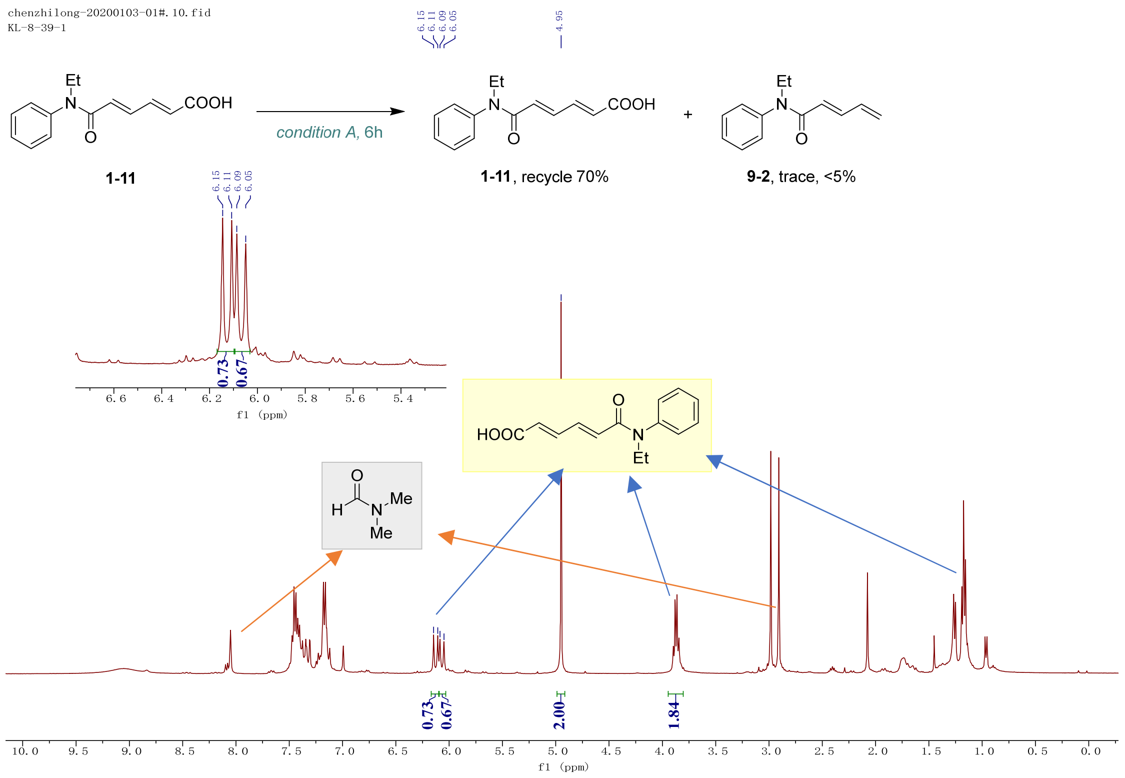

Supplementary Fig. 26

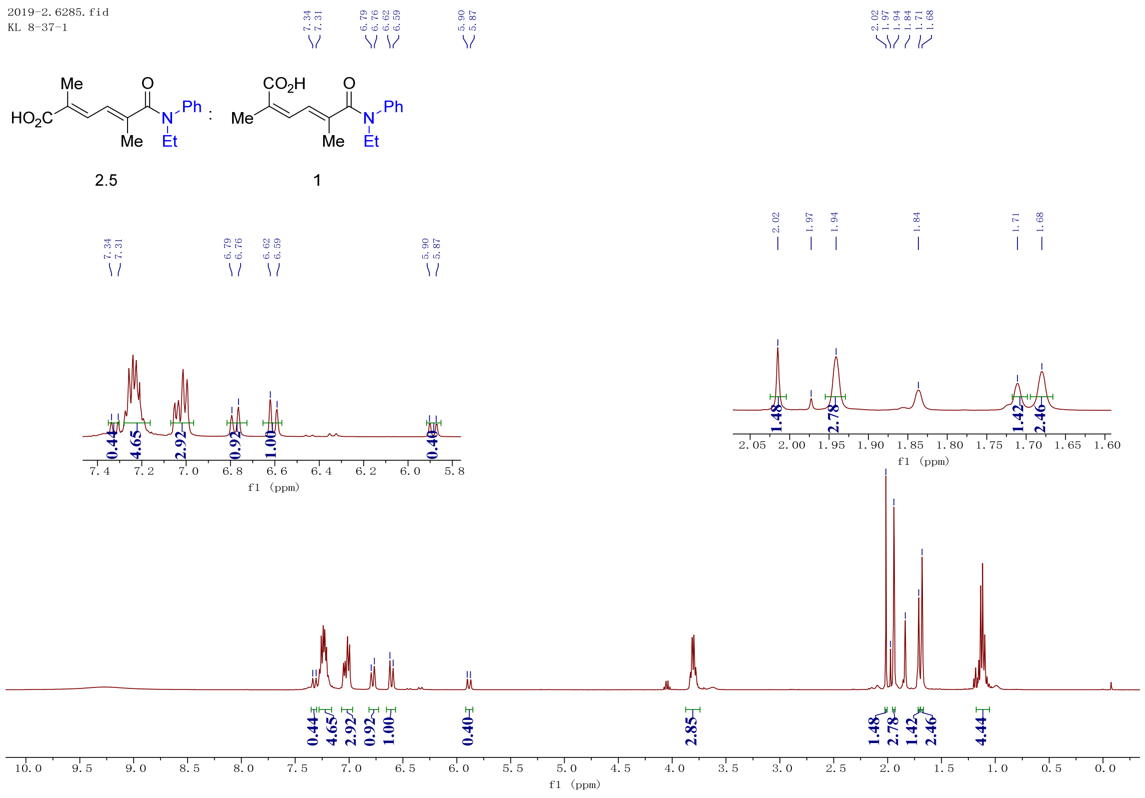

Supplementary Fig. 27

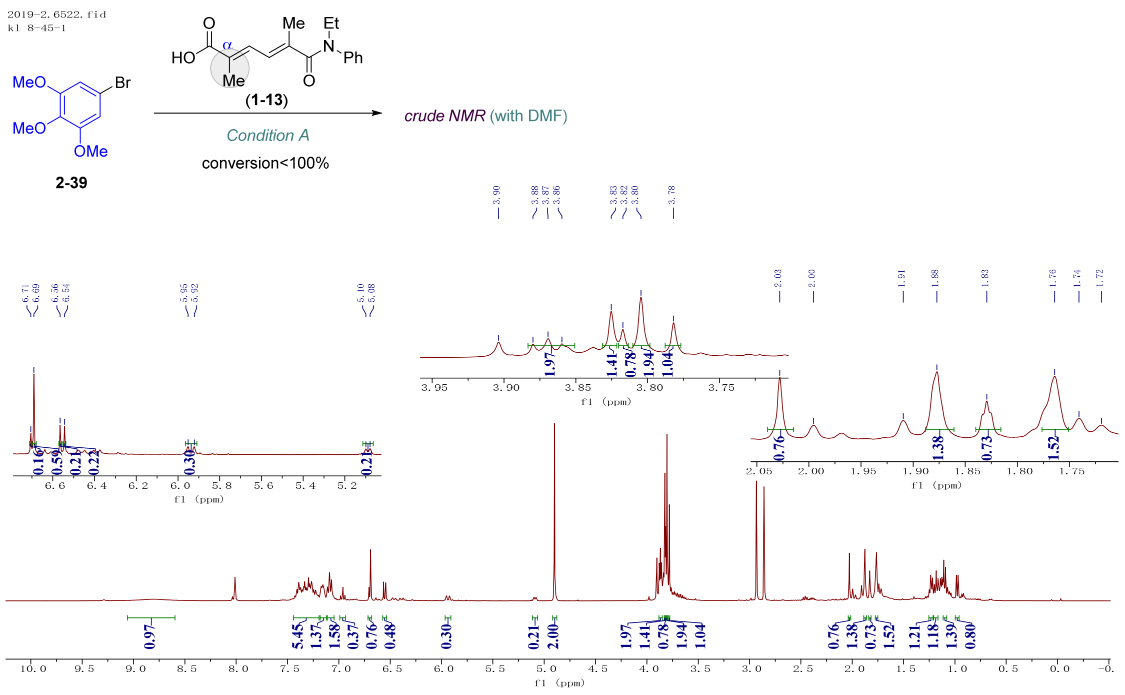

Supplementary Fig. 28

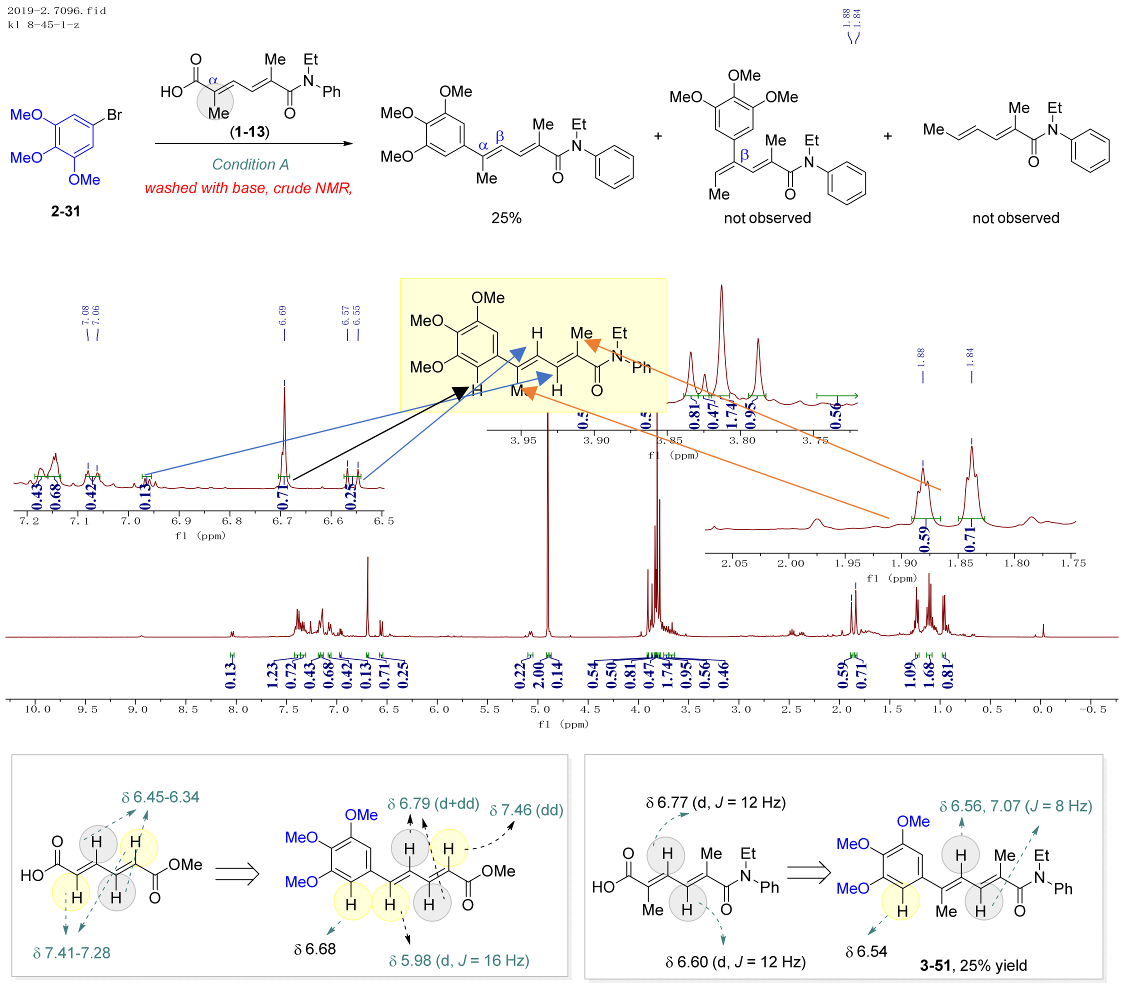

Supplementary Fig. 29

LC-HRMS analysis

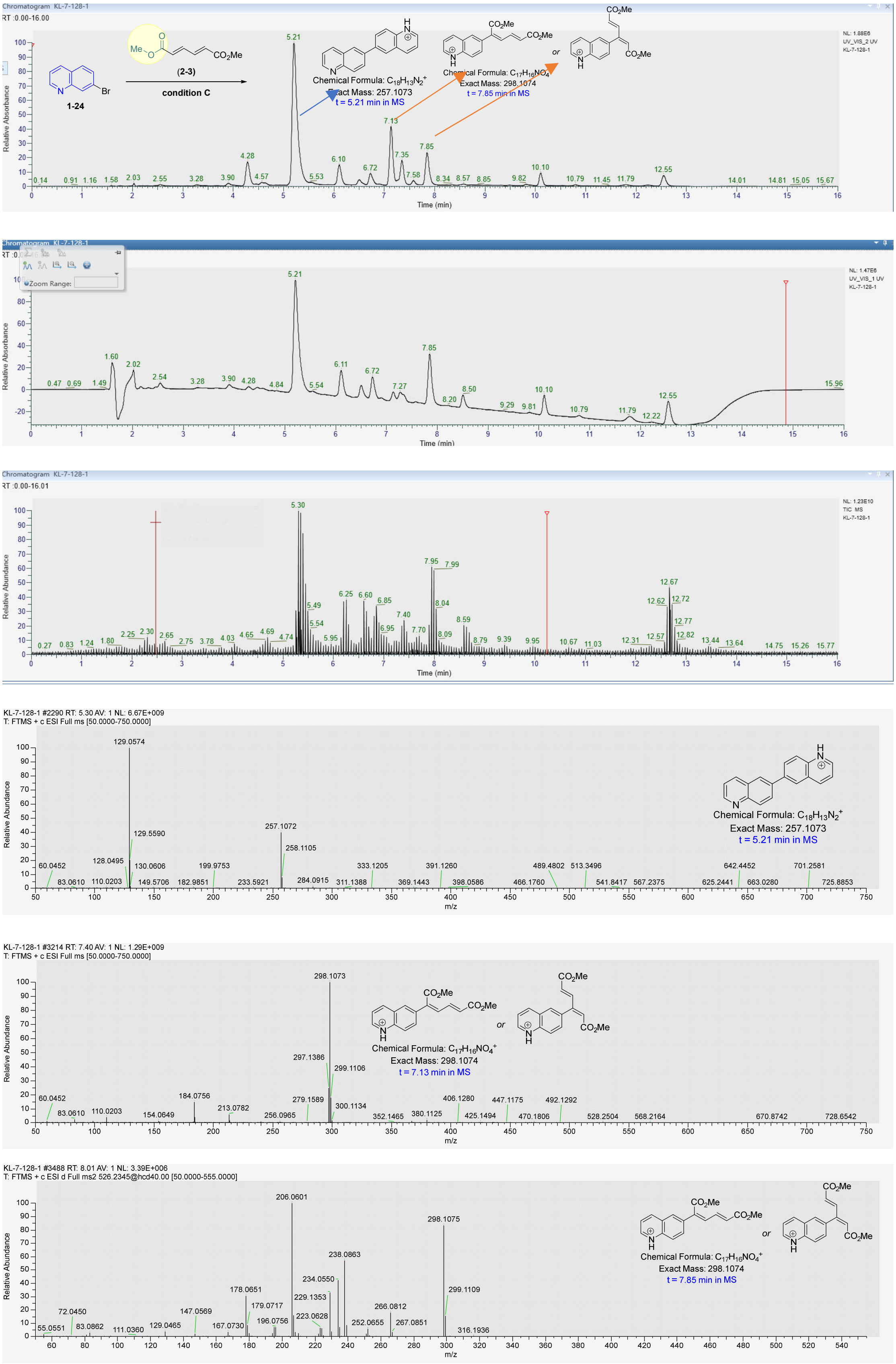

Supplementary Fig. 30

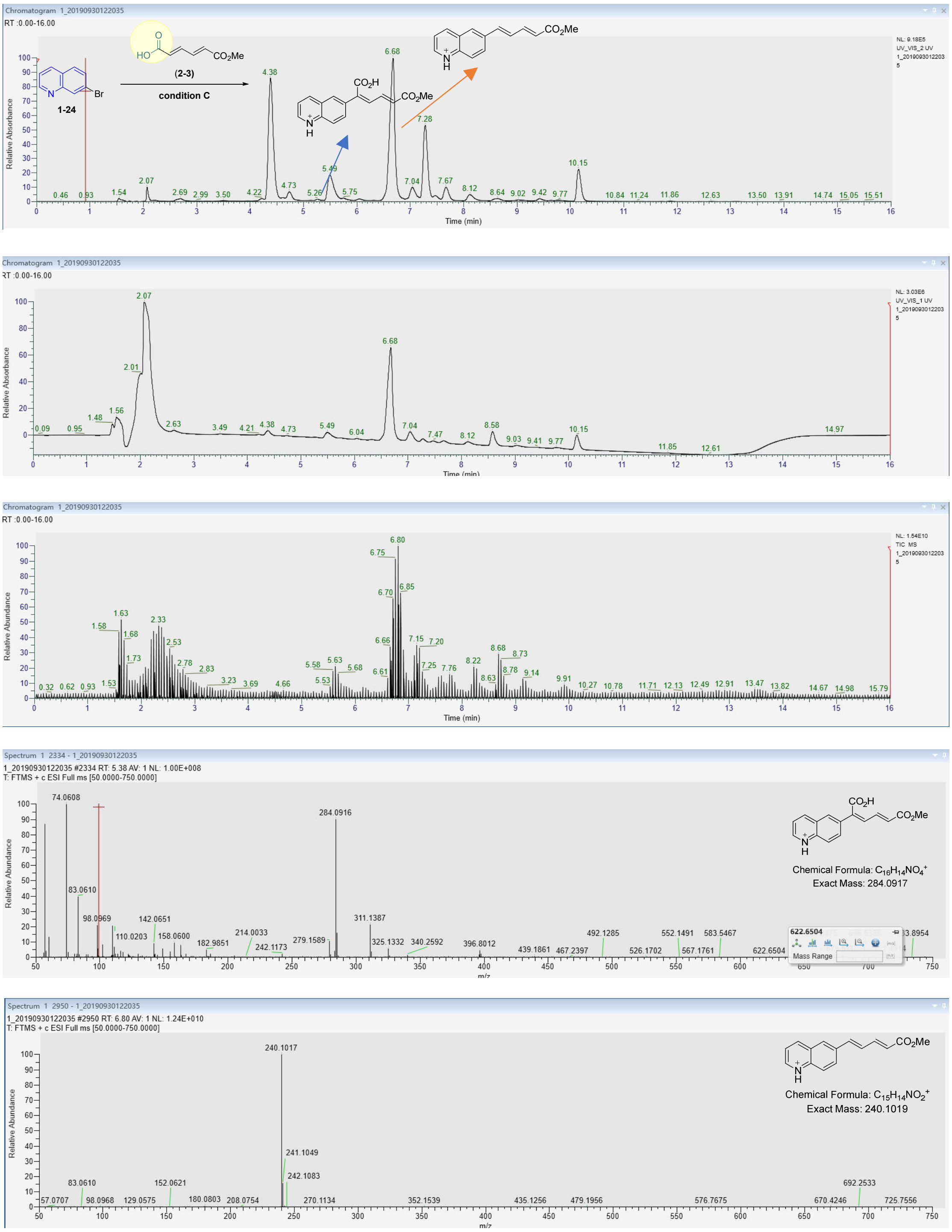

Supplementary Fig. 31

VIII. Cytotoxicity study

Cell culture Human non-small cell lung cancer A549 and H1299 cells, Human colon cancer HCT116 and HT29, Human leukemia K562 and MV-4-11 cells, and Human liver cancer HepG2 cells were purchased from ATCC (Manassas, VA). A549 cells was cultured in F-12K medium, containing 10% FBS, 100 U/mL penicillin and 100 μg/mL streptomycin at 37°C in a humidified atmosphere of 5% CO_2. HCT116 and HT29 cells were cultured in McCoy' 5A, containing 10% FBS, 100 U/mL penicillin and 100 μg/mL streptomycin at 37°C in a humidified atmosphere of 5% CO_2. K562 and H1299 cells were cultured in RPMI-1640, containing 10% FBS, 100 U/mL penicillin and 100 μg/mL streptomycin at 37°C in a humidified atmosphere of 5% CO_2. MV-4-11 cells were cultured in IMDM containing 10% FBS, 100 U/mL penicillin and 100 μg/mL streptomycin at 37°C in a humidified atmosphere of 5% CO_2. HepG2 cells were cultured in DMEM, containing 10% FBS, 5 mM Hepes, 2 mM Glutamine, 100 U/mL penicillin and 100 μg/mL streptomycin and stored at 4 °C, and then cultured in 37°C in a humidified atmosphere of 5% CO_2 before being treated with tested compounds.

Cytotoxicity Studies The cells were seeded in 96-well plates at certain density of cells/well (2000 cells/well for HCT116 and A549 cells; 3000 cells/well for HT29 cells; 4000 cells/well for H1299 cells; 5000 cells/well for HepG2 cells; 6000 cells/well for MV-4-11 cells; 8000 cells/well for K562 cells). After cultured for overnight, the cells were treated with 20 μL of tested compounds (30 μM) for 72 h. And then 10 μL of CCK-8 solution to each well, followed by incubating the plate for 1-4h at 37 °C in a humidified atmosphere of 5% CO_2. The absorbance at 450 nm was measured with a microplate reader (Nivo). Inhibitory rate of growth was calculated by the following formula: inhibitory rate (%) = (A_{450} of vehicle control – A_{450} of treated cells) / (A_{450} of vehicle control – A_{450} of blank control)*100.

IX. Characterization data of products and intermediates

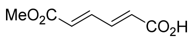

1-2

(2*E*, 4*E*)-6-methoxy-6-oxohexa-2,4-dienoic acid (1-2)[11] was prepared according to Supplementary Fig. 1 (1.06 g, 50% yield, 6.8 mmol scale) as white solid.

^{1}H NMR (400 MHz, DMSO-d_6) δ 12.66 (br, 1H), 7.42-7.30 (m, 2H), 6.47-6.32 (m, 2H), 3.71 (s, 3H).

^{13}C NMR (101 MHz, DMSO-d_6) δ 166.9, 166.0, 141.6, 140.7, 129.9, 127.6, 51.7 (two carbons)

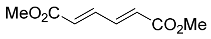

1-3

Diethyl (2*E*, 4*E*)-hexa-2,4-dienedioate (1-3)[1] was prepared according to Supplementary Fig. 1 (3.08 g, 45% yield, 18 mmol scale, *E*, *E*: *E*, *Z* = 3:1) as white solid.

^{1}H NMR (400 MHz, $CDCl_3$) 7.33-7.29 (m, 2H), 6.21-6.18 (m, 2H), 3.77 (s, 6H).

^{13}C NMR (101 MHz, $CDCl_3$) δ 166.3, 140.9, 128.0, 51.9

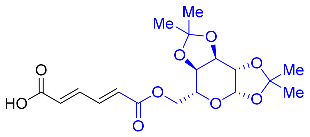

1-5

(2*E*, 4*E*)-6-oxo-6-(((3a*S*, 5*R*, 5a*R*, 8a*R*, 8b*S*)-2,2,7,7-tetramethyltetrahydro-5H-bis([1,3]dioxolo)[4,5-b:4',5'-d]pyran-5-yl)methoxy)hexa-2,4-dienoic acid (1-5) was prepared according to Supplementary Fig. 3 (261 mg, 23% yield, 0.68 mmol scale) as yellow oil.

H NMR (400 MHz, $CDCl_3$) δ 7.38-7.30 (m, 2H), 6.29-6.16 (m, 2H), 5.55 (d, *J* = 5.0 Hz, 1H), 4.63 (dd, *J* = 7.9, 2.5 Hz, 1H), 4.40-4.35 (m, 1H), 4.35-4.29 (m, 2H), 4.27-4.25 (m, 1H), 4.09-4.05 (m, 1H), 1.51 (s, 3H), 1.45 (s, 3H), 1.33 (d, *J* = 4.5 Hz, 6H)

^{13}C NMR (101 MHz, $CDCl_3$) δ 165.6, 142.7, 140.8, 128.8, 127.6, 109.7, 108.8, 96.3, 71.0, 70.7, 70.4, 65.9, 63.9, 60.4, 26.0, 25.9, 24.9, 24.5

HRMS (ESI, m/z)**:** Calcd for $C_{18}H_{23}O_9^-$ ($[M-H]^-$) 383.1348, found 383.1344.

IR (KBr, cm^{-1}): 2990, 1719, 1615, 1385, 1070, 1004, 895, 866

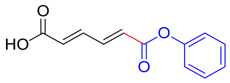

1-6

(2*E*, 4*E*)-6-oxo-6-phenoxyhexa-2,4-dienoic acid (1-6) was prepared according to Supplementary Fig. 3 (156 mg, 24% yield, 0.72 mmol scale) as yellow solid.

^{1}H NMR (400 MHz, DMSO-d_6) δ 12.73 (br, 1H), 7.57 (dd, *J* = 15.3, 11.3 Hz, 1H), 7.46-7.35 (m, 3H), 7.27 (t, *J* = 7.4 Hz, 1H), 7.18 (d, *J* = 8.0 Hz, 2H), 6.65 (d, *J* = 15.2 Hz, 1H), 6.43 (d, *J* = 15.2 Hz, 1H)

^{13}C NMR (101 MHz, DMSO-d_6) δ 166.8, 164.2, 150.3, 143.3, 140.4, 130.6, 129.6, 126.9, 126.0, 121.8

HRMS (ESI, m/z): Calcd for $C_{12}H_9O_4^-$ ($[M-H]^-$) 217.0506, found 217.0504.

IR (KBr, cm^{-1}): 2925, 1737, 1685, 1494, 1267, 1187, 1125, 1008

Mp.: 138.1-128.9℃.

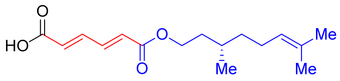

1-7

(2*E*, 4*E*)-6-(((*S*)-3,7-dimethyloct-6-en-1-yl)oxy)-6-oxohexa-2,4-dienoic acid (**1-7**) was prepared according to Supplementary Fig. 3 (310 mg, 37% yield, 1.11 mmol scale) as yellow oil.

^{1}H NMR (400 MHz, $CDCl_3$) δ 7.47-7.23 (m, 2H), 6.23 (dd, *J* = 14.7, 13.0 Hz, 2H), 5.13-5.04 (m, 1H), 4.31 -4.15 (m, 2H), 2.08-1.88 (m, 2H), 1.80-1.68 (m, 1H), 1.68 (s, 3H), 1.60 (s, 3H), 1.59-1.45 (m, 2H), 1.43-1.30 (m, 1H), 1.27-1.14 (m, 1H), 0.93 (d, *J* = 6.4 Hz, 3H)

^{13}C NMR (101 MHz, $CDCl_3$) δ 171.0, 165.8, 143.1, 140.2, 131.4, 129.5, 127.3, 124.5, 63.6, 36.9, 35.3, 29.5, 25.7, 25.3, 19.4, 17.6

HRMS (ESI, m/z): Calcd for $C_{16}H_{23}O_4^-$ ([M-H]$^-$) 279.1602, found 279.1599

IR (KBr, cm^{-1}): 2986, 2925, 1705, 1614, 1308, 1229, 1006, 858

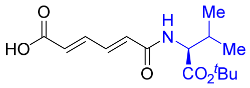

1-11

(2*E*, 4*E*)-6-(((*S*)-1-(tert-butoxy)-3-methyl-1-oxobutan-2-yl)amino)-6-oxohexa-2,4-dienoic acid (**1-11**) was prepared according to Supplementary Fig. 3 (221 mg, 99% yield, 0.75 mmol scale) as yellow solid.

H NMR (400 MHz, Methanol-*d*4) δ 7.38-7.23 (m, 2H), 6.56-6.52 (m, 1H), 6.23-6.19 (m, 1H), 4.32 (d, *J* = 5.8 Hz, 1H), 2.20-2.12 (m, 1H), 1.47 (s, 9H), 0.97 (d, *J* = 6.9 Hz, 6H)

^{13}C NMR (101 MHz, Methanol-*d*4) δ 172.1, 169.3, 167.4, 142.9, 138.8, 131.9, 128.8, 82.9, 60.0, 31.9, 28.3, 19.5, 18.5

HRMS (ESI, m/z): Calcd for $C_{15}H_{22}NO_5^-$ ([M-H]$^-$) 296.1503, found 296.1500

IR (KBr, cm^{-1}): 2979, 1715, 1605, 1298, 1271, 1153, 1003, 848

Mp.: 94.8-95.4℃.

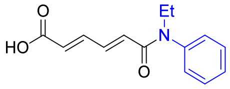

1-12

(2*E*, 4*E*)-6-(ethyl(phenyl)amino)-6-oxohexa-2,4-dienoic acid (**1-12**) was prepared according to Supplementary Fig. 3 (161mg, 22% yield, 0.66 mmol scale) as white solid.

H NMR (400 MHz, Methanol-*d*4) δ 7.51-740 (m, 3H), 7.25-7.22 (m, 3H), 7.06 (dd, *J* = 15.2, 11.4 Hz, 1H), 6.10 (dd, *J* = 26.3, 15.1 Hz, 2H), 3.84 (q, *J* = 7.1 Hz, 2H), 1.14 (t, *J* = 7.1 Hz, 3H)

^{13}C NMR (101 MHz, Methanol-*d*4) δ 169.2, 166.5, 142.8, 142.4, 139.5, 131.0, 130.4, 129.5, 129.4, 128.8, 45.7, 13.1

HRMS (ESI, m/z): Calcd for $C_{14}H_{14}NO_3^-$ ([M-H]$^-$) 244.0979, found 244.0977

IR (KBr, cm^{-1}): 2984, 1689, 1646, 1593, 1395, 1274, 1002, 702

Mp.: 105.8-107.1℃

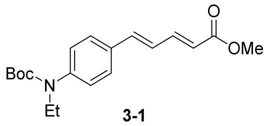

3-1

Methyl (2*E*, 4*E*)-5-(4-((*tert*-butoxycarbonyl)(ethyl)amino)phenyl)penta-2,4-dienoate (**3-1**) was

obtained according to **General Procedure A** as yellow solid (45.3 mg, 55% yield, **condition A**).

^{1}H NMR (400 MHz, $CDCl_3$) δ 7.45-7.41 (m, 3H), 7.19 (d, *J* = 8.3 Hz, 2H), 6.86-6.83 (m, 2H), 5.99 (d, *J* = 15.3 Hz, 1H), 3.77 (s, 3H), 3.68 (q, *J* = 7.1 Hz, 2H), 1.44 (s, 9H), 1.15 (t, *J* = 7.1 Hz, 3H)

^{13}C NMR (101 MHz, $CDCl_3$) δ 167.5, 154.2, 144.8, 143.2, 139.8, 133.3, 127.5, 126.9, 126.0, 120.6, 80.3, 51.6, 44.8, 28.3, 13.9

HRMS (ESI, m/z) calcd for $C_{19}H_{25}NO_4^+$ ([M + H]$^+$) 332.1856, found 332.1857

IR (KBr, cm^{-1}): 2976, 2930, 1708, 1689, 1406, 1250, 1140, 999, 863

M.p.: 84.6-85.2℃.

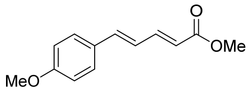

3-2

Methyl (2*E*, 4*E*)-5-(4-methoxyphenyl)penta-2,4-dienoate (**3-2**)[12] was obtained according to **General Procedure A** as yellow solid (43mg, 49% yield for **Condition A**).

^{1}H NMR (400 MHz, $CDCl_3$) δ 7.48-7.38 (m, 3H), 6.91-6.83 (m, 3H), 6.75 (dd, *J* = 15.4, 10.9 Hz, 1H), 5.94 (d, *J* = 15.2 Hz, 1H), 3.83 (s, 3H), 3.76 (s, 3H)

^{13}C NMR (101 MHz, $CDCl_3$) δ 167.7, 160.4, 145.3, 140.3, 128.8, 128.6, 124.1, 119.5, 114.3, 55.3, 51.5

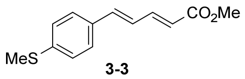

3-3

Methyl (2*E*, 4*E*)-5-(4-(methylthio)phenyl)penta-2,4-dienoate (3-3) was obtained according to **General Procedure A** as yellow solid (37.4 mg, 64% yield for **Condition A**).

^{1}H NMR (400 MHz, $CDCl_3$) δ 7.43 (ddd, *J* = 15.3, 7.2, 3.1 Hz, 1H), 7.38-7.36 (m, 2H), 7.21-7.19 (m, 2H), 6.83 (d, *J* = 7.2 Hz, 2H), 5.97 (d, *J* = 15.3 Hz, 1H), 3.76 (s, 3H), 2.49 (s, 3H)

^{13}C NMR (101 MHz, $CDCl_3$) δ 167.4, 144.8, 140.1, 139.9, 132.7, 127.5, 126.2, 125.4, 120.3, 51.5, 15.3

HRMS (ESI, m/z) calcd for $C_{13}H_{15}O_2S^+$ ([M + H]$^+$): 235.0787, found 235.0788

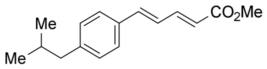

3-4

Methyl (2*E*, 4*E*)-5-(4-isobutylphenyl)penta-2,4-dienoate (3-4) was obtained according to **General Procedure A** as white solid (31.5 mg, 52% yield for **Condition A**).

^{1}H NMR (400 MHz, $CDCl_3$) δ 7.48-7.42 (m, 1H), 7.38 (d, *J* = 8.1 Hz, 2H), 7.13 (d, *J* = 7.9 Hz, 2H), 6.91-6.80 (m, 2H), 5.97 (d, *J* = 15.3 Hz, 1H), 3.76 (s, 3H), 2.47 (d, *J* = 7.2 Hz, 2H), 1.86 (dp, *J* = 13.6, 7.1 Hz, 1H), 0.90 (d, *J* = 6.6 Hz, 6H)

^{13}C NMR (101 MHz, $CDCl_3$) δ 167.6, 145.1, 143.2, 140.7, 133.6, 129.6, 127.0, 125.3, 120.2, 51.5, 45.3, 30.2, 22.3

HRMS (ESI, m/z) calcd for $C_{16}H_{21}O_2^+$ ([M + H]$^+$): 245.1536, found 245.1531

IR (KBr, cm^{-1}): 2923, 1710, 1627, 1243, 1133, 997, 844, 703

M.p.: 67.6-68.5℃

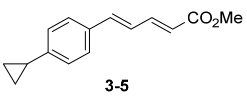

3-5

Methyl (2*E*, 4*E*)-5-(4-cyclopropylphenyl)penta-2,4-dienoate[13] (**3-5**) was obtained according to **General Procedure A** as yellow solid (28 mg, 49% yield for **Condition A**).

^{1}H NMR (400 MHz, $CDCl_3$) δ 7.44 (dd, *J* = 15.3, 9.7 Hz, 1H), 7.35 (d, *J* = 8.1 Hz, 2H), 7.04 (d, *J* = 8.1 Hz, 2H), 6.89-6.76 (m, 2H), 5.96 (d, *J* = 15.3 Hz, 1H), 3.77 (s, 3H), 1.94-1.85 (m, 1H), 1.02-0.95 (m, 2H), 0.76-0.67 (m, 2H)

^{13}C NMR (101 MHz, $CDCl_3$) δ 167.5, 145.6, 145.1, 140.5, 133.2, 127.2, 125.9, 125.1, 120.0, 51.5, 15.4, 9.7

HRMS (ESI, m/z) calcd for $C_{15}H_{17}O_2^+$ ([M + H]$^+$) 229.1223, found 229.1223.

IR (KBr, cm^{-1}): 3440, 2997, 1709, 1602, 1241, 1139, 1024, 810

M.p.: 82.5-83.4℃

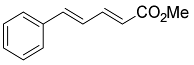

3-6

Methyl (2*E*, 4*E*)-5-phenylpenta-2,4-dienoate (**3-6**) was obtained according to **General Procedure A** as white solid (39 mg, 52% yield for **Condition B**).

^{1}H NMR (400 MHz, $CDCl_3$) δ 7.48-7.43 (m, 3H), 7.38-7.30 (m, 3H), 6.89 (d, *J* = 8.5 Hz, 2H), 6.00 (d, *J* = 15.3 Hz, 1H), 3.77 (s, 3H)

^{13}C NMR (101 MHz, $CDCl_3$) δ 167.5, 144.8, 140.5, 136.0, 129.1, 128.8, 127.2, 126.2, 120.8, 51.6

HRMS (ESI, m/z) calcd for $C_{12}H_{13}O_2^+$ ([M + H]$^+$) 189.0910, found 189.0909

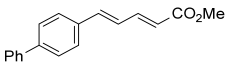

3-7

Methyl (2*E*, 4*E*)-5-([1,1'-biphenyl]-4-yl)penta-2, 4-dienoate (**3-7**) was obtained according to **General Procedure A** as yellow solid (31.9 mg, 48% yield for **Condition A**).

^{1}H NMR (400 MHz, $CDCl_3$) δ 7.63-7.59 (m, 4H), 7.54 (d, *J* = 8.4 Hz, 2H), 7.50-7.43 (m, 3H), 7.39-7.34 (m, 1H), 6.93 (d, *J* = 8.0 Hz, 2H), 6.02 (d, *J* = 15.2 Hz, 1H), 3.79 (s, 3H)

^{13}C NMR (101 MHz, $CDCl_3$) δ 167.5, 144.9, 141.7, 140.3, 140.1, 135.0, 128.8, 127.64, 127.61, 127.4, 126.9, 126.1, 120.7, 51.6

HRMS (ESI, m/z) calcd for $C_{18}H_{17}O_2^+$ ([M + H]$^+$) 265.1223, found 265.1223.

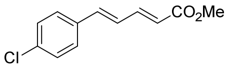

3-8

Methyl (2*E*, 4*E*)-5-(4-chlorophenyl)penta-2, 4-dienoate (**3-8**) was obtained according to **General Procedure A** as white solid (33.8 mg, 39% yield for **Condition B**).

^{1}H NMR (400 MHz, $CDCl_3$) δ 7.44-7.40 (m, 1H), 7.39-7.36 (m, 2H), 7.33-7.30 (m, 2H), 6.83-6.82 (m, 2H), 5.99 (d, *J* = 15.3 Hz, 1H), 3.76 (s, 3H)

^{13}C NMR (101 MHz, $CDCl_3$) δ 167.3, 144.4, 139.0, 134.7, 134.4, 129.0, 128.3, 126.7, 121.3, 51.6

HRMS (ESI, m/z) calcd for $C_{12}H_{12}ClO_2^+$ ([M + H]$^+$) 223.0520, found 233.0520

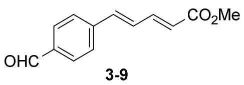

3-9

Methyl (2*E*, 4*E*)-5-(4-formylphenyl)penta-2, 4-dienoate (3-9) was obtained according to **General Procedure A** as yellow solid (15 mg, 28% yield for **Condition A**).

^{1}H NMR (400 MHz, $CDCl_3$) δ 9.99 (s, 1H), 7.86 (d, *J* = 8.1 Hz, 2H), 7.60 (d, *J* = 8.1 Hz, 2H), 7.45 (dd, *J* = 15.3, 10.3 Hz, 1H), 7.03-6.90 (m, 2H), 6.07 (d, *J* = 15.3 Hz, 1H), 3.78 (s, 3H)

^{13}C NMR (101 MHz, $CDCl_3$) δ 191.4, 167.1, 143.8, 141.8, 138.6, 136.2, 130.1, 129.3, 127.6, 122.9, 51.7

HRMS (ESI, m/z) calcd for $C_{13}H_{13}O_3^+$ ($[M + H]^+$) 217.0859, found 217.0860

IR (KBr, cm^{-1}): 2952, 1712, 1697, 1625, 1213, 1137, 992, 811

M.p.: 93.9-94.8℃.

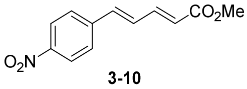

3-10

Methyl (2*E*,4*E*)-5-(4-nitrophenyl)penta-2,4-dienoate (**3-10**) was obtained according to **General Procedure A** as yellow solid (16.9 mg, 29% yield for **Condition A**).

^{1}H NMR (400 MHz, $CDCl_3$) δ 8.23-8.21 (m, 2H), 7.62-7.59 (m, 2H), 7.48-7.44 (m, 1H), 7.04-6.91 (m, 2H), 6.11 (d, *J* = 15.3 Hz, 1H), 3.79 (s, 3H)

^{13}C NMR (101 MHz, $CDCl_3$) δ 167.0, 147.6, 143.4, 142.2, 137.4, 130.2, 127.6, 124.2, 123.7, 51.8

HRMS (ESI, m/z): Calcd for $C_{12}H_{12}NO_4^+$ ($[M + H]^+$) 234.0761, found 234.0765

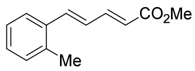

3-11

Methyl (2*E*, 4*E*)-5-(o-tolyl)penta-2,4-dienoate (**3-11**) was obtained according to **General Procedure A** as yellow oil (62.1 mg, 77% yield for **Condition B**).

^{1}H NMR (400 MHz, $CDCl_3$) δ 7.56-7.45 (m, 2H), 7.23-7.14 (m, 4H), 6.80 (ddd, *J* = 15.5, 11.1, 0.8 Hz, 1H), 6.00 (d, *J* = 15.3 Hz, 1H), 3.78 (s, 3H), 2.39 (s, 3H)

^{13}C NMR (101 MHz, $CDCl_3$) δ 167.5, 145.1, 138.2, 136.5, 134.9, 130.7, 128.9, 127.2, 126.3, 125.6, 120.6, 51.5, 19.7

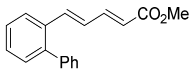

3-12

Methyl (2*E*, 4*E*)-5-([1,1'-biphenyl]-2-yl)penta-2,4-dienoate (**3-12**) was obtained according to **General Procedure A** as yellow oil (44.7 mg, 68% yield for **Condition B**).

^{1}H NMR (400 MHz, $CDCl_3$) δ 7.73-7.67 (m, 1H), 7.45-7.36 (m, 5H), 7.32 (tt, *J* = 7.7, 1.9 Hz, 4H), 6.92-6.80 (m, 2H), 5.97 (d, *J* = 15.3 Hz, 1H), 3.75 (s, 3H)

^{13}C NMR (101 MHz, $CDCl_3$) δ 167.5, 145.1, 142.1, 140.2, 139.5, 133.9, 130.4, 129.80, 128.7, 128.2, 127.6, 127.4, 126.8, 125.9, 120.4, 51.5

HRMS (ESI, m/z): Calcd for $C_{18}H_{17}O_2^+$ ($[M + H]^+$) 265.1223, found 265.1228

IR (KBr, cm^{-1}): 3023, 1713, 1624, 1435, 1267, 1139, 1002, 703

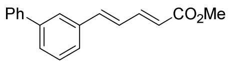

3-13

Methyl (2*E*, 4*E*)-5-([1,1'-biphenyl]-3-yl)penta-2, 4-dienoate (**3-13**) was obtained according to **General Procedure A** as white solid (34.4 mg, 53% yield for **Condition B**).

^{1}H NMR (400 MHz, $CDCl_3$) δ 7.67 (s, 1H), 7.62-7.58 (m, 2H), 7.55-7.51 (m, 1H), 7.52-7.41 (m, 5H), 7.41 -7.36 (m, 1H), 6.96 (d, *J* = 8.5 Hz, 2H), 6.02 (d, *J* = 15.3 Hz, 1H), 3.78 (s, 3H)

^{13}C NMR (101 MHz, $CDCl_3$) δ 167.4, 144.7, 141.9, 140.6, 140.4, 136.4, 129.2, 128.8, 127.9, 127.6, 126.5, 126.02, 125.96, 121.0, 51.6

HRMS (ESI, m/z): Calcd for $C_{18}H_{17}O_2^+$ ([M + H]$^+$) 265.1223, found 265.1223

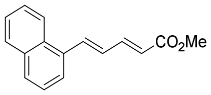

3-14

Methyl (2*E*, 4*E*)-5-(naphthalen-1-yl)penta-2,4-dienoate (**3-14**) was obtained according to **General Procedure A** as yellow oil (29.1 mg, 49% yield for **Condition B**).

^{1}H NMR (400 MHz, $CDCl_3$) δ 8.13 (dd, *J* = 8.4, 1.3 Hz, 1H), 7.89-7.84 (m, 2H), 7.76-7.68 (m, 2H), 7.62-7.47 (m, 4H), 6.97 (dd, *J* = 14.9, 11.5 Hz, 1H), 6.06 (d, *J* = 15.3 Hz, 1H), 3.81 (s, 3H)

^{13}C NMR (101 MHz, $CDCl_3$) δ 167.5, 144.4, 137.2, 133.7, 133.2, 131.1, 129.4, 128.8, 128.7, 126.6, 126.0, 125.5, 124.1, 123.2, 121.0, 51.6

HRMS (ESI, m/z): Calcd for $C_{14}H_{11}O_3$ ([M + H]$^+$) 265.1223, found 265.1223.

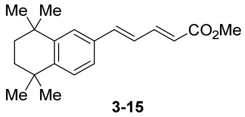

3-15

Methyl (2*E*, 4*E*)-5-(5,5,8,8-tetramethyl-5,6,7,8-tetrahydronaphthalen-2-yl)penta-2,4-dienoate (**3-15**) was obtained according to **General Procedure A** as pale yellow oil (36.5mg, 49% yield for **Condition A**).

^{1}H NMR (400 MHz, $CDCl_3$) δ 7.40 (dd, *J* = 15.3, 9.8 Hz, 1H), 7.31 (d, *J* = 1.7 Hz, 1H), 7.25-7.19 (m, 2H), 6.86-6.76 (m, 2H), 5.93 (d, *J* = 15.2 Hz, 1H), 3.72 (s, 3H), 1.64 (s, 4H), 1.24 (d, *J* = 9.2 Hz, 12H).

^{13}C NMR (101 MHz, $CDCl_3$) δ 167.6, 146.5, 145.4, 145.2, 141.0, 133.2, 127.1, 126.0, 125.1, 124.0, 120.0, 51.5, 35.0, 34.9, 34.4, 34.2, 31.8, 31.7

HRMS (ESI, m/z): Calcd for $C_{20}H_{27}O_2^+$ ([M + H]$^+$) 299.2006, found 299.2006.

IR (KBr, cm^{-1}): 3419, 2935, 1890, 1435, 1371, 1212, 804

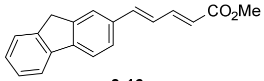

3-16

Methyl (2*E*, 4*E*)-5-(9H-fluoren-2-yl)penta-2,4-dienoate (**3-16**) was obtained according to **General Procedure A** as a yellow solid (31.9mg, 46% yield for **Condition A**).

^{1}H NMR (400 MHz, $CDCl_3$) δ 7.76 (dd, *J* = 15.3, 7.7 Hz, 2H), 7.64 (s, 1H), 7.56-7.45 (m, 3H), 7.39 (t, *J* = 7.4 Hz, 1H), 7.37-7.28 (m, 1H), 7.01-6.87 (m, 2H), 6.00 (d, *J* = 15.2 Hz, 1H), 3.89 (s, 2H), 3.79 (s, 3H)

^{13}C NMR (101 MHz, $CDCl_3$) δ 167.6, 145.1, 143.8, 143.7, 142.9, 141.05, 141.01, 134.6, 127.2, 126.9, 126.5, 125.5, 125.1, 123.6, 120.2, 120.14, 120.10, 51.5, 36.8

HRMS (ESI, m/z): Calcd for $C_{19}H_{17}O_2^+$ ([M + H]$^+$) 277.1223, found 277.1220

IR (KBr, cm^{-1}): 2961, 1714, 1625, 1332, 1243, 1138, 1002, 734

M.p.:122.6-123.1℃.

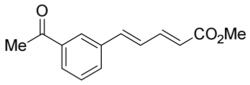

3-17

Methyl (2*E*, 4*E*)-5-(3-acetylphenyl)penta-2,4-dienoate (**3-17**) was obtained according to **General Procedure A** as white solid (17.2 mg, 30% yield for **Condition A**).

^{1}H NMR (400 MHz, $CDCl_3$) δ 8.04 (t, *J* = 1.8 Hz, 1H), 7.88 (dt, *J* = 7.8, 1.4 Hz, 1H), 7.65 (dt, *J* = 7.8, 1.5 Hz, 1H), 7.50-7.42 (m, 2H), 6.99-6.90 (m, 2H), 6.04 (d, *J* = 15.2 Hz, 1H), 3.78 (s, 3H), 2.63 (s, 3H)

^{13}C NMR (101 MHz, $CDCl_3$) δ 197.7, 167.3, 144.2, 139.1, 137.5, 136.5, 131.4, 129.1, 128.7, 127.4, 126.7, 121.7, 51.7, 26.7

HRMS m/z (ESI) Calcd for $C_{14}H_{15}O_3^+$([M + H]$^+$) 231.1016, found 231.1013

IR (KBr, cm^{-1}): 2923, 1713, 1681, 1627, 1263, 1246, 1139, 1012

M.p.: 78.8-79.6℃

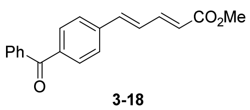

3-18

Methyl (2*E*, 4*E*)-5-(4-benzoylphenyl)penta-2,4-dienoate (**3-18**) was obtained according to **General Procedure A** as yellow solid (46.7 mg, 64% yield for **Condition C**).

^{1}H NMR (400 MHz, $CDCl_3$) δ 7.82-7.76 (m, 4H), 7.62-7.54 (m, 3H), 7.52-7.45 (m, 3H), 7.03-6.90 (m, 2H), 6.07 (d, *J* = 15.3 Hz, 1H), 3.78 (s, 3H).

^{13}C NMR (101 MHz, $CDCl_3$) δ 195.9, 167.2, 144.1, 139.8, 139.0, 137.4, 132.5, 130.6, 139.9, 128.5, 128.32, 128.29, 126.9, 122.3, 51.6

HRMS m/z (ESI) Calcd for $C_{19}H_{17}O_3^+$([M + H]$^+$) 293.1172, found 293.1169

IR (KBr, cm^{-1}): 2923, 1718, 1644, 1626, 1287, 1134, 996, 789

M.p.: 137.0-137.9℃

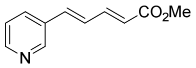

3-19

Methyl (2*E*, 4*E*)-5-(pyridin-3-yl)penta-2,4-dienoate (**3-19**)[13] was obtained according to **General Procedure A** as yellow solid (46.5 mg, 62% yield for **Condition A**).

^{1}H NMR (400 MHz, $CDCl_3$) δ 8.67 (s, 1H), 8.51 (d, *J* = 1.4 Hz, 1H), 7.82-7.74 (m, 1H), 7.44 (dd, *J* = 15.3, 9.9 Hz, 1H), 7.29 (dd, *J* = 8.0, 4.8 Hz, 1H), 6.97-6.83 (m, 2H), 6.04 (d, *J* = 15.3 Hz, 1H), 3.78 (s, 3H)

^{13}C NMR (101 MHz, $CDCl_3$) δ 167.2, 149.7, 149.0, 143.9, 136.4, 133.3, 131.7, 128.1, 123.6, 122.2, 51.7

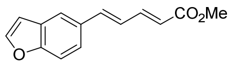

3-21

Methyl (2*E*, 4*E*)-5-(benzofuran-5-yl)penta-2,4-dienoate (**3-21**) was obtained according to **General Procedure A** as a white solid (26.1 mg, 46% yield for **Condition A**).

^{1}H NMR (400 MHz, $CDCl_3$) δ 7.68 (s, 1H), 7.63 (d, *J* = 2.2 Hz, 1H), 7.52-7.41 (m, 3H), 7.00 (d, *J* = 15.5 Hz, 1H), 6.86 (dd, *J* = 15.5, 10.9 Hz, 1H), 6.76 (d, *J* = 2.1 Hz, 1H), 5.99 (d, *J* = 15.2 Hz, 1H), 3.78 (s, 3H)
^{13}C NMR (101 MHz, $CDCl_3$) δ 167.6, 155.4, 145.8, 145.1, 141.0, 131.1, 128.0, 125.1, 123.5, 120.4, 120.0, 111.8, 106.7, 51.6
HRMS m/z (ESI) calcd for $C_{14}H_{13}O_3^+$ ([M + H]$^+$) 229.0859, found 229.0859.
M.p.: 93.6-99.5℃

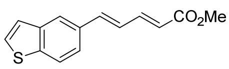

3-22

Methyl (2*E*, 4*E*)-5-(benzo[b]thiophen-5-yl)penta-2,4-dienoate (**3-22**) was obtained according to **General Procedure A** as a yellow solid (27.6mg, 45% yield for **Condition A**).
^{1}H NMR (400 MHz, $CDCl_3$) δ 7.86-7.83 (m, 2H), 7.52-7.45 (m, 3H), 7.33 (d, *J* = 5.4 Hz, 1H), 7.03-6.90 (m, 2H), 6.01 (d, *J* = 15.2 Hz, 1H), 3.78 (s, 3H)
^{13}C NMR (101 MHz, $CDCl_3$) δ 167.5, 144.9, 140.8, 140.3, 140.0, 132.4, 127.3, 125.8, 124.0, 122.9, 122.74, 122.65, 120.4, 51.5
HRMS m/z (ESI) calcd for $C_{14}H_{13}O_2S^+$ ([M + H]$^+$) 245.0631, found 245.0628
IR (KBr, cm^{-1}): 2960, 1711, 1627, 1243, 1133, 997, 844, 703
M.p.: 113.7-114.1℃

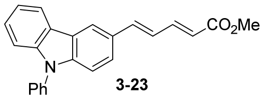

3-23

Methyl (2*E*, 4*E*)-5-(9-phenyl-9H-carbazol-3-yl)penta-2,4-dienoate (**3-23**) was obtained according to **General Procedure A** as yellow oil (32.7mg, 37% yield for **Condition A**).
^{1}H NMR (400 MHz, $CDCl_3$) δ 8.23 (s, 1H), 8.16 (d, *J* = 7.7 Hz, 1H), 7.61 (d, *J* = 7.7 Hz, 2H), 7.59-7.50 (m, 4H), 7.52-7.48 (m, 1H), 7.44-7.40 (m, 2H), 7.37-7.30 (m, 2H), 7.12 (d, *J* = 15.5 Hz, 1H), 6.95 (dd, *J* = 15.4, 11.0 Hz, 1H), 6.00 (d, *J* = 15.2 Hz, 1H), 3.79 (s, 3H).
^{13}C NMR (101 MHz, $CDCl_3$) δ 167.8, 145.6, 141.7, 141.4, 141.4, 137.2, 129.9, 128.2, 127.7, 127.0, 126.4, 125.3, 123.9, 123.2, 120.4, 120.4, 119.7, 119.1, 110.1, 110.1, 51.5
HRMS m/z (ESI): Calcd for $C_{24}H_{20}NO_2^+$ ([M + H]$^+$) 354.1489, found 354.1496
IR (KBr, cm^{-1}): 3432, 1712, 1596, 1502, 1233, 1134, 1002, 860

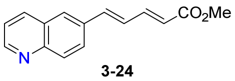

3-24

Methyl (2*E*, 4*E*)-5-(quinolin-6-yl)penta-2,4-dienoate (**3-24**) was obtained according to **General Procedure A** as white solid (42.2 mg, 71% yield for **Condition A**).
^{1}H NMR (400 MHz, $CDCl_3$) δ 8.91 (d, *J* = 6.0 Hz, 1H), 8.16-8.08 (m, 2H), 7.78 (d, *J* = 8.5 Hz, 1H), 7.69 (d, *J* = 10.3 Hz, 1H), 7.50 (dd, *J* = 15.3, 9.8 Hz, 1H), 7.38 (dd, *J* = 8.2, 4.2 Hz, 1H), 7.13-7.00 (m, 2H), 6.06 (d, *J* = 15.3 Hz, 1H), 3.78 (s, 3H)
^{13}C NMR (101 MHz, $CDCl_3$) δ 167.3, 151.0, 148.4, 144.3, 139.7, 137.1, 135.7, 128.8, 128.5, 128.3, 127.9, 124.3, 121.8, 121.4, 51.6, 29.7
HRMS m/z (ESI): Calcd for $C_{15}H_{14}NO_2^+$ ([M + H]$^+$) 240.1019, found 240.1023

IR (KBr, cm^{-1}): 2948, 1697, 1625, 1598, 1508, 1506, 1462, 1127, 1005

M.p.: 126.2-127.4℃

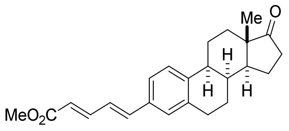

3-25

Methyl (2*E*, 4*E*)-5-((8*S*, 9*S*, 13*S*, 14*S*)-13-methyl-17-oxo-7,8,9,11,12,13,14,15,16,17-decahydro-6*H*-cyclopenta[a]phenanthren-3-yl)penta-2,4-dienoate (**3-25**) was obtained according to **General Procedure A** as white solid (25 mg, 38% yield for **Condition A**).

^{1}H NMR (400 MHz, $CDCl_3$) δ 7.44 (ddd, *J* = 15.3, 6.4, 3.9 Hz, 1H), 7.31-7.27 (m, 2H), 7.20 (s, 1H), 6.85 (s, 1H), 6.84 (d, *J* = 2.7 Hz, 1H), 5.97 (d, *J* = 15.3 Hz, 1H), 3.77 (s, 3H), 2.93 (dd, *J* = 9.0, 4.3 Hz, 2H), 2.51 (dd, *J* = 18.7, 8.7 Hz, 1H), 2.46-2.40 (m, 1H), 2.34-2.26 (m, 1H), 2.21-2.12 (m, 1H), 2.08-1.94 (m, 3H), 1.68-1.61 (m, 1H), 1.55-1.41 (m, 5H), 0.91 (s, 3H)

^{13}C NMR (101 MHz, $CDCl_3$) δ 167.6, 145.1, 141.2, 140.5, 137.0, 133.6, 127.9, 125.8, 125.6, 124.6, 120.3, 51.5, 50.5, 47.9, 44.6, 38.1, 35.8, 31.6, 29.7, 29.3, 26.4, 25.7, 21.6, 13.8

HRMS m/z (ESI) Calcd for $C_{24}H_{29}O_3^+$ ($[M + H]^+$) 365.2111, found 365.2117

IR (KBr, cm^{-1}): 2925, 2853, 1738, 1622, 1231, 1131, 998, 818

M.p.: 167.4-168.3℃

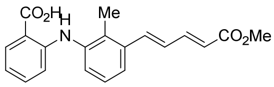

3-26

2-((3-((1*E*, 3*E*)-5-methoxy-5-oxopenta-1,3-dien-1-yl)-2-methylphenyl)amino)benzoic acid (**3-26**) was obtained according to **General Procedure A** as white solid (30.7 mg, 37%% yield for **Condition A**).

^{1}H NMR (400 MHz, DMSO-d_6) δ 9.50 (s, 1H), 7.89 (dd, *J* = 7.9, 1.6 Hz, 1H), 7.57-7.51 (m, 1H), 7.51-7.42 (m, 2H), 7.35-7.23 (m, 3H), 7.04 (dd, *J* = 15.2, 11.1 Hz, 1H), 6.74-6.68 (m, 2H), 6.13 (d, *J* = 15.3 Hz, 1H), 3.69 (s, 3H), 2.23 (s, 3H)

^{13}C NMR (101 MHz, $CDCl_3$) δ 170.2, 167.7, 148.4, 145.3, 139.3, 138.6, 136.5, 134.2, 131.8, 131.5, 128.1, 126.6, 124.9, 122.4, 120.7, 116.6, 113.2, 111.6, 51.4, 13.5

HRMS m/z (ESI) Calcd for $C_{20}H_{20}NO_4^+$ ($[M + H]^+$) 338.1387, found 338.1381

IR (KBr, cm^{-1}): 2922, 1711, 1667, 1486, 1265, 1251, 1140, 750

M.p.: 206.8-207.4℃

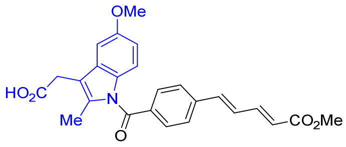

3-27

2-(5-methoxy-1-(4-((1*E*, 3*E*)-5-methoxy-5-oxopenta-1,3-dien-1-yl)benzoyl)-2-methyl-1*H*-indol-3-yl)acetic acid (**3-27**) was obtained according to **General Procedure A** as yellow solid (62.6 mg, 58% yield for **Condition A**).

^{1}H NMR (400 MHz, DMSO-d_6) δ 7.73 (d, *J* = 8.2 Hz, 2H), 7.65 (d, J = 8.3 Hz, 2H), 7.45 (dd, *J* = 15.1, 10.6

Hz, 1H), 7.30 (dd, J = 15.5, 10.6 Hz, 1H), 7.20 (d, J = 15.5 Hz, 1H), 7.02 (d, J = 2.6 Hz, 1H), 6.89 (d, J = 9.0 Hz, 1H), 6.68 (dd, J = 9.0, 2.6 Hz, 1H), 6.18 (d, J = 15.1 Hz, 1H), 3.74 (s, 3H), 3.69 (s, 3H), 3.65 (s, 2H), 2.22 (s, 3H)

^{13}C NMR (101 MHz, DMSO-d_6) δ 172.1, 166.5, 155.5, 144.4, 140.1, 139.1, 135.2, 135.1, 130.7, 130.3, 129.9, 129.2, 127.5, 122.2, 114.5, 113.2, 111.2, 101.7, 55.4, 51.5, 29.6, 13.1

HRMS m/z (ESI) Calcd for $C_{25}H_{24}NO_6^+$ ([M + H]$^+$) 434.1598, found 434.1606

IR (KBr, cm^{-1}): 2921, 1710, 1673, 1624, 1459, 1350, 1242, 1142

M.p.: 177.9-178.3℃

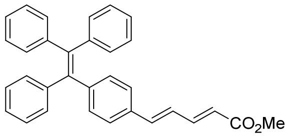

3-28

Methyl (2*E*, 4*E*)-5-(4-(1,2,2-triphenylvinyl)phenyl)penta-2,4-dienoate (**3-28**) was obtained according to **General Procedure A** as green solid (59.8 mg, 53% yield for **Condition A**).

^{1}H NMR (400 MHz, $CDCl_3$) δ 7.42 (ddd, J = 15.3, 6.9, 3.5 Hz, 1H), 7.22-7.18 (m, 2H), 7.14-7.09 (m, 9H), 7.08-6.98 (m, 8H), 6.81 (s, 1H), 6.79 (d, J = 3.4 Hz, 1H), 5.96 (d, J = 15.2 Hz, 1H), 3.77 (s, 3H)

^{13}C NMR (101 MHz, $CDCl_3$) δ 167.5, 144.9, 144.8, 143.5, 143.4, 141.6, 140.3, 140.2，133.9, 131.8, 131.33, 131.27, 127.8, 127.7, 127.6, 126.63, 126.56, 126.54, 126.52, 125.9, 120.4, 51.5.

HRMS m/z (ESI) Calcd for $C_{32}H_{27}O_2^+$ ([M + H]$^+$) 443.2006, found 443.2001

IR (KBr, cm^{-1}): 3023, 1708, 1624, 1598, 1242, 1139, 749, 699

M.p.: 153.4-154.3℃

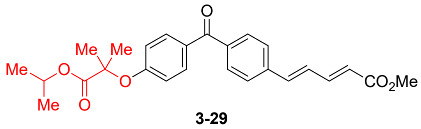

3-29

Methyl (2*E*, 4*E*)-5-(4-(4-((1-isopropoxy-2-methyl-1-oxopropan-2-yl)oxy)benzoyl)phenyl)penta-2,4-dienoate (**3-29**) was obtained according to **General Procedure A** as pale yellow solid (51.3 mg, 47% yield for **Condition A**).

^{1}H NMR (400 MHz, $CDCl_3$) δ 7.77-7.71 (m, 4H), 7.57-7.53 (m, 2H), 7.46 (ddd, J = 15.4, 8.9, 1.3 Hz, 1H), 7.00-6.93 (m, 2H), 6.88-6.83 (m, 2H), 6.06 (d, J = 15.4 Hz, 1H), 5.16-5.02 (m, 1H), 3.78 (s, 3H), 1.66 (s, 6H), 1.20 (d, J = 6.3 Hz, 6H)

^{13}C NMR (101 MHz, $CDCl_3$) δ 194.6, 173.1, 167.2, 159.6, 144.1, 139.4, 139.1, 138.1, 131.9, 130.4, 130.3, 128.3, 126.8, 122.1, 117.2, 76.3, 69.3, 51.6, 25.3, 21.5

HRMS m/z (ESI) Calcd for $C_{26}H_{29}O_6^+$ ([M + H]$^+$) 437.1959, found 437.1953

IR (KBr, cm^{-1}): 2980, 1731, 1718, 1627, 1280, 1239, 1152, 927

M.p.: 109.6-110.4℃

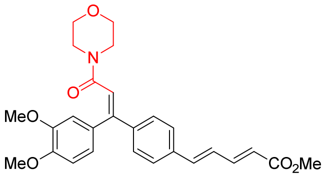

3-30

Methyl (2*E*, 4*E*)-5-(4-((Z)-1-(3,4-dimethoxyphenyl)-3-morpholino-3-oxoprop-1-en-1-yl)phenyl)penta-2,4-dienoate (**3-30**) was obtained according to **General Procedure A** as yellow oil (63 mg, 54% yield for **Condition A**).

^{1}H NMR (400 MHz, $CDCl_3$) δ 7.50-7.39 (m, 3H), 7.31-7.26 (m, 2H), 6.91-6.86 (m, 2H), 6.81 (ddd, *J* = 15.9, 6.7, 2.6 Hz, 3H), 6.25 (d, *J* = 2.9 Hz, 1H), 6.02 (dd, *J* = 15.3, 5.3 Hz, 1H), 3.90 (d, *J* = 6.7 Hz, 3H), 3.80 (d, *J* = 2.6 Hz, 3H), 3.77 (d, *J* = 2.7 Hz, 3H), 3.56- 3.48 (m, 4H), 3.29-3.24 (m, 2H), 3.19-3.11 (m, 2H)

^{13}C NMR (101 MHz, $CDCl_3$) δ 167.30, 148.7, 146.6, 144.5, 144.4, 141.3, 139.5, 139.5, 136.3, 130.7, 129.9, 128.5, 127.1, 127.0, 126.9, 126.7, 122.1, 121.3, 121.1, 119.7, 112.5, 111.0, 110.7, 110.7, 66.2, 55.89, 55.85, 55.83, 55.80, 51.5, 46.6, 41.5, 41.4

HRMS m/z (ESI) Calcd for $C_{27}H_{30}NO_6^+$ ($[M + H]^+$) 464.2068, found 464.2063

IR (KBr, cm^{-1}): 2963, 2852, 1712, 1626, 1434, 1259, 1135, 727

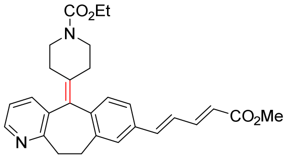

3-31

Ethyl 4-(8-((1*E*, 3*E*)-5-methoxy-5-oxopenta-1,3-dien-1-yl)-10,11-dihydro-5*H*-benzo[4,5]cyclohepta-[1,2-b]pyridin-5-ylidene)piperidine-1-carboxylate (**3-31**) was obtained according to **General Procedure A** as yellow oil (48.2 mg, 42% yield for **Condition A**).

^{1}H NMR (400 MHz, $CDCl_3$) δ 8.41 (dd, *J* = 4.8, 1.7 Hz, 1H), 7.70-7.63 (m, 1H), 7.58-7.40 (m, 3H), 7.28-7.26 (m, 1H), 7.20 (d, *J* = 7.8 Hz, 1H), 7.11 (dd, *J* = 7.7, 4.8 Hz, 1H), 6.85 (d, *J* = 4.3 Hz, 1H), 5.98 (d, *J* = 15.3 Hz, 1H), 4.14 (q, *J* = 7.0 Hz, 2H), 3.89-3.78 (m, 2H), 3.76 (s, 3H), 3.48-3.32 (m, 2H), 3.23-3.08 (m, 2H), 2.93-2.80 (m, 2H), 2.57-2.26 (m, 4H), 1.25 (t, *J* = 7.1 Hz, 3H)

^{13}C NMR (101 MHz, $CDCl_3$) δ 167.4, 155.4, 146.4, 144.7, 140.0, 137.5, 137.3, 135.1, 134.5, 132.0, 131.9, 129.8, 128.5, 128.3, 127.9, 125.9, 124.8, 122.2, 120.6, 61.2, 51.5, 44.71, 44.65, 31.7, 31.5, 30.7, 30.4, 14.6

HRMS m/z (ESI) Calcd for $C_{28}H_{31}N_2O_4^+$ ($[M + H]^+$) 459.2278, found 459.2274

IR (KBr, cm^{-1}): 2923, 1698, 1437, 1229, 1135, 1116, 998, 801

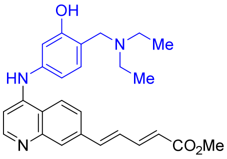

3-32

Methyl (2*E*, 4*E*)-5-(4-((3-((diethylamino)methyl)-4-hydroxyphenyl)amino)quinolin-7-yl)penta-2,4-dienoate (**3-32**) was obtained according to **General Procedure A** as brown solid (22.7 mg, 21% yield for **Condition A**).

^{1}H NMR (400 MHz, Methanol-d$_4$) δ 8.28 (d, *J* = 8.7 Hz, 2H), 7.84 (s, 1H), 7.77 (d, *J* = 8.9 Hz, 1H), 7.48 (dd, *J* = 15.2, 10.5 Hz, 1H), 7.29-7.17 (m, 4H), 7.11 (d, J = 15.6 Hz, 1H), 6.94 (d, *J* = 9.2 Hz, 1H), 6.66 (dd, *J* = 6.0, 2.7 Hz, 1H), 6.13 (d, *J* = 15.2 Hz, 1H), 4.07 (s, 1H), 3.76 (s, 3H), 3.71 (d, *J* = 6.4 Hz, 2H), 2.95 (q, *J* = 7.2 Hz, 4H), 1.26 (t, *J* = 7.3 Hz, 6H).

^{13}C NMR (101 MHz, Methanol-d$_4$) δ 168.9, 153.4, 149.1, 146.6, 145.7, 140.2, 131.6, 130.3, 128.4, 128.1, 125.6, 124.6, 123.6, 123.3, 119.8, 117.8, 101.6, 68.8, 52.2, 48.1, 10.5

HRMS m/z (ESI) Calcd for $C_{26}H_{30}N_3O_3^+$ ([M + H]$^+$) 432.2282, found 432.2286

IR (KBr, cm^{-1}): 3428, 2929, 1710, 1623, 1570, 1497, 1260, 1138

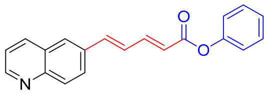

3-33

Phenyl (2*E*, 4*E*)-5-(quinolin-7-yl)penta-2,4-dienoate (**3-33**) was obtained according to **General Procedure A** as yellow solid (21.8 mg, 29% yield for **Condition A**).

^{1}H NMR (400 MHz, CDCl$_3$) δ 9.01 (dd, *J* = 4.3, 1.7 Hz, 1H), 8.25-8.18 (m, 2H), 7.90 (d, *J* = 8.5 Hz, 1H), 7.84-7.73 (m, 2H), 7.51-7.46 (m, 3H), 7.36-7.32 (m, 1H), 7.27-7.19 (m, 4H), 6.34 (d, *J* = 15.2 Hz, 1H)

^{13}C NMR (101 MHz, CDCl$_3$) δ 165.2, 151.1, 150.7, 148.3, 146.0, 140.7, 137.0, 135.7, 129.4, 129.1, 128.7, 128.3, 127.7, 125.7, 124.3, 121.57, 121.54, 121.42

HRMS m/z (ESI) Calcd for $C_{20}H_{16}NO_2^+$([M + H]$^+$) 302.1176, found 302.1172

IR (KBr, cm^{-1}): 3448, 2928, 1726, 1625, 1198, 1121, 839, 704

M.p.:155.4-156.3℃

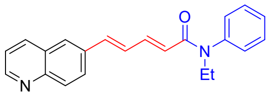

3-34

(2*E*, 4*E*)-*N*-ethyl-*N*-phenyl-5-(quinolin-7-yl)penta-2,4-dienamide (**3-34**) was obtained according to **General Procedure A** as brown oil (53.1 mg, 65% yield for **Condition A**)

^{1}H NMR (400 MHz, CDCl$_3$) δ 8.86 (dd, *J* = 4.3, 1.7 Hz, 1H), 8.10-8.02 (m, 1H), 8.02-7.98 (m, 1H), 7.70 (d, *J* = 8.5 Hz, 1H), 7.58 (dd, *J* = 8.6, 1.7 Hz, 1H), 7.52-7.48 (m, 1H), 7.46-7.41 (m, 2H), 7.40-7.37 (m, 1H), 7.33 (dd, *J* = 8.2, 4.3 Hz, 1H), 7.21-7.16 (m, 2H), 6.99 (d, *J* = 15.6 Hz, 1H), 6.84 (dd, *J* = 15.6, 10.9 Hz, 1H), 5.90 (d, *J* = 14.8 Hz, 1H), 3.86 (q, *J* = 7.1 Hz, 2H), 1.16 (t, *J* = 7.1 Hz, 3H)

^{13}C NMR (101 MHz, CDCl$_3$) δ 165.4, 150.8, 148.3, 141.9, 141.2, 137.9, 137.5, 135.6, 129.5, 128.7, 128.4, 128.20, 128.18, 128.0, 127.7, 124.3, 123.5, 121.1, 44.3, 13.0

HRMS m/z (ESI) Calcd for $C_{22}H_{21}N_2O^+$ ([M + H]$^+$) 329.1648, found 329.1643

IR (KBr, cm^{-1}): 2973, 1647, 1593, 1392, 1272, 1127, 998, 789

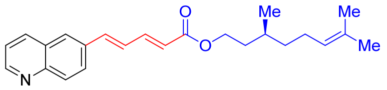

3-35

(*S*)-3,7-dimethyloct-6-en-1-yl (2*E*, 4*E*)-5-(quinolin-7-yl)penta-2,4-dienoate (**3-35**) was obtained according to **General Procedure A** as brown oil (64.7 mg, 71% yield for **Condition A**).

^{1}H NMR (400 MHz, CDCl$_3$) δ 8.90 (dd, *J* = 4.3, 1.7 Hz, 1H), 8.09 (dd, *J* = 10.8, 1.9 Hz, 2H), 7.77 (d, *J* = 8.5 Hz, 1H), 7.68 (dd, *J* = 8.5, 1.7 Hz, 1H), 7.48 (dd, *J* = 15.3, 9.6 Hz, 1H), 7.36 (dd, *J* = 8.2, 4.2 Hz, 1H),

7.12-6.96 (m, 2H), 6.05 (d, *J* = 15.3 Hz, 1H), 5.14-5.05 (m, 1H), 4.26-4.15 (m, 2H), 2.06-1.90 (m, 2H), 1.79-1.70 (m, 1H), 1.68 (s, 3H), 1.60 (s, 3H), 1.58-1.44 (m, 2H), 1.42-1.30 (m, 1H), 1.25-1.18 (m, 1H), 0.93 (d, *J* = 6.5 Hz, 3H)

^{13}C NMR (101 MHz, $CDCl_3$) δ 166.9, 151.0, 1484, 143.9, 139.5, 137.1, 135.6, 131.3, 128.8, 128.5, 128.2, 128.0, 124.5, 124.3, 122.4, 121.4, 63.0, 36.9, 35.4, 29.5, 25.7, 25.3, 19.4, 17.6

HRMS m/z (ESI) Calcd for $C_{24}H_{30}NO_2^+$ ([M + H]$^+$) 364.2271, found 324.2267

IR (KBr, cm^{-1}): 2961, 2925, 1710, 1623, 1570, 1497, 1260, 1138

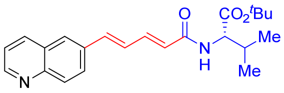

3-36

***Tert*-butyl ((2*E*, 4*E*)-5-(quinolin-7-yl)penta-2,4-dienoyl)-*L*-valinate** (**3-36**) was obtained according to **General Procedure A** as brown oil (34.9 mg, 37% yield for **Condition A**).

^{1}H NMR (400 MHz, $CDCl_3$) δ 8.89 (dd, *J* = 4.3, 1.7 Hz, 1H), 8.11-8.06 (m, 2H), 7.75 (d, *J* = 8.5 Hz, 1H), 7.65 (dd, *J* = 8.6, 1.7 Hz, 1H), 7.47-7.41 (m, 1H), 7.36 (dd, *J* = 8.2, 4.3 Hz, 1H), 7.01 (d, *J* = 6.5 Hz, 2H), 6.33 (d, *J* = 8.8 Hz, 1H), 6.12 (d, *J* = 14.9 Hz, 1H), 4.60 (dd, *J* = 8.8, 4.6 Hz, 1H), 2.2-2.16 (m, 1H), 1.47 (s, 9H), 0.95 (dd, *J* = 12.4, 6.9 Hz, 6H)

^{13}C NMR (101 MHz, $CDCl_3$) δ 171.4, 165.6, 150.8, 148.2, 140.8, 138.4, 137.5, 135.8, 128.3, 128.2, 128.1, 128.0, 124.8, 124.5, 121.2, 82.1, 57.4, 31.7, 28.0, 18.9, 17.7

HRMS m/z (ESI) Calcd for $C_{23}H_{29}N_2O_3^+$ ([M + H]$^+$) 381.2173, found 381.2167

IR (KBr, cm^{-1}): 3314, 2969, 1730, 1621, 1532, 1389, 1148, 999

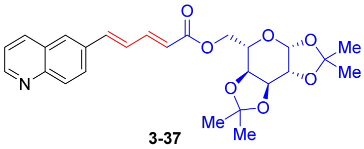

3-37

((3*aS*, 5*R*, 5*aR*, 8*aR*, 8*bS*)-2,2,7,7-tetramethyltetrahydro-5*H*-bis([1,3]dioxolo)[4,5-b:4',5'-d]pyran-5-yl)methyl (2*E*, 4*E*)-5-(quinolin-7-yl)penta-2,4-dienoate (**3-37**) was obtained according to **General Procedure A** as yellow oil (67.8 mg, 73% yield for **Condition A**).

^{1}H NMR (400 MHz, $CDCl_3$) δ 8.89 (dd, *J* = 4.3, 1.7 Hz, 1H), 8.12-8.05 (m, 2H), 7.76 (d, *J* = 8.5 Hz, 1H), 7.67 (dd, *J* = 8.5, 1.7 Hz, 1H), 7.49 (dd, *J* = 15.3, 10.0 Hz, 1H), 7.36 (dd, J = 8.2, 4.2 Hz, 1H), 7.10-6.97 (m, 2H), 6.09 (d, *J* = 15.3 Hz, 1H), 5.55 (d, *J* = 5.0 Hz, 1H), 4.62 (dd, *J* = 7.9, 2.5 Hz, 1H), 4.42-4.38 (m, 1H), 4.32 (dd, *J* = 5.0, 2.5 Hz, 1H), 4.31-4.25 (m, 2H), 4.13-4.04 (m, 1H), 1.52 (s, 3H), 1.45 (s, 3H), 1.33 (d, J = 7.2 Hz, 6H)

^{13}C NMR (101 MHz, $CDCl_3$) δ 166.6, 150.9, 148.3, 144.5, 139.8, 137.1, 135.8, 128.7, 128.5, 128.2, 127.9, 124.3, 121.9, 121.4, 109.6, 108.8, 96.3, 71.0, 70.7, 70.4, 66.0, 63.4, 26.0, 25.9, 24.9, 24.5

HRMS m/z (ESI) Calcd for $C_{26}H_{30}NO_7^+$ ([M + H]$^+$) 468.2017, found 468.2020

IR (KBr, cm^{-1}): 2988, 1712, 1626, 1255, 1211, 1069, 1003, 896

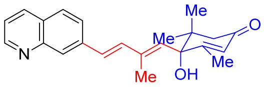

3-39

(*R*)-4-hydroxy-3, 5, 5-trimethyl-4-((1*E*, 3*E*)-2-methyl-4-(quinolin-7-yl)buta-1,3-dien-1-yl)cyclohex-2-en-1-one (**3-39**) was obtained according to **General Procedure A** as brown oil (E/Z = 5:1, 20.6 mg, 25%

yield for **Condition A**).

^{1}H NMR (400 MHz, $CDCl_3$) δ 8.70 (dd, *J* = 4.2, 1.6 Hz, 1H), 7.99 (d, *J* = 7.2 Hz, 1H), 7.86 (s, 1H), 7.60 (d, J = 8.6 Hz, 1H), 7.28-7.19 (m, 2H), 6.92 (d, *J* = 15.7 Hz, 1H), 6.51 (s, 1H), 5.86 (d, *J* = 15.8 Hz, 1H), 5.70 (s, 1H), 2.34 (d, *J* = 17.0 Hz, 1H), 2.14 (d, *J* = 17.1 Hz, 1H), 1.93 (d, J = 1.1 Hz, 3H), 1.77 (d, *J* = 1.2 Hz, 3H), 0.93 (s, 3H), 0.91 (s, 3H)

^{13}C NMR (101 MHz, $CDCl_3$) δ 198.1, 150.2, 147.6, 138.7, 136.2, 135.1, 132.3, 129.6, 128.7, 128.5, 127.5, 127.1, 126.8, 126.5, 120.9, 79.6, 49.8, 41.5, 24.4, 23.1, 21.6, 19.2

HRMS m/z (ESI) Calcd for $C_{23}H_{26}NO_2$+ ([M + H]$^+$) 348.1958, found 348.1958

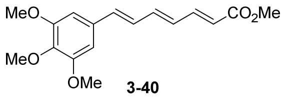

3-40

Methyl (2*E*, 4*E*, 6*E*)-7-(3,4,5-trimethoxyphenyl)hepta-2,4,6-trienoate (**3-40**) was obtained according to **General Procedure A** as yellow oil (21.3mg, 28% yield for **Condition A**).

^{1}H NMR (400 MHz, $CDCl_3$) δ 7.37 (dd, *J* = 15.3, 11.3 Hz, 1H), 6.76 (dd, *J* = 15.8, 5.3 Hz, 1H), 6.66 (d, *J* = 6.8 Hz, 2H), 6.44 (dd, *J* = 14.2, 11.3 Hz, 1H), 5.91 (d, *J* = 15.2 Hz, 1H), 3.89 (s, 6H), 3.86 (s, 3H), 3.76 (s, 3H)

^{13}C NMR (101 MHz, $CDCl_3$) δ 167.5, 153.4, 144.6, 140.7, 136.6, 132.2, 130.0, 127.4, 120.3, 105.5, 103.9, 60.9, 56.1, 51.5

HRMS (ESI, m/z): Calcd for $C_{17}H_{21}O_5^+$ ([M + H]$^+$) 305.1384, found 305.1387

IR (KBr, cm^{-1}): 2943, 1729, 1589, 1508, 1462, 1239, 1127, 1005

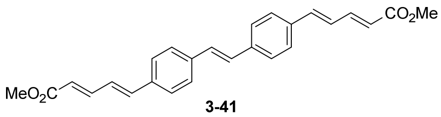

3-41

Dimethyl 5,5'-(((*E*)-ethene-1,2-diyl)bis(4,1-phenylene))(2*E*,2'*E*,4*E*,4'*E*)-bis(penta-2,4-dienoate) (**3-41**) was obtained according to **General Procedure A** as yellow solid (13 mg, 22% yield, **Condition A**).

^{1}H NMR (400 MHz, $CDCl_3$) δ 7.52-7.43 (m, 10H), 7.13 (s, 2H), 6.90 (d, *J* = 5.7 Hz, 4H), 6.01 (d, *J* = 15.2 Hz, 2H), 3.78 (s, 6H)

^{13}C NMR (101 MHz, $CDCl_3$) δ 167.5, 144.8, 140.0, 137.9, 135.5, 128.7, 127.6, 127.0, 126.2, 120.8, 51.6

HRMS m/z (ESI): Calcd for $C_{26}H_{25}O_4^+$ ([M + H]$^+$) 401.1747, found 401.1757

IR (KBr, cm^{-1}): 3434, 2925, 1720, 1625, 1246, 1172, 1029, 826

M.p.: 260.3-261.2℃

5-((1*E*,3*E*)-4-phenylbuta-1,3-dien-1-yl)benzofuran (5-3) was prepared according to following procedure as shown in Supplementary Fig. 32.

To the solution of compound **3-6** (17.6 mg, 0.094 mmol, 1.0 equivalent) in THF/H_2O (1 mL/0.5mL) was added LiOH (12mg, 0.5mmol, 5.3 equivalent). Next, the reaction was stirred at room temperature until no starting material **3-6** left, followed by being quenched by adding HCl (1M). The reaction mixture was extracted by EtOAc, washed by H_2O and brine, dried by Na_2SO_4. Compound **3-38** (16 mg, 98% yield) was obtained by filtration and concentration as a white solid.

Compound **5-3** was prepared from compound **3-38** (65 mg, 0.375 mmol, 1.5 equivalent) and 5-bromobenzofuran (**2-21**, 49 mg, 0.25 mmol, 1.0 equivalent), and obtained as white solid (25.8 mg, 42% yield, **General Procedure A**, **Condition A**).

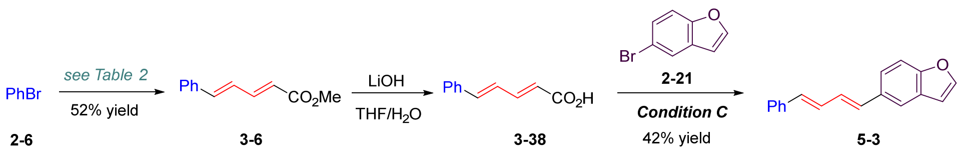

Supplementary Fig. 32 Preparation of compound **5-3** *via* cascade Heck-decarboxylic coupling

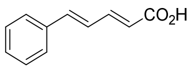

3-38

(2*E*, 4*E*)-5-phenylpenta-2,4-dienoic acid (**3-38**)

H NMR (400 MHz, $CDCl_3$) δ 7.55 (dd, *J* = 15.2, 9.7 Hz, 1H), 7.51-7.45 (m, 2H), 7.41-7.32 (m, 3H), 7.00-6.86 (m, 2H), 6.01 (d, *J* = 15.2 Hz, 1H)

^{13}C NMR (101 MHz, $CDCl_3$) δ 172.4, 147.0, 141.6, 135.8, 129.3, 128.8, 127.3, 125.9, 120.3

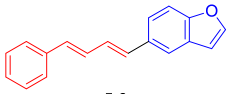

5-3

5-((1*E*, 3*E*)-4-phenylbuta-1,3-dien-1-yl)benzofuran (5-3)

^{1}H NMR (400 MHz, $CDCl_3$) δ 7.65 (d, *J* = 1.6 Hz, 1H), 7.62 (d, *J* = 2.2 Hz, 1H), 7.49-7.41 (m, 4H), 7.34 (t, *J* = 7.7 Hz, 2H), 7.23 (t, *J* = 7.5 Hz, 1H), 7.03-6.90 (m, 2H), 6.82-6.74 (m, 2H), 6.67 (d, *J* = 14.7 Hz, 1H)

^{13}C NMR (101 MHz, $CDCl_3$) δ 154.7, 145.5, 137.5, 133.1, 132.5, 132.1, 129.4, 128.6, 128.3, 127.9, 127.4, 126.3, 122.9, 119.1, 111.5, 106.7

HRMS m/z (ESI) Calcd for $C_{18}H_{15}O^+$ ($[M + H]^+$) 247.1117, found 247.1115

IR (KBr, cm^{-1}): 3435, 2968, 1666, 1464, 1264, 1122, 994, 751

M.p.: 106.3-107.5℃

Di-*tert*-butyl (((1*E*, 3*E*)-buta-1,3-diene-1,4-diyl)bis(4,1-phenylene))bis(ethylcarbamate) (**5-4**) was prepared from starting materials **1-3** (64 mg, 0.45 mmol, 3.0 equivalent) and **2-1** (45 mg, 0.15 mmol, 1.0 equivalent) in the presence of Pd-XPhos-G2 (12 mg, 10% mmol) and CsOAc (87 mg, 0.45 mmol, 3.0 equivalent) in 4.0 mL DMF (**General Procedure A**, **condition A**) as shown in following Supplementary Fig. 33, and obtained as brown oil (22.7 mg, 37% yield)

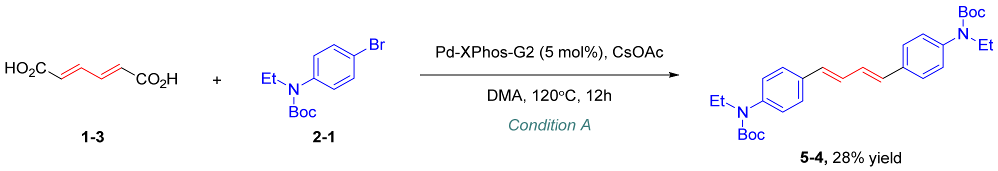

Supplementary Fig. 33 Preparation of compound **5-4** *via* dual coupling

Di-*tert*-butyl (((1*E*, 3*E*)-buta-1,3-diene-1,4-diyl)bis(4,1-phenylene))bis(ethylcarbamate) (**5-1**)

^{1}H NMR (400 MHz, $CDCl_3$) δ 7.40-7.38 (m, 4H), 7.15 (d, *J* = 8.1 Hz, 4H), 6.93-6.89 (m, 2H), 6.66-6.62 (m, 2H), 3.68 (q, *J* = 7.1 Hz, 4H), 1.45 (s, 18H), 1.15 (t, *J* = 7.1 Hz, 6H)

^{13}C NMR (101 MHz, $CDCl_3$) δ 154.4, 141.7, 134.9, 132.1, 129.1, 127.0, 126.6, 80.1, 44.8, 28.4, 13.9

HRMS m/z (ESI) Calcd for $C_{30}H_{41}N_2O_4^+$ ($[M + H]^+$) 493.3061, found 493.3065

IR (KBr, cm^{-1}): 3424, 2976, 1700, 1391, 1281, 1152, 997, 857

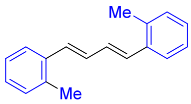

5-5

(1*E*, 3*E*)-1,4-di-o-tolylbuta-1,3-diene[14] (**5-5**) was obtained in the same procedure as product **5-4** as a brown solid (15.8 mg, 23% yield, 0.07 mmol scale).

^{1}H NMR (400 MHz, $CDCl_3$) δ 7.57 (d, *J* = 7.4 Hz, 1H), 7.22-7.15 (m, 3H), 6.92 (d, *J* = 2.8 Hz, 2H), 2.40 (s, 3H).

^{13}C NMR (101 MHz, $CDCl_3$) δ136.69, 136.11, 131.20, 130.99, 130.78, 127.96, 126.65, 125.52, 20.41

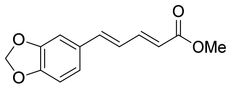

3-42

Methyl (2*E*, 4*E*)-5-(benzo[d][1,3]dioxol-5-yl)penta-2,4-dienoate （**3-42**） was obtained according to **General Procedure A** as yellow solid (32 mg, 55% yield, **General Procedure A, Condition A**)

^{1}H NMR (400 MHz, $CDCl_3$) δ 7.42 (dd, *J* = 15.2, 10.8 Hz, 1H), 6.99 (d, *J* = 1.7 Hz, 1H), 6.91 (dd, *J* = 8.0, 1.6 Hz, 1H), 6.83-6.78 (m, 2H), 6.70 (dd, *J* = 15.5, 10.8 Hz, 1H), 5.98 (s, 2H), 5.95 (d, *J* = 15.3 Hz, 1H), 3.76 (s, 3H)

^{13}C NMR (101 MHz, $CDCl_3$) δ 167.6, 148.6, 148.3, 145.0, 140.3, 130.5, 124.5, 122.9, 119.9, 108.5, 105.9, 101.4, 51.5

HRMS m/z (ESI) calcd for $C_{14}H_{16}O_3$ $[M + Na]^+$: 255.0628, found 255.0628

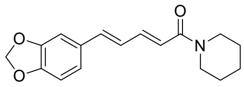

5-6, piperine

(2*E*, 4*E*)-5-(benzo[d][1,3]dioxol-5-yl)-1-(piperidin-1-yl)penta-2,4-dien-1-one[15] (**5-6**, **piperine**) was prepared according to **Scheme S6** as white solid (40.5 mg, 47% yield, 0.4 mmol scale)

^{1}H NMR (400 MHz, $CDCl_3$) δ 7.40 (ddd, *J* = 14.7, 8.5, 1.7 Hz, 1H), 6.97 (d, *J* = 1.7 Hz, 1H), 6.88 (dd, *J* = 8.0, 1.7 Hz, 1H), 6.75 (dd, *J* = 14.6, 8.4 Hz, 3H), 6.43 (d, *J* = 14.7 Hz, 1H), 5.97 (s, 2H), 3.57 (d, *J* = 43.6 Hz, 4H), 1.71-1.63 (m, 2H), 1.62-1.51 (m, 4H)

^{13}C NMR (101 MHz, $CDCl_3$) δ 165.4, 148.13, 148.06, 142.5, 138.2, 131.0, 125.3, 122.5, 119.9, 108.4, 105.6, 101.2, 46.9, 43.2, 26.7, 25.6, 24.6

Methyl (2*E*, 4*E*)-5-(3-(pyridin-3-yl)phenyl)penta-2,4-dienoate (**3-44**) was prepared according to following procedure as shown in Supplementary Fig. 34.

Preparation of 3-(hetero)arylbromobenzne **2-30** was achieved according to literature. To the dry 25 mL Schlenk tube was added 1,3-dibromobenzene (**2-29**, 702 mg, 3.0 mmol, 1.5 equivalents), pyridin-3-ylboronic acid (**6-1**, 246 mg, 2.0 mmol, 1.0 equivalent), Pd(dppf)Cl_2 (59 mg, 0.08 mmol, 4 mol%) and K_3PO_4 (2.12 g, 10 mmol, 5.0 equivalents) was added THF (8 mL) and H_2O (10 mL) under nitrogen balloon. Next, the reaction mixture was warmed to 70℃ and continued stirring for 1h, followed by being quenched by H_2O. The mixture was then extracted by EtOAc, washed by H_2O and brine, dried by Na_2SO_4. The

desired product **2-29** was obtained as colorless oil (112 mg, 24% yield) after filtration, concentration and flash chromatography.

Compound **3-44** was prepared according to **General Procedure A** (**Condition A**) from compound **1-2** and **2-30** and obtained as brown oil (48.1 mg, 73% yield).

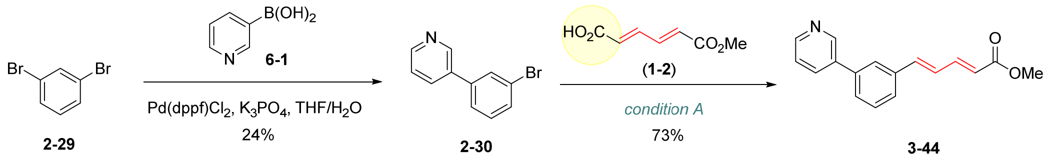

Supplementary Fig. 34 Preparation 3-arylbenzyldiene compounds *via* cascade Suzuki-Heck-decarboxylic couplings procedure

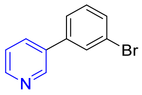

2-30

3-(3-bromophenyl)pyridine (2-30)

^{1}H NMR (400 MHz, $CDCl_3$) δ 8.80 (dd, *J* = 2.4, 0.8 Hz, 1H), 8.61 (dd, *J* = 4.9, 1.6 Hz, 1H), 7.83 (ddd, *J* = 8.0, 2.4, 1.7 Hz, 1H), 7.71 (t, *J* = 1.9 Hz, 1H), 7.54-7.47 (m, 2H), 7.39-7.31 (m, 2H)

^{13}C NMR (101 MHz, $CDCl_3$) δ 149.0, 148.1, 139.9, 135.2, 134.3, 131.0, 130.5, 130.1, 125.7, 123.6, 123.1

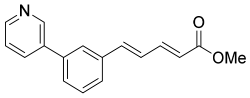

3-44

Methyl (2*E*, 4*E*)-5-(3-(pyridin-3-yl)phenyl)penta-2,4-dienoate (**3-44**)

^{1}H NMR (400 MHz, $CDCl_3$) δ 8.85-8.84 (m, 1H), 8.61 (dd, *J* = 4.8, 1.7 Hz, 1H), 7.89 (dt, *J* = 8.0, 2.0 Hz, 1H), 7.63 (d, *J* = 1.9 Hz, 1H), 7.55-7.46 (m, 2H), 7.48-7.43 (m, 2H), 7.38 (ddd, *J* = 7.9, 4.9, 0.9 Hz, 1H), 6.98-6.92 (m, 2H), 6.03 (d, *J* = 15.3 Hz, 1H), 3.77 (s, 3H)

^{13}C NMR (101 MHz, $CDCl_3$) δ 167.3, 148.5, 148.0, 144.4, 139.8, 138.4, 136.8, 136.2, 134.5, 129.5, 127.7, 126.9, 126.7, 125.9, 123.6, 121.4, 51.6

HRMS m/z (ESI) Calcd for $C_{17}H_{16}NO_2^+$ ([M + H]$^+$) 266.1176, found 266.1180

IR (KBr, cm^{-1}): 2925, 1715, 1627, 1435, 1241, 1137, 1000, 789

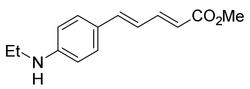

Methyl (2*E*, 4*E*)-5-(4-(ethylamino)phenyl)penta-2,4-dienoate (**3-45**) was obtained according to **Supplementary Fig. 10** as yellow solid (58 mg, 98% yield).

^{1}H NMR (400 MHz, $CDCl_3$) δ 7.44 (dd, *J* = 15.2, 11.0 Hz, 1H), 7.30 (d, *J* = 8.5 Hz, 2H), 6.80 (d, *J* = 15.4 Hz, 1H), 6.67 (dd, *J* = 15.4, 11.0 Hz, 1H), 6.55 (d, *J* = 8.4 Hz, 2H), 5.87 (d, *J* = 15.2 Hz, 1H), 3.86 (br, 1H), 3.75 (s, 3H), 3.18 (q, *J* = 7.2 Hz, 2H), 1.26 (t, *J* = 7.2 Hz, 3H)

^{13}C NMR (101 MHz, $CDCl_3$) δ 167.9, 149.2, 146.0, 141.4, 128.8, 125.0, 121.6, 117.7, 112.5, 51.4, 38.1, 14.7

HRMS m/z (ESI) Calcd for $C_{14}H_{18}NO_2^+$ ([M + H]$^+$) 232.1332, found 232.1332

IR (KBr, cm^{-1}): 3377, 2967, 2926, 1667, 1587, 1312, 1133, 1004, 821

M.p.:129.9-130.8℃

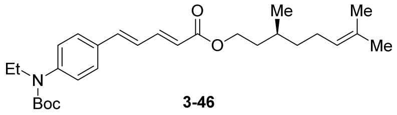

3-46

(*S*)-3,7-dimethyloct-6-en-1-yl (2E,4E)-5-(4-((tert-butoxycarbonyl)(ethyl)amino)phenyl)penta-2,4-dienoate (**3-46**) was obtained according to **General Procedure A** as yellow oil (47.6 mg, 42% yield, **Condition A**)

^{1}H NMR (400 MHz, $CDCl_3$) δ 7.31-7.20 (m, 3H), 7.07-6.97 (m, 2H), 6.78-6.62 (m, 2H), 5.81 (d, *J* = 15.3 Hz, 1H), 4.98-4.88 (m, 1H), 4.09-3.97 (m, 2H), 3.52 (q, *J* = 7.1 Hz, 2H), 1.91-1.74 (m, 2H), 1.61-1.53 (m, 1H), 1.51 (s, 3H), 1.44 (s, 3H), 1.42-1.31 (m, 2H), 1.27 (s, 9H), 1.22-1.16 (m, 1H), 1.09-1.00 (m, 1H), 0.98 (t, *J* = 7.1 Hz, 3H), 0.77 (d, *J* = 6.5 Hz, 3H)

^{13}C NMR (101 MHz, $CDCl_3$) δ 167.1, 144.5, 139.6, 131.3, 129.6, 127.4, 126.9, 126.0, 124.6, 121.2, 80.3, 62.9, 44.8, 37.0, 35.5, 29.5, 28.3, 25.7, 25.4, 19.4, 17.7, 13.9

HRMS m/z (ESI) Calcd for $C_{28}H_{42}NO_4^+$ ($[M + H]^+$) 456.3108, found 456.3113

IR (KBr, cm^{-1}): 2969, 2928, 1703, 1601, 1389, 1283, 1152, 993

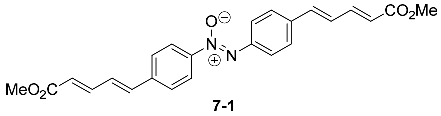

7-1

(*Z*)-1, 2-bis(4-((1*E*, 3*E*)-5-methoxy-5-oxopenta-1,3-dien-1-yl)phenyl)diazene 1-oxide (**7-1**) was obtained according to Supplementary Fig. 10 as yellow solid (37 mg, 71% yield).

^{1}H NMR (400 MHz, $CDCl_3$) δ 8.33-8.29 (m, 2H), 8.23 (d, *J* = 8.7 Hz, 2H), 7.62-7.54 (m, 4H), 7.50-7.43 (m, 2H), 7.02-6.90 (m, 4H), 6.07 (dd, *J* = 15.3, 11.9 Hz, 2H), 3.79 (d, *J* = 2.5 Hz, 6H)

^{13}C NMR (101 MHz, CDCl3) δ 167.3, 167.2, 148.0, 144.4, 144.3, 143.9, 139.4, 139.3, 138.4, 137.4, 128.8, 127.7, 127.5, 127.4, 126.3, 122.9, 122.6, 121.8, 51.7, 51.6

HRMS m/z (ESI) calcd for $C_{24}H_{23}N_2O_5^+$ ($[M + H]^+$) 419.1601, found 419.1600.

IR (KBr, cm^{-1}) 3441, 1708, 1621, 1454, 1247, 1137, 994, 855

M.p.: 215.4-216.3℃

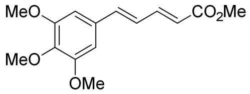

3-47

Methyl (2*E*, 4*E*)-5-(3,4,5-trimethoxyphenyl)penta-2,4-dienoate (**3-47**) was obtained according to **General Procedure A** as yellow oil (32.9 mg, 47% yield for **Condition A**).

^{1}H NMR (400 MHz, $CDCl_3$) δ 7.47-7.39 (m, 1H), 6.85-6.74 (m, 2H), 6.68 (s, 2H), 5.99 (d, *J* = 15.2 Hz, 1H), 3.89 (s, 6H), 3.86 (s, 3H), 3.76 (s, 3H)

^{13}C NMR (101 MHz, $CDCl_3$) δ 167.5, 153.4, 144.7, 140.4, 139.1, 131.6, 125.6, 120.5, 104.3, 60.9, 56.1, 51.5

HRMS (ESI, m/z): Calcd for $C_{15}H_{19}O_5^+$ ($[M + H]^+$) 279.1227, found 279.1232

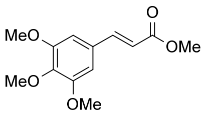

8-1

Methyl (*E*)-3-(3, 4, 5-trimethoxyphenyl)acrylate (**8-1**) was obtained according to **General Procedure A** as yellow solid (11.5 mg, 18% yield, **Condition A**).

^{1}H NMR (400 MHz, $CDCl_3$) δ 7.61 (d, *J* = 15.9 Hz, 1H), 6.75 (s, 2H), 6.35 (d, *J* = 15.9 Hz, 1H), 3.88 (d, *J* = 3.5 Hz, 9H), 3.81 (s, 3H)

^{13}C NMR (101 MHz, $CDCl_3$) δ 167.4, 153.4, 144.8, 140.1, 129.9, 117.0, 105.2, 61.0, 56.1, 51.7

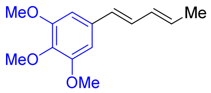

5-7

1, 2, 3-trimethoxy-5-((1*E*, 3*E*)-penta-1,3-dien-1-yl)benzene (**5-7**) was obtained according to **General Procedure A** as colorless oil (30% yield by NMR, **condition C,** isolated 39.5 mg, 17% yield by Al_2O_3 column).

^{1}H NMR (400 MHz, $CDCl_3$) δ 6.68-6.61 (m, 1H), 6.60 (s, 2H), 6.35 (d, *J* = 15.6 Hz, 1H), 6.25-6.16 (m, 1H), 5.84 (dd, *J* = 14.8, 7.1 Hz, 1H), 3.88 (s, 6H), 3.84 (s, 3H), 1.83 (d, *J* = 8.4 Hz, 2H).

^{13}C NMR (101 MHz, $CDCl_3$) δ 153.3, 137.4, 133.4, 131.6, 130.3, 129.6, 128.9, 103.0, 60.9, 56.0, 18.4

HRMS m/z (ESI) Calcd for $C_{14}H_{19}O_3^+$ ([M + H]$^+$) 235.1329, found 235.1326

IR (KBr, cm^{-1}): 2927, 1579, 1508, 1454, 1242, 1128, 1008, 988

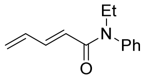

9-2

(***E***)**-N-ethyl-N-phenylpenta-2,4-dienamide** (**9-2**) was isolated as byproduct during preparation of product 3-34 from substrate **1-11** and **2-24** according to **General procedure A** (**Condition A**), isolate as xx solid (6.5 mg, 9% yield based on **1-11**)

^{1}H NMR (400 MHz, $CDCl_3$) δ 7.42 (t, J = 7.4 Hz, 2H), 7.35 (t, *J* = 7.3 Hz, 1H), 7.30-7.24 (m, 1H), 7.18-7.12 (m, 2H), 6.23 (dt, *J* = 16.9, 10.6 Hz, 1H), 5.74 (d, *J* = 15.0 Hz, 1H), 5.51 (d, *J* = 16.9 Hz, 1H), 5.32 (d, J = 10.0 Hz, 1H), 3.83 (q, *J* = 7.2 Hz, 2H), 1.14 (t, *J* = 7.1 Hz, 3H)

Supplementary reference

1. Zhou, L-H., Yu, X-Q. & Pu, L. Reactivity of a propiolate dimer with nucleophiles and an efficient synthesis of dimethyl α-aminoadipate. *Tetra. Lett.* **51**, 425-427 (2010).
2. Ke, L., Zhu, G., Qian, H., Xiang, G., Chen, Q. & Chen, Z. Catalytic selective oxidative coupling of secondary *N*-alkylanilines: an approach to azoxyarene. *Org. Lett.* **21**, 4008-4013 (2019)
3. Myers, A. G., Tanaka, D. & Mannion, M. R. Development of a Decarboxylative Palladation Reaction and Its Use in a Heck-type Olefination of Arene Carboxylates. *J. Am. Chem. Soc.* **124**, 11250-11251 (2002).
4. Goossen, L. J., Rodríguez, N., Melzer, B., Linder, C., Deng, G. & Levy, L. M. Biaryl Synthesis via Pd-Catalyzed Decarboxylative Coupling of Aromatic Carboxylates with Aryl Halides. *J. Am. Chem. Soc.* **129**, 4824-4833 (2007)
5. Wang, Z., Ding, Q., He, X. & Wu, J. Palladium-catalyzed decarboxylative cross-coupling reaction of cinnamic acid with aryl iodide. *Org. Bio. Chem.* 7, 863-865 (2009).
6. Heck, R. F. *Palldium-catalyzed vinylation of organic halides, Organic Reactions* (Hoboken, NJ, United States, 27, 1982)
7. Solabannavar, S. B., Helavi, V. B., Desai, U. V. & Mane, R. B. Application of Amberlite IRA-400 (Basic) as a Base in Heck Reaction. *Syn. Comm.* **33**, 361-365 (2003)
8. Yamashita, M., Hirano, K., Satoh, T. & Miura, M. Synthesis of 1,4-Diarylbuta-1,3-dienes through Palladium- Catalyzed Decarboxylative Coupling of Unsaturated Carboxylic Acids. *Adv. Syn. Cat.* **353**, 631-636 (2011).
9. Mitsudo, T., Fischetti, W. & Heck, R. F. Palladium-catalyzed syntheses of aryl polyenes. *J. Org. Chem.* **49**, 1640-1646 (1984)
10. Al-Huniti, M. H., Perez, M. A., Garr, M. K. & Croatt, M. P. Palladium-Catalyzed Chemoselective Protodecarboxylation of Polyenoic Acids. *Org. Lett.* **20**, 7375-7379 (2018)
11. Elvidge, J. A., Linstead, R. P., Sims, P. & Orkin, B. A. The third isomeric (*cis-trans-*) muconic acid. *J. Chem. Soc. (Resumed).*, 2235-2241 (1950)
12. Lekkala, R., Lekkala, R., Moku, B. & Qin, H-L. SO_2F_2 mediated dehydrative cross-coupling of alcohols with electron-deficient olefins in DMSO using a Pd-catalyst: one-pot transformation of alcohols into 1,3-dienes. *Org. Chem. Front.*, **6**, 796-800 (2019)
13. Schabel, T. & Plietker, B. Microwave-Accelerated Ru-Catalyzed Hydrovinylation of Alkynes and Enynes: A Straightforward Approach toward 1,3-Dienes and 1,3,5-Trienes. *Chem. Eur. J.* **19**, 6938-6941 (2013)
14. Wilklow-Marnell, M., Bo Li, B., Zhou, T., Krogh-Jespersen, K., Brennessel, W. W., Emge, T, J., Goldman, A. S., Orcid & Jones, W. D., catalytic dehydrogenative C–C coupling by a pincer-ligated iridium complex, *J. Am. Chem. Soc.*, **139**, 26, 8977-8989 (2017)
15. Christopher J. Teskey, Pauline Adler, Carlos R. Gonçalves & Nuno Maulide, *Angew. Chem. Int. Ed.* **58**, 447-451 (2019)

X. NMR spectrum of products and intermediates

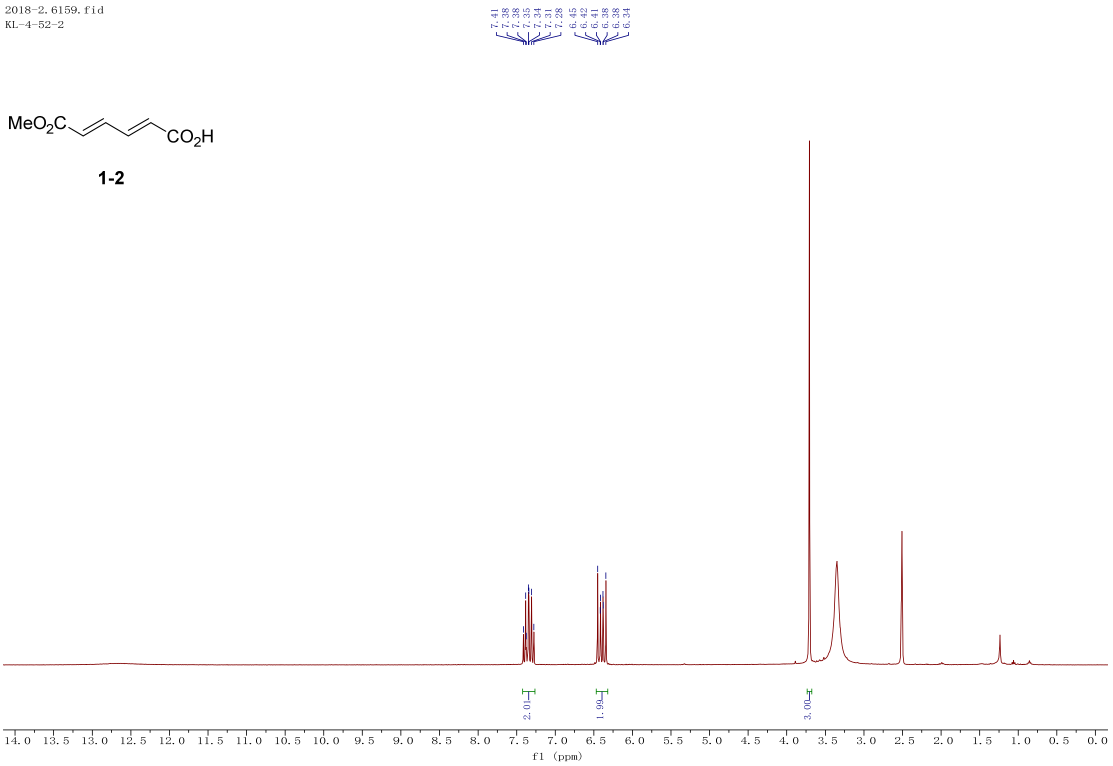

Supplementary Fig. 35

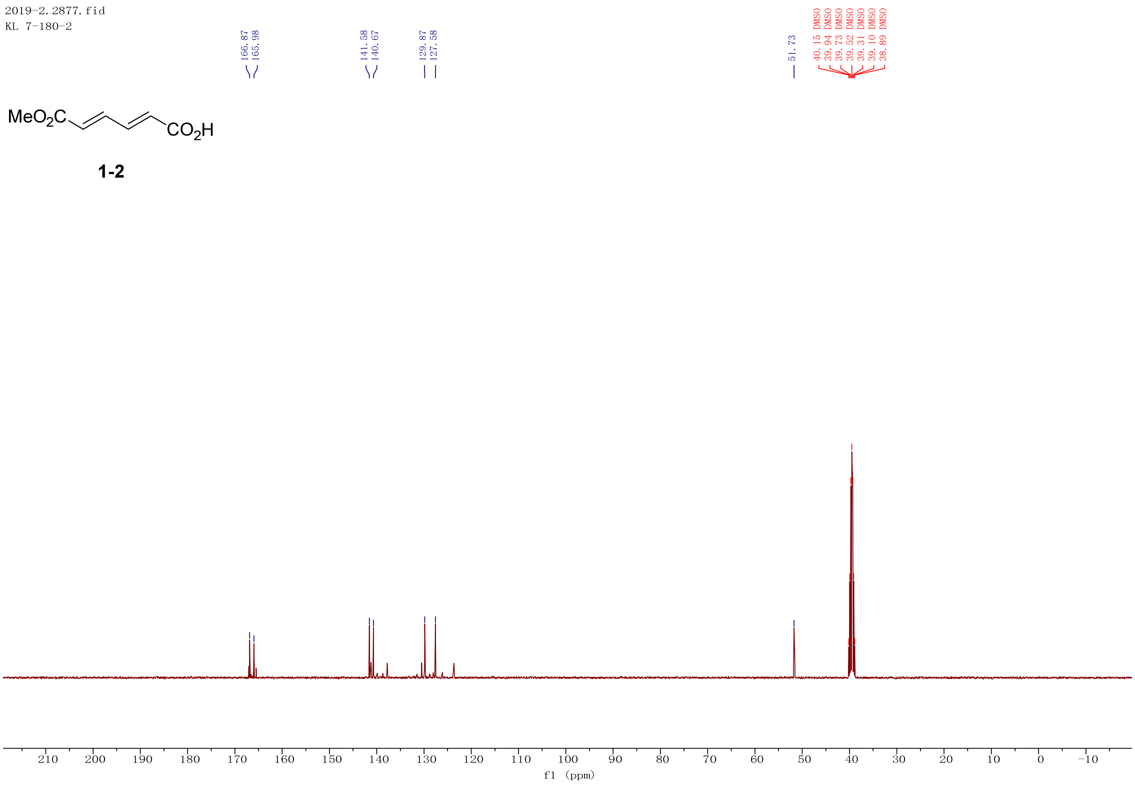

Supplementary Fig. 36

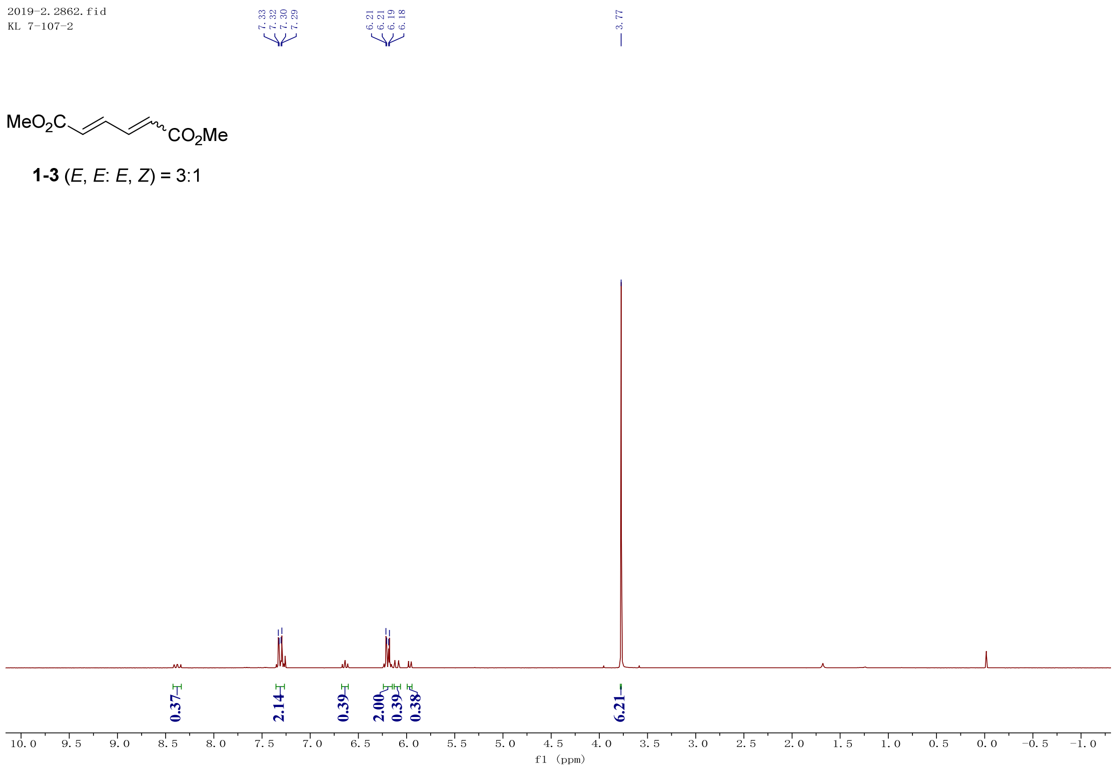

Supplementary Fig. 37

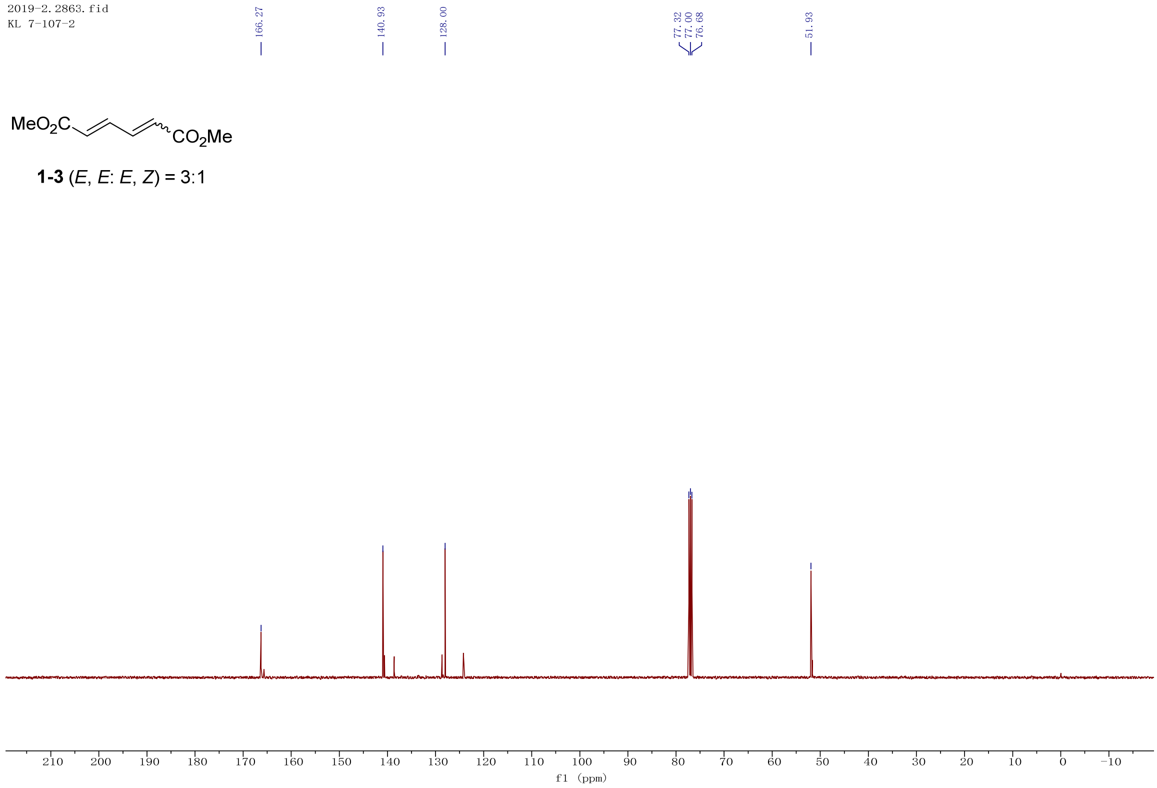

Supplementary Fig. 38

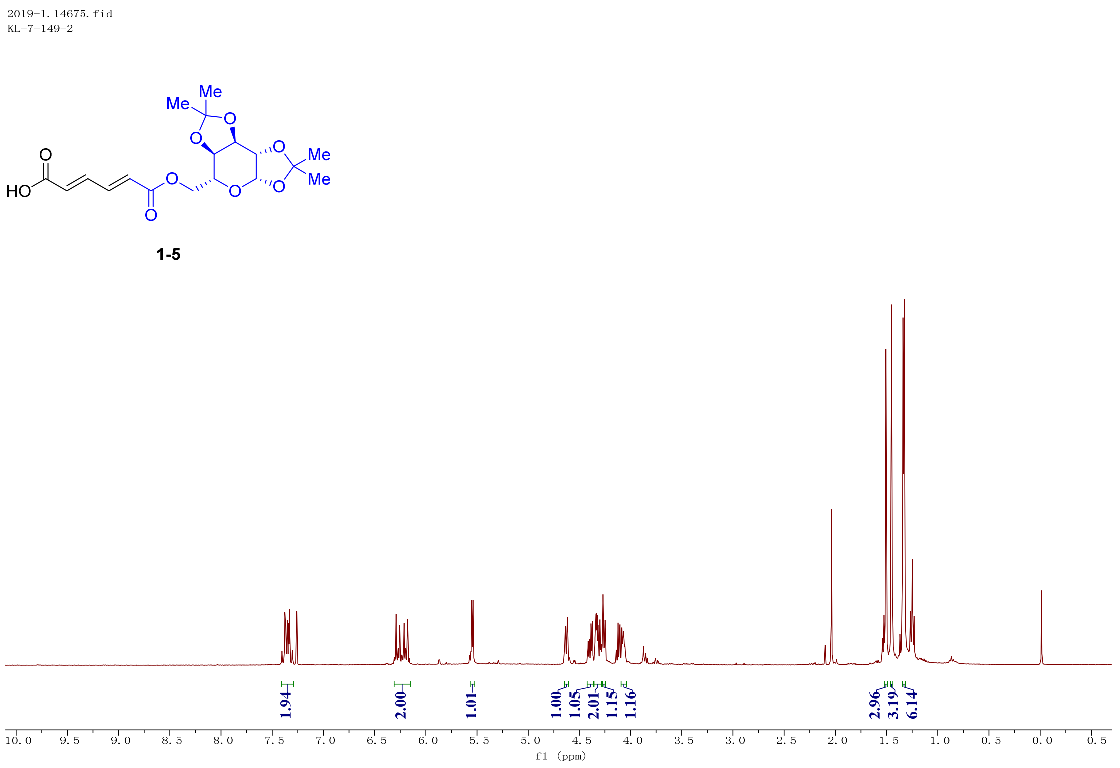

Supplementary Fig. 39

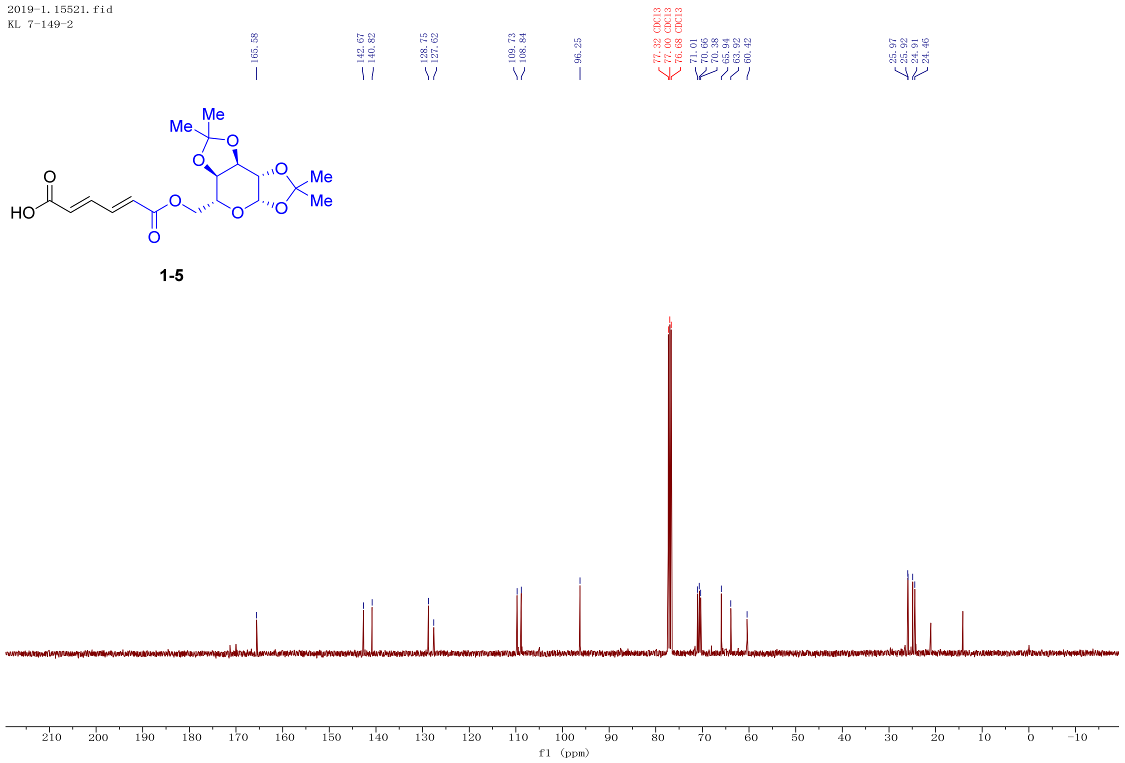

Supplementary Fig. 40

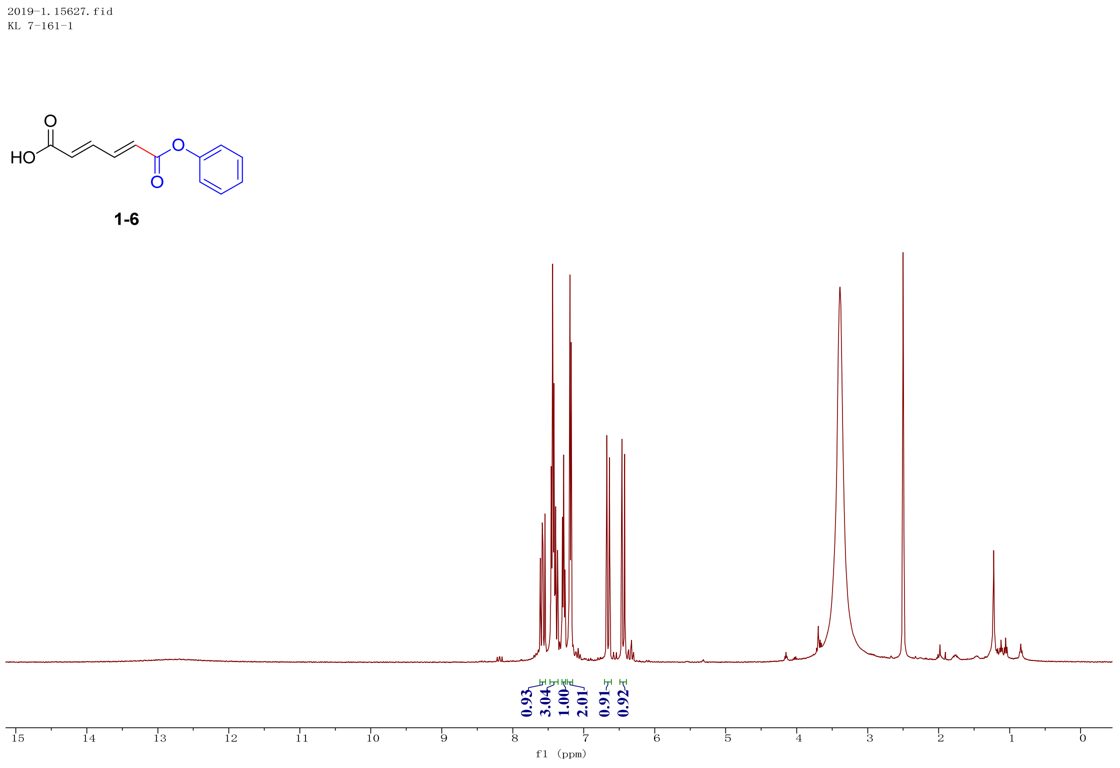

Supplementary Fig. 41

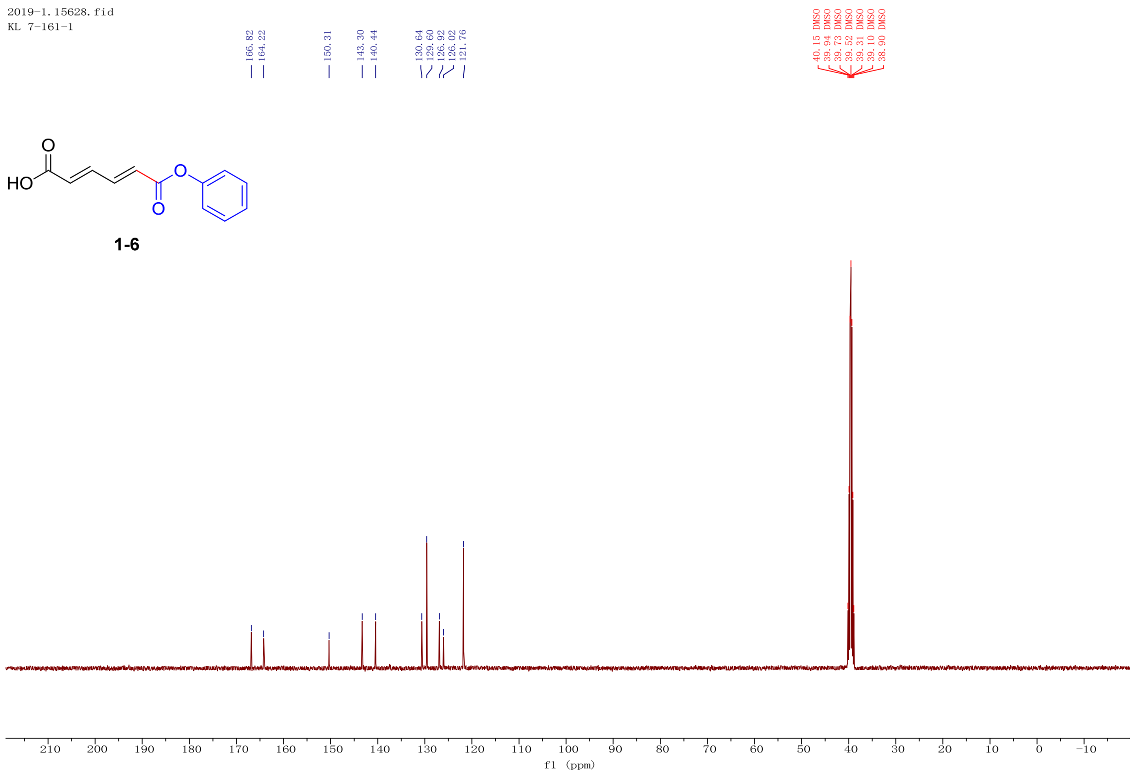

Supplementary Fig. 42

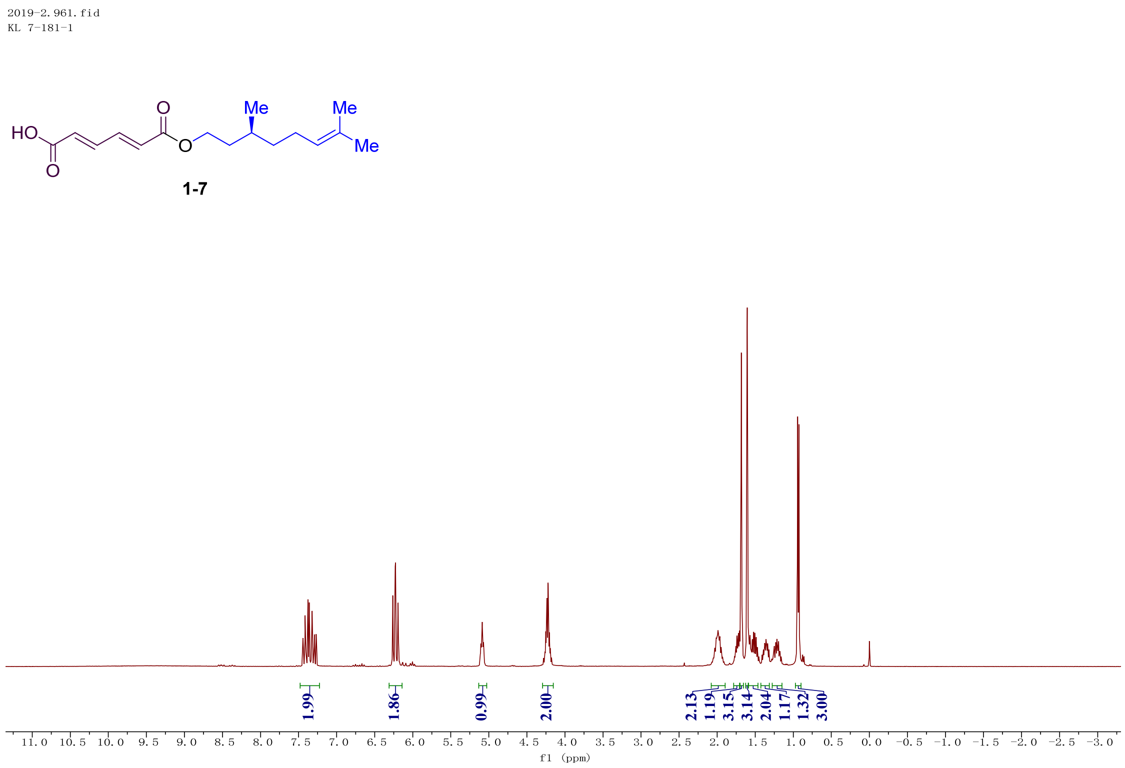

Supplementary Fig. 43

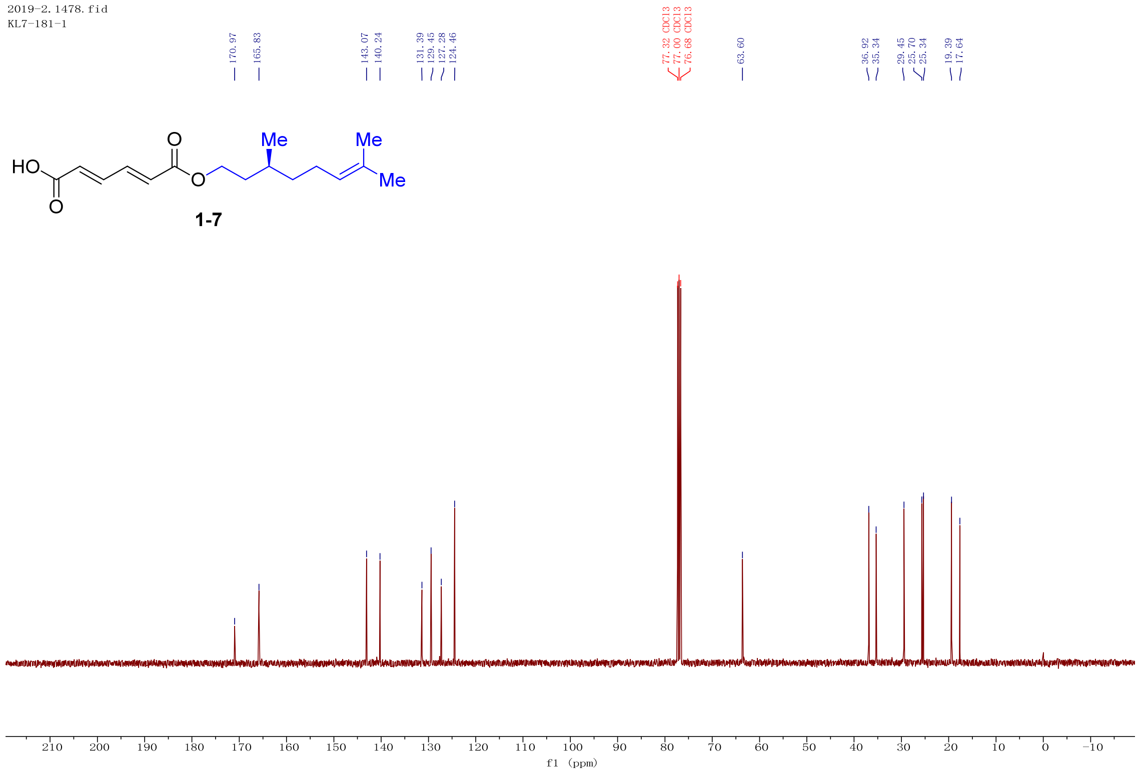

Supplementary Fig. 44

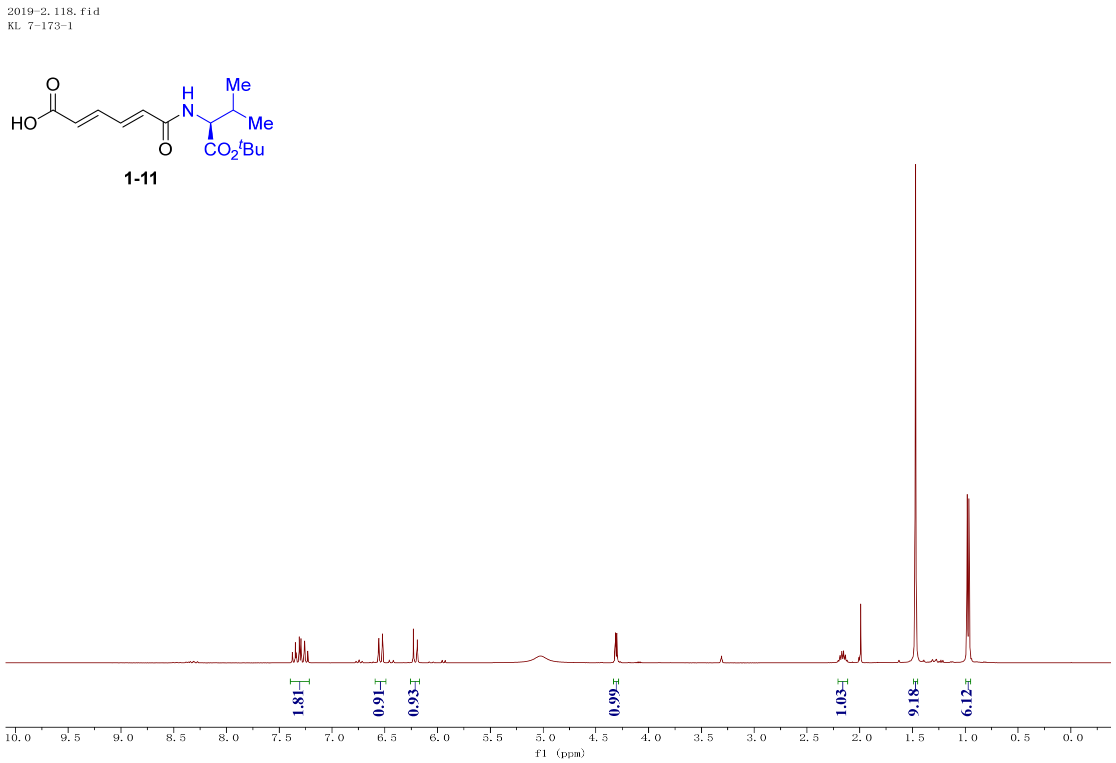

Supplementary Fig. 45

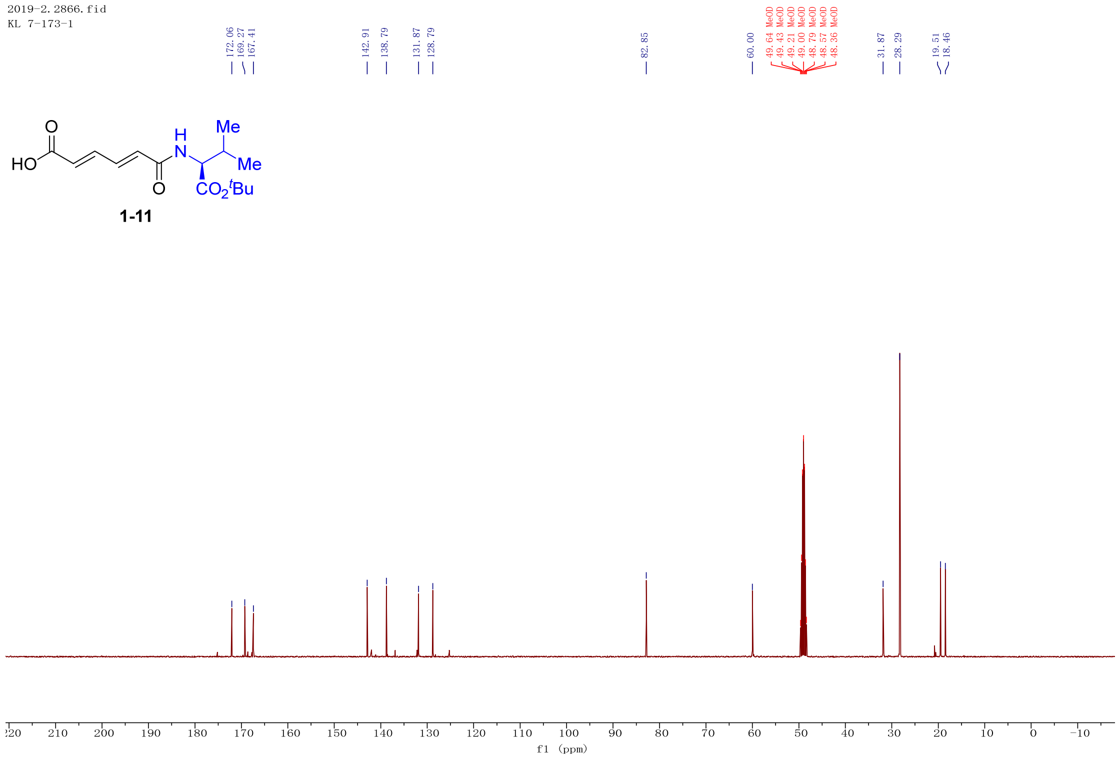

Supplementary Fig. 46

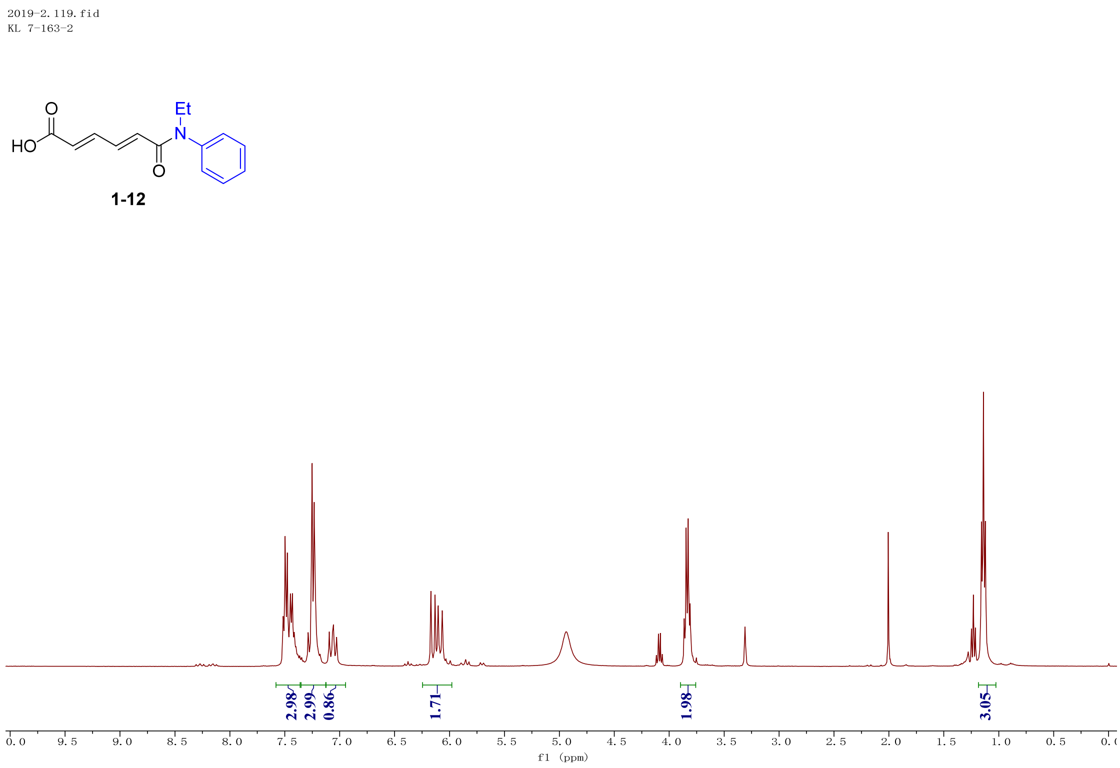

Supplementary Fig. 47

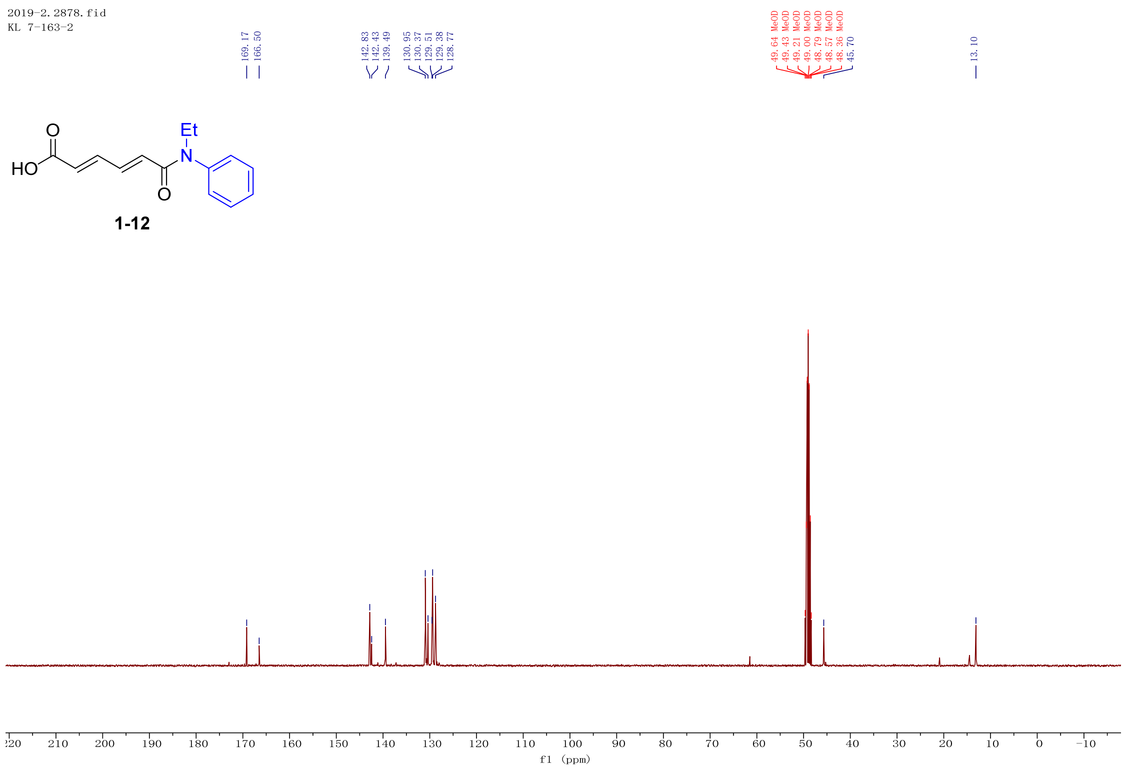

Supplementary Fig. 48

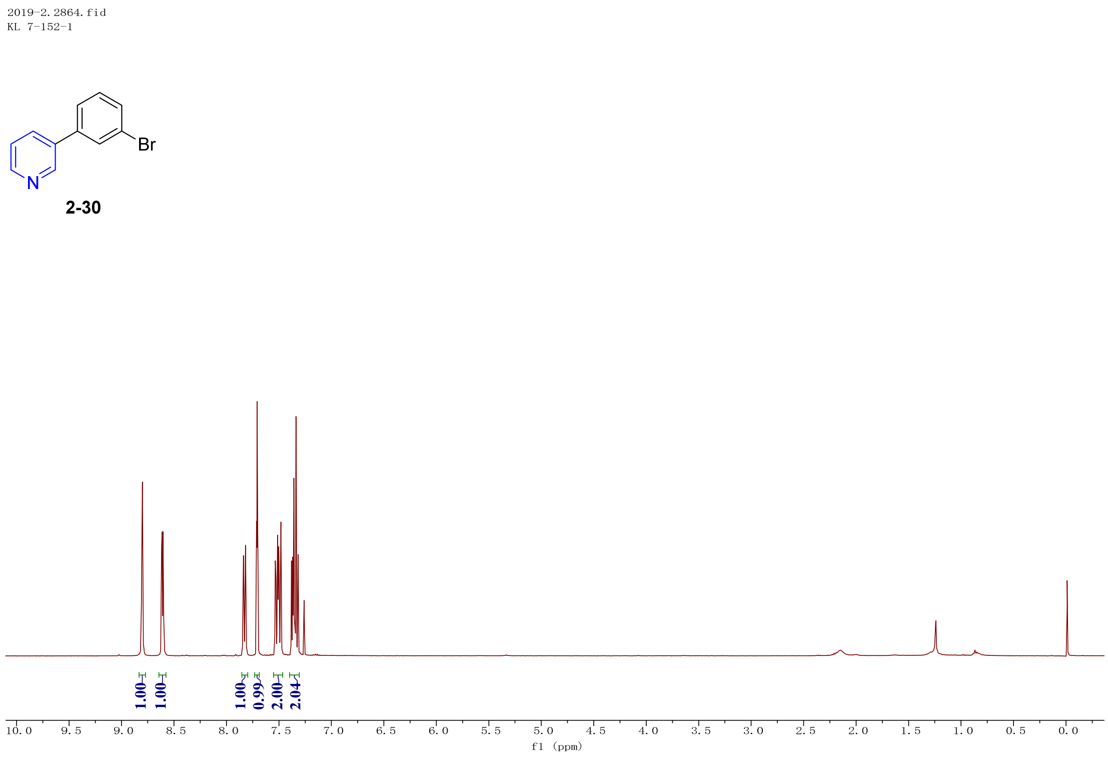

Supplementary Fig. 49

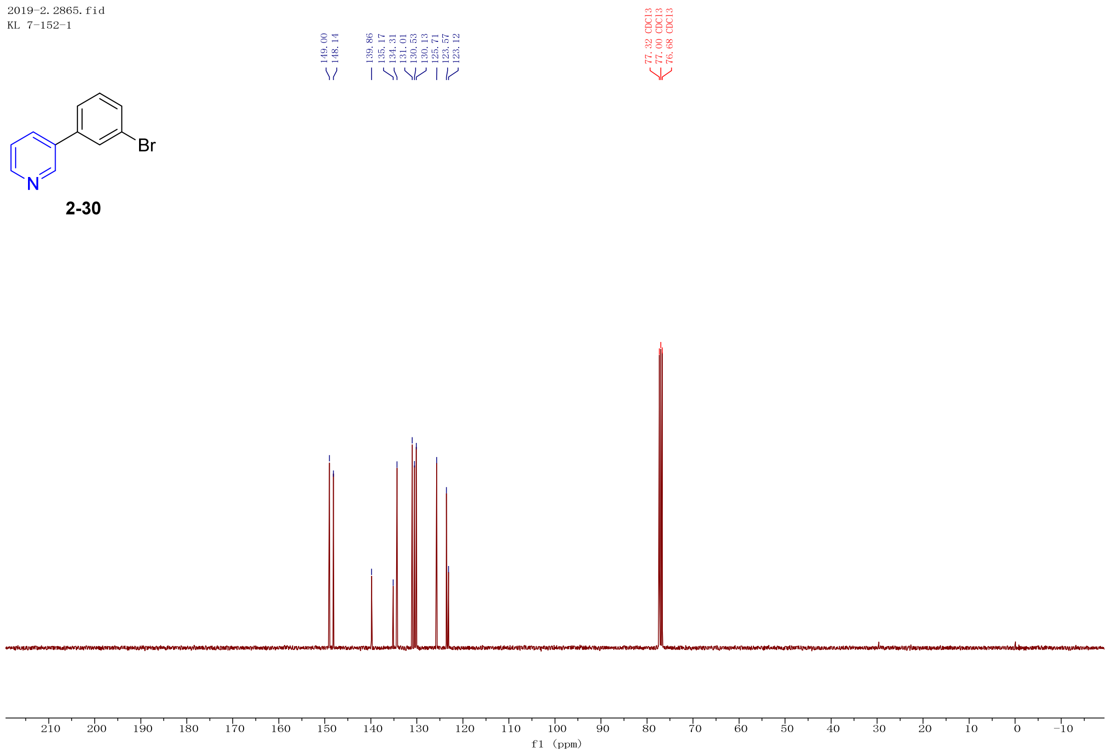

Supplementary Fig. 50

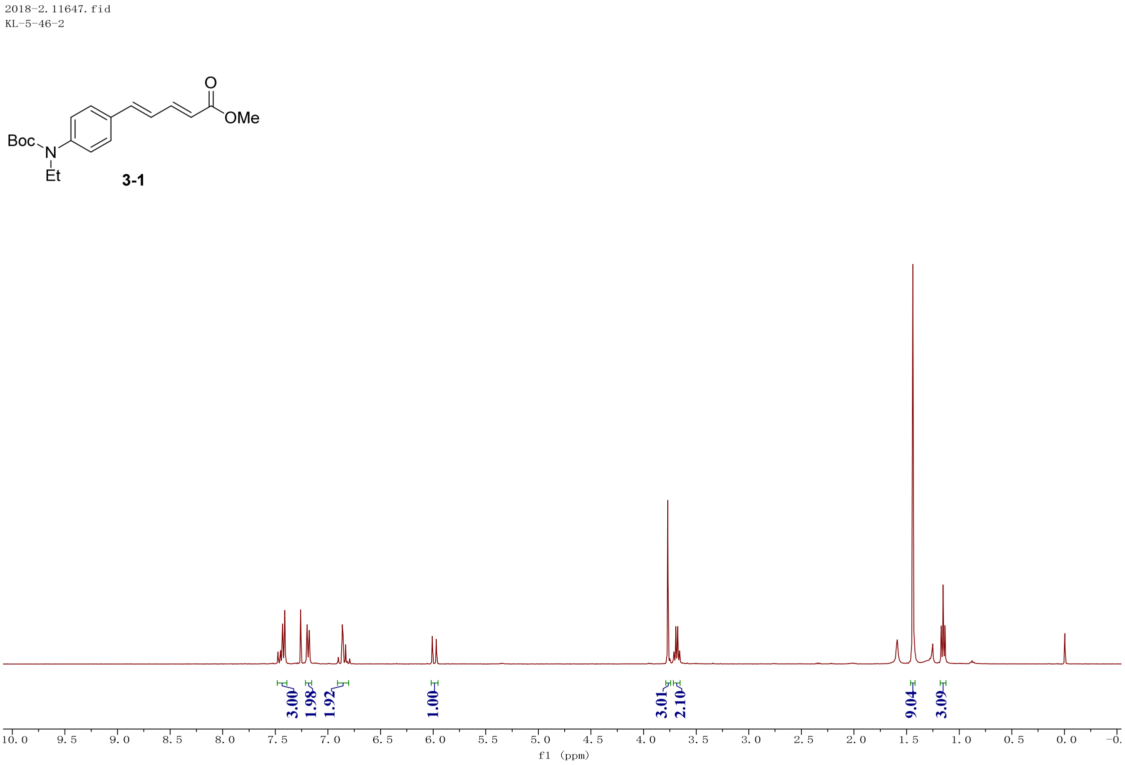

Supplementary Fig. 51

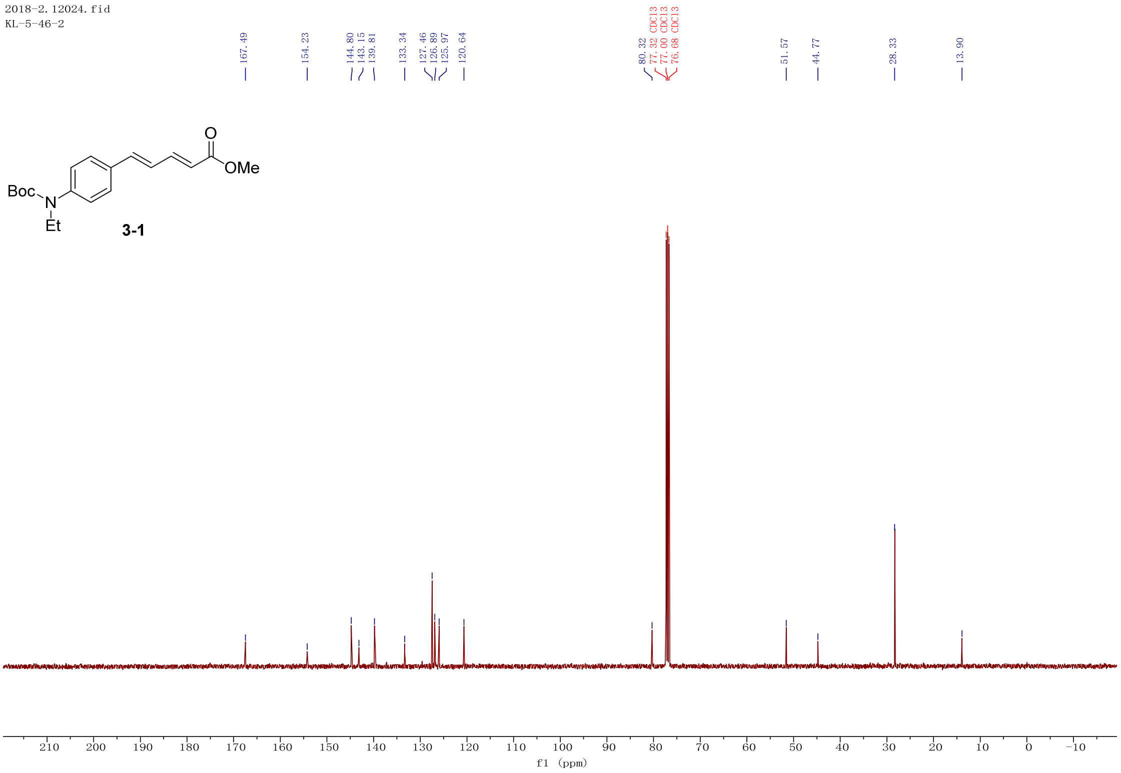

Supplementary Fig. 52

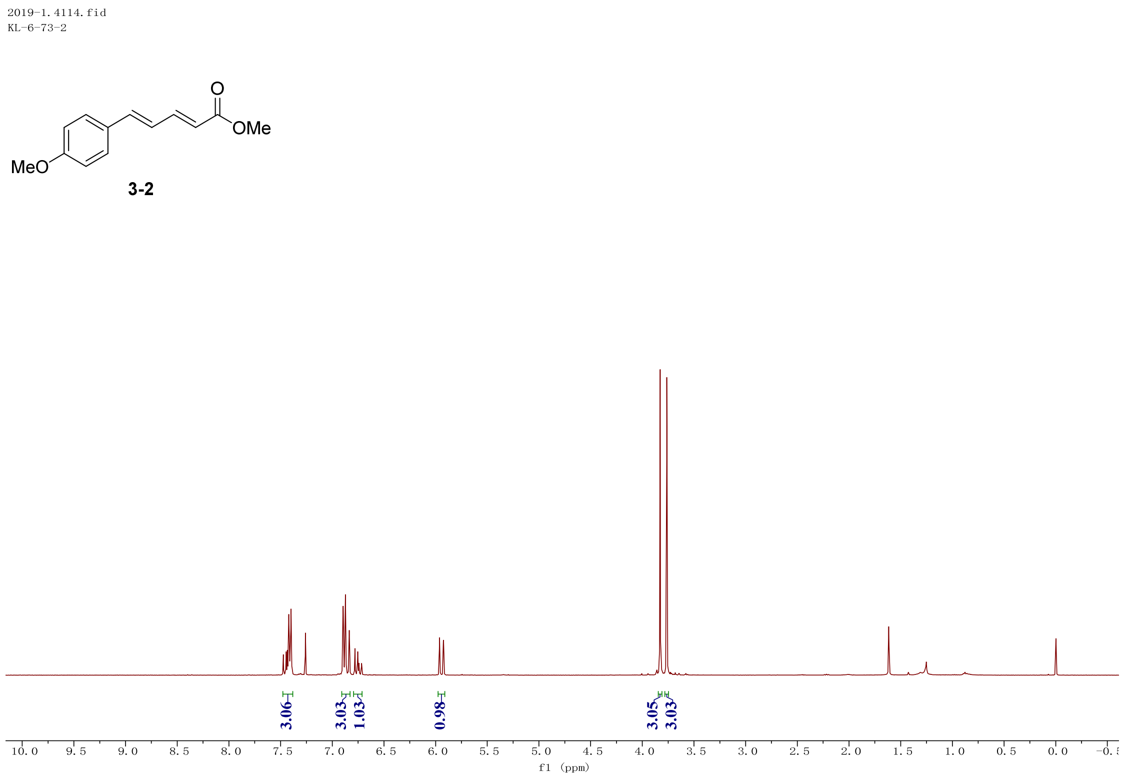

Supplementary Fig. 53

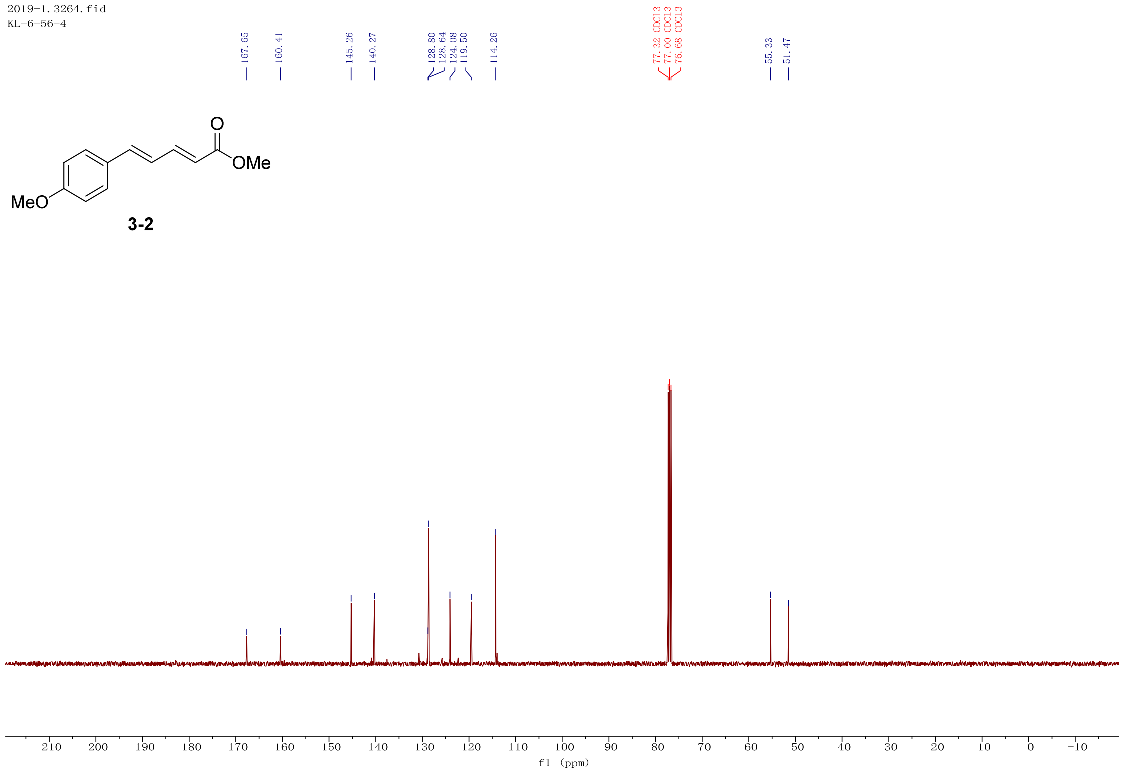

Supplementary Fig. 54

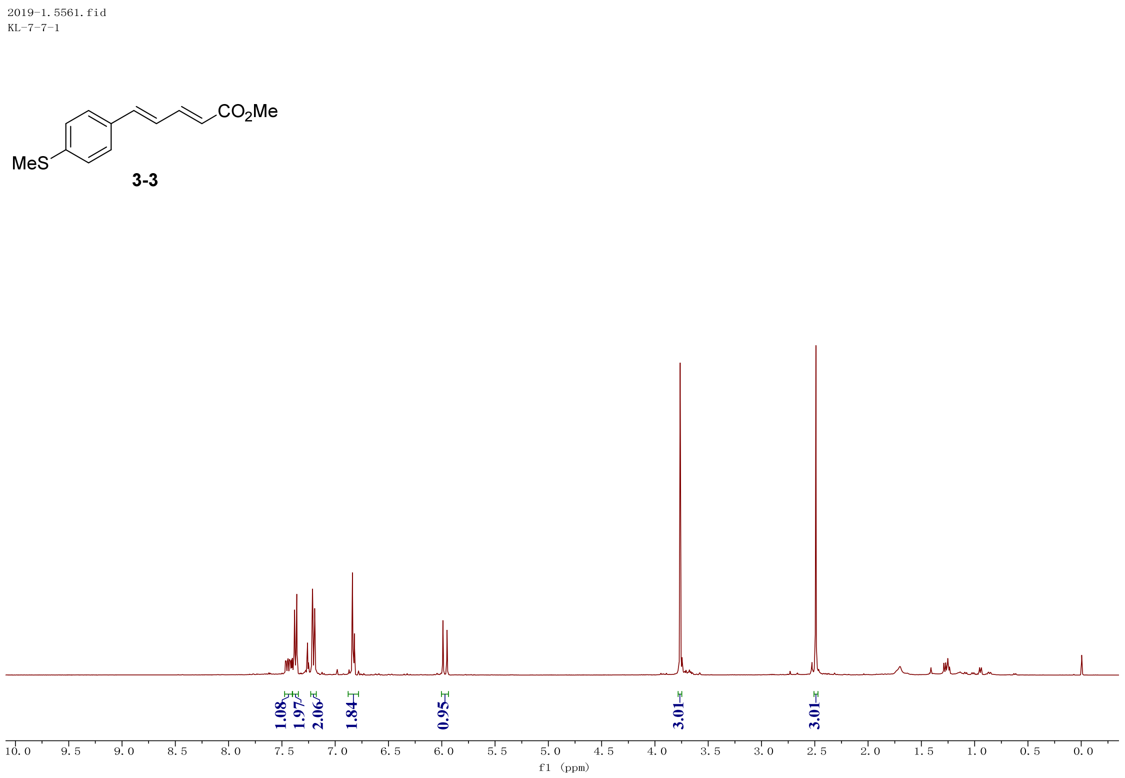

Supplementary Fig. 55

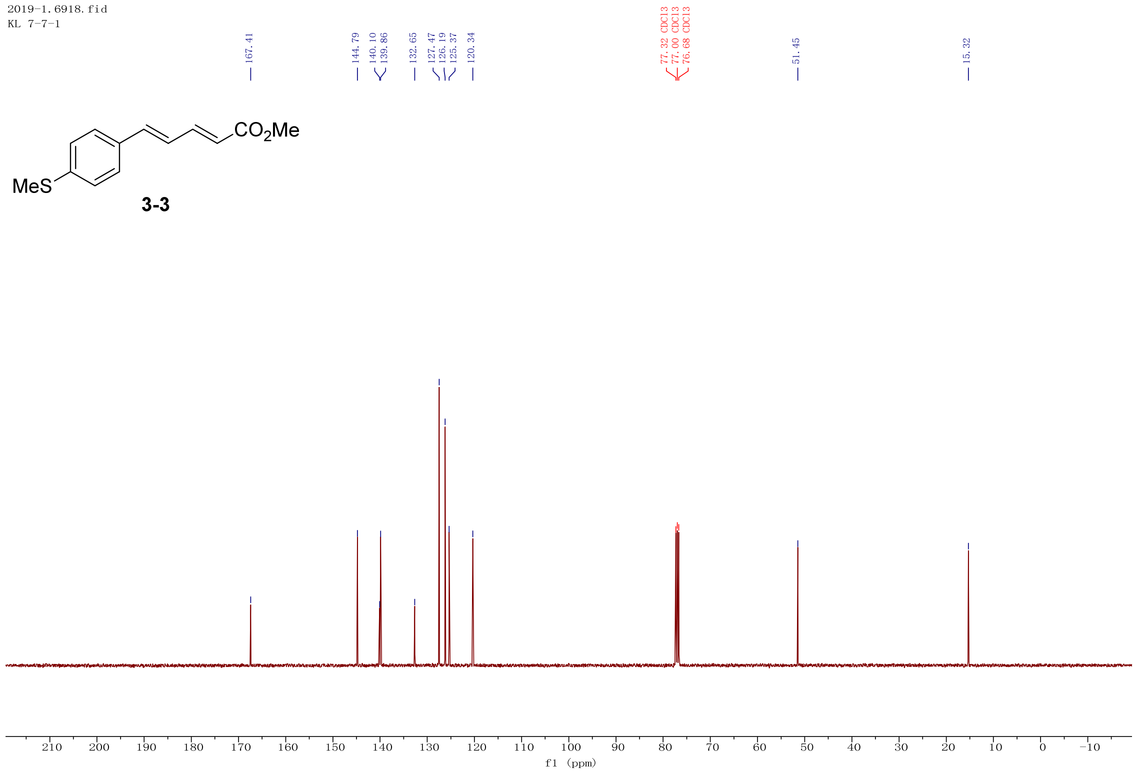

Supplementary Fig. 56

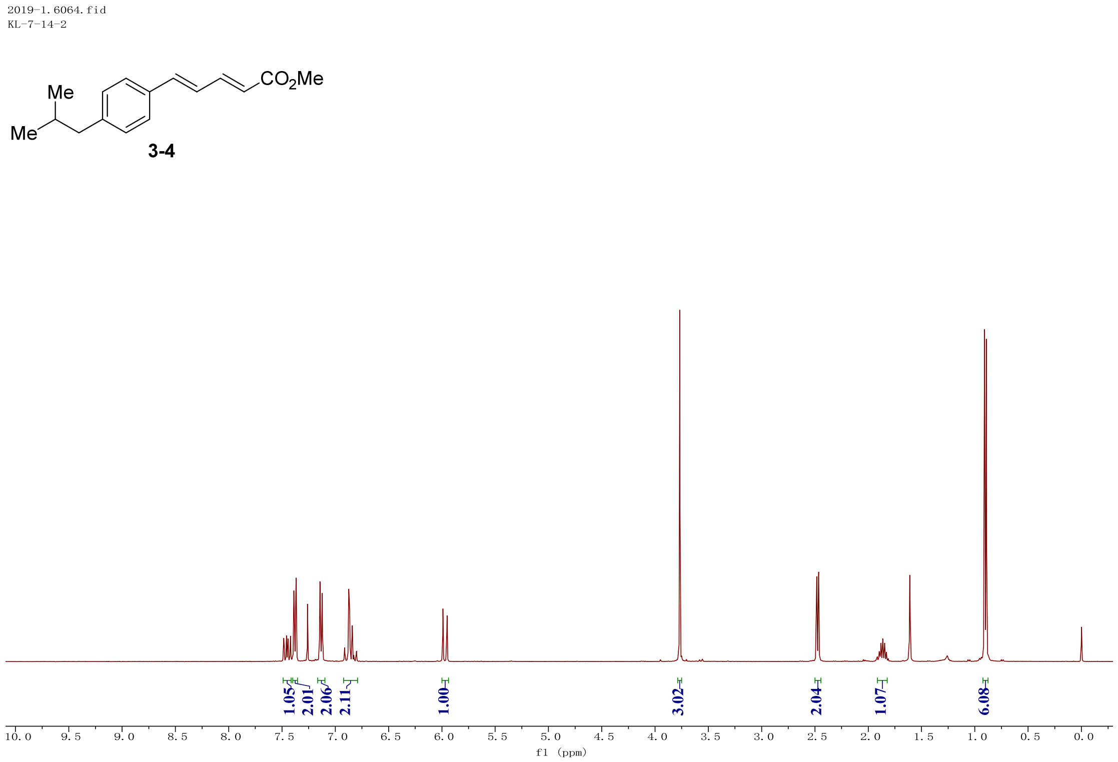

Supplementary Fig. 57

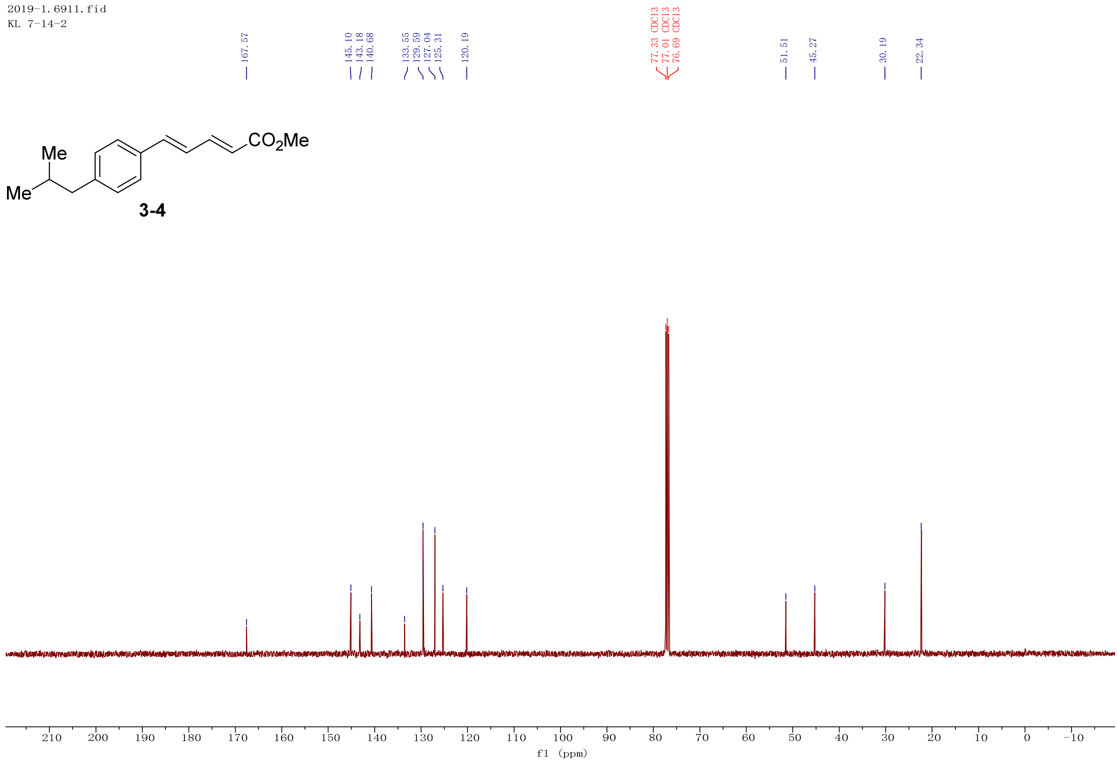

Supplementary Fig. 58

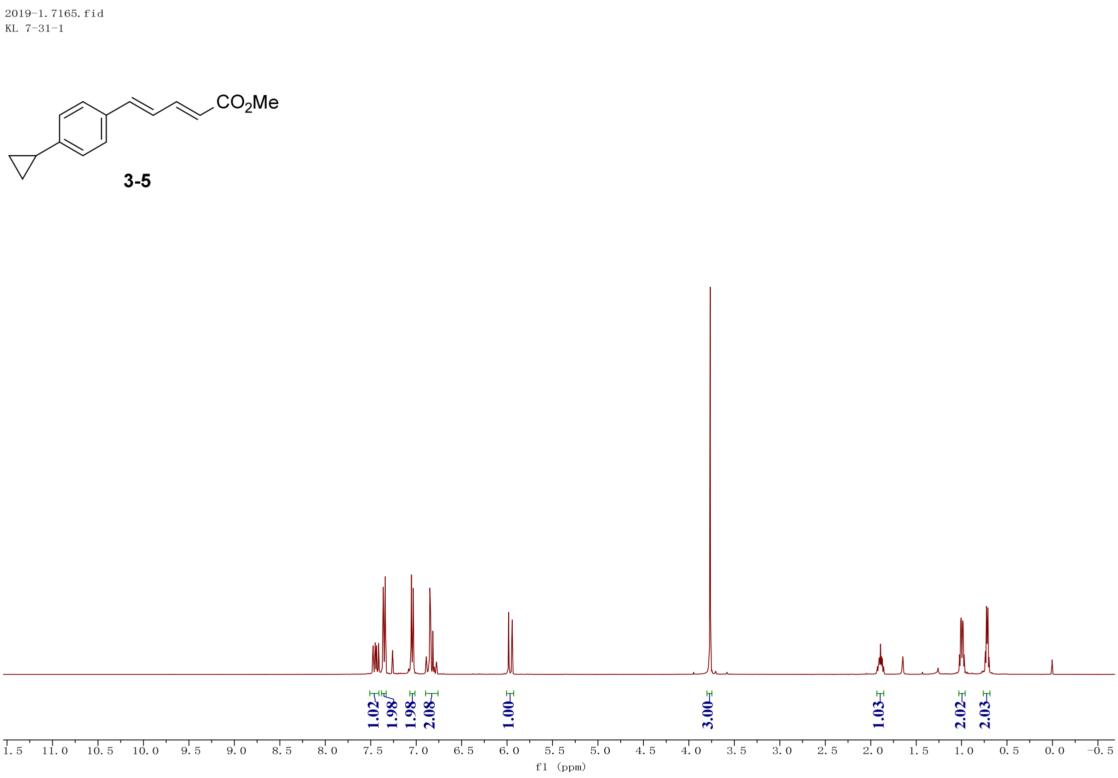

Supplementary Fig. 59

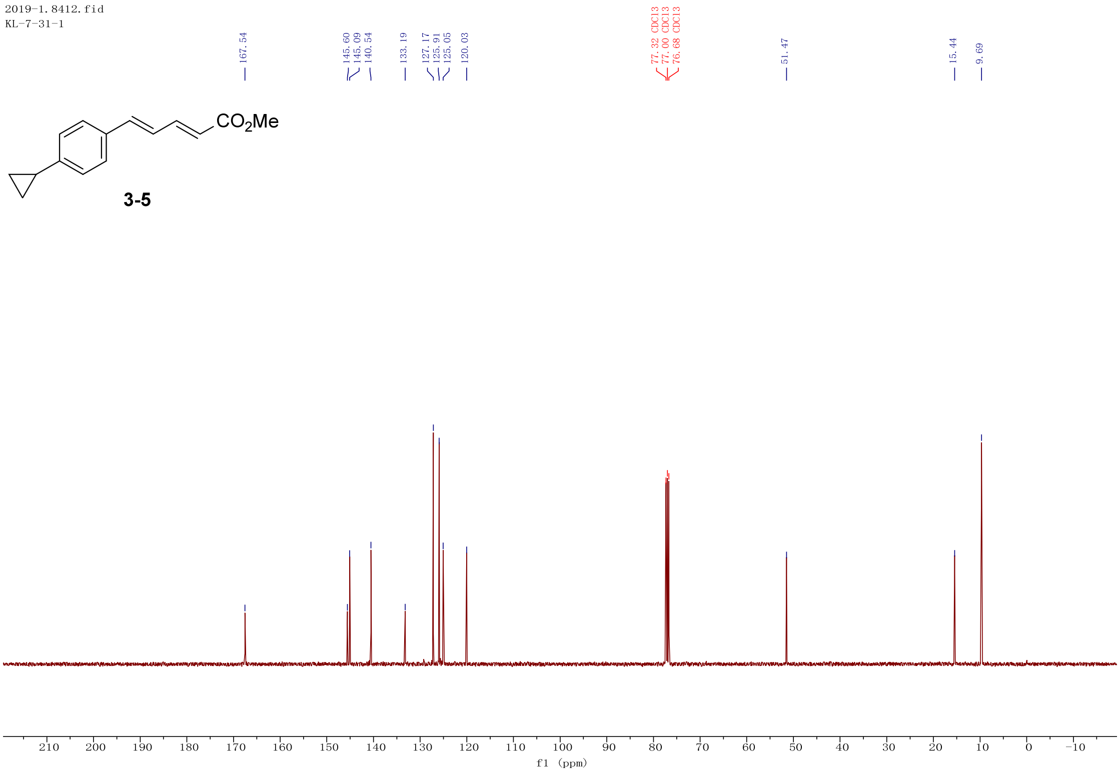

Supplementary Fig. 60

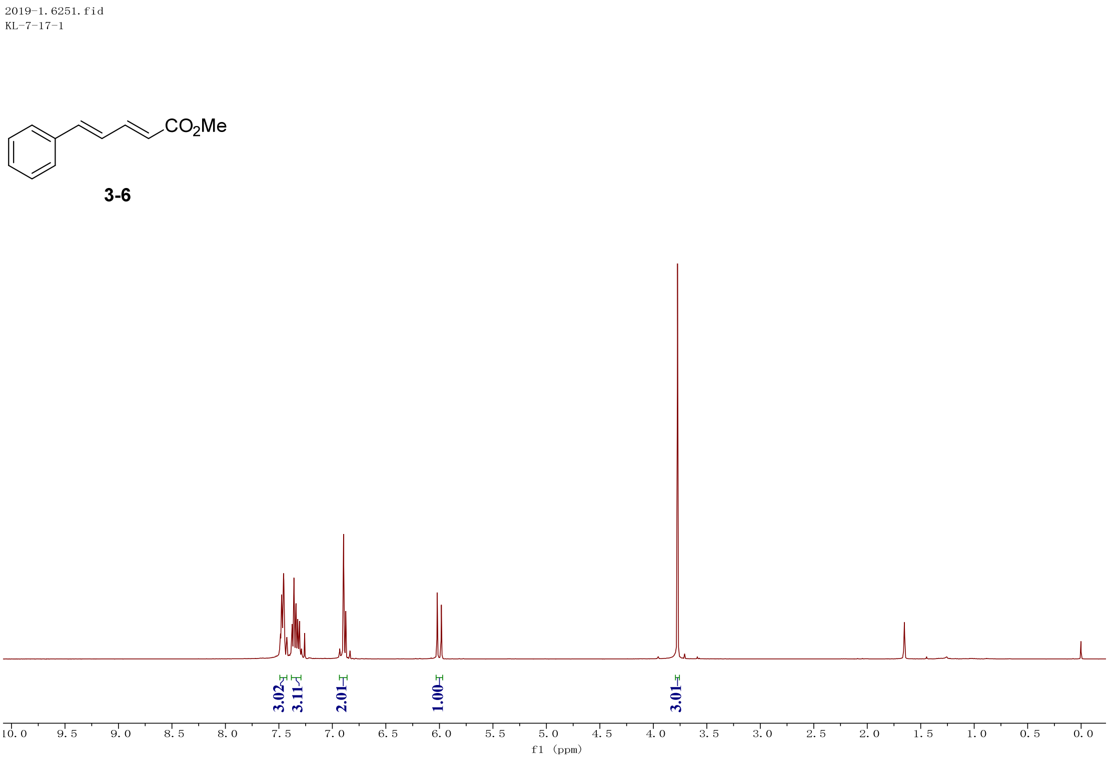

Supplementary Fig. 61

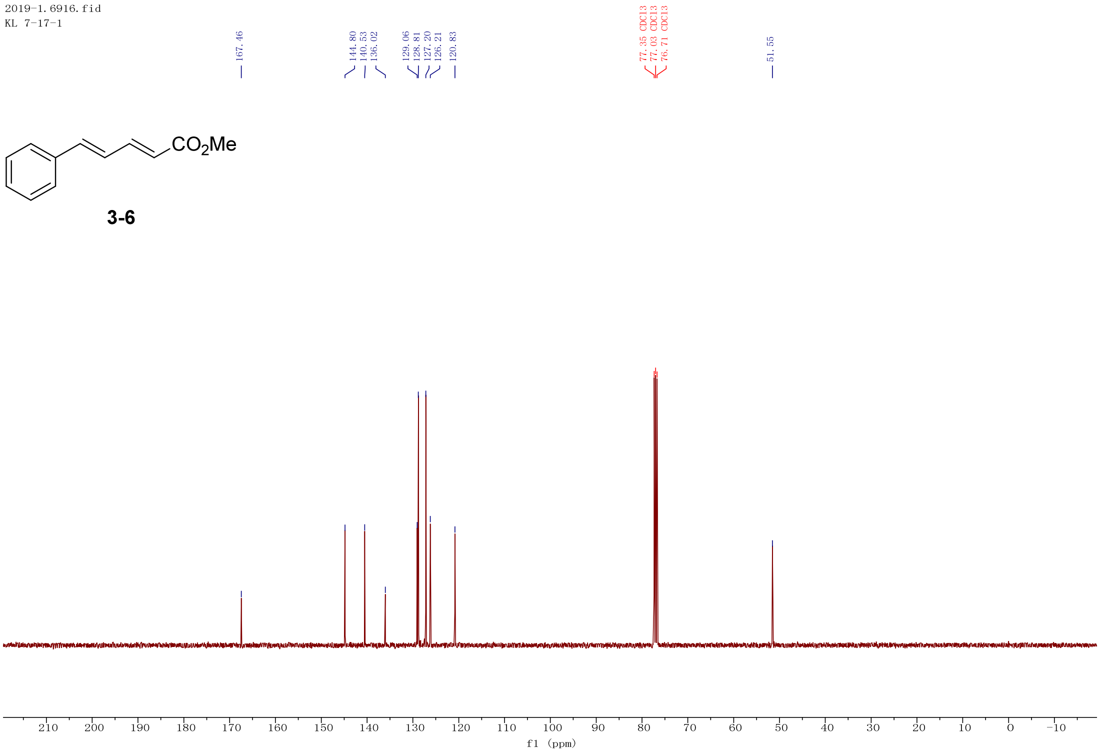

Supplementary Fig. 62

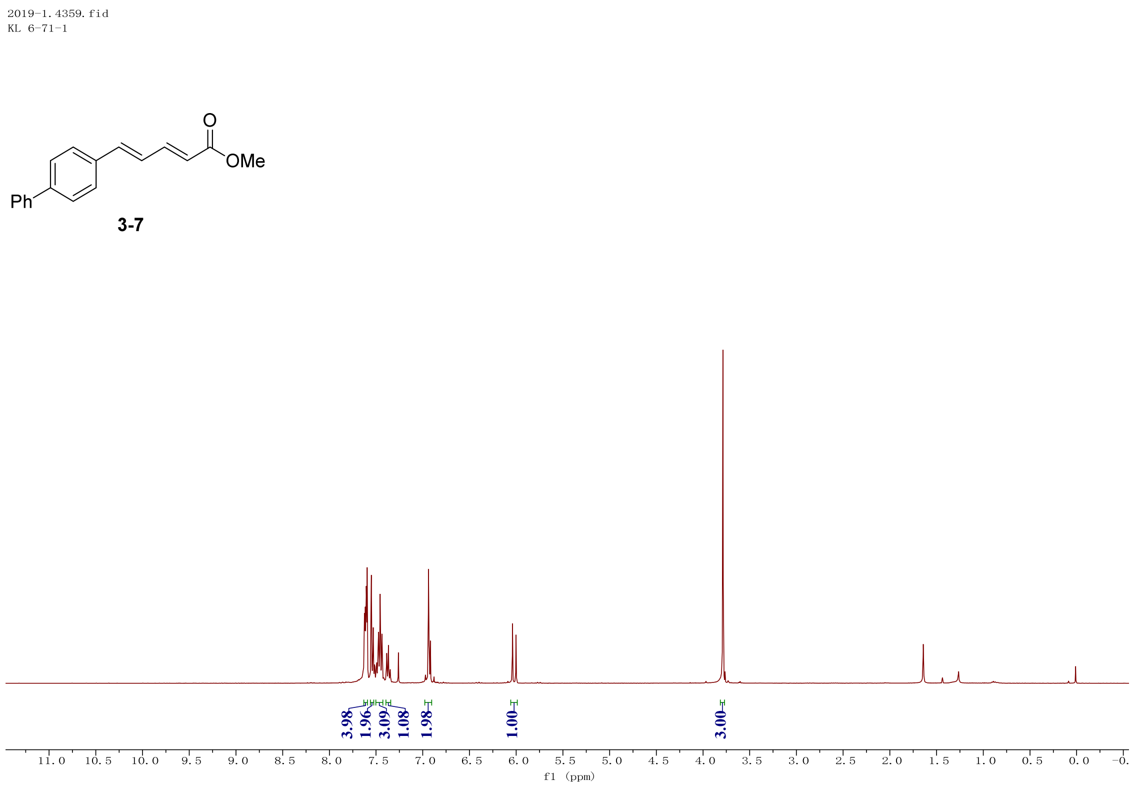

Supplementary Fig. 63

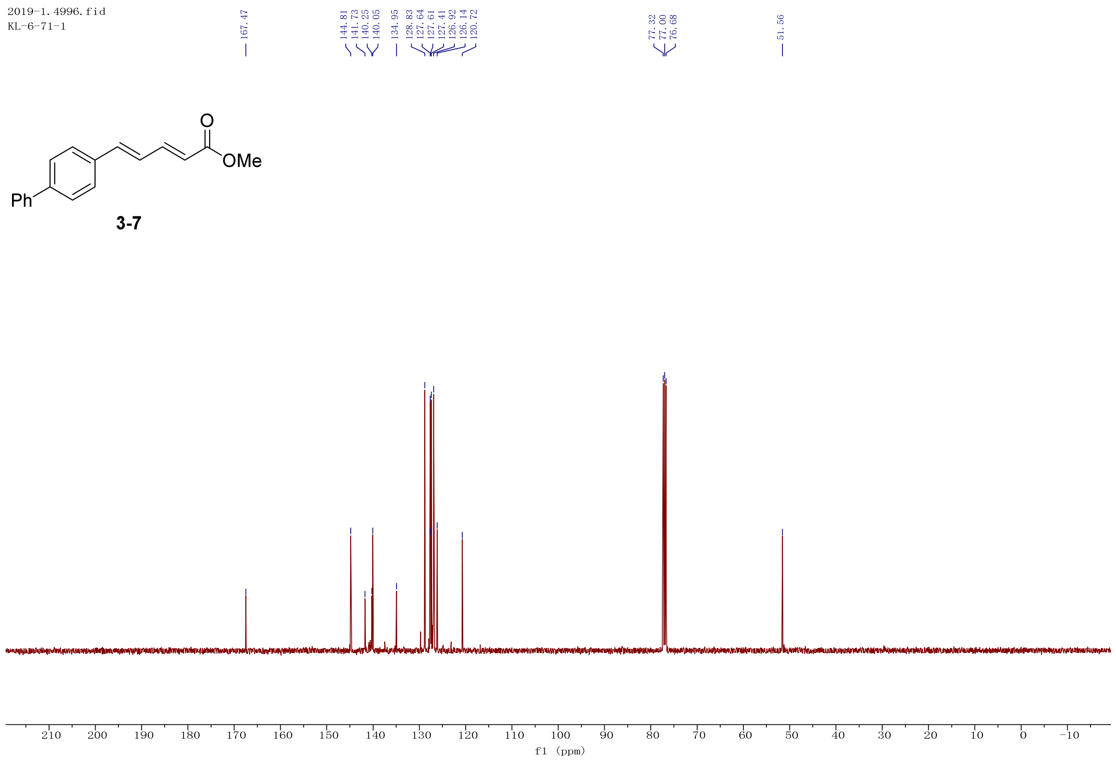

Supplementary Fig. 64

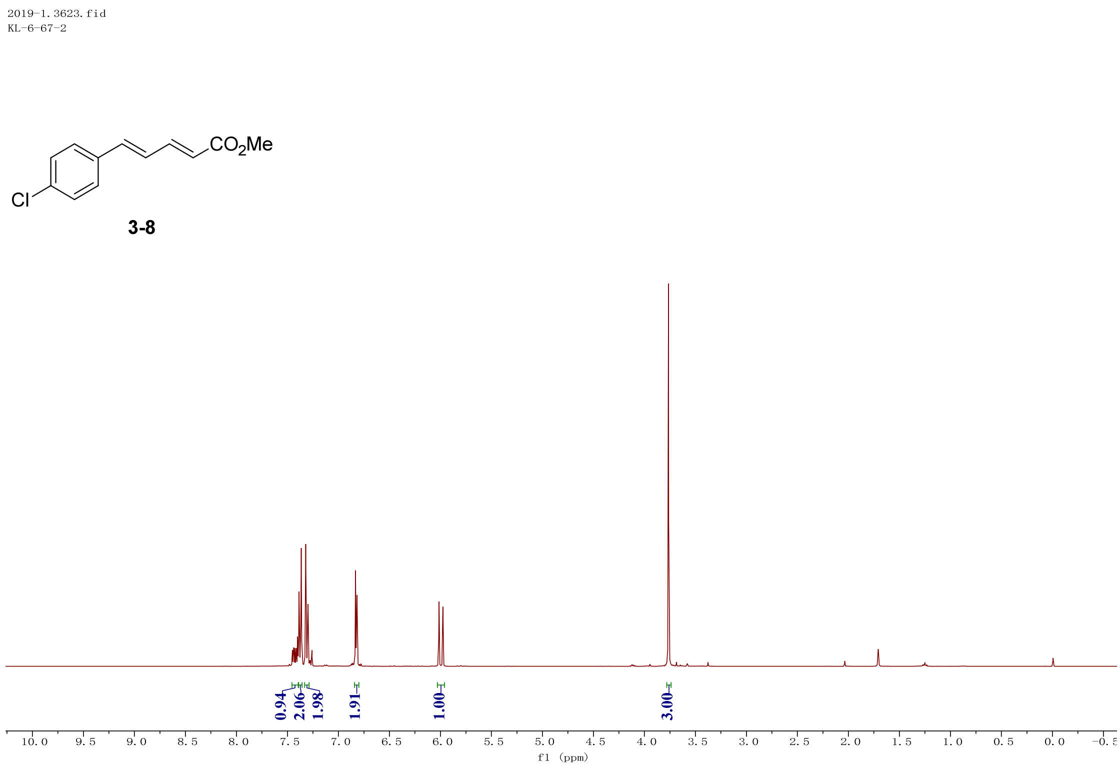

Supplementary Fig. 65

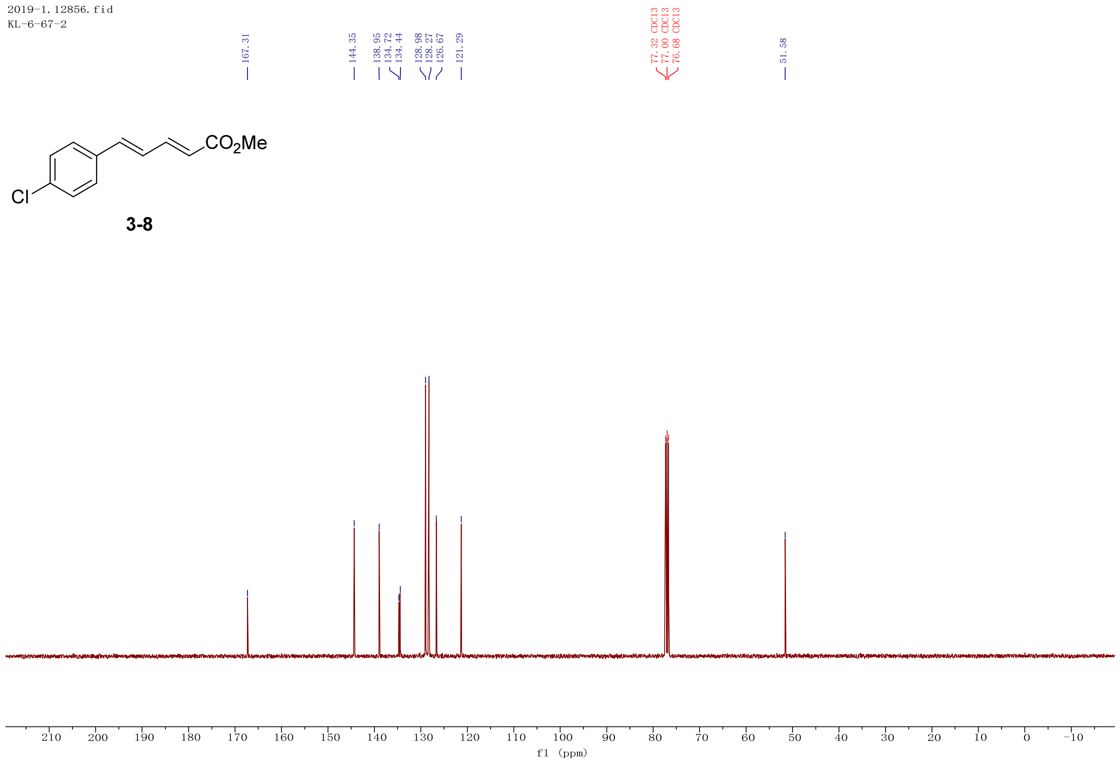

Supplementary Fig. 66

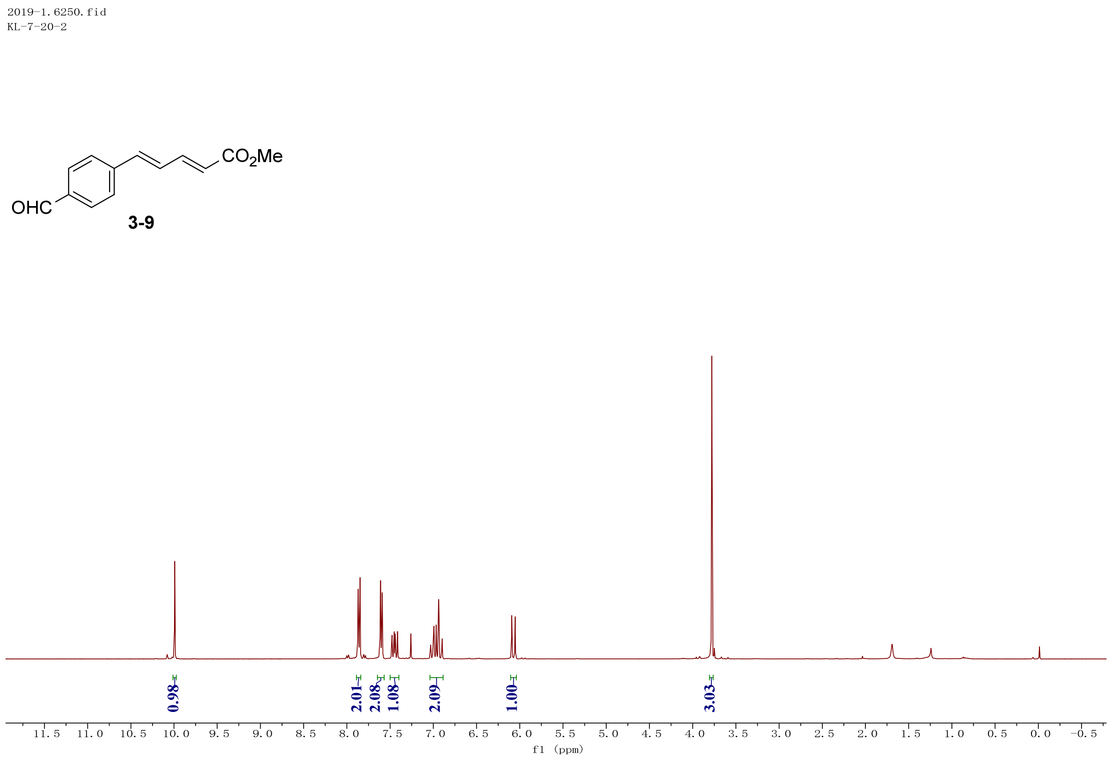

Supplementary Fig. 67

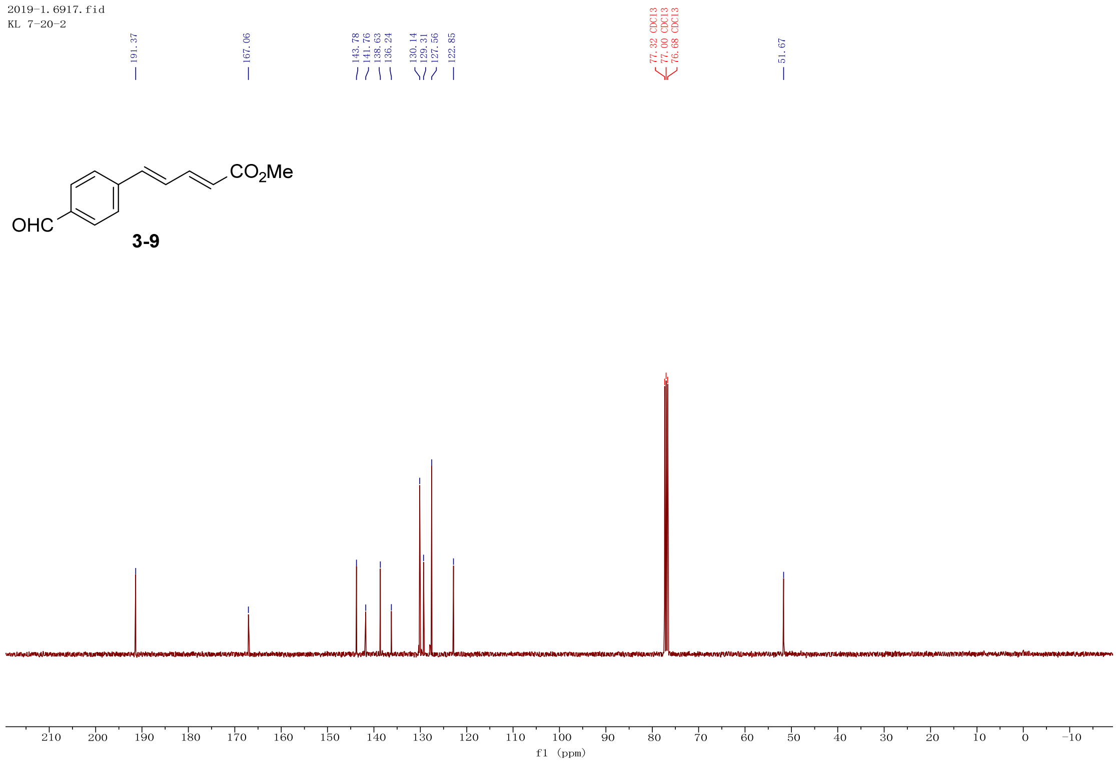

Supplementary Fig. 68

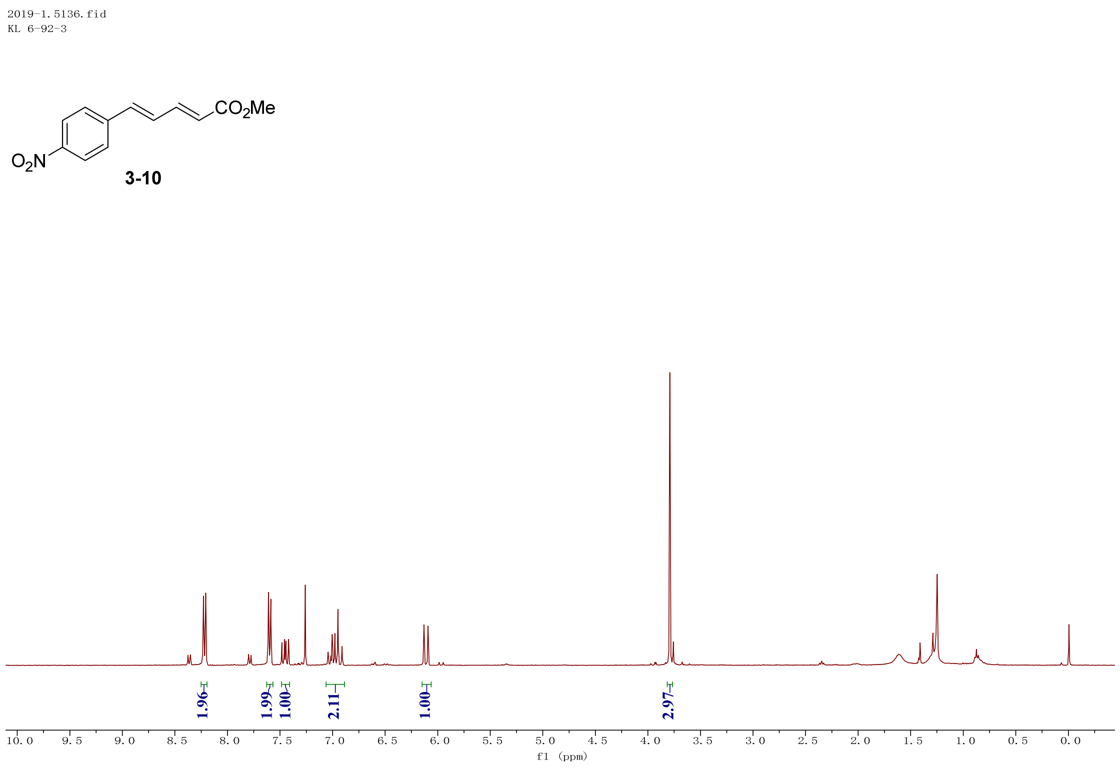

Supplementary Fig. 69

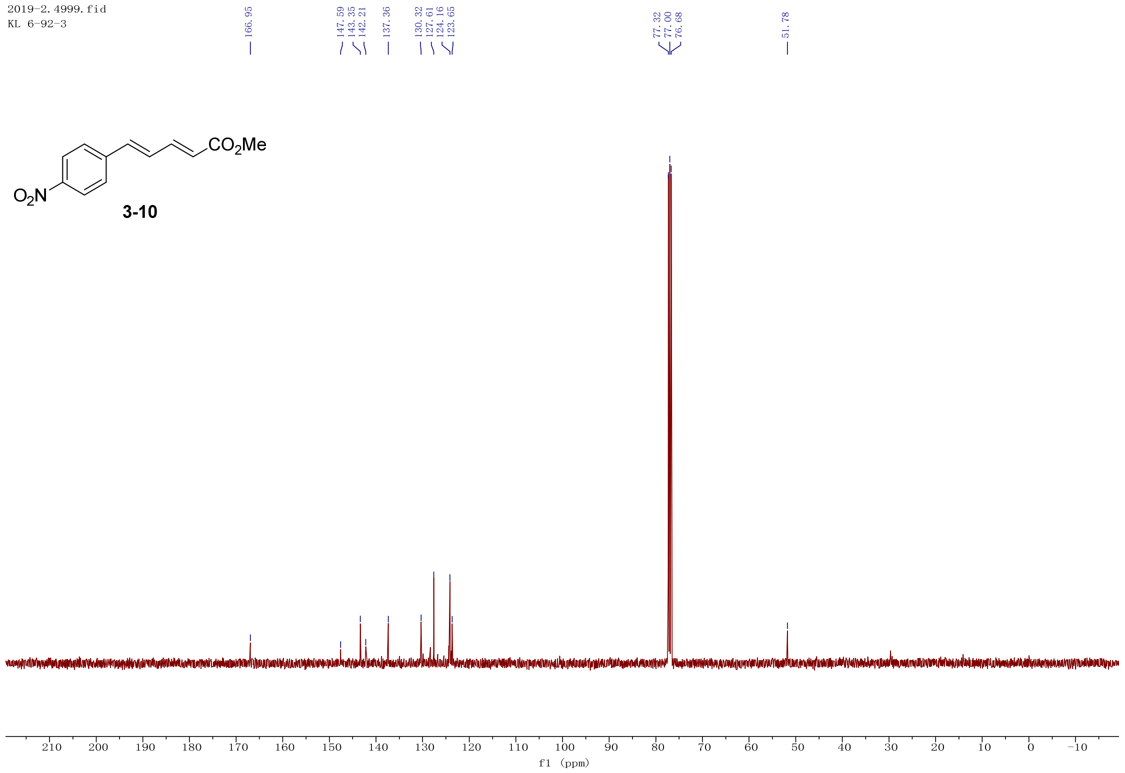

Supplementary Fig. 70

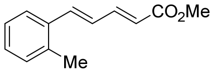

3-11

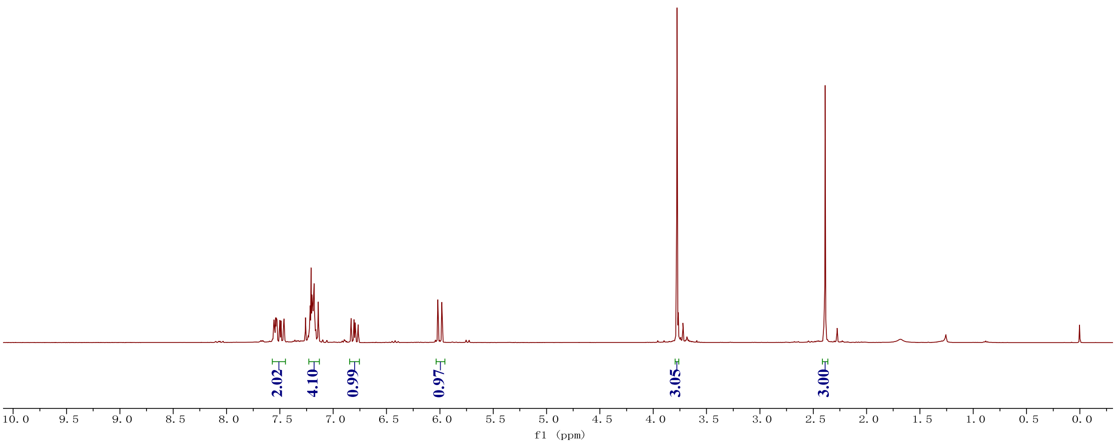

Supplementary Fig. 71

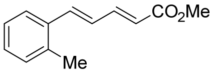

3-11

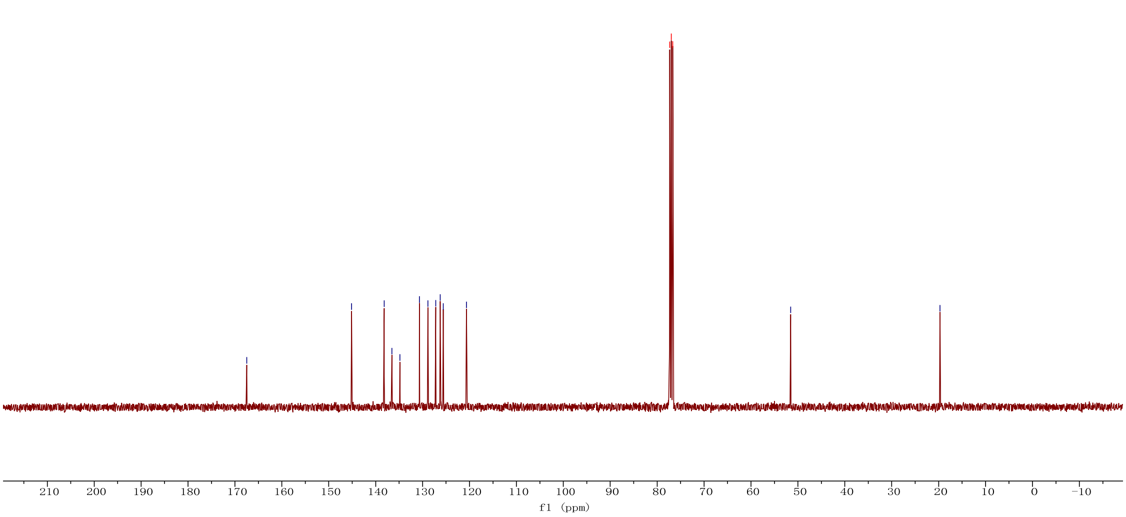

Supplementary Fig. 72

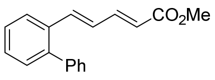

3-12

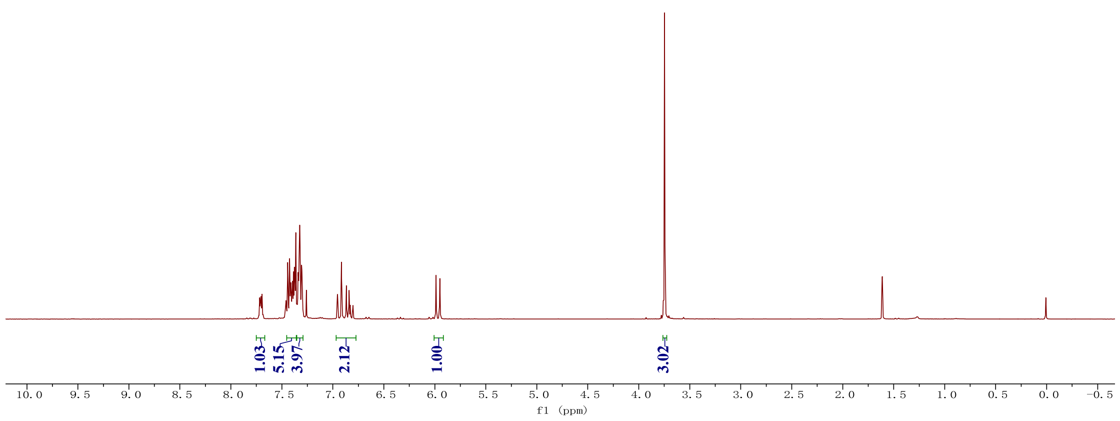

Supplementary Fig. 73

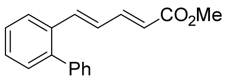

3-12

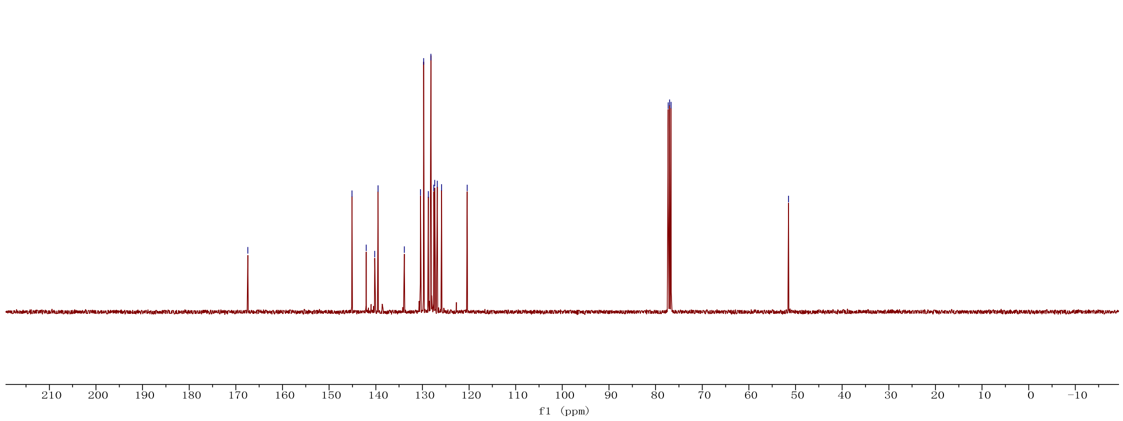

Supplementary Fig. 74

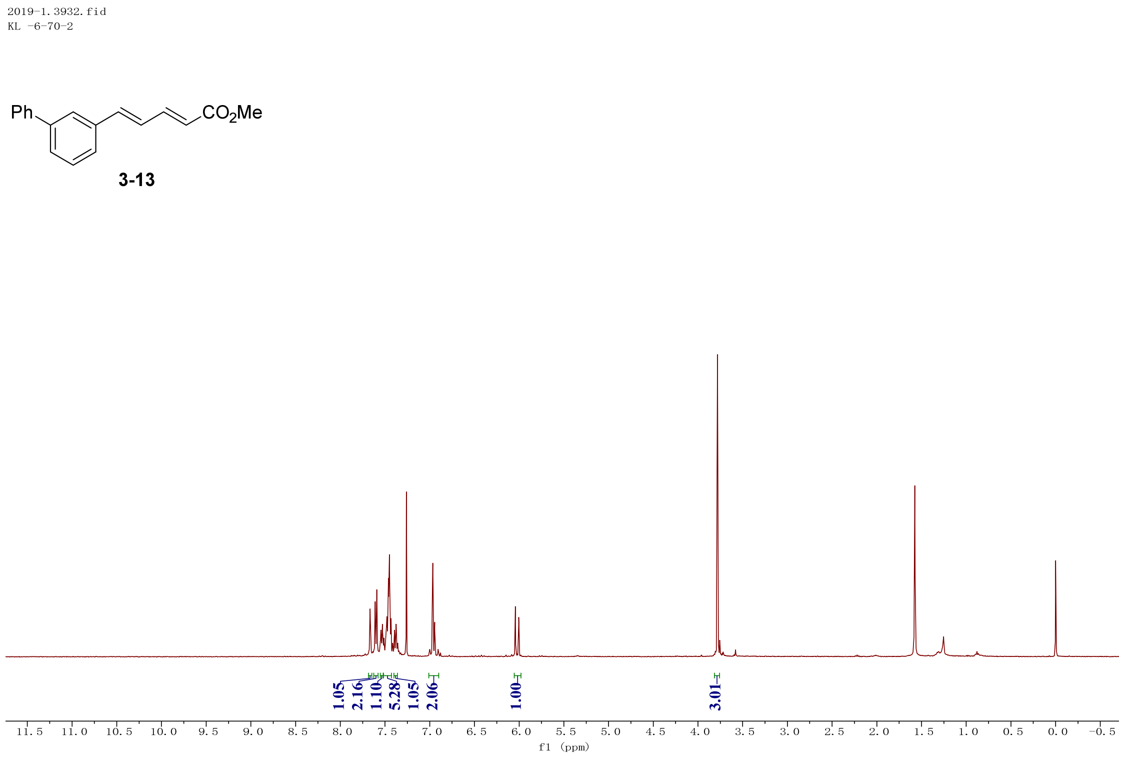

Supplementary Fig. 75

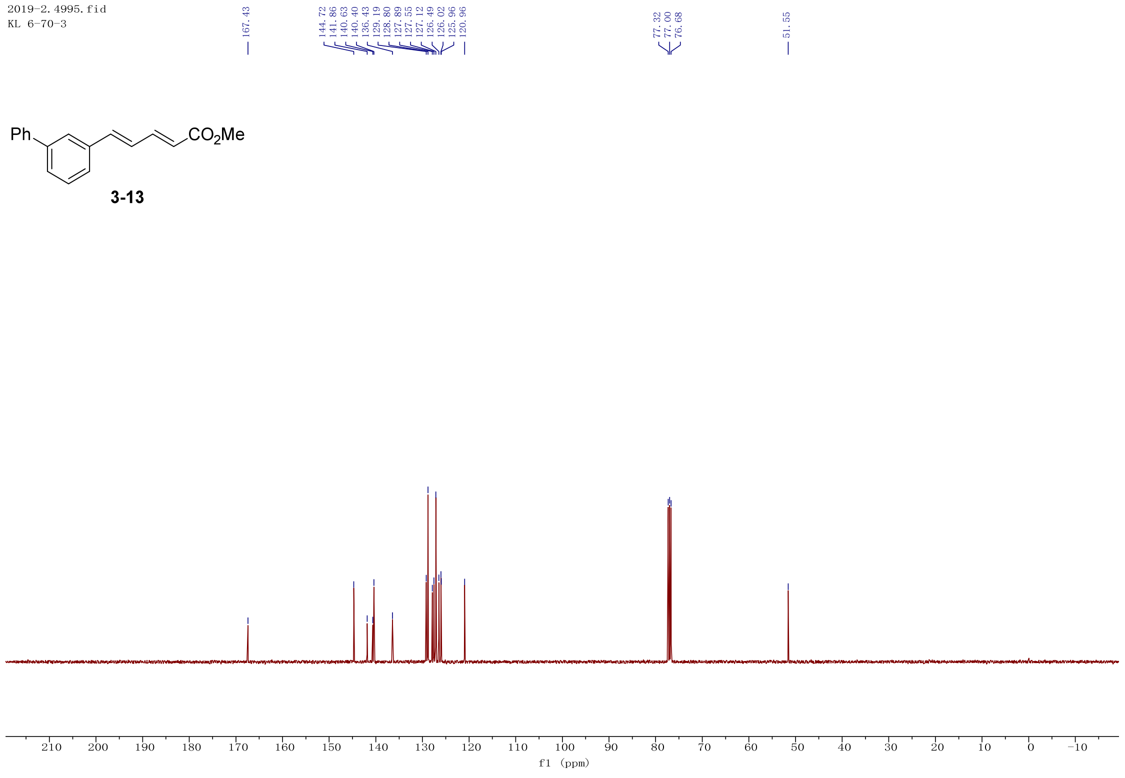

Supplementary Fig. 76

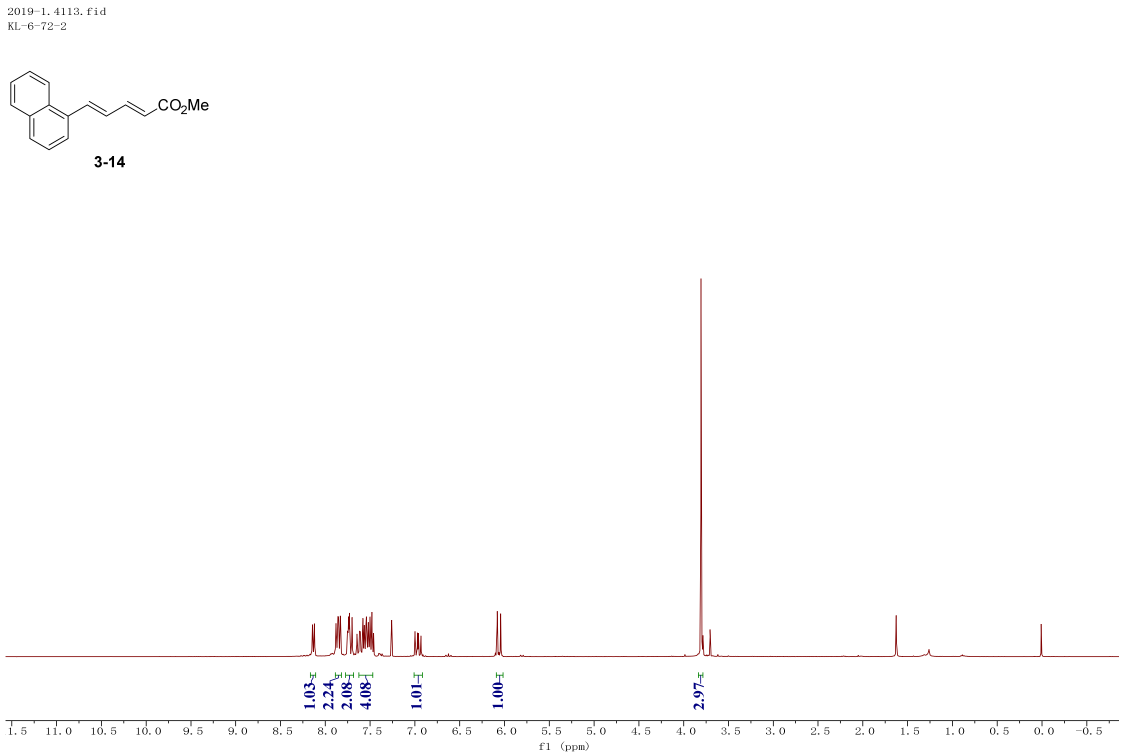

Supplementary Fig. 77

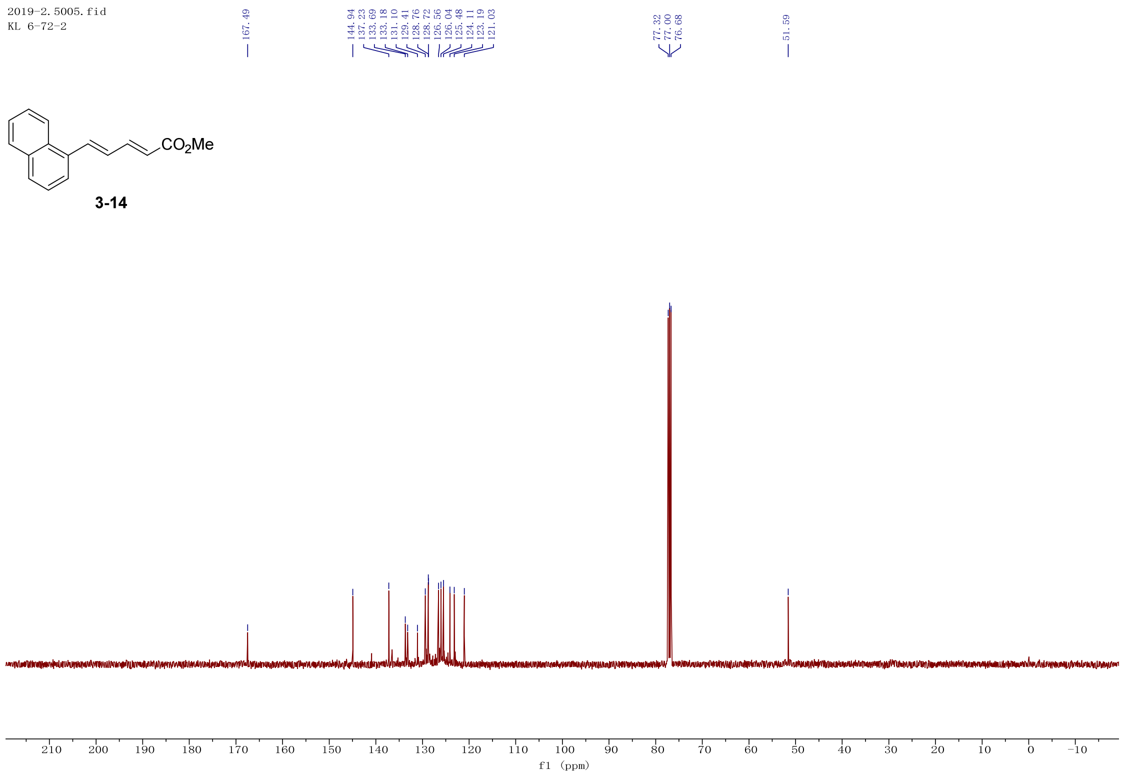

Supplementary Fig. 78

2019-1.6861.fid
KL-7-29-2

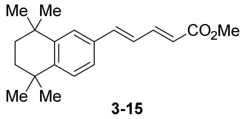

3-15

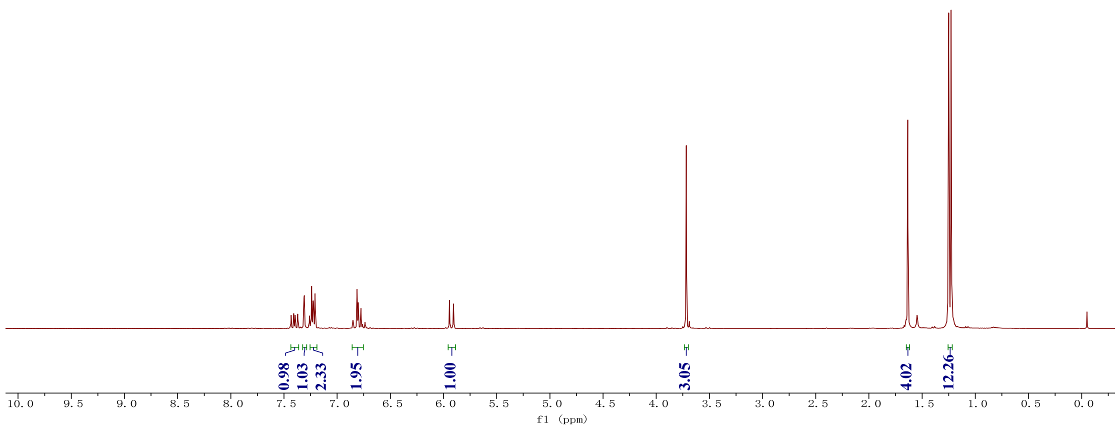

Supplementary Fig. 79

2019-1.6862.fid
KL-7-29-2

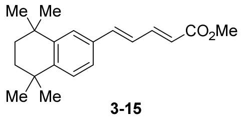

3-15

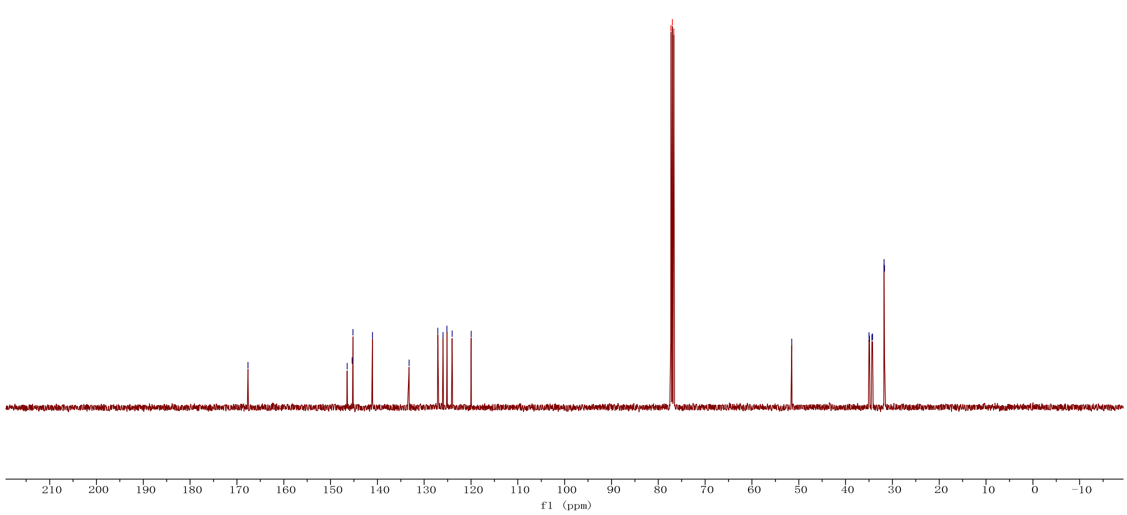

Supplementary Fig. 80

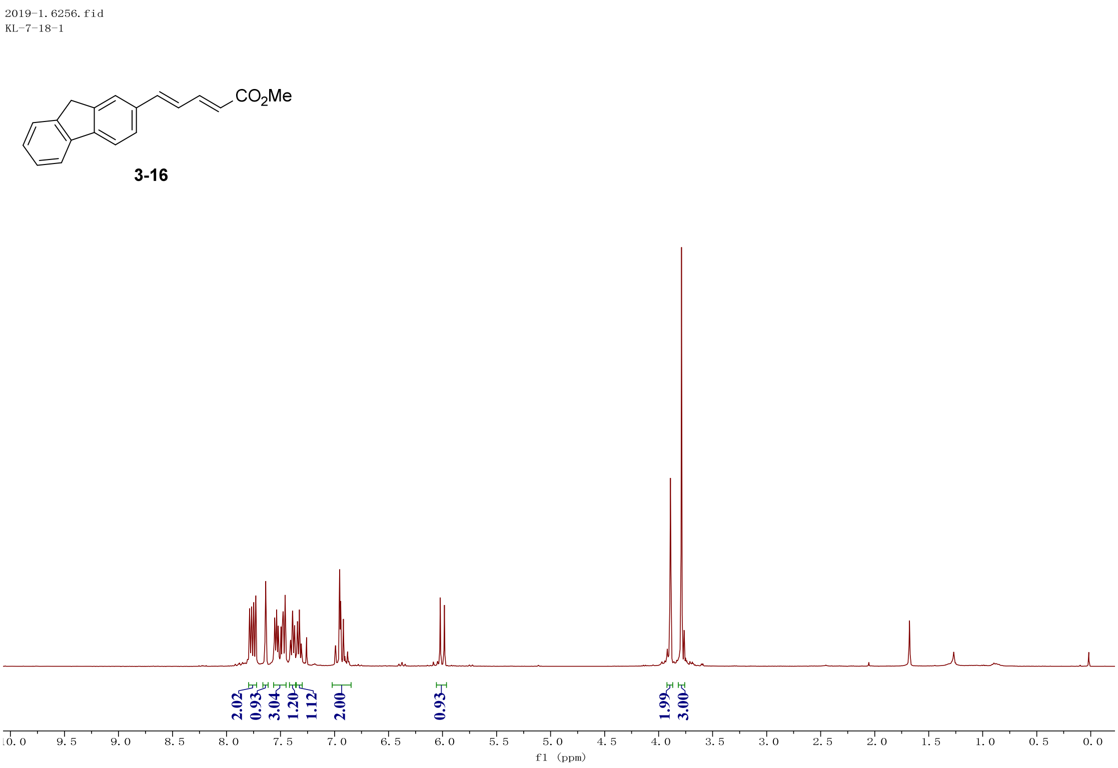

Supplementary Fig. 81

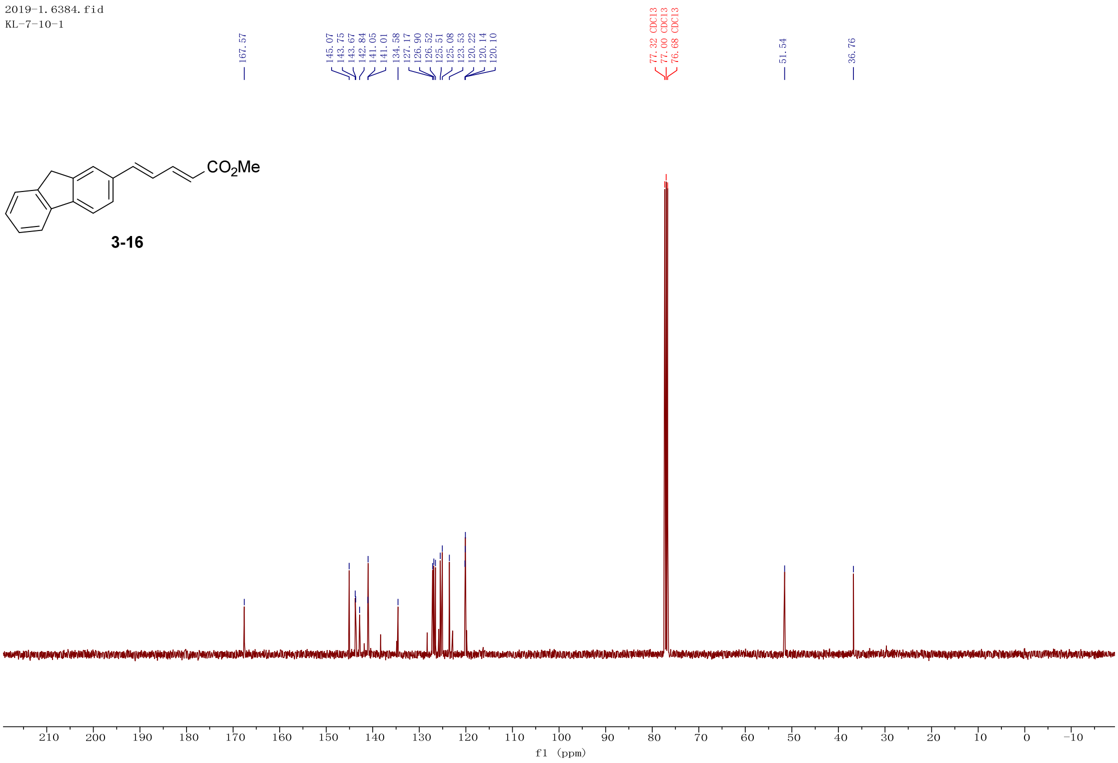

Supplementary Fig. 82

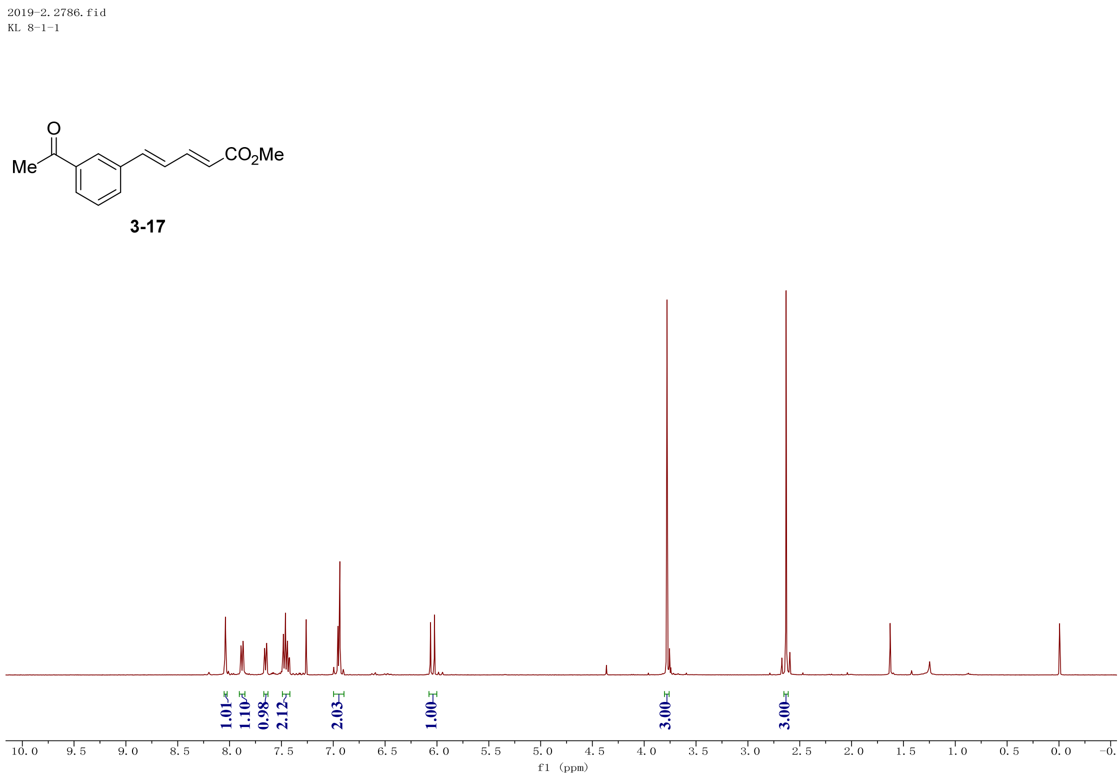

3-17

Supplementary Fig. 83

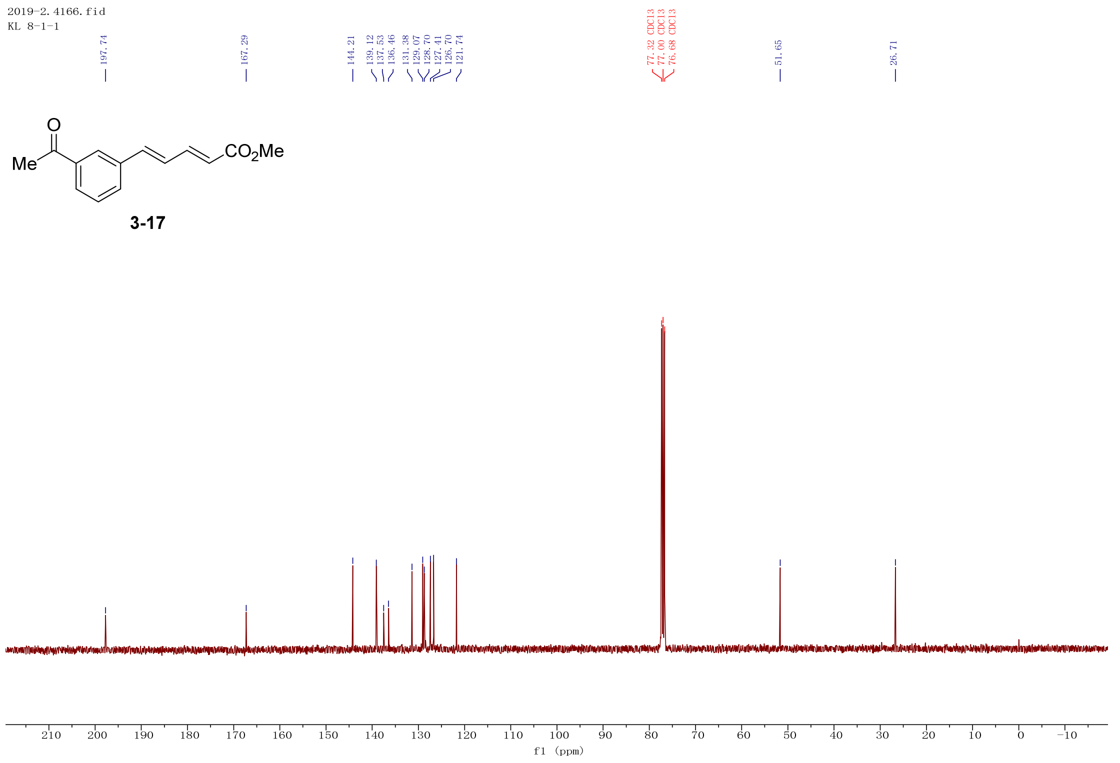

3-17

Supplementary Fig. 84

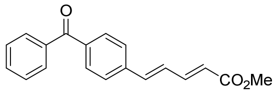

3-18

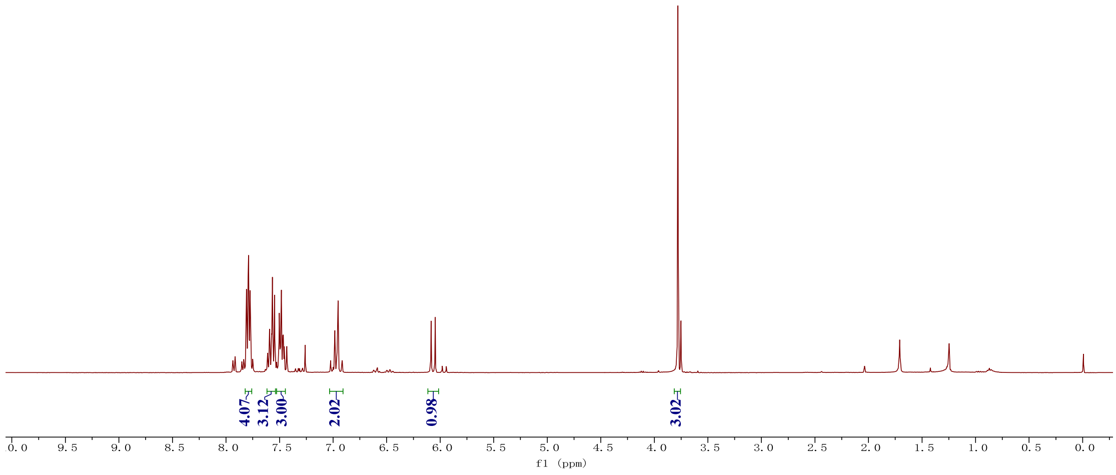

Supplementary Fig. 85

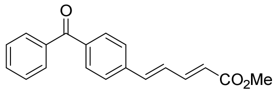

3-18

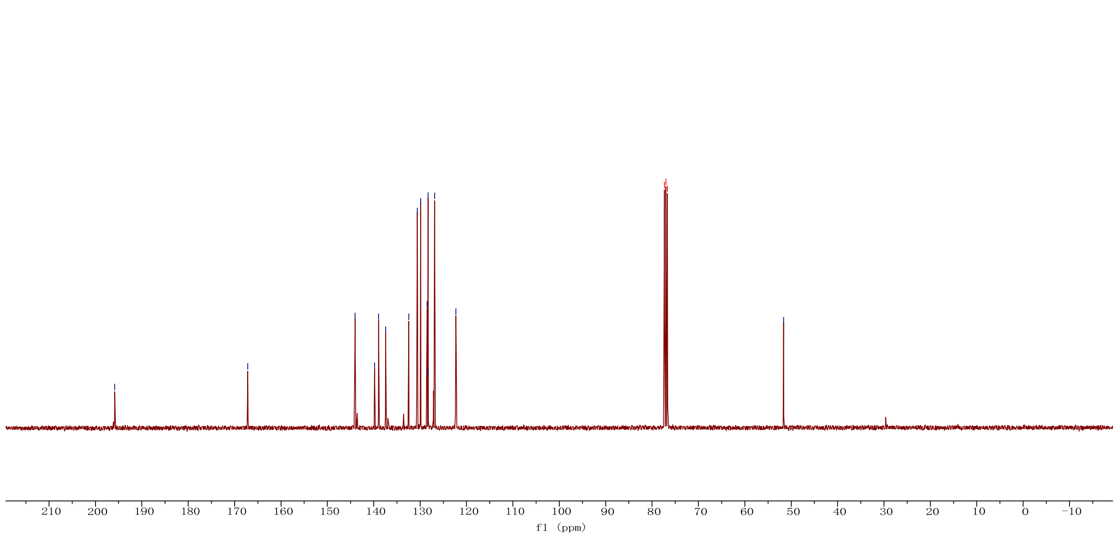

Supplementary Fig. 86

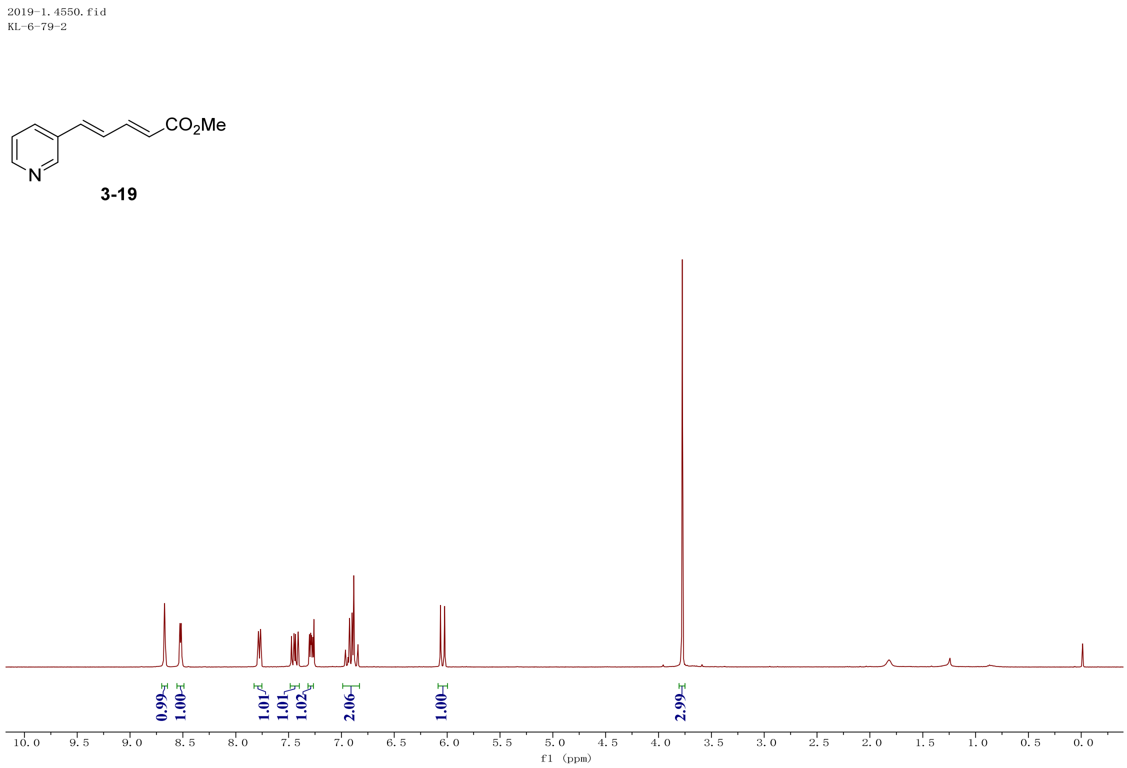

Supplementary Fig. 87

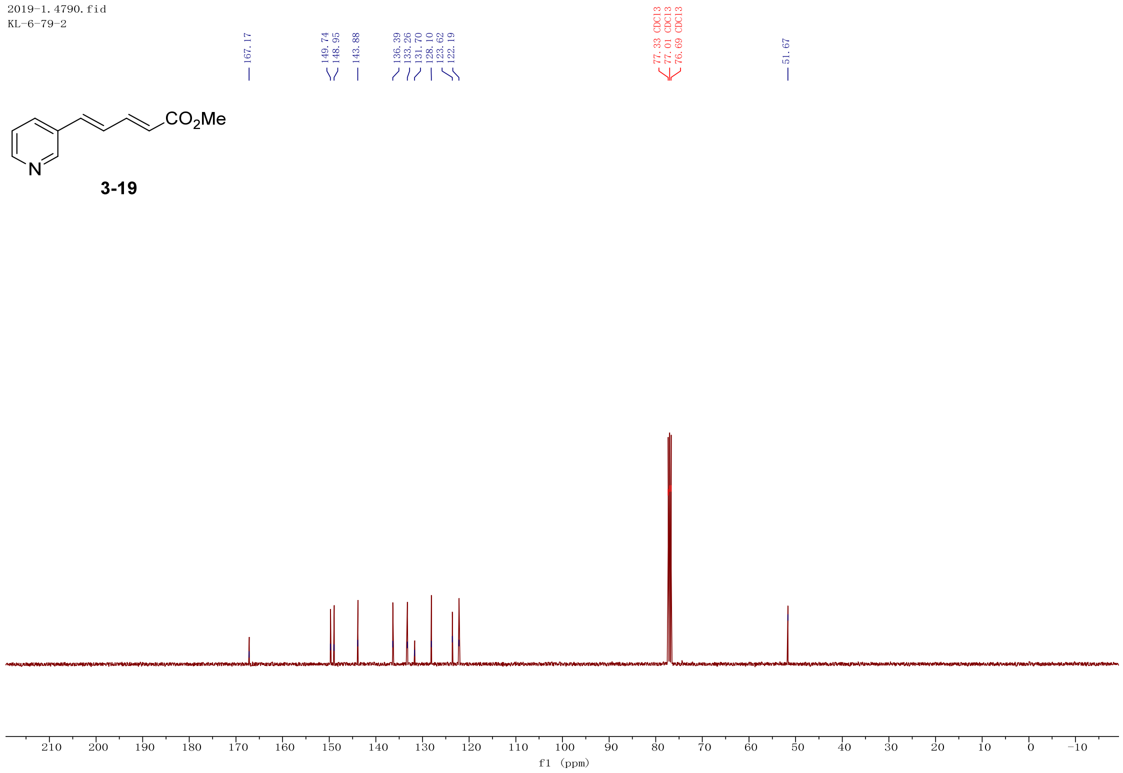

Supplementary Fig. 88

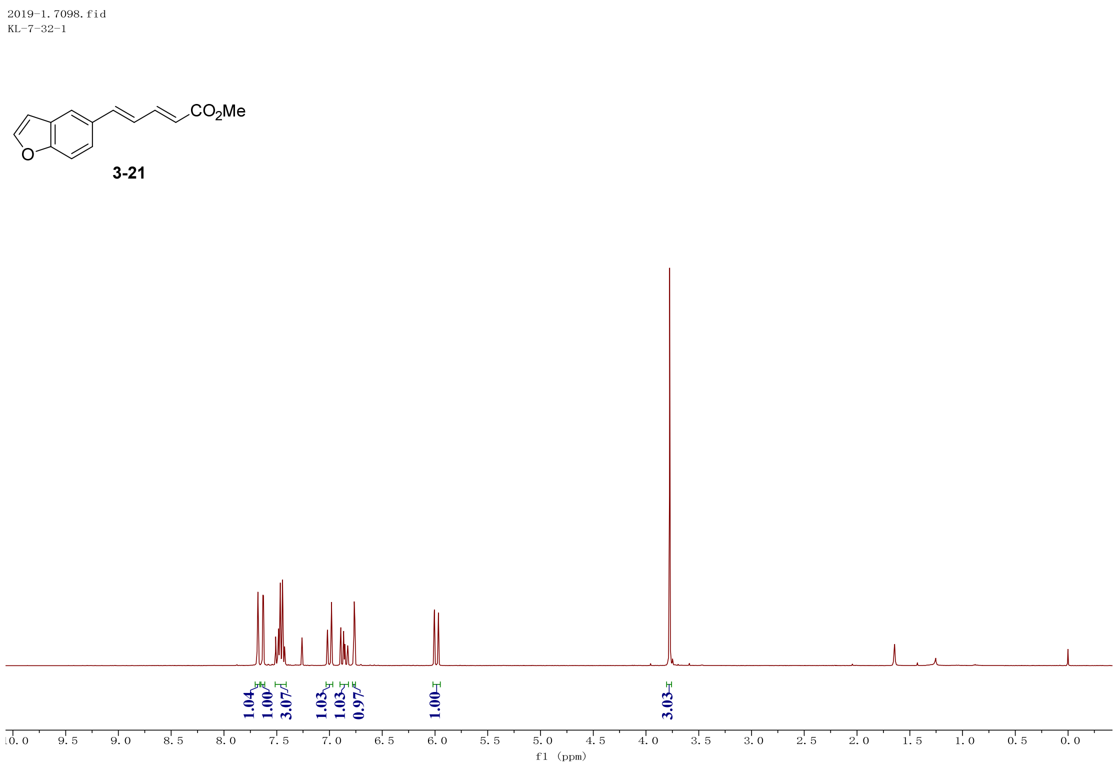

Supplementary Fig. 89

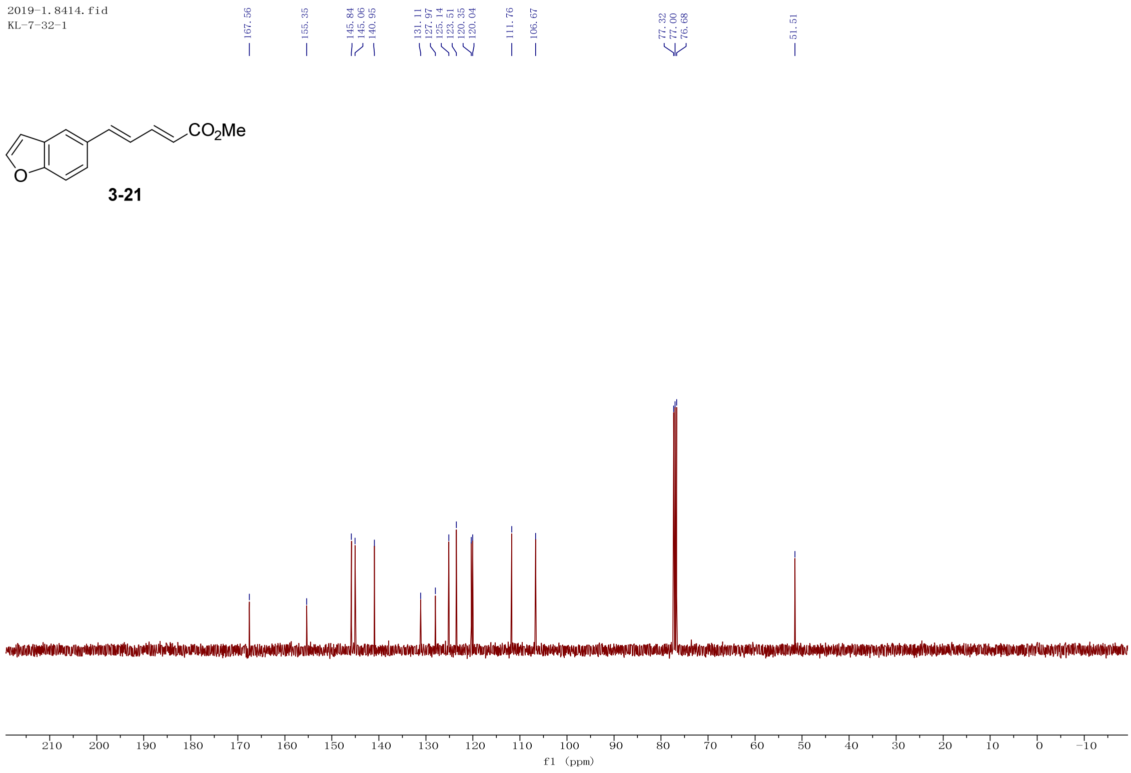

Supplementary Fig. 90

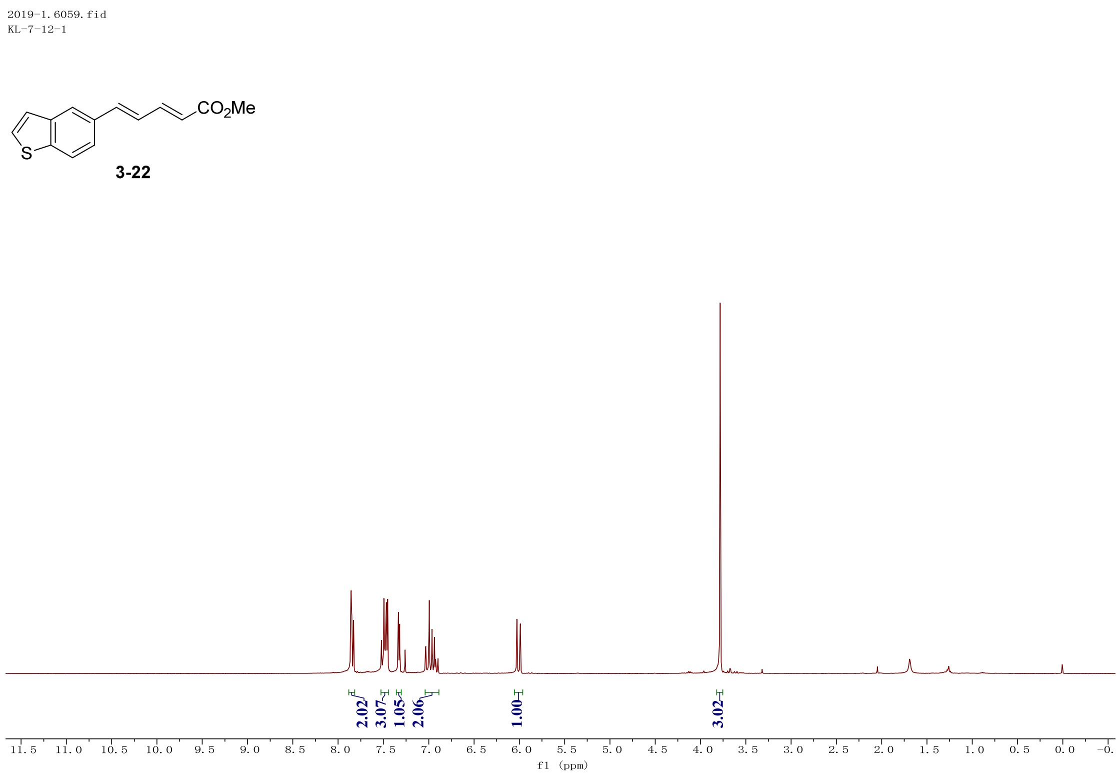

Supplementary Fig. 91

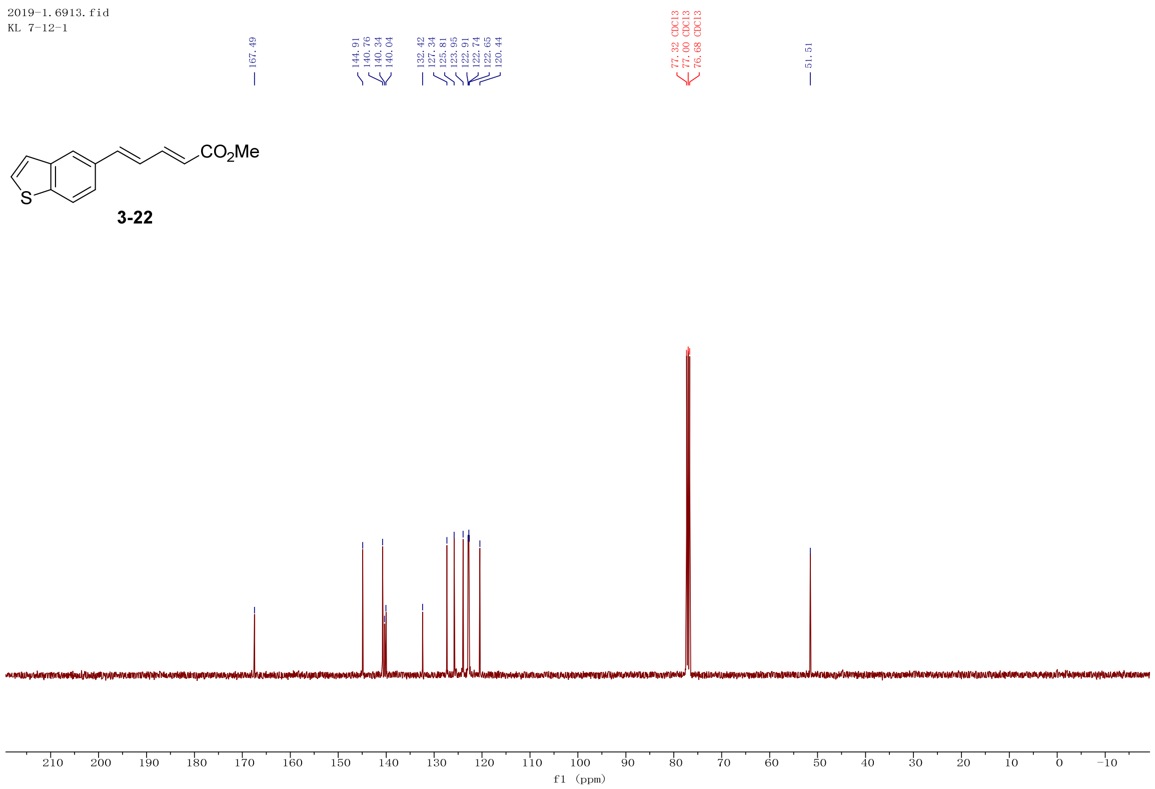

Supplementary Fig. 92

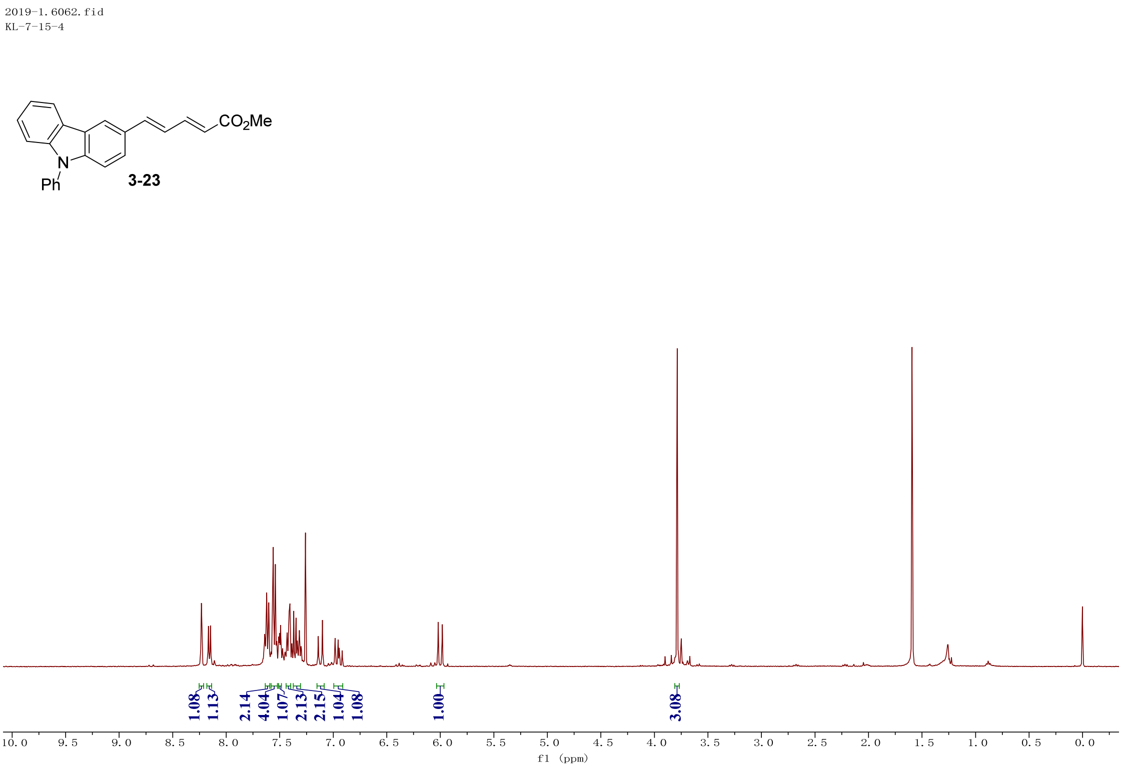

Supplementary Fig. 93

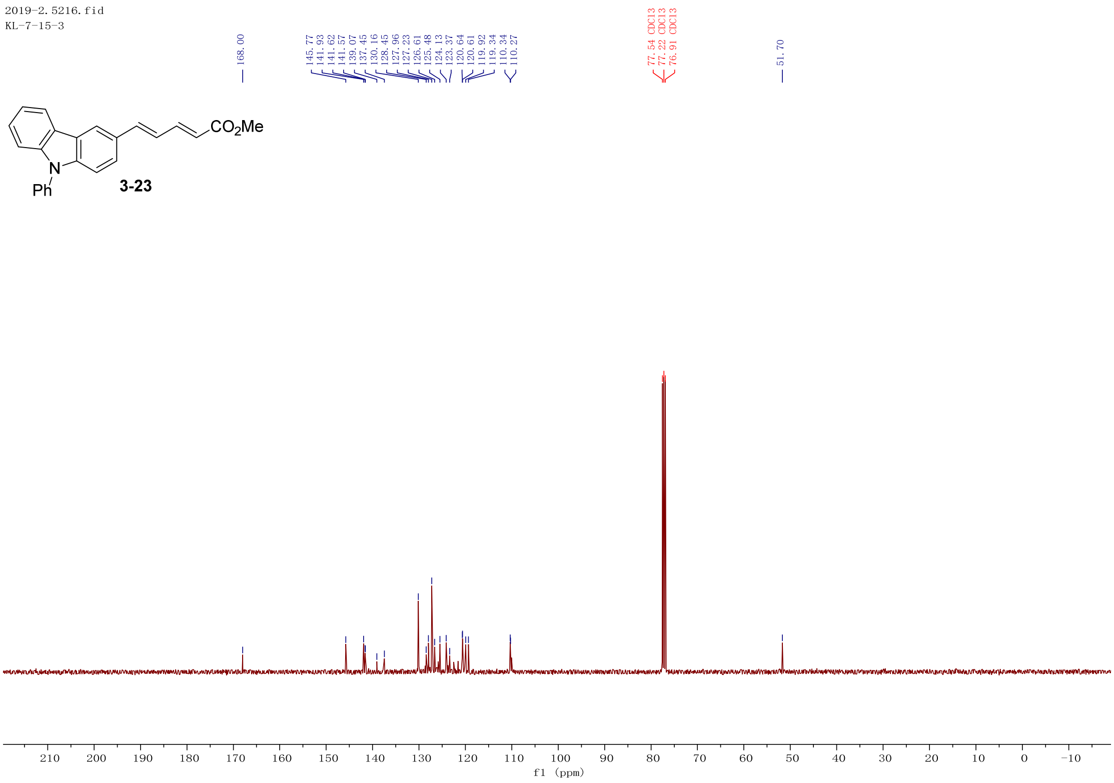

Supplementary Fig. 94

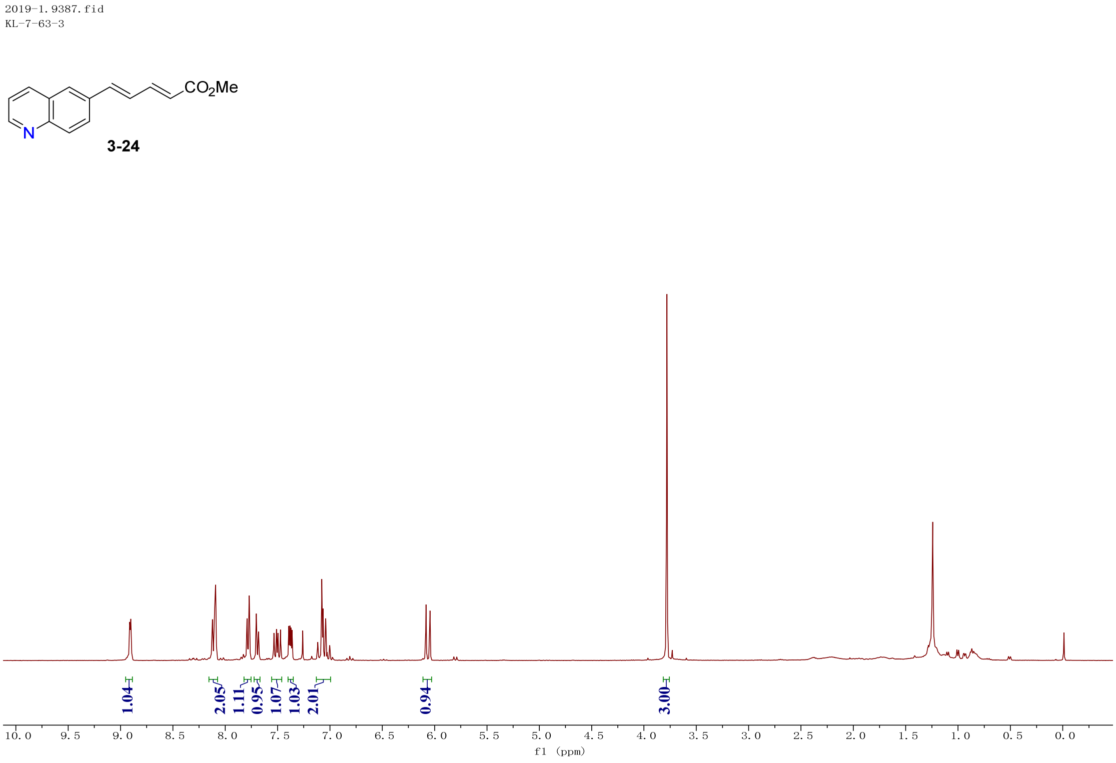

3-24

Supplementary Fig. 95

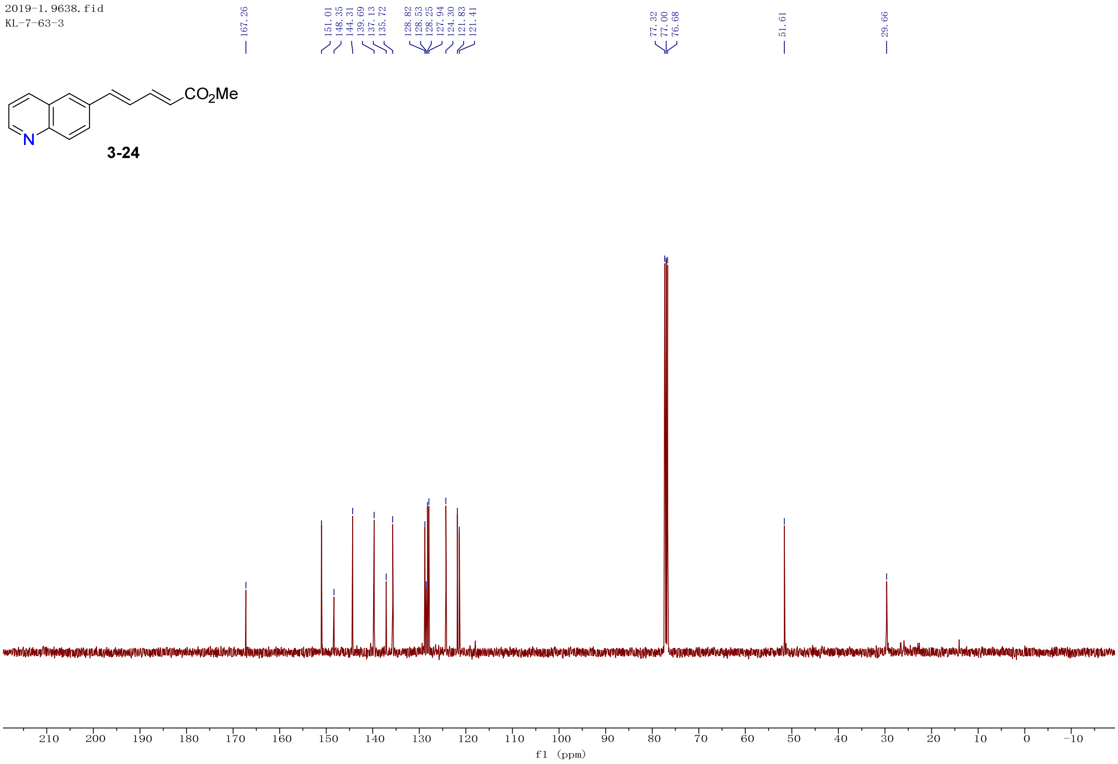

3-24

Supplementary Fig. 96

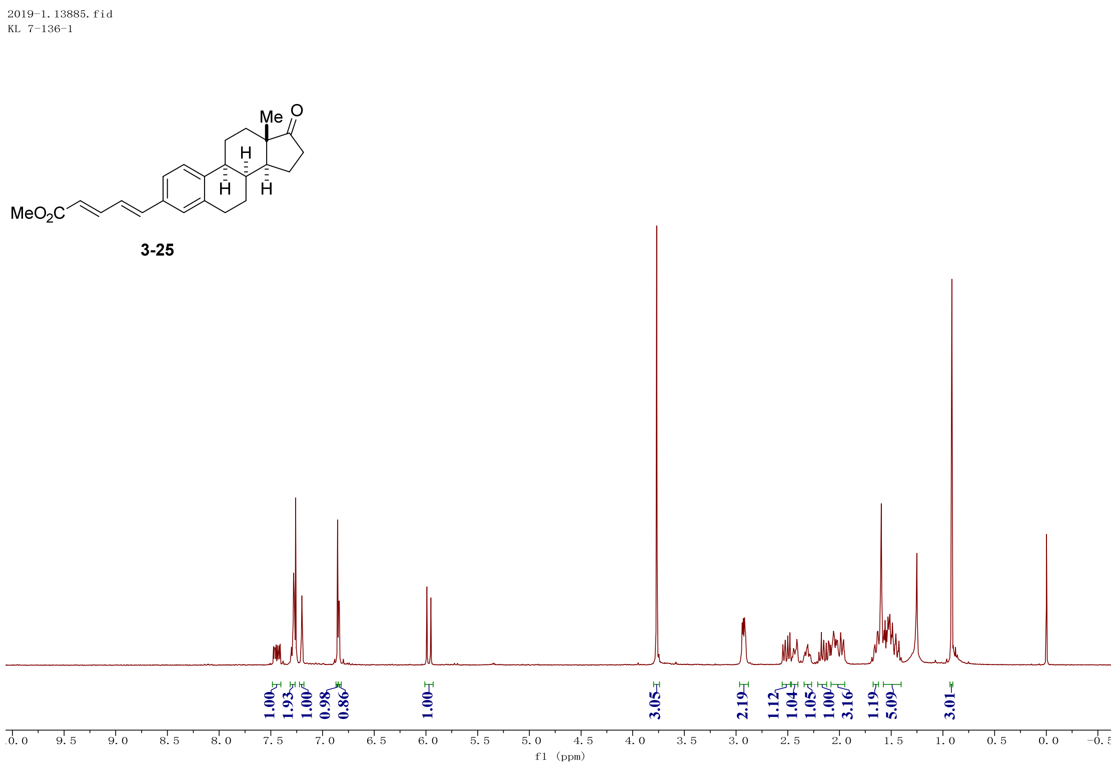

Supplementary Fig. 97

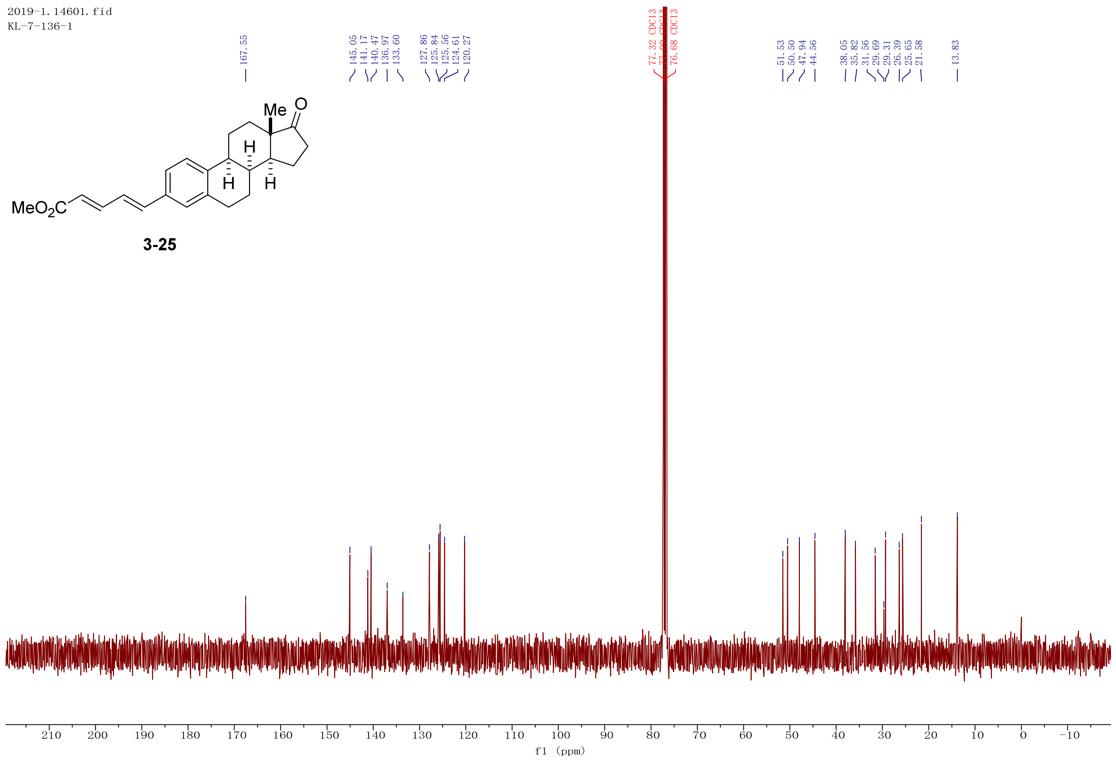

Supplementary Fig. 98

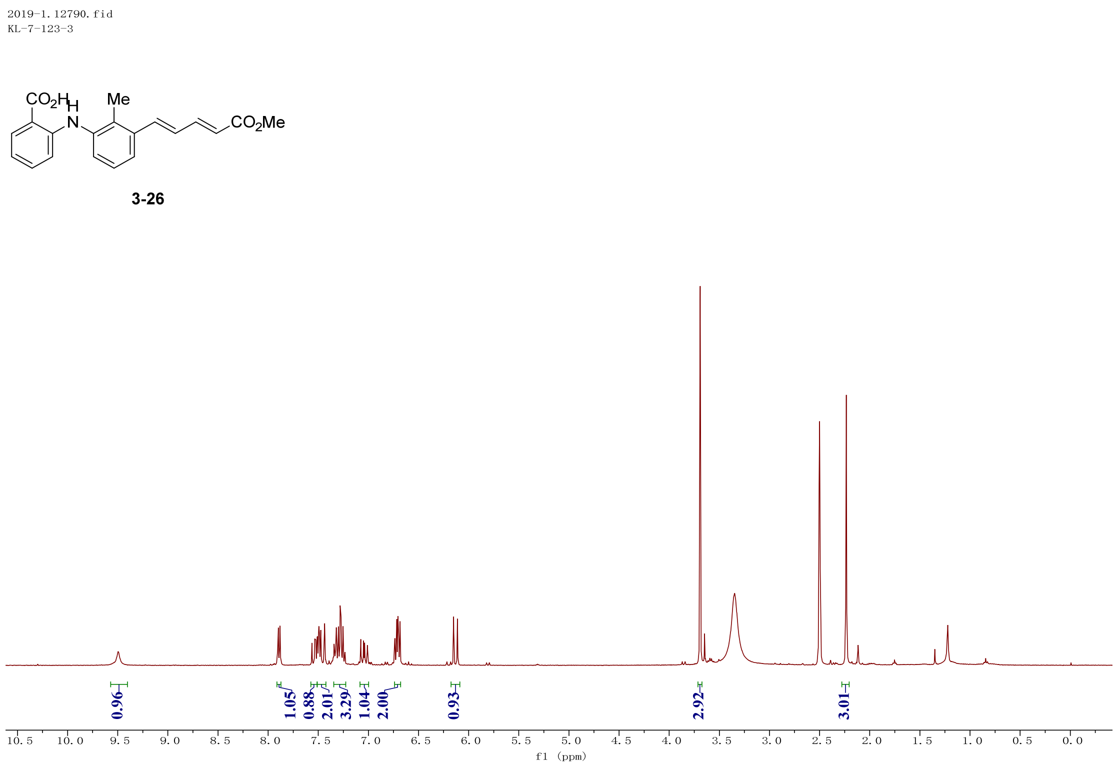

Supplementary Fig. 99

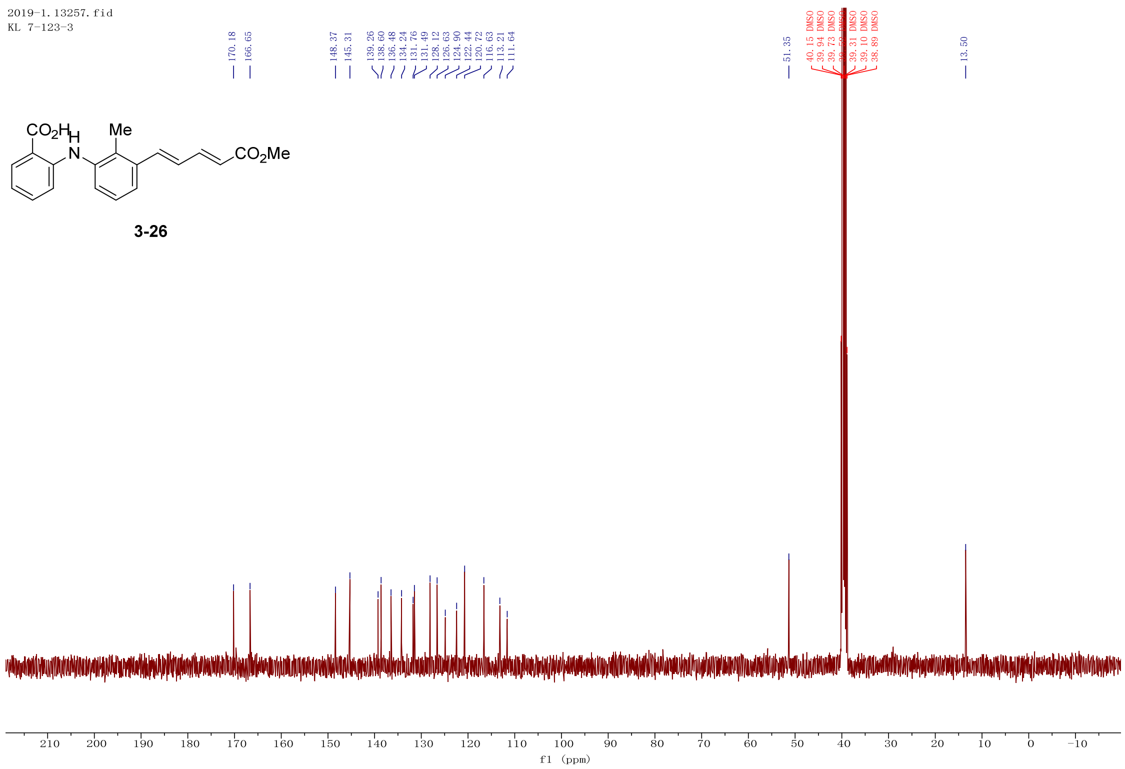

Supplementary Fig. 100

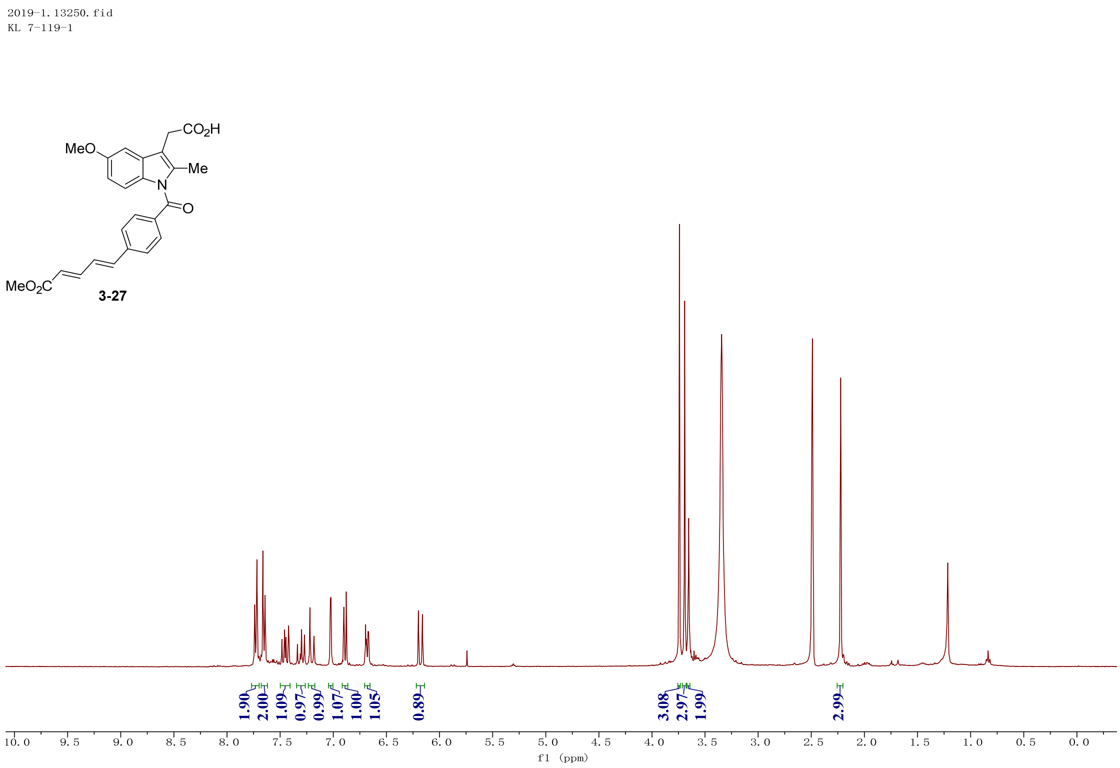

Supplementary Fig. 101

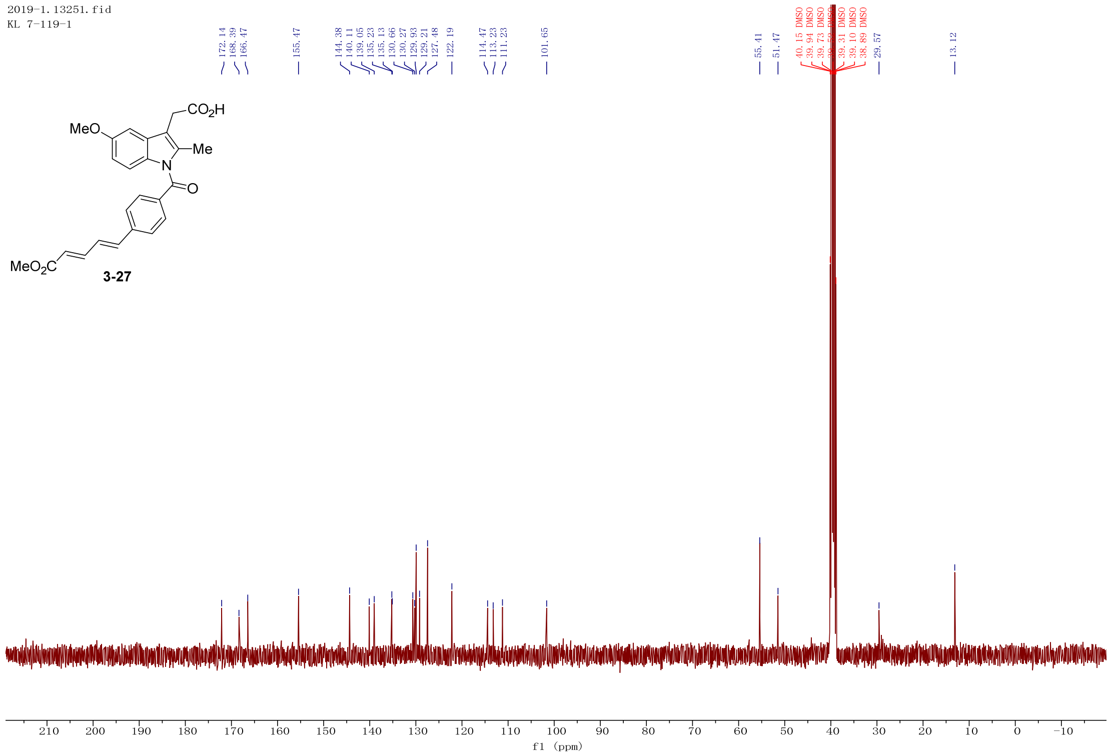

Supplementary Fig. 102

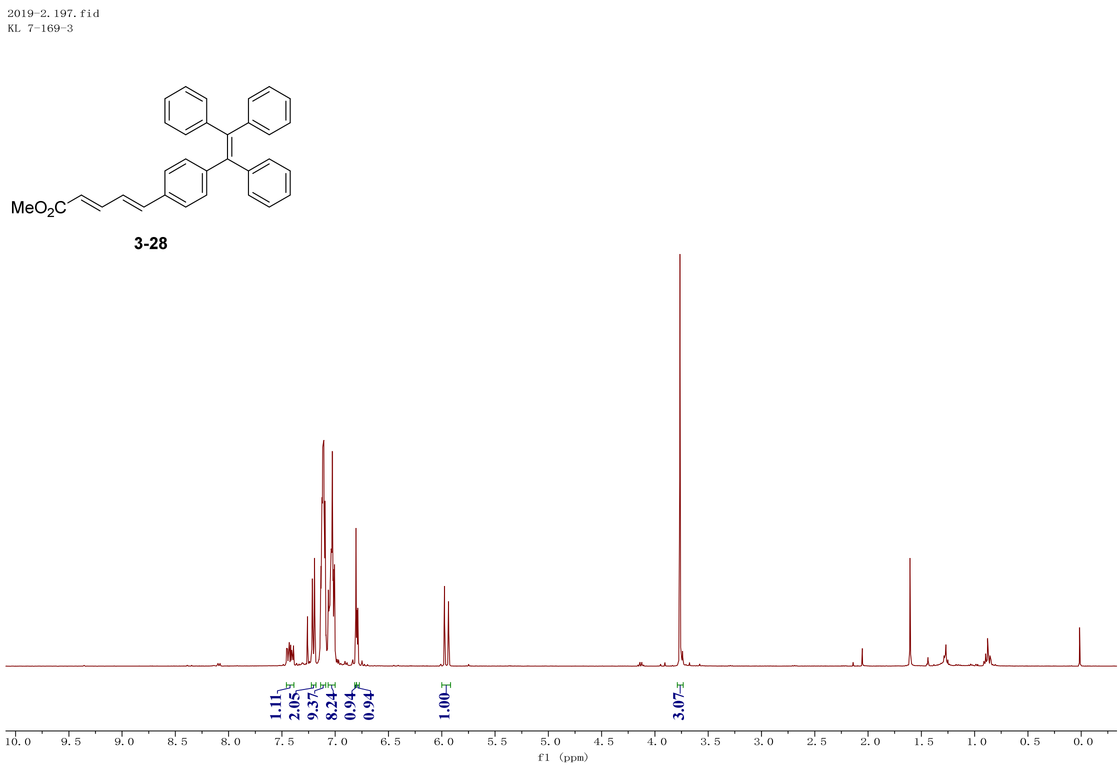

Supplementary Fig. 103

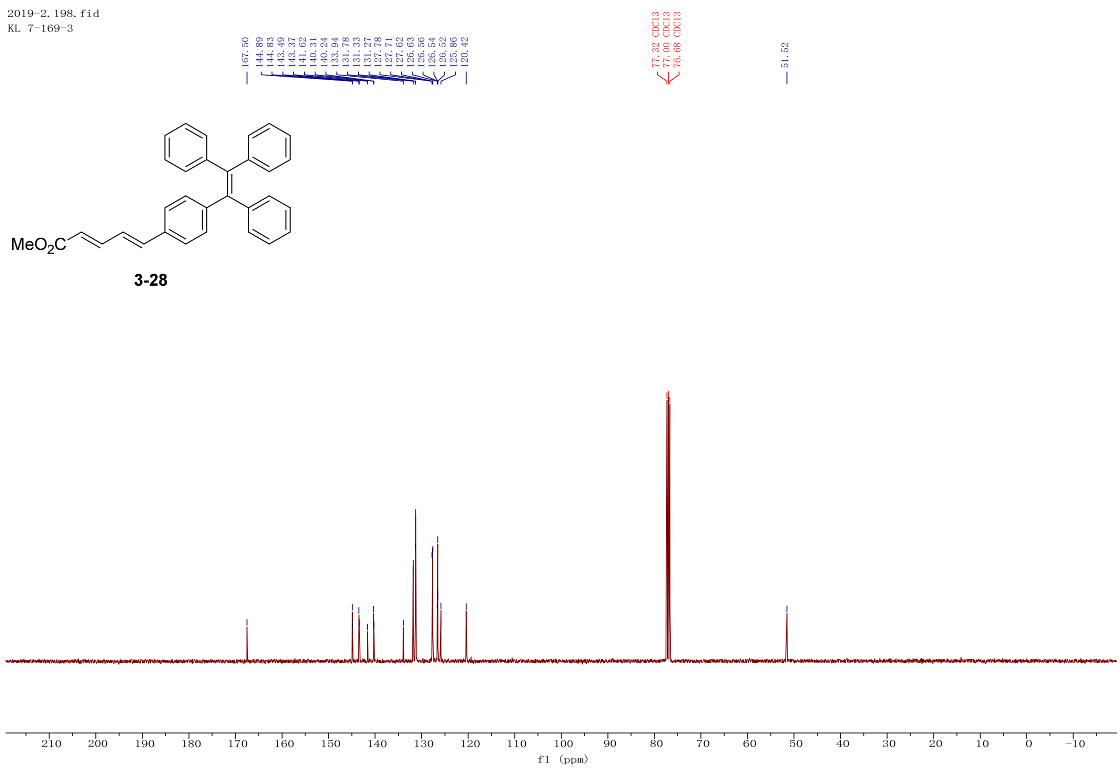

Supplementary Fig. 104

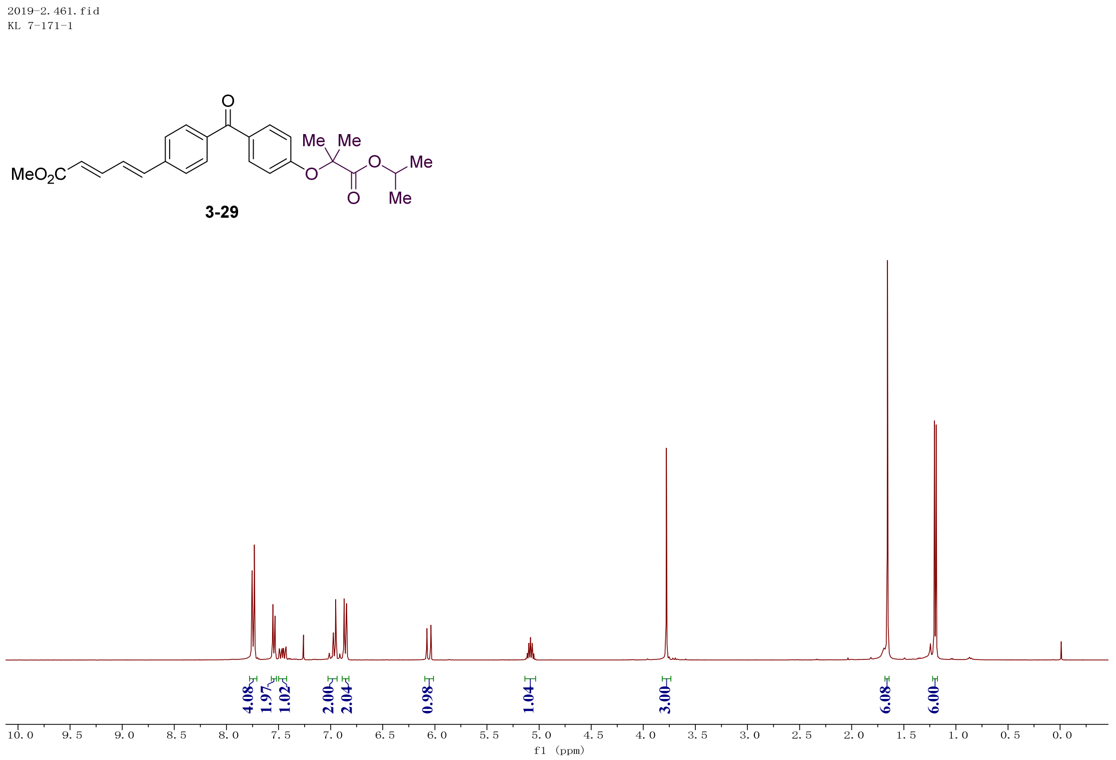

Supplementary Fig. 105

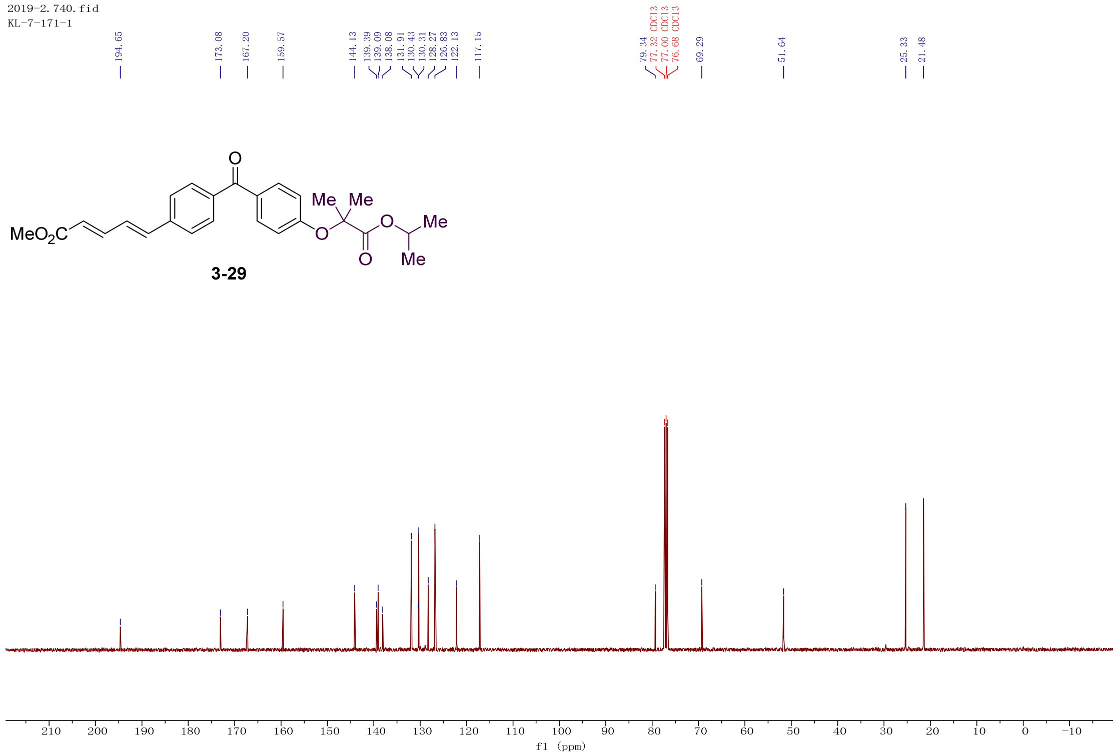

Supplementary Fig. 106

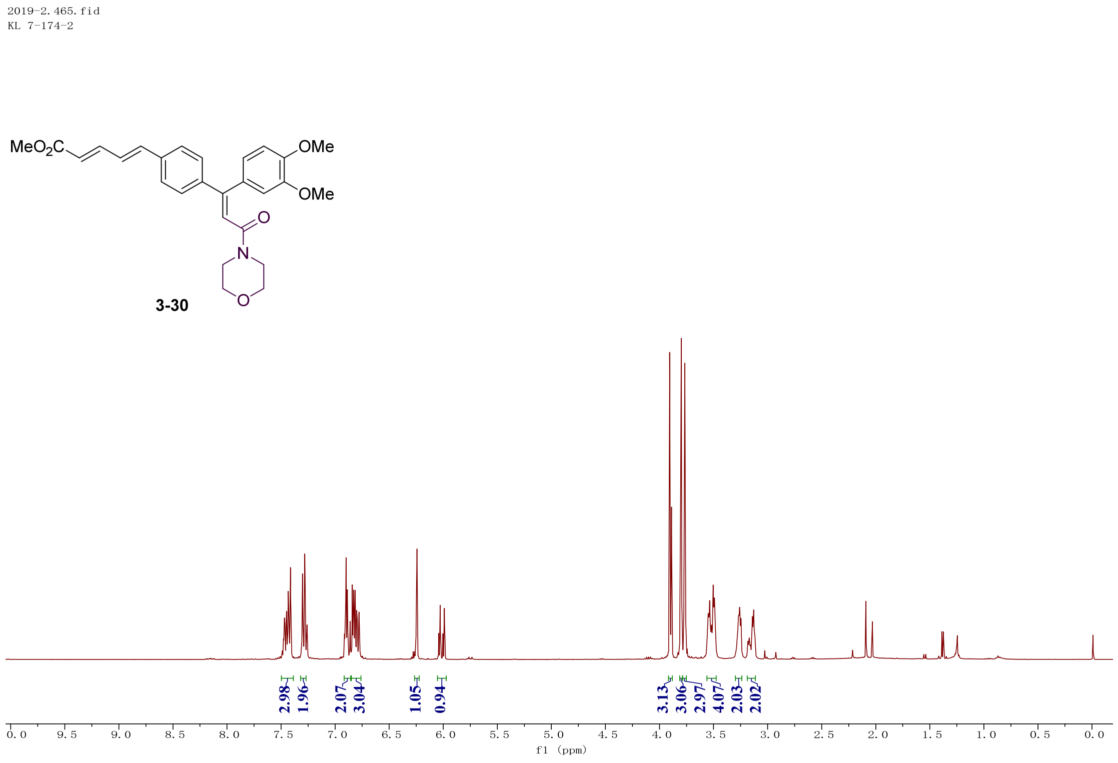

Supplementary Fig. 107

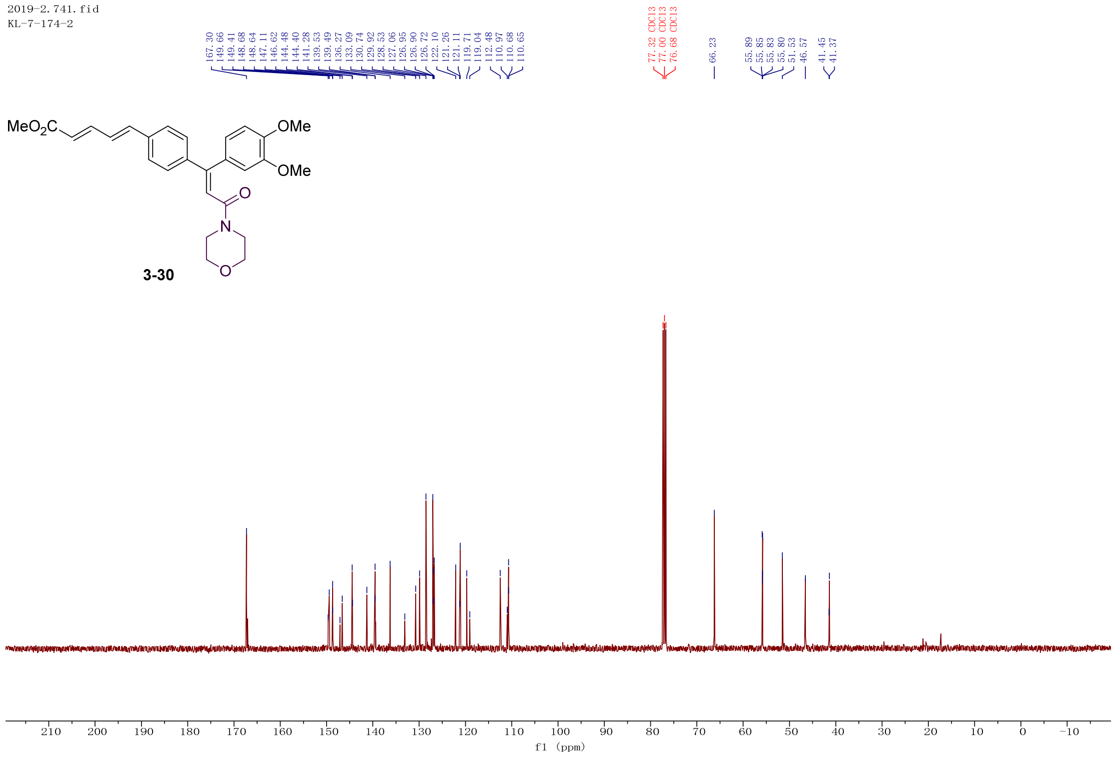

Supplementary Fig. 108

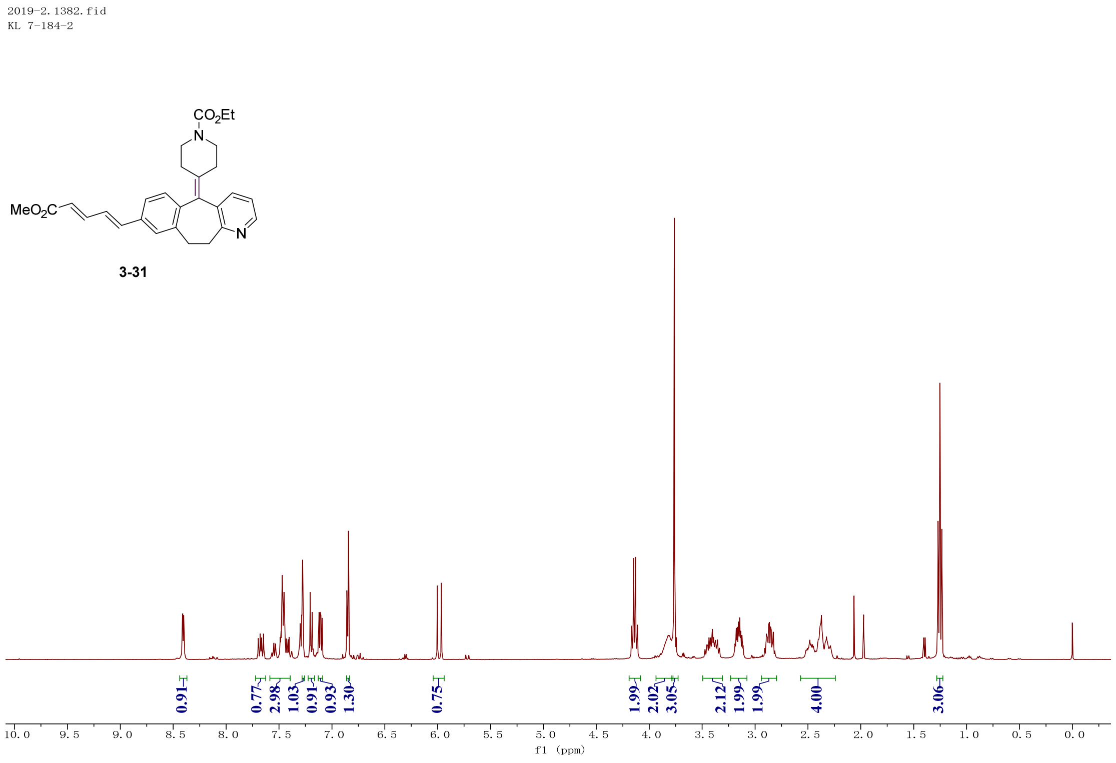

Supplementary Fig. 109

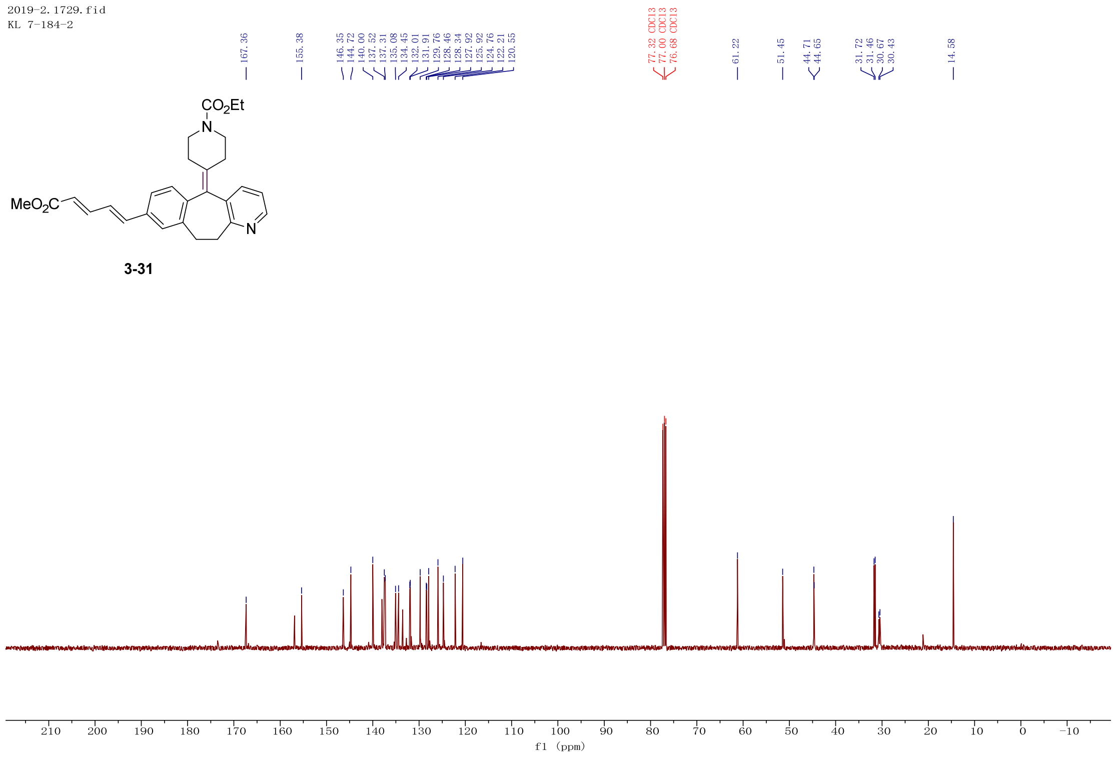

Supplementary Fig. 110

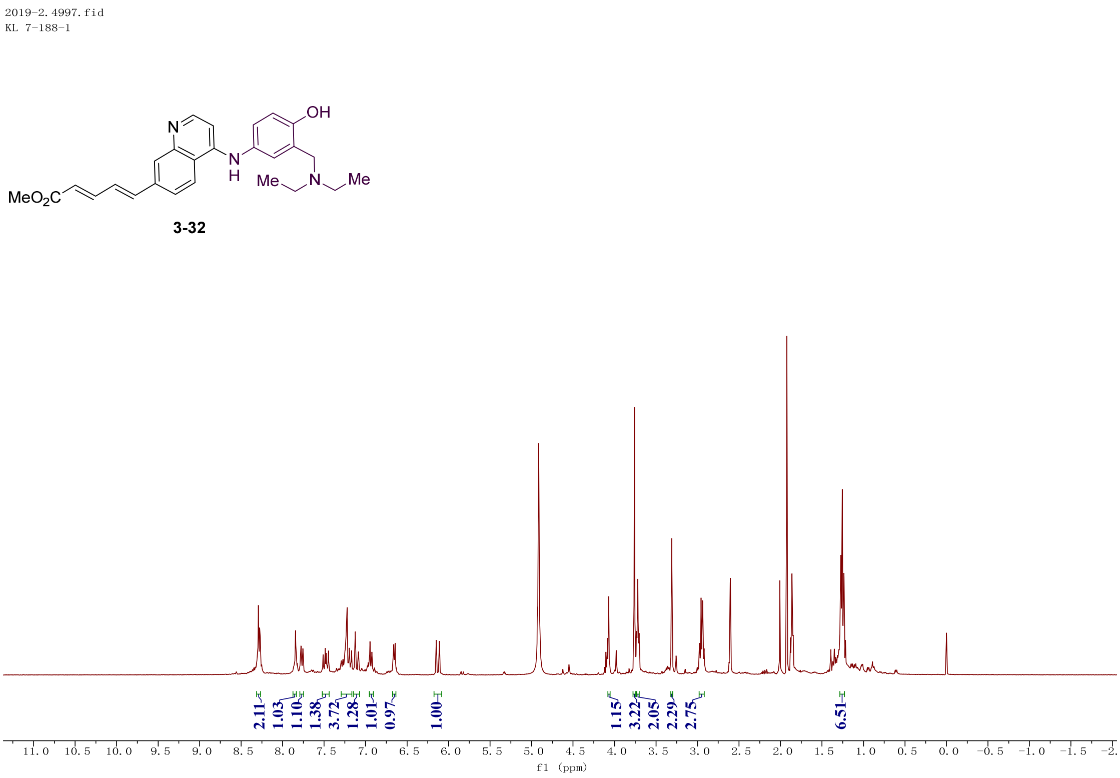

Supplementary Fig. 111

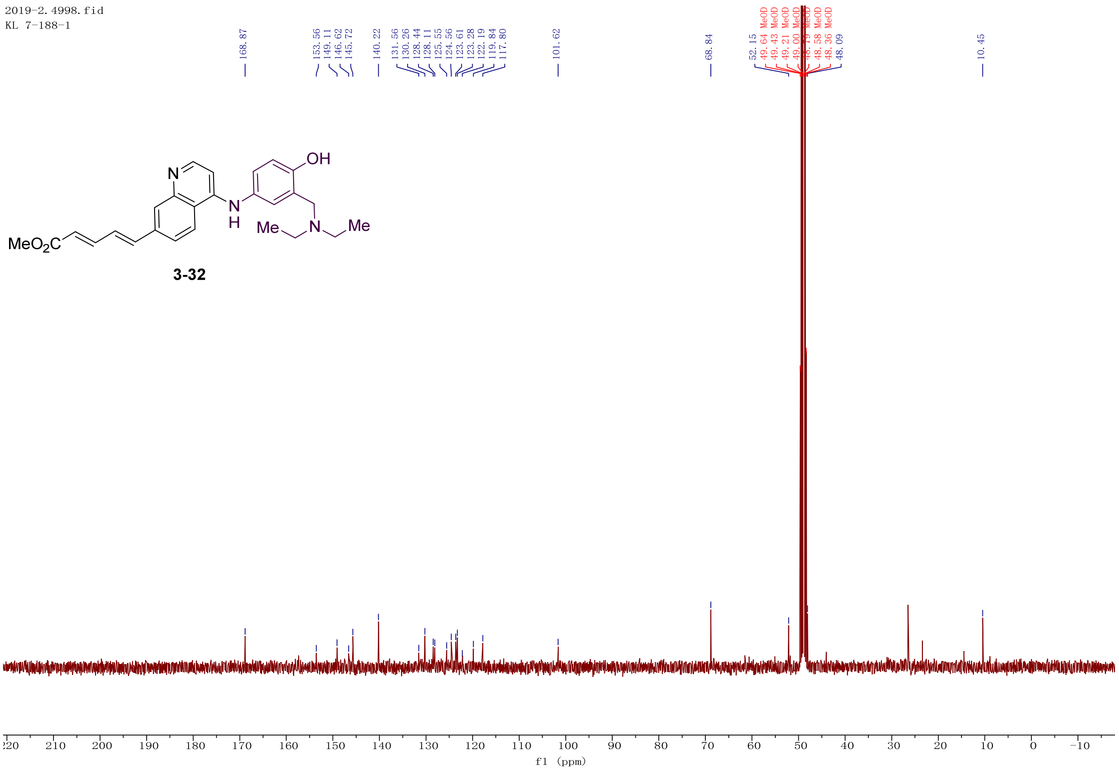

Supplementary Fig. 112

2019-2.457.fid
KL-7-170-2

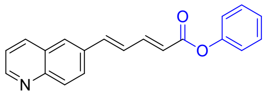

3-33

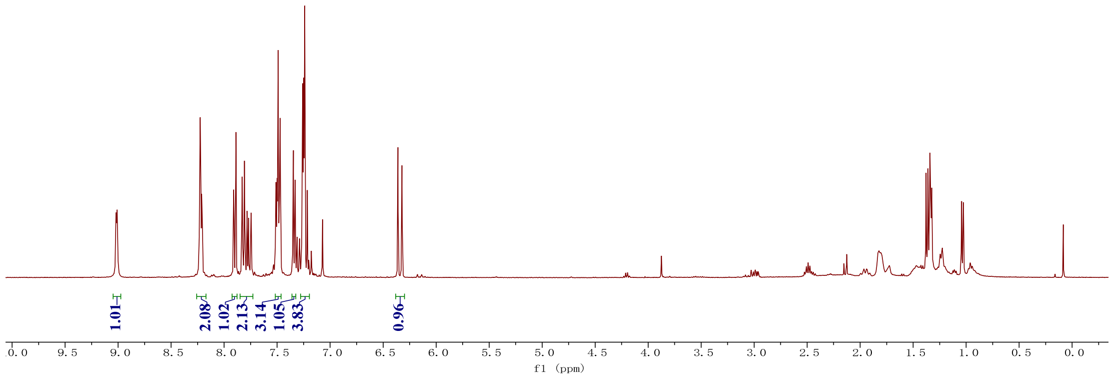

Supplementary Fig. 113

2019-2.742.fid
KL-7-170-2

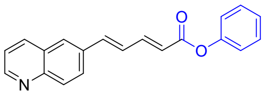

3-33

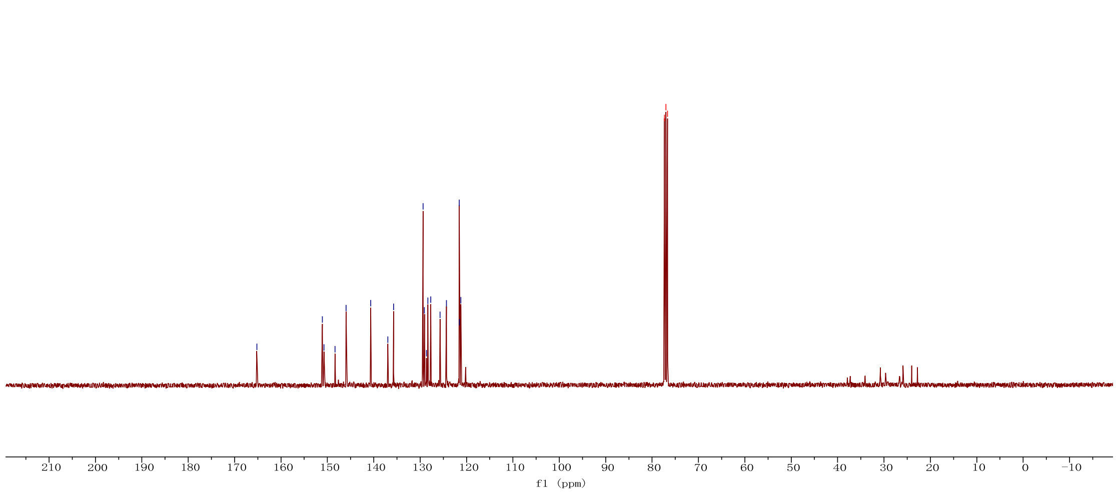

Supplementary Fig. 114

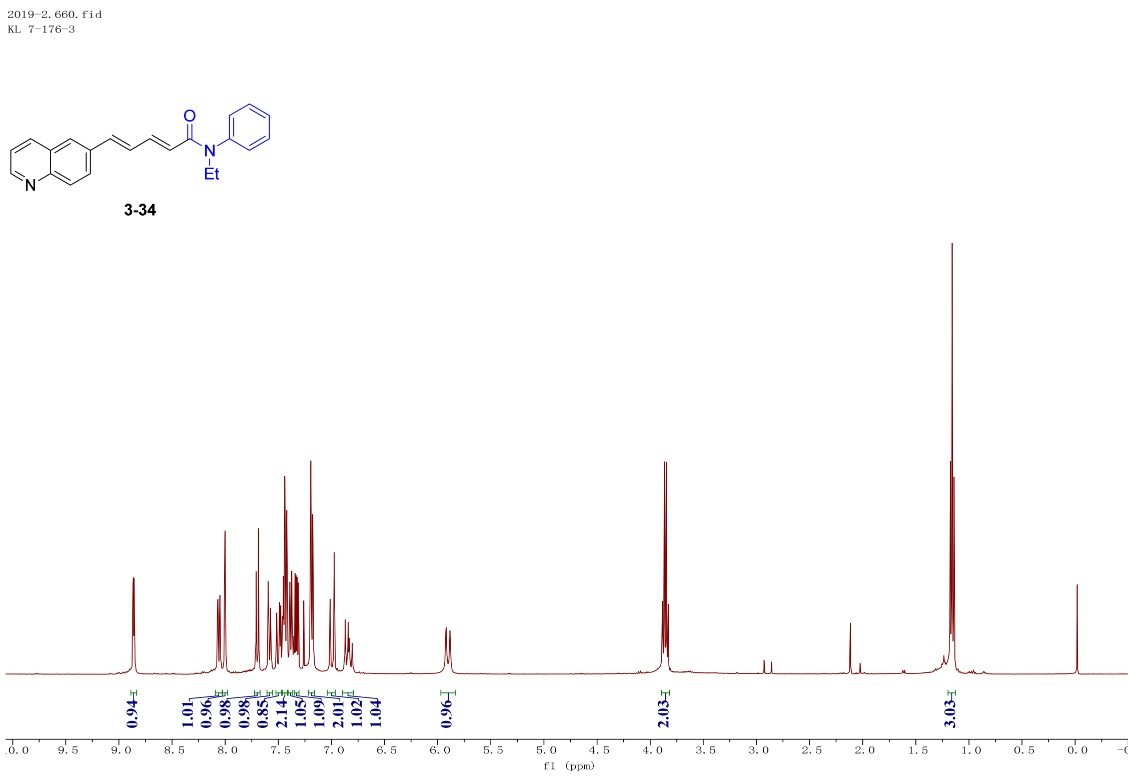

Supplementary Fig. 115

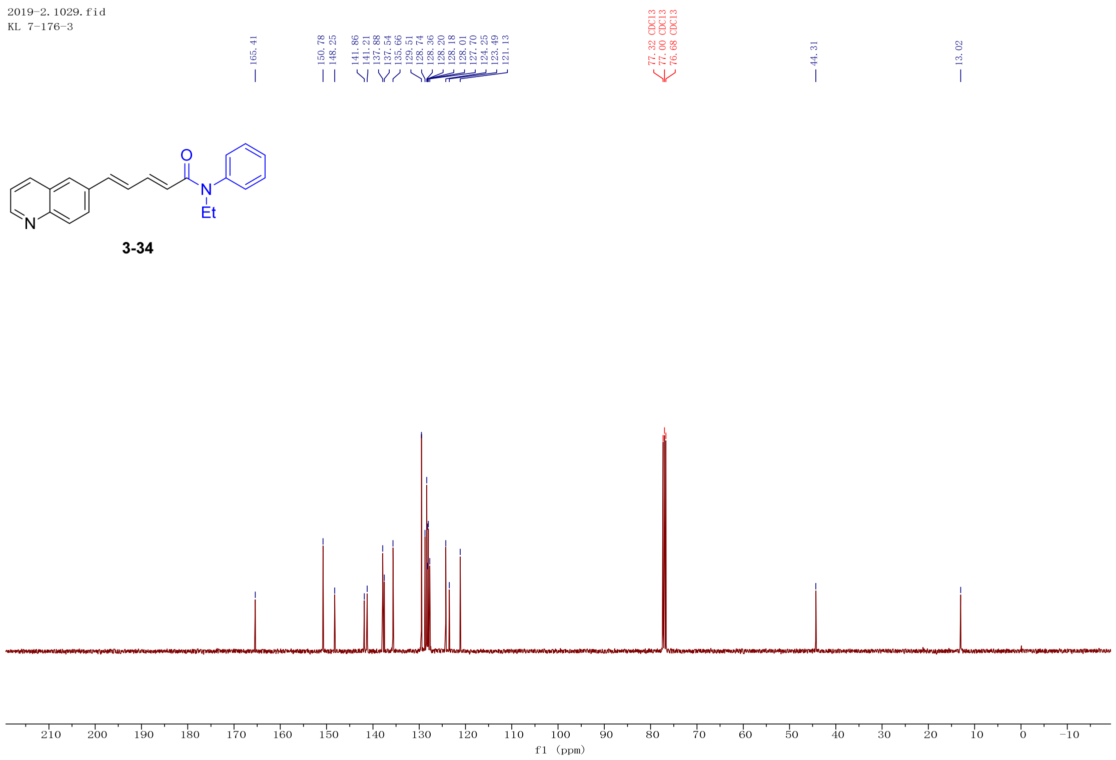

Supplementary Fig. 116

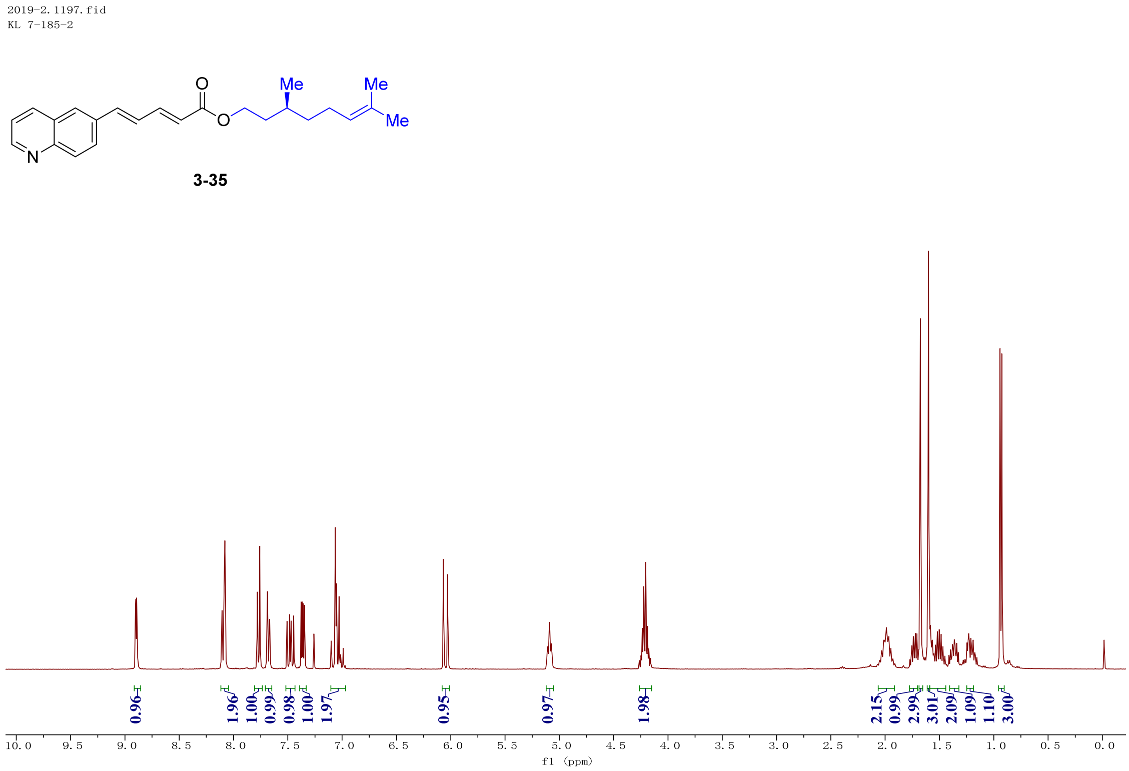

Supplementary Fig. 117

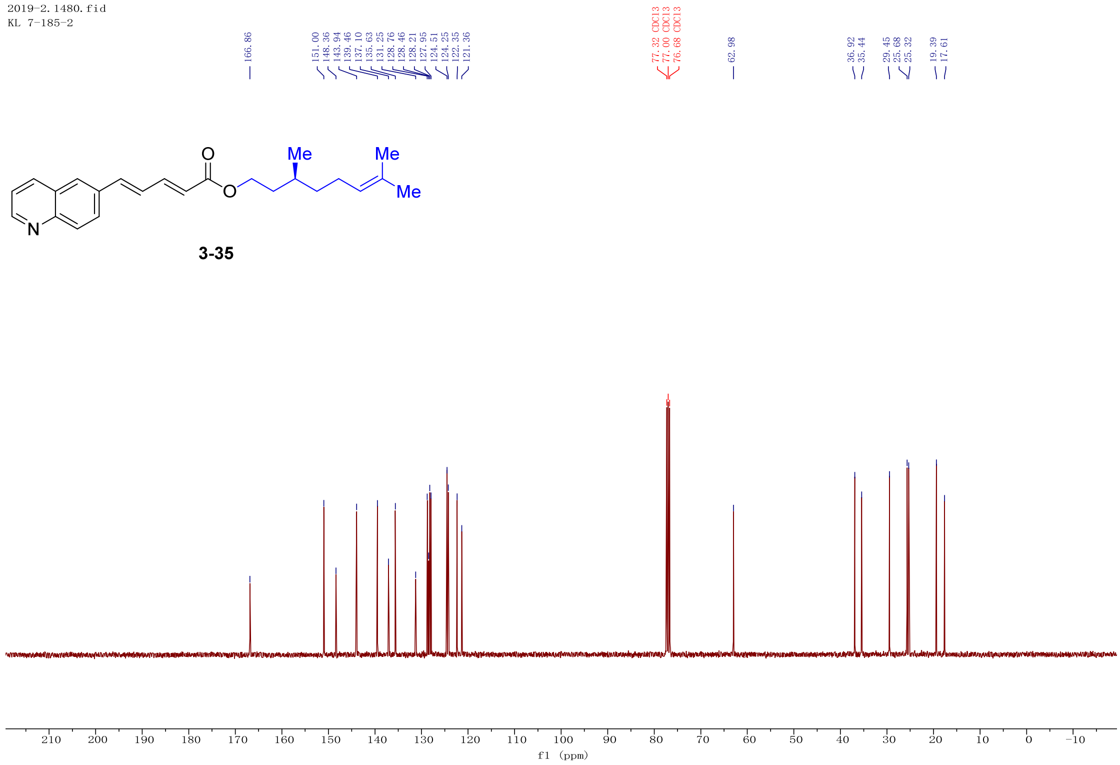

Supplementary Fig. 118

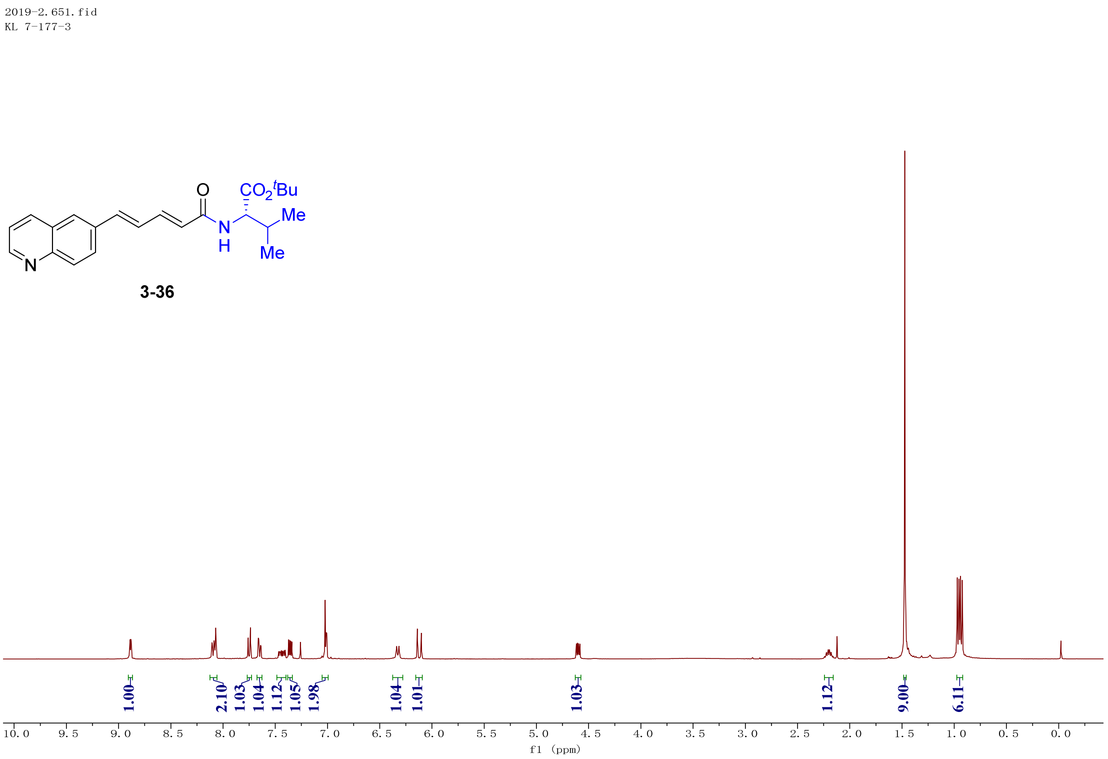

Supplementary Fig. 119

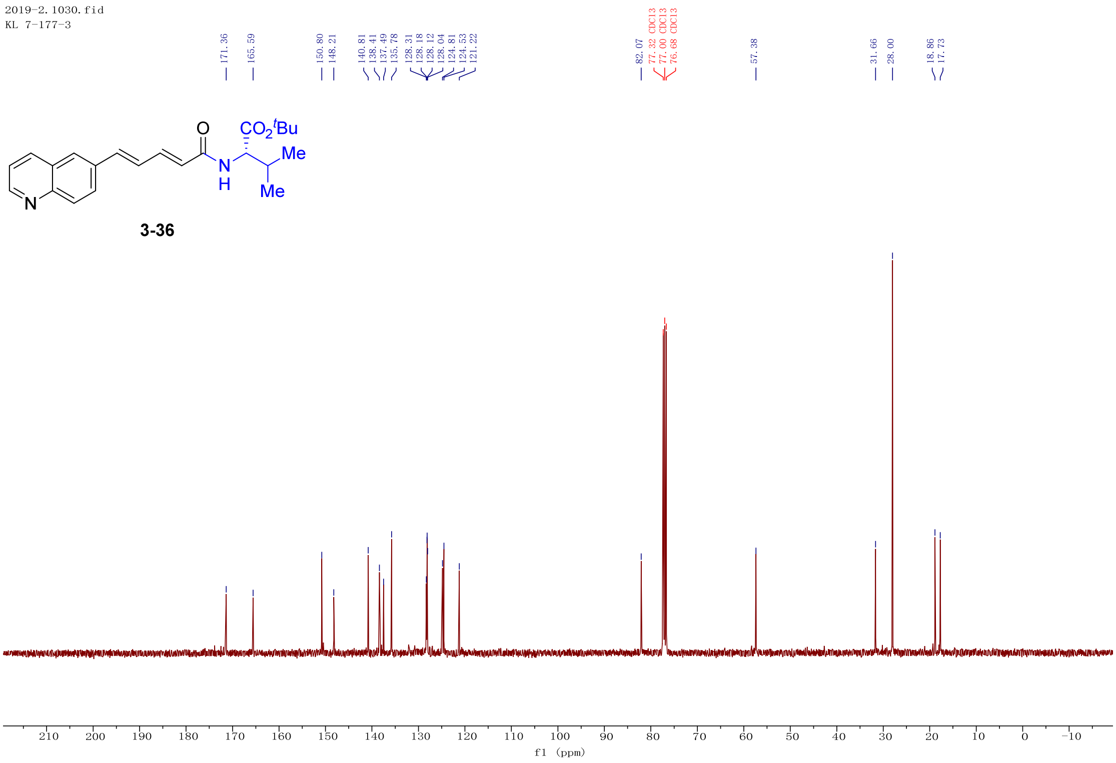

Supplementary Fig. 120

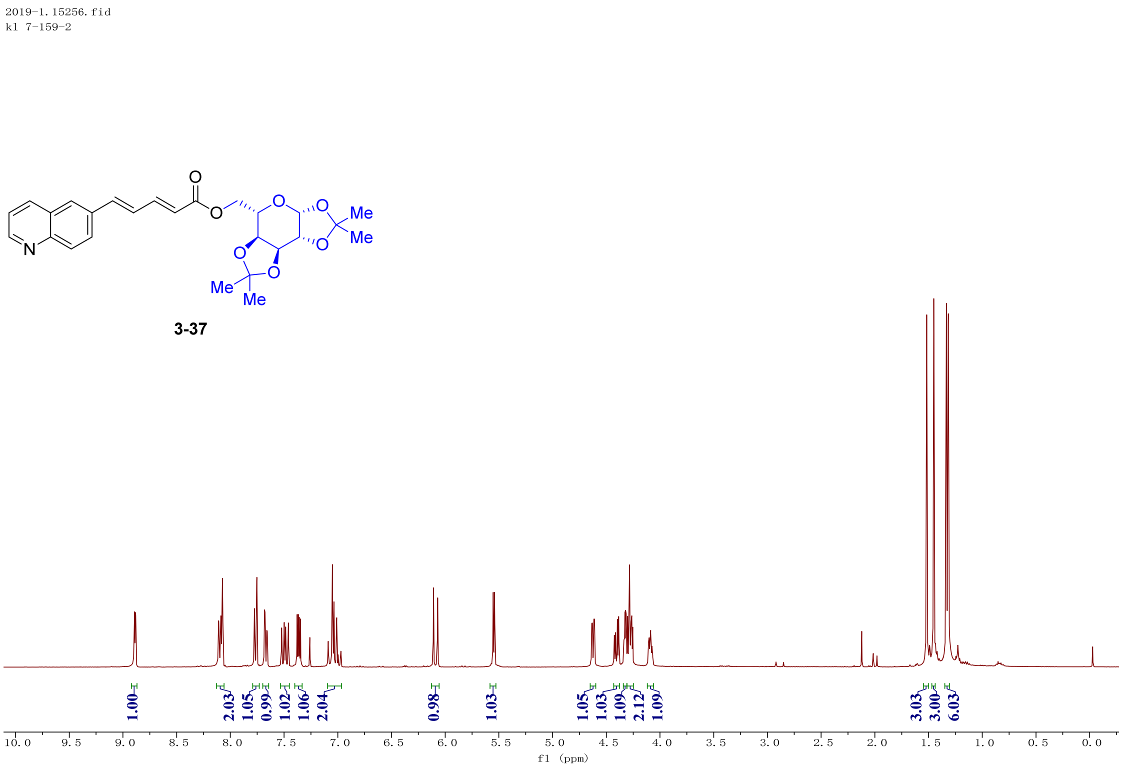

Supplementary Fig. 121

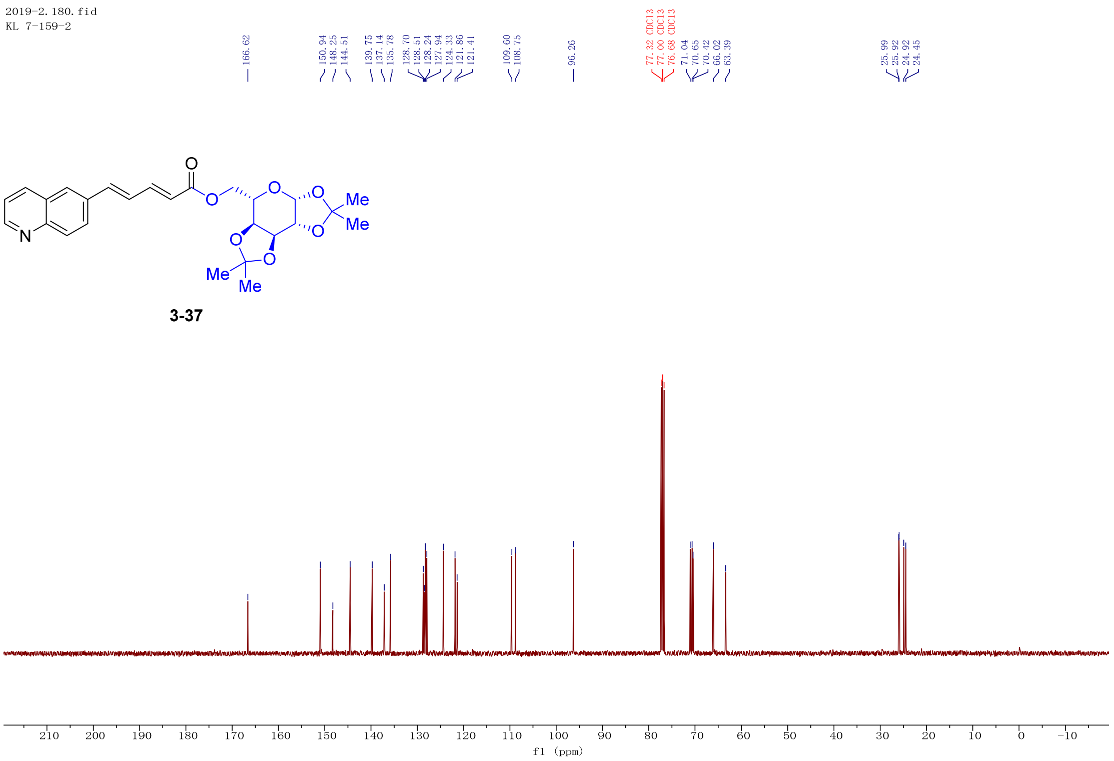

Supplementary Fig. 122

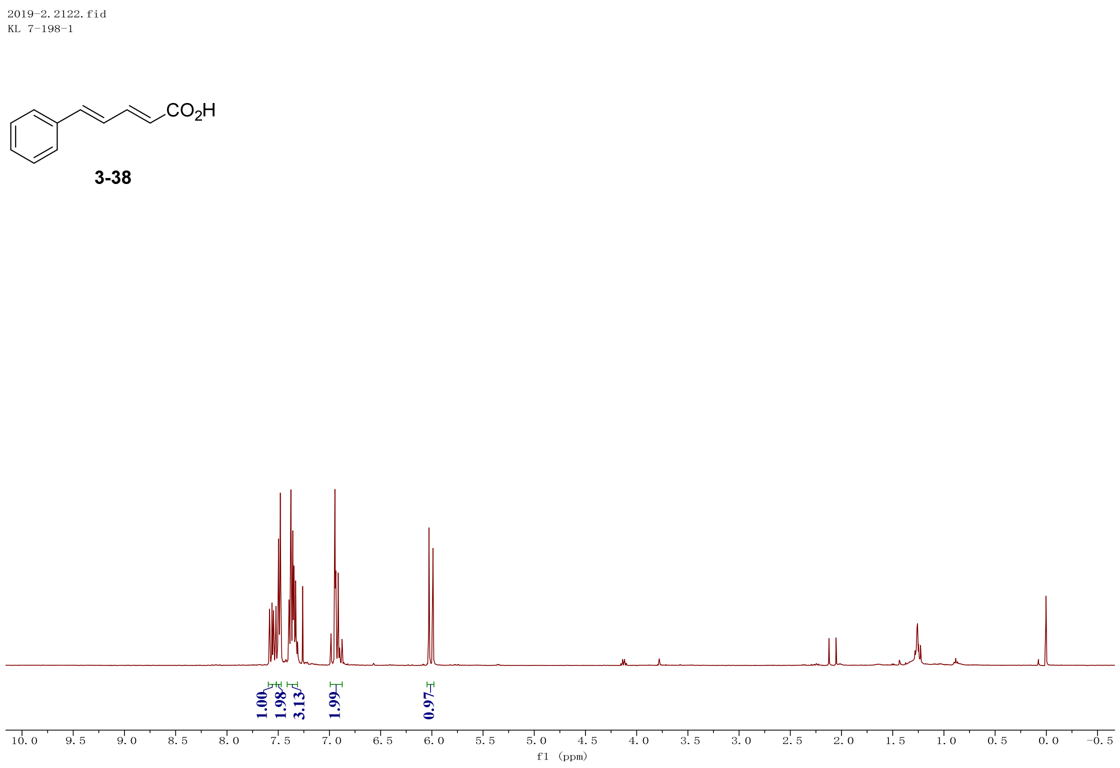

Supplementary Fig. 123

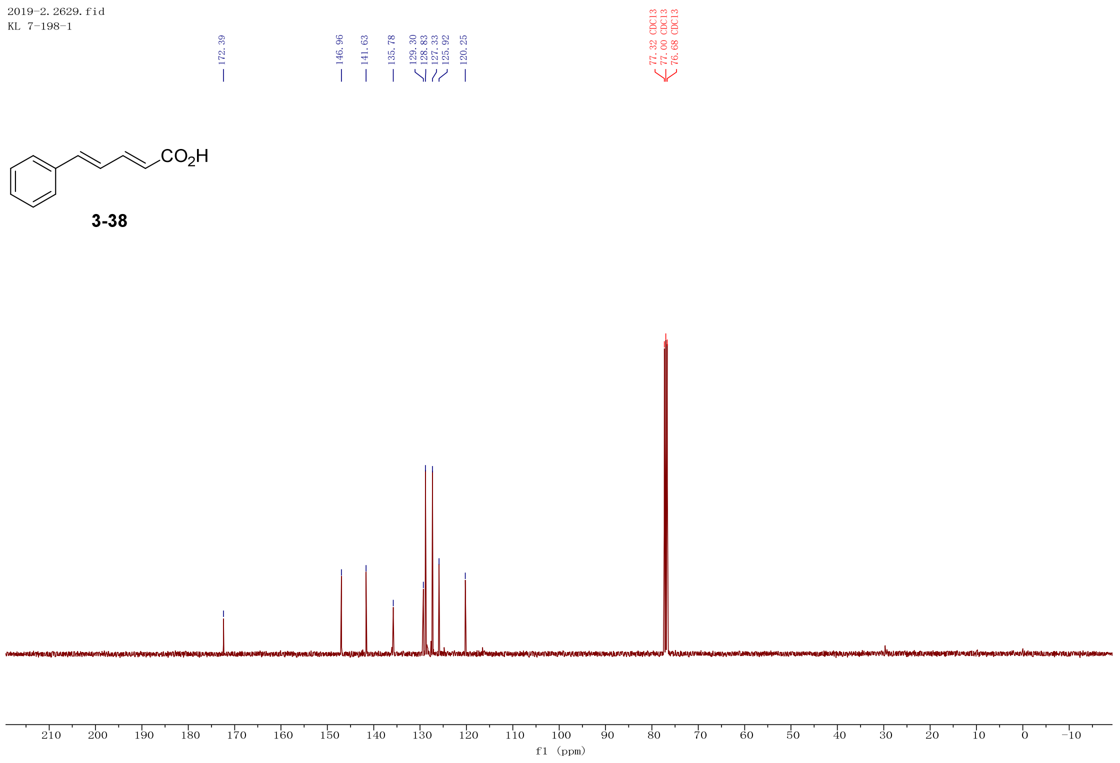

Supplementary Fig. 124

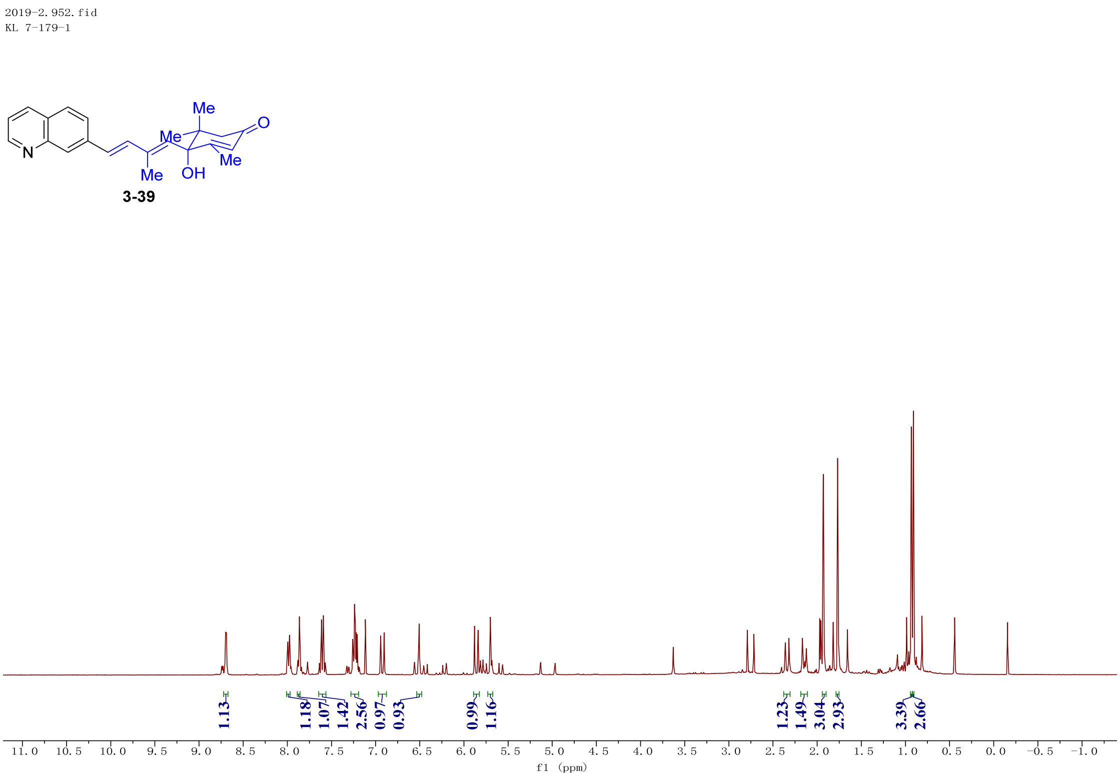

Supplementary Fig. 125

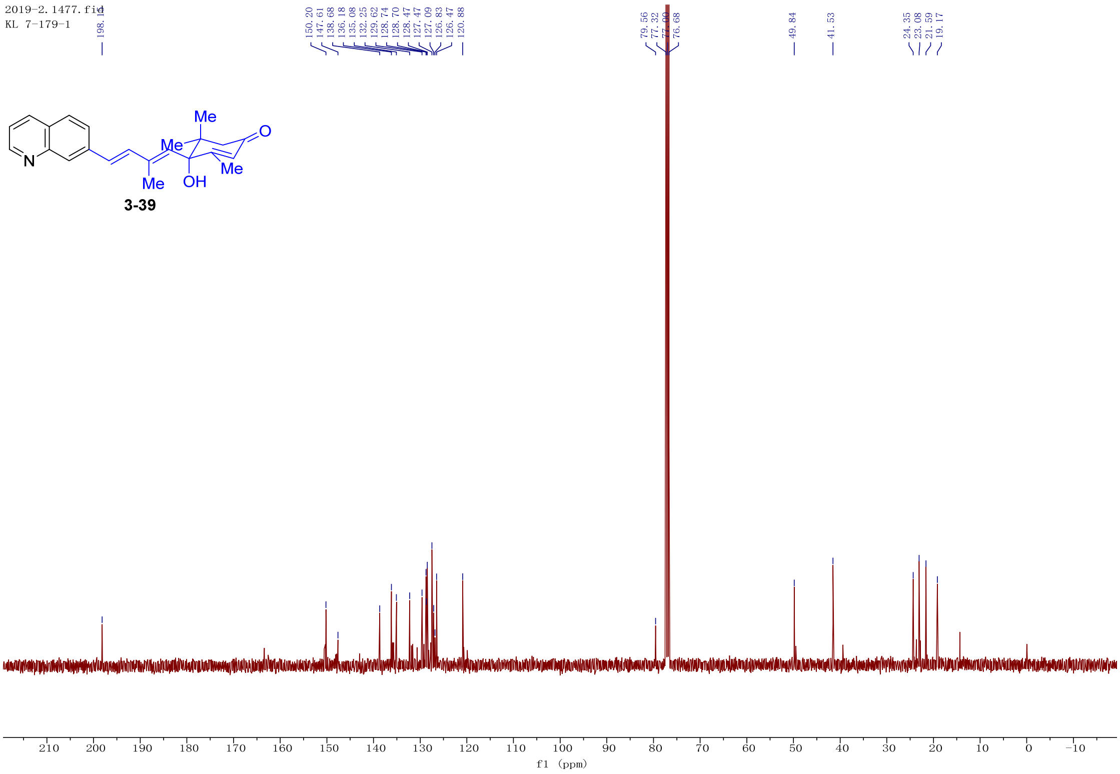

Supplementary Fig. 126

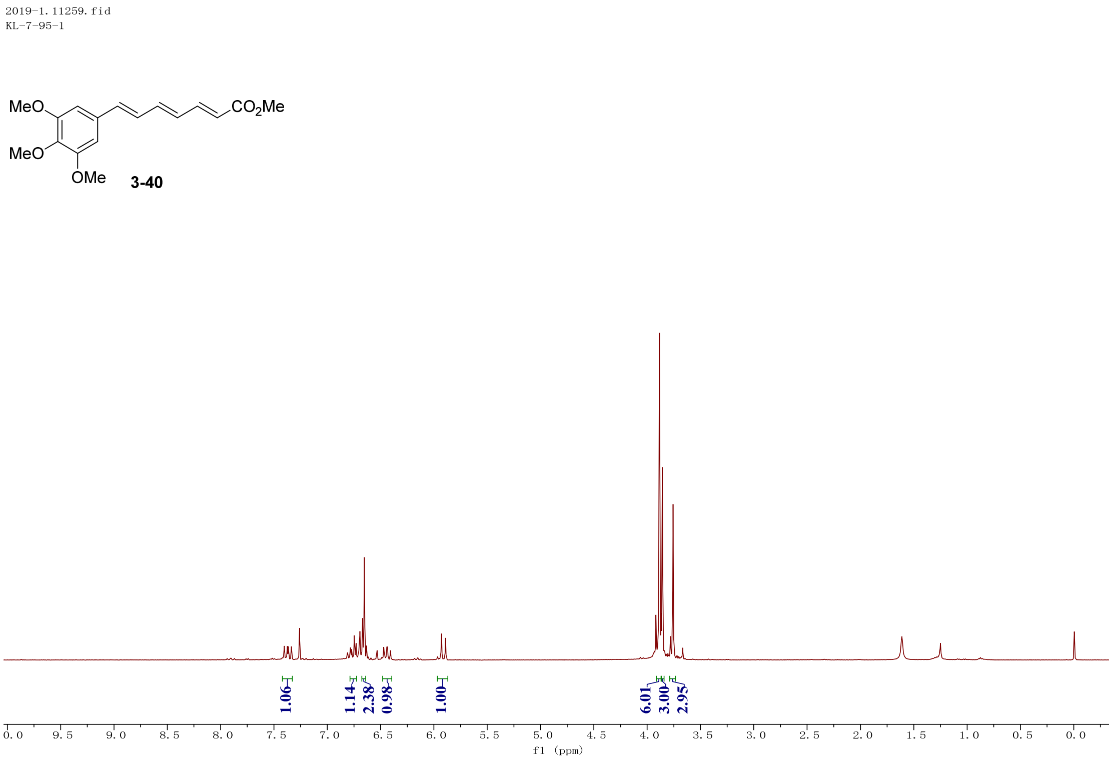

Supplementary Fig. 127

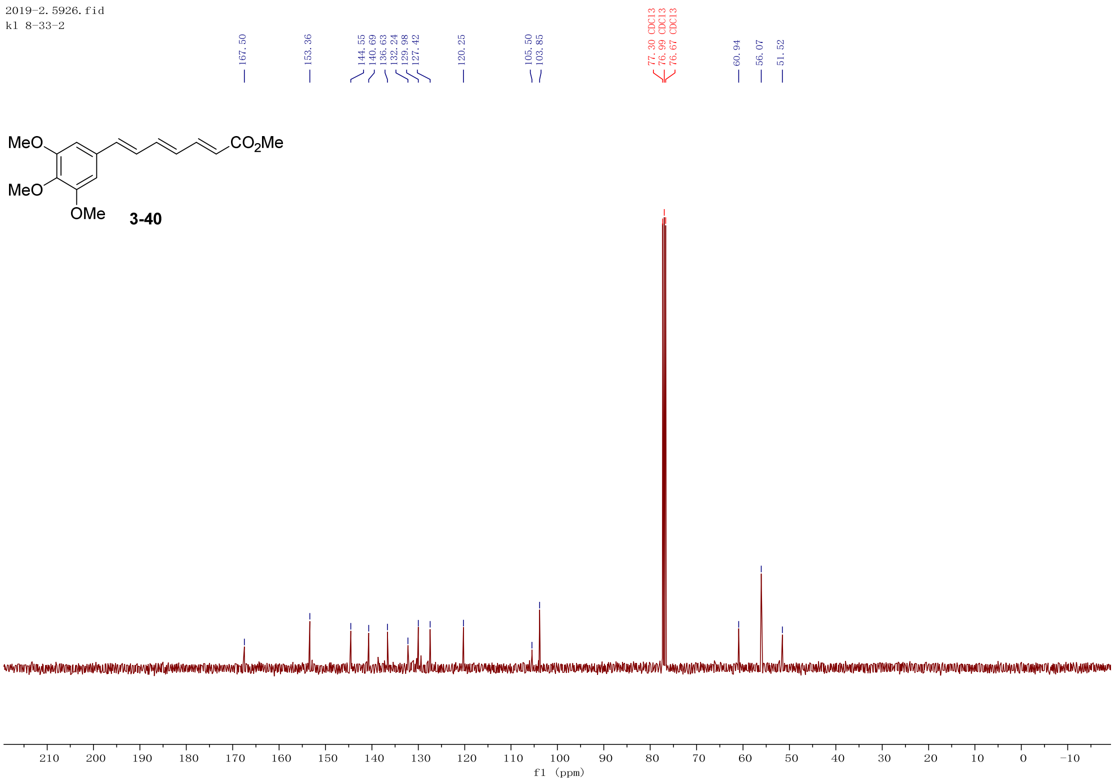

Supplementary Fig. 128

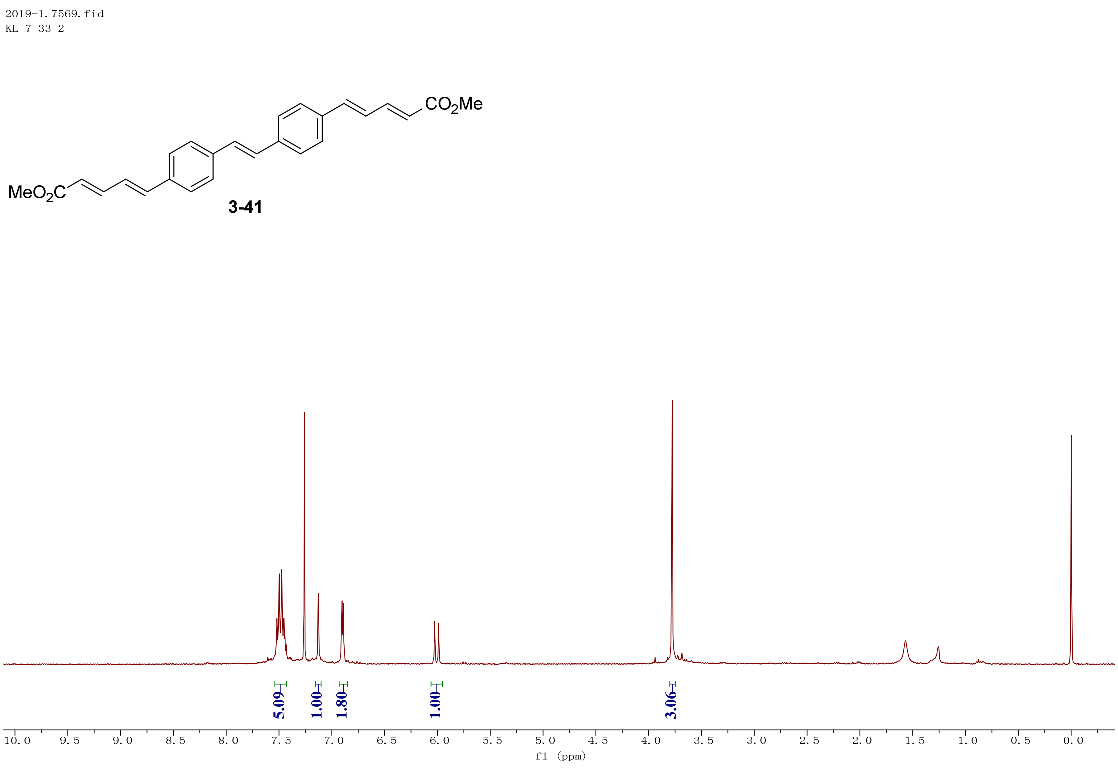

Supplementary Fig. 129

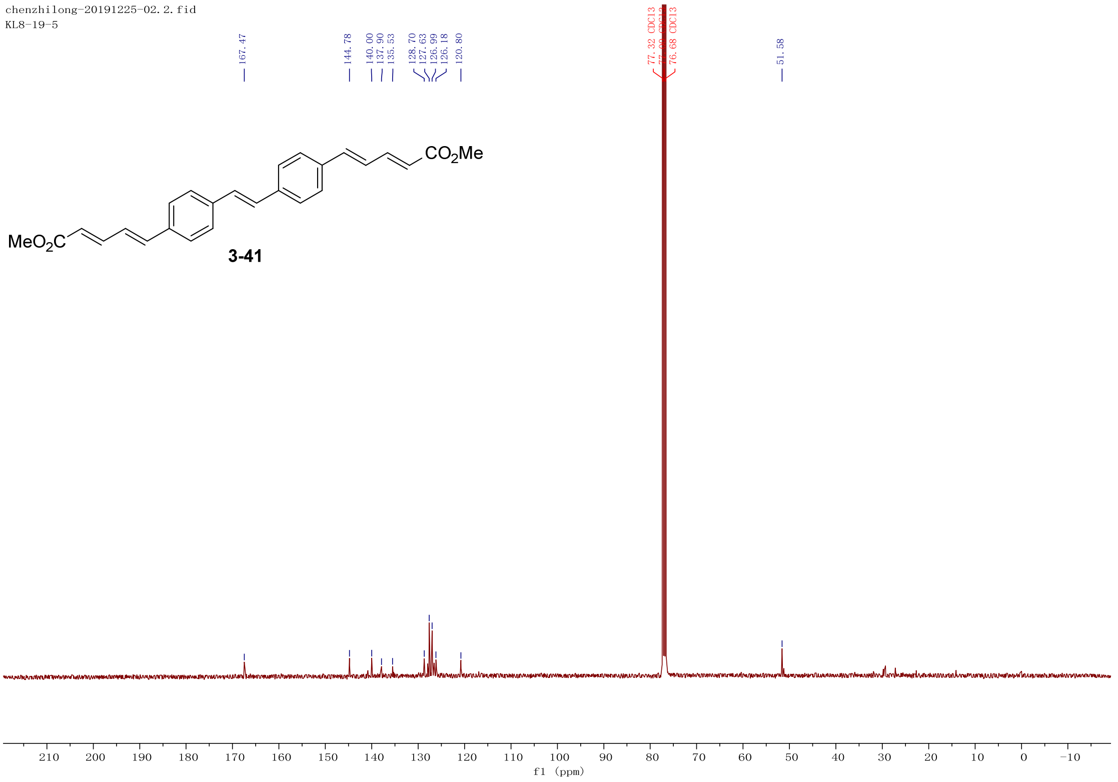

Supplementary Fig. 130

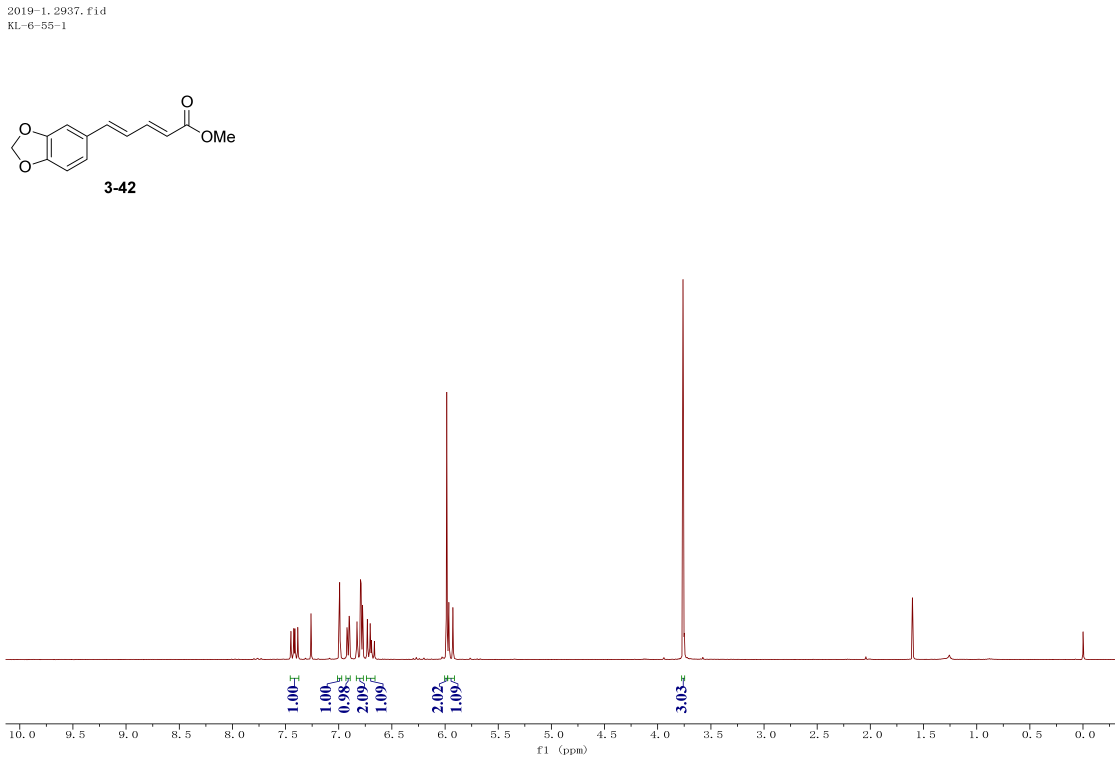

Supplementary Fig. 131

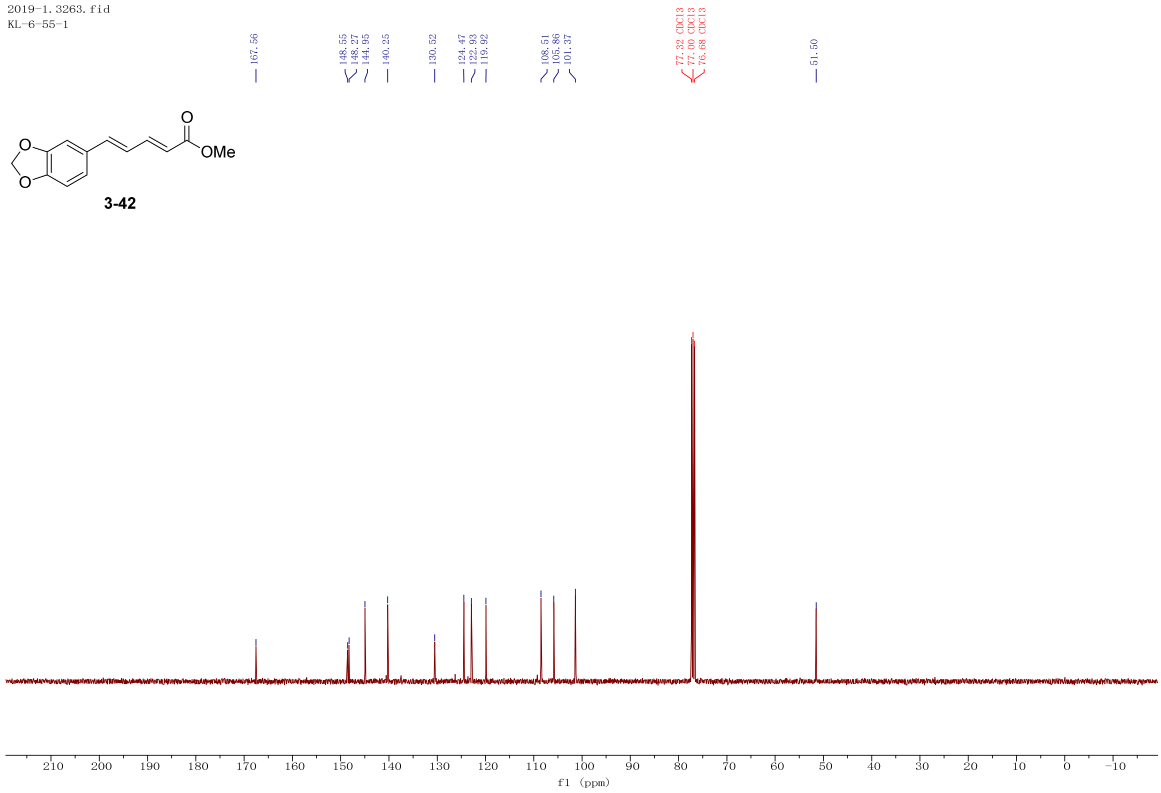

Supplementary Fig. 132

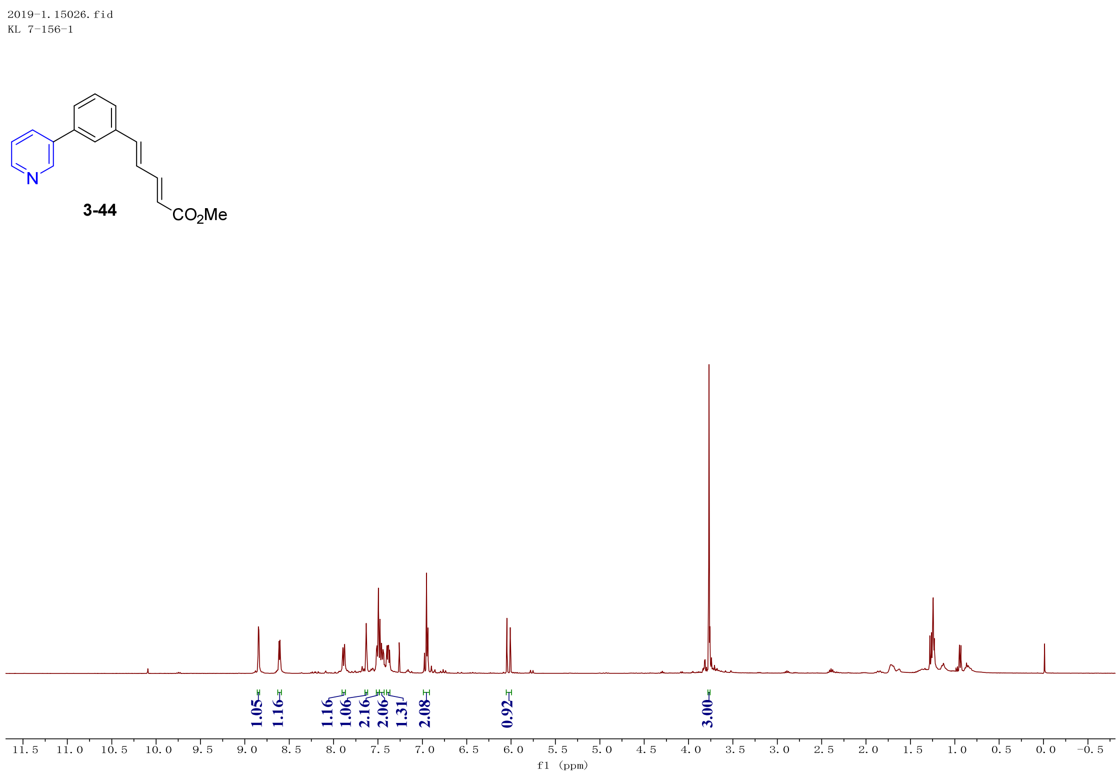

Supplementary Fig. 133

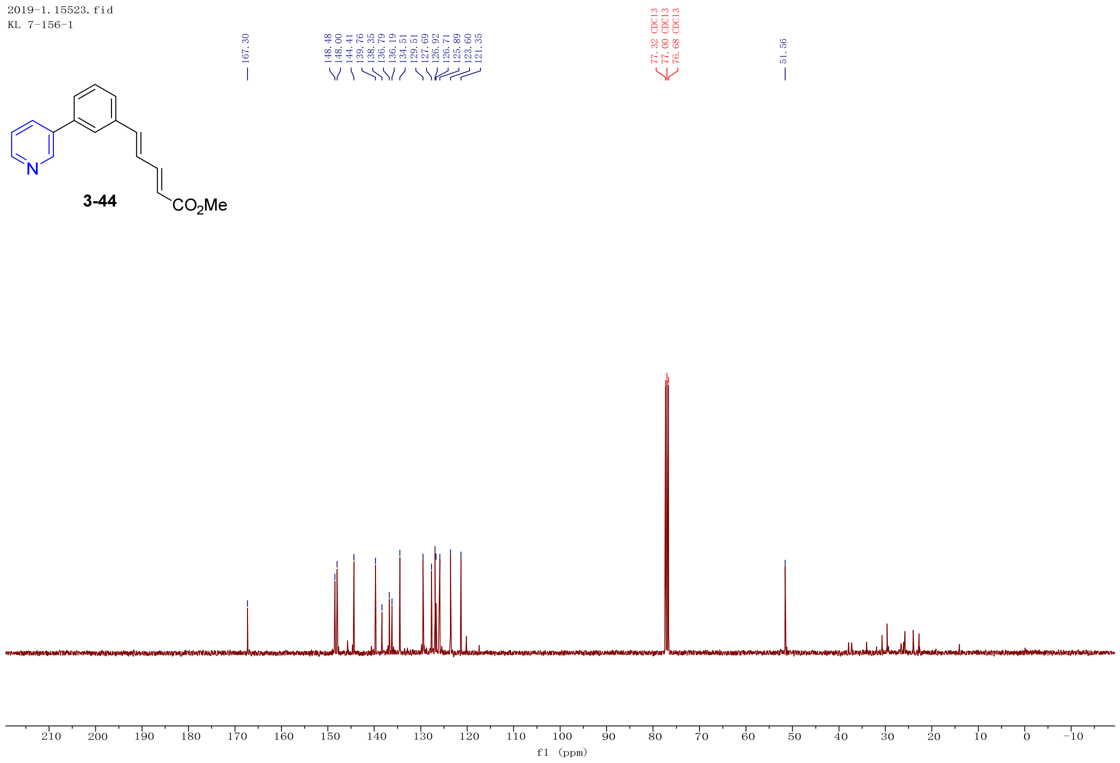

Supplementary Fig. 134

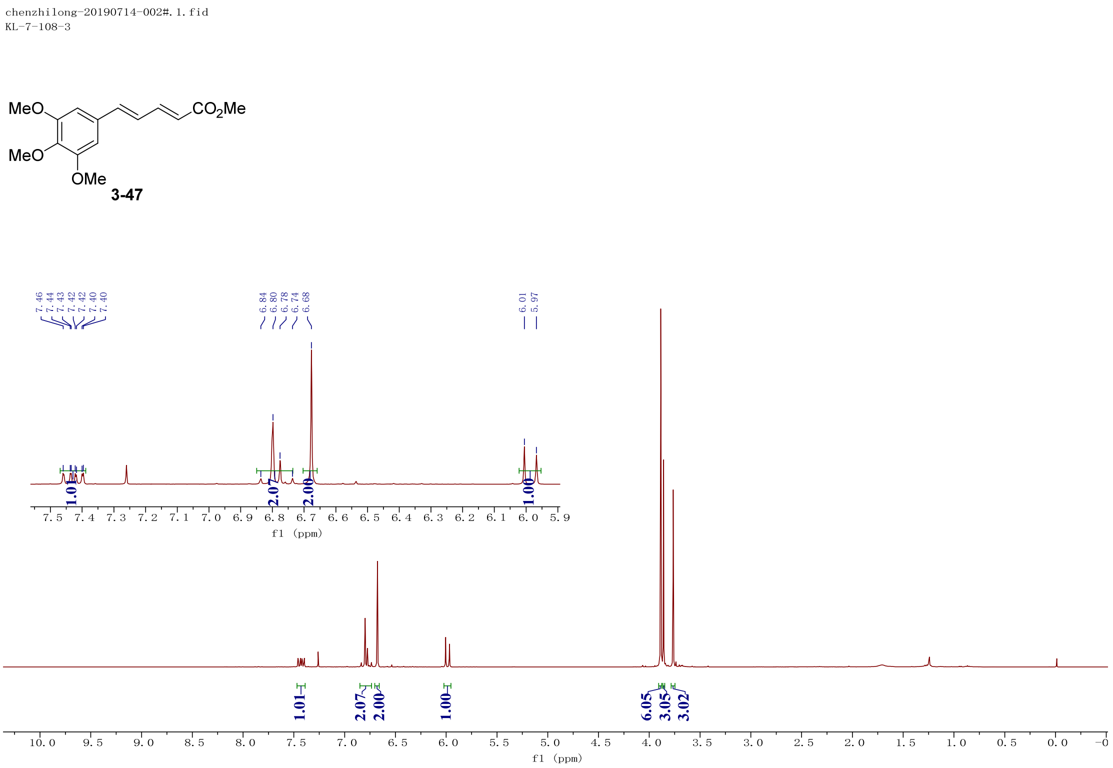

Supplementary Fig. 135

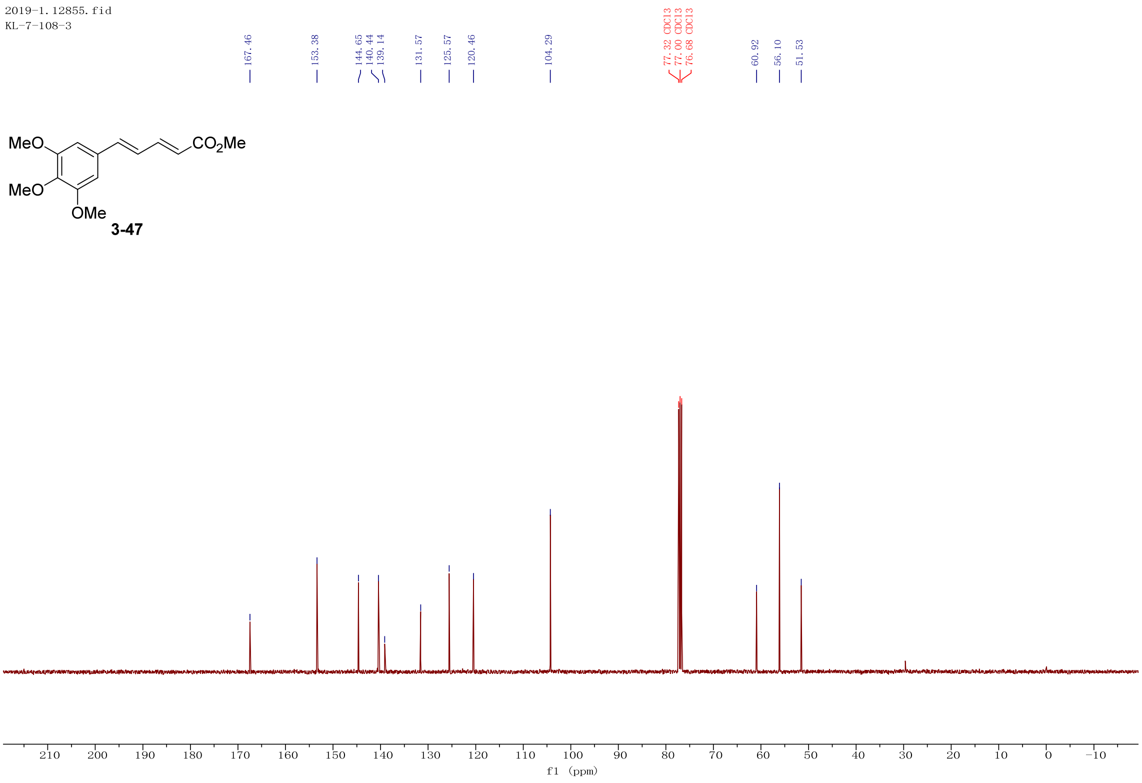

Supplementary Fig. 136

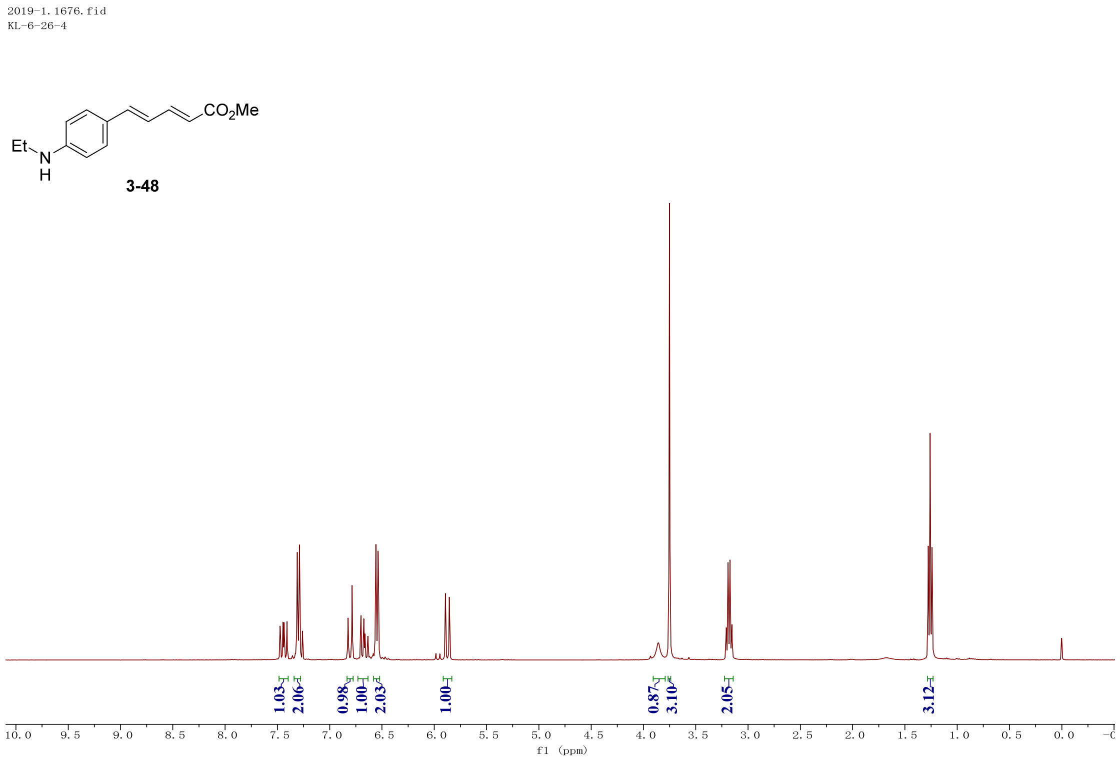

Supplementary Fig. 137

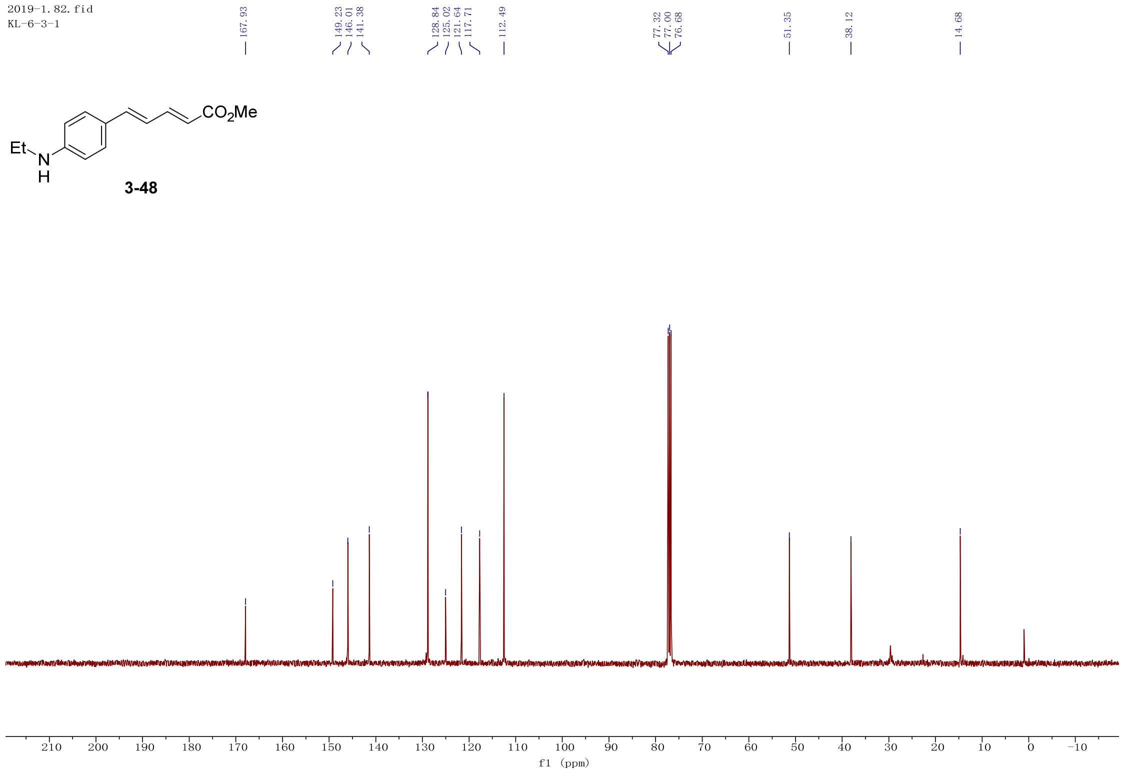

Supplementary Fig. 138

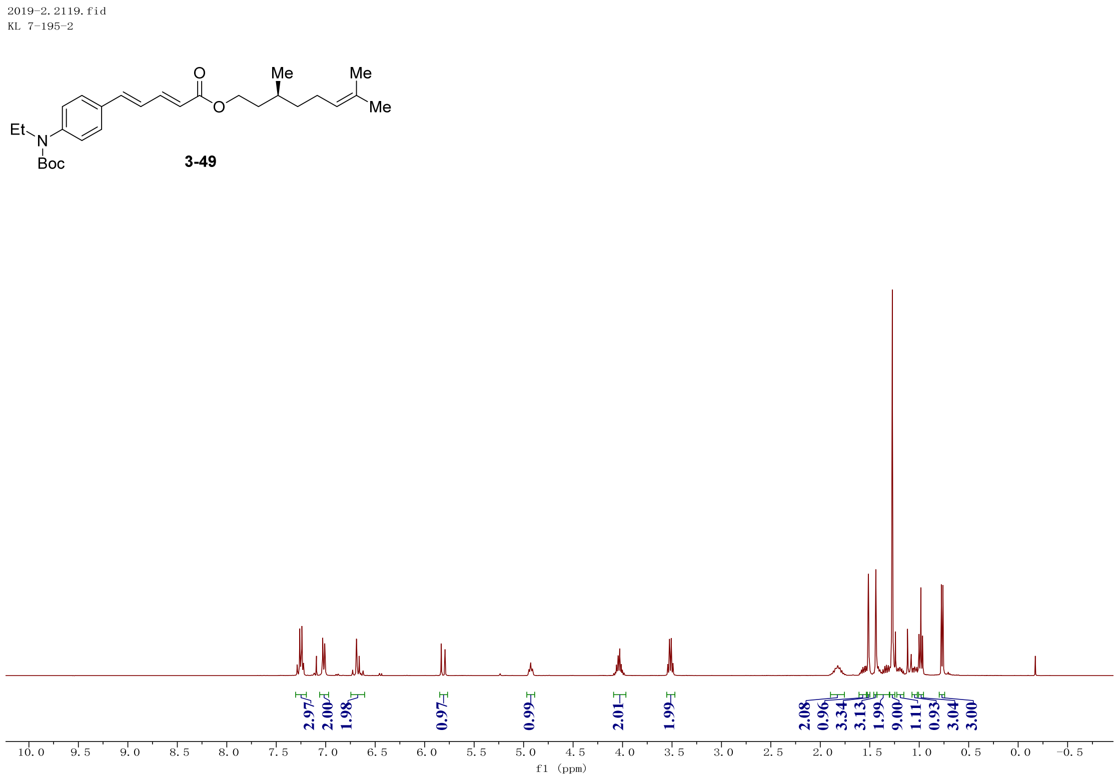

Supplementary Fig. 139

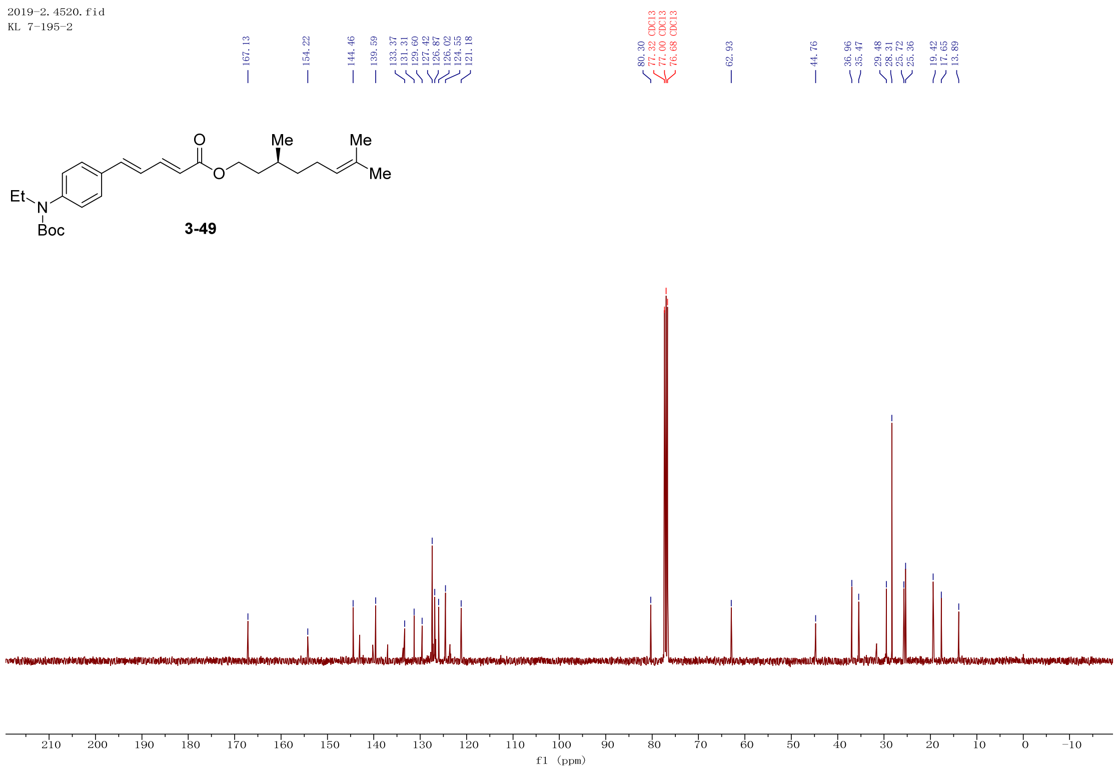

Supplementary Fig. 140

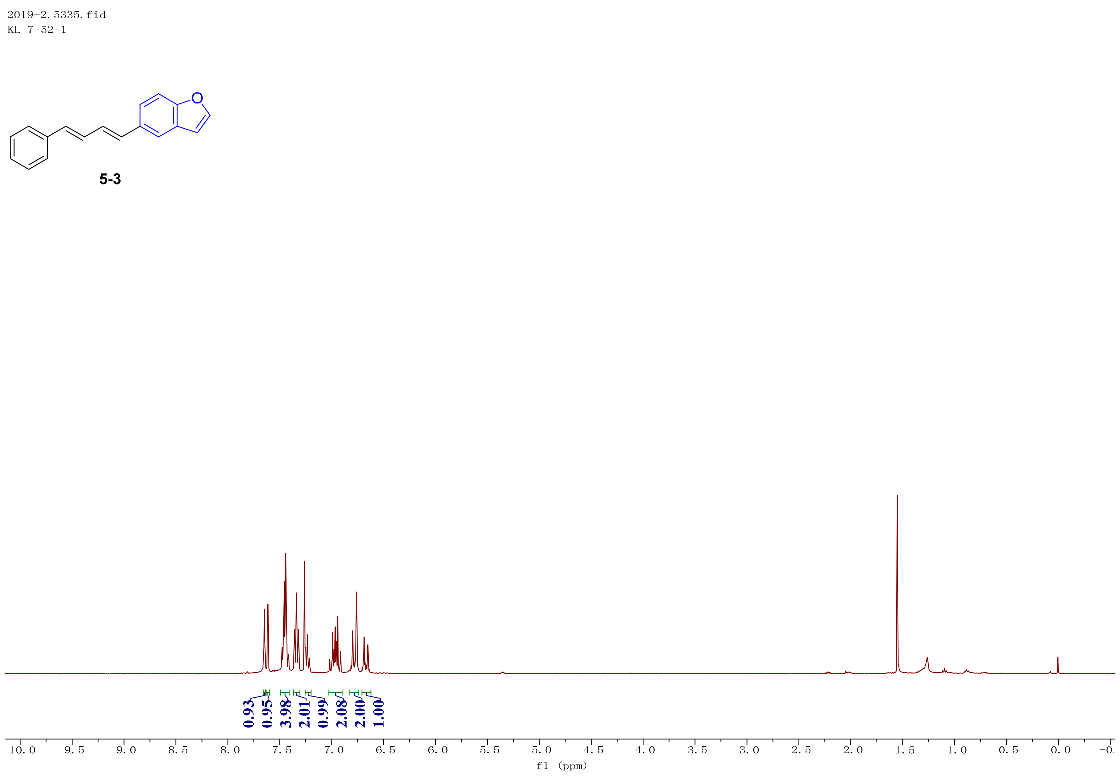

Supplementary Fig. 141

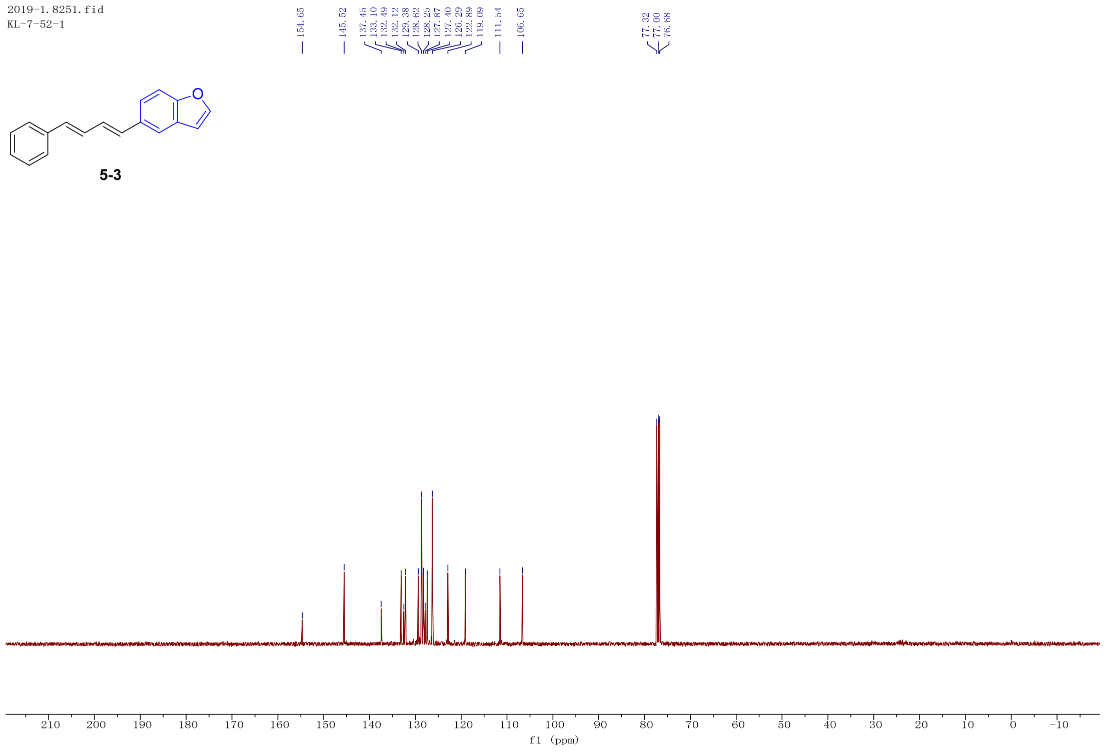

Supplementary Fig. 142

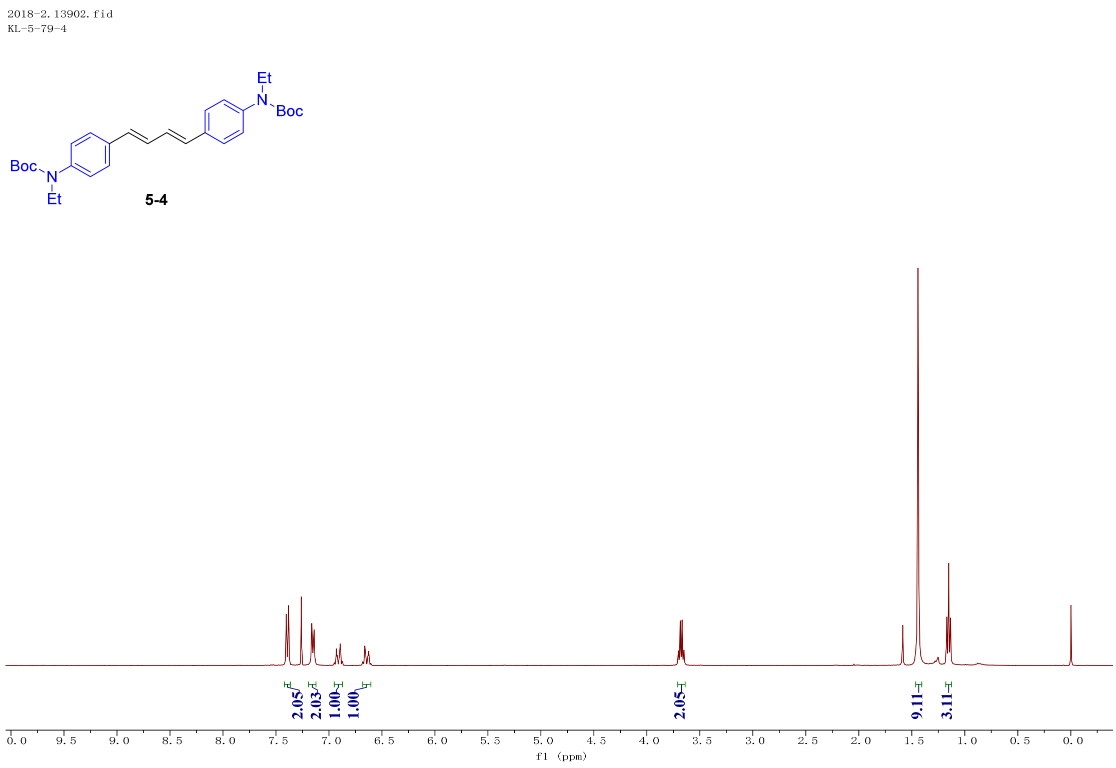

Supplementary Fig. 143

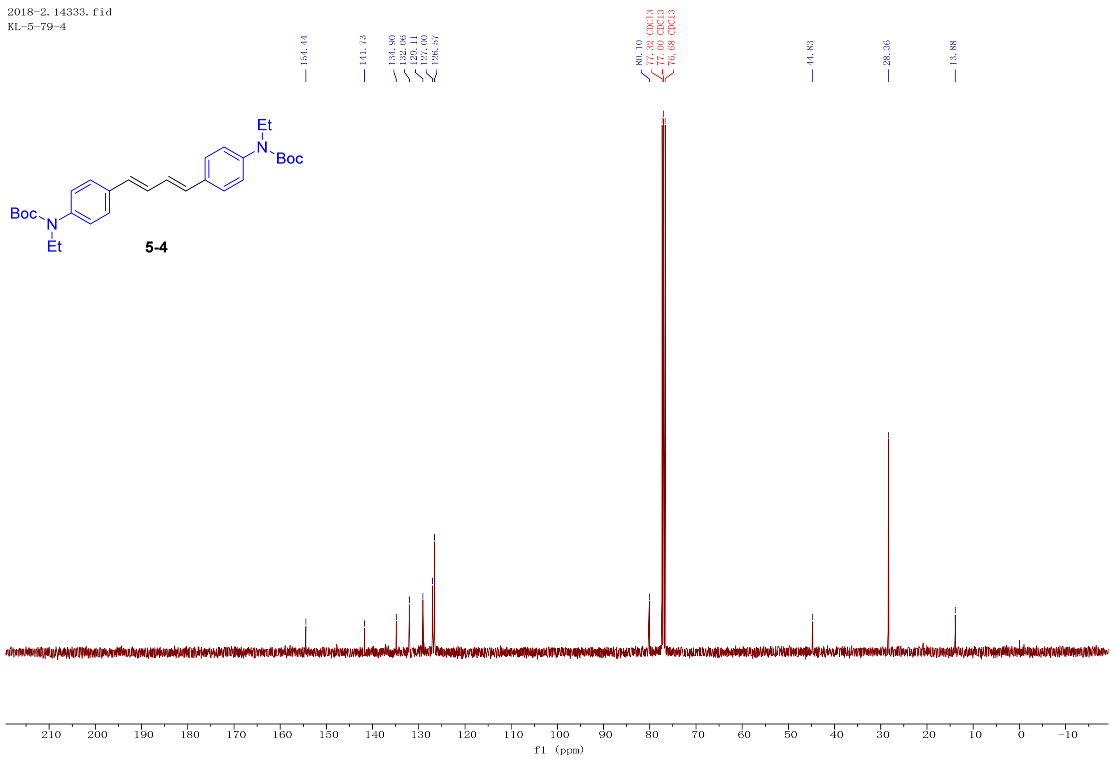

Supplementary Fig. 144

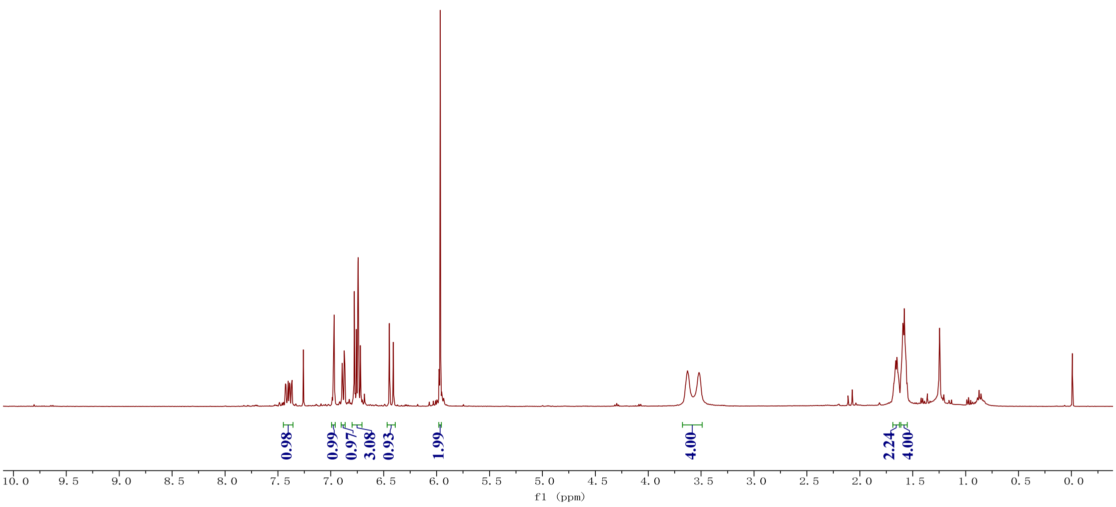

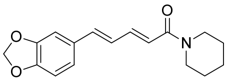

5-6, piperine

Supplementary Fig. 145

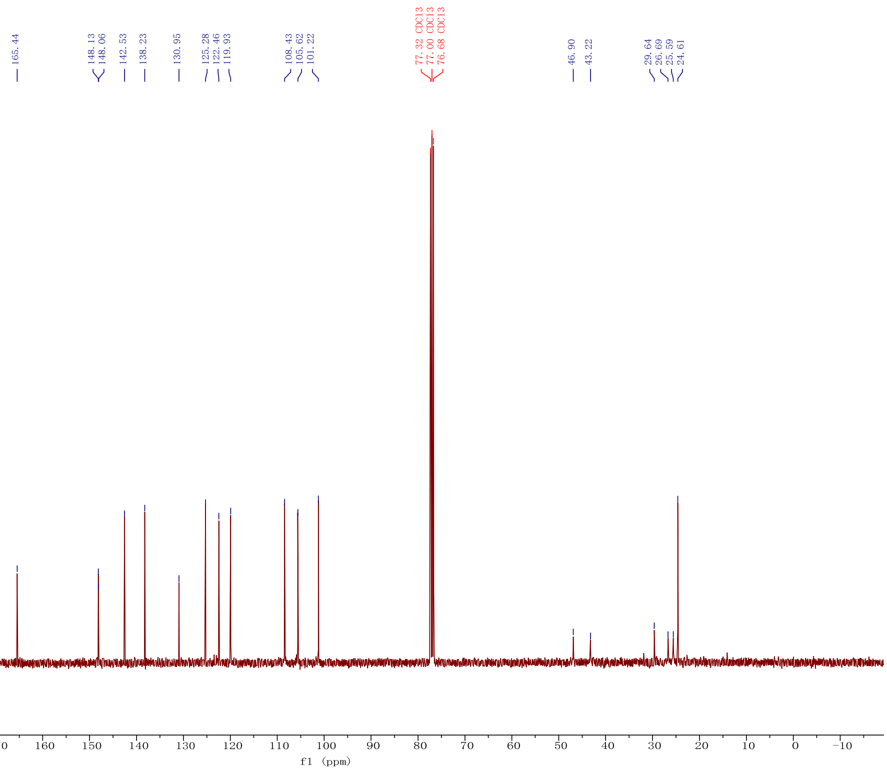

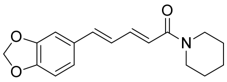

5-6, piperine

Supplementary Fig. 146

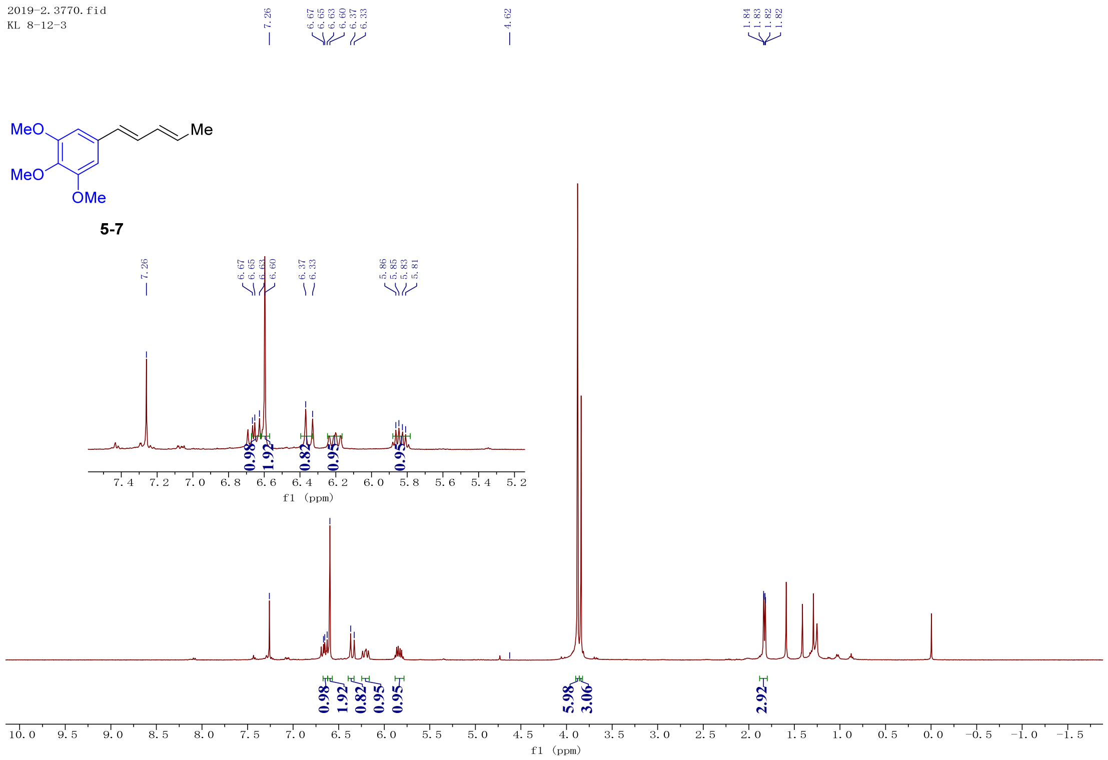

Supplementary Fig. 147

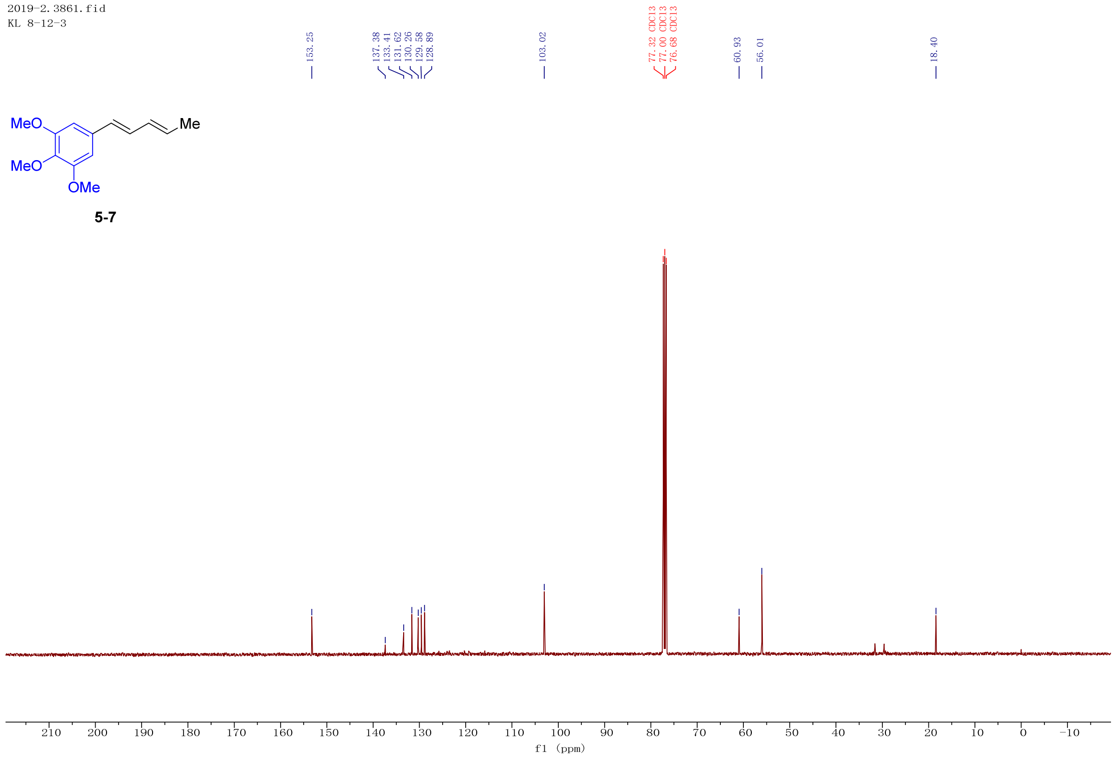

Supplementary Fig. 148

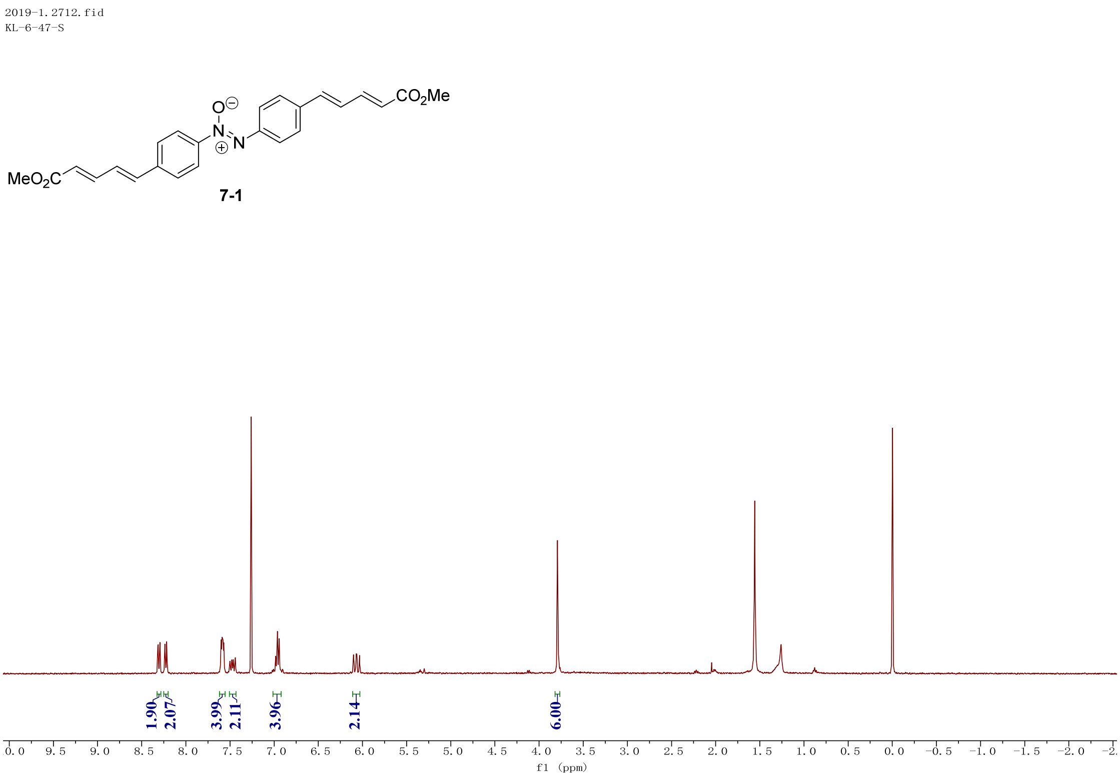

Supplementary Fig. 149

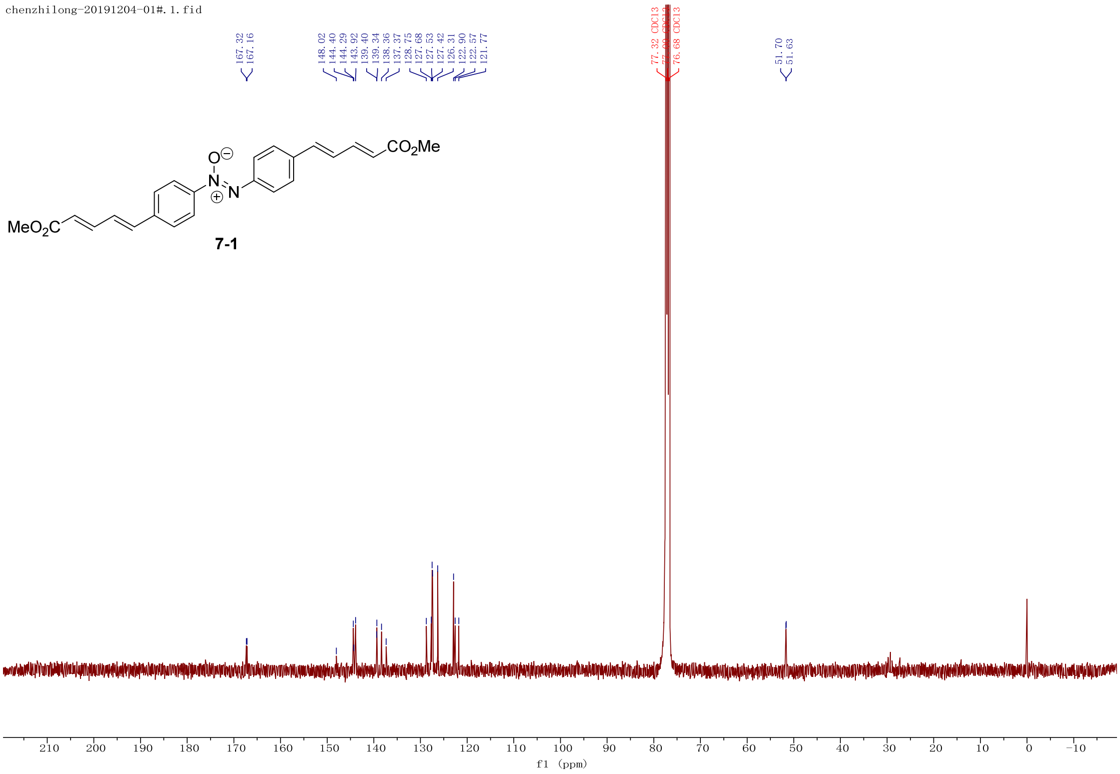

Supplementary Fig. 150

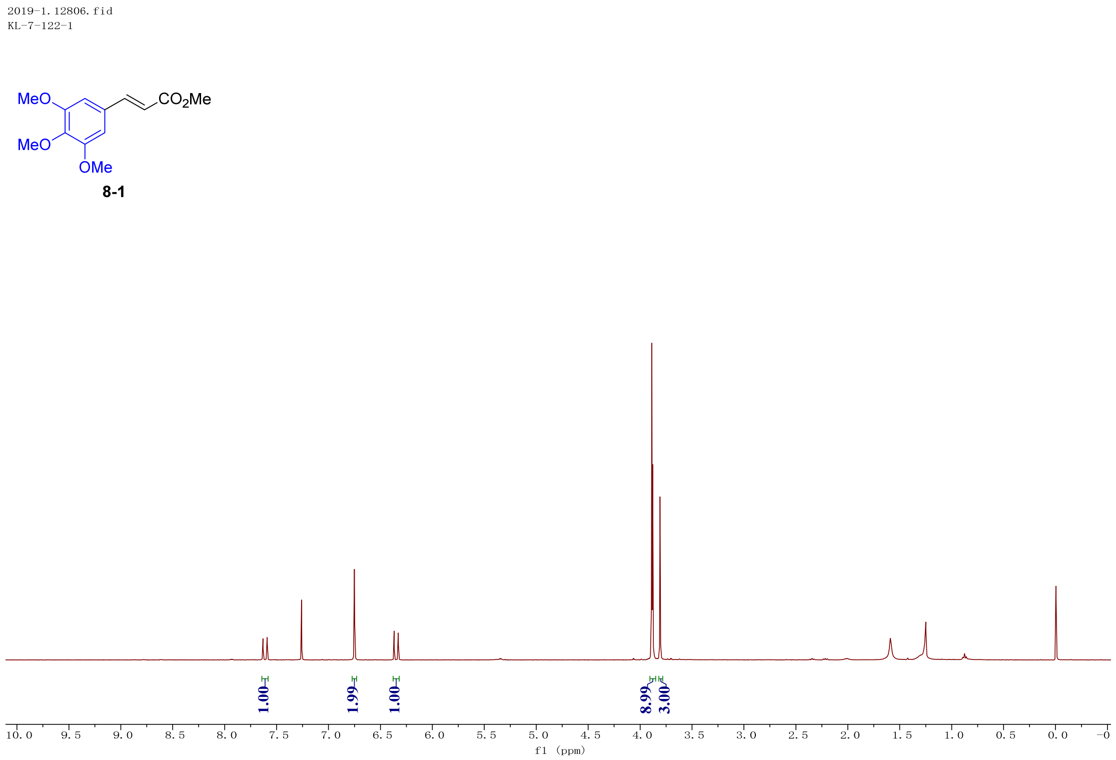

Supplementary Fig. 151

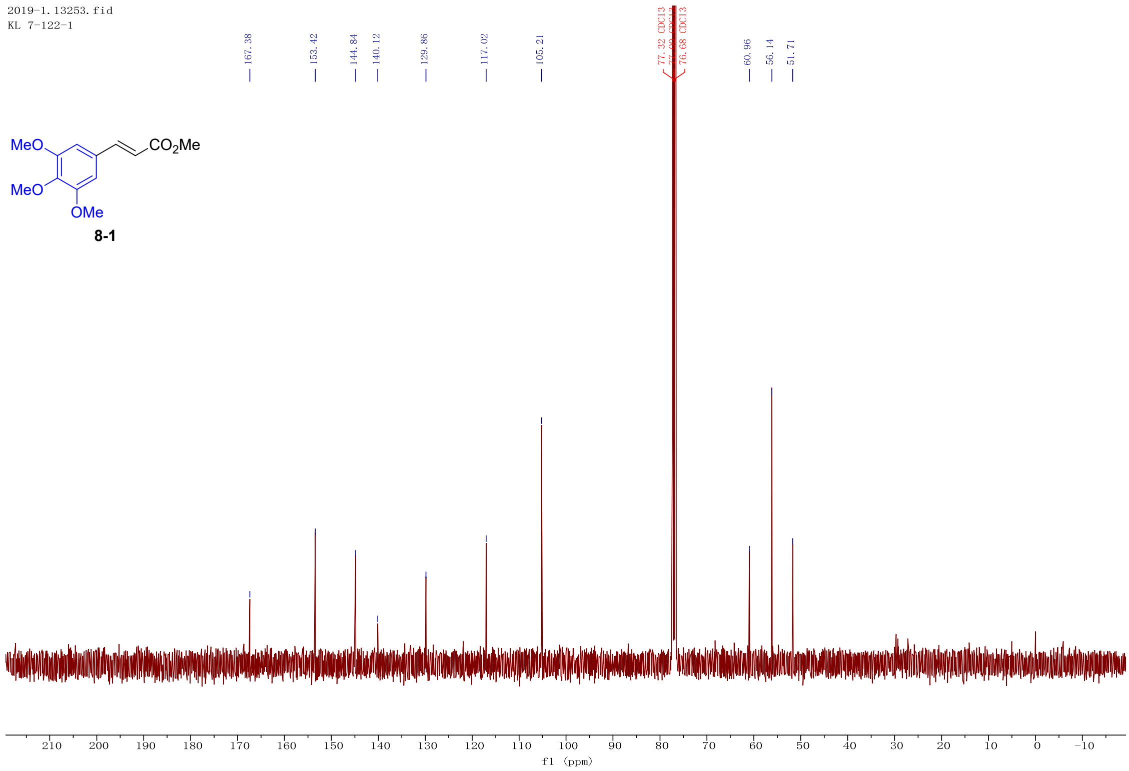

Supplementary Fig. 152
