# Supplementary material for: Dienedioic acid as a useful diene building block via directed Heck-decarboxylate coupling
Source: Commun Chem. 2020 Apr 20;3:48. doi: 10.1038/s42004-020-0295-0 (PMC9814911; doi:10.1038/s42004-020-0295-0)
Supplement: Supplementary file 2 — Description of Additional Supplementary Files [file 42004_2020_295_MOESM2_ESM.pdf]

Supplementary Data 1. Crystallographic information file for compound **3-2**.
